# Structure‐Based Design, Synthesis, and Biological Evaluation of Triazole‐Based smHDAC8 Inhibitors

**DOI:** 10.1002/cmdc.201900583

**Published:** 2020-01-09

**Authors:** Dmitrii V. Kalinin, Sunit K. Jana, Maxim Pfafenrot, Alokta Chakrabarti, Jelena Melesina, Tajith B. Shaik, Julien Lancelot, Raymond J. Pierce, Wolfgang Sippl, Christophe Romier, Manfred Jung, Ralph Holl

**Affiliations:** ^1^ Department of Chemistry Institute of Organic Chemistry University of Hamburg Martin-Luther-King-Platz 6 20146 Hamburg Germany; ^2^ German Center for Infection Research (DZIF) partner site Hamburg-Lübeck-Borstel-Riems; ^3^ Institute of Pharmaceutical and Medicinal Chemistry University of Münster Corrensstr. 48 48149 Münster Germany; ^4^ Cells-in-Motion Cluster of Excellence (EXC 1003 - CiM) University of Münster Schlossplatz 4 48149 Münster Germany; ^5^ NRW Graduate School of Chemistry University of Münster Wilhelm-Klemm-Str. 10 48149 Münster Germany; ^6^ Institute of Pharmaceutical Sciences Albert-Ludwigs-Universität Freiburg Albertstr. 25 79104 Freiburg Germany; ^7^ Institute of Pharmacy Martin Luther University of Halle-Wittenberg Wolfgang-Langenbeck Str. 4 06120 Halle/Saale Germany; ^8^ Département de Biologie Structurale Intégrative Institut de Génétique et Biologie Moléculaire et Cellulaire (IGBMC) Université de Strasbourg, CNRS, INSERM 1 rue Laurent Fries 67404 Illkirch Cedex France; ^9^ Université de Lille, CNRS, Inserm, CHU Lille, Institut Pasteur de Lille U1019-UMR 8204-CIIL-Centre d'Infection et d'Immunité de Lille 59000 Lille France

**Keywords:** *Schistosoma mansoni*, histone deacetylases, triazole derivatives, crystal structures, molecular docking studies

## Abstract

Schistosomiasis is a neglected tropical disease caused by parasitic flatworms of the genus *Schistosoma*, which affects over 200 million people worldwide and leads to at least 300,000 deaths every year. In this study, initial screening revealed the triazole‐based hydroxamate **2 b** (*N*‐hydroxy‐1‐phenyl‐1*H*‐1,2,3‐triazole‐4‐carboxamide) exhibiting potent inhibitory activity toward the novel antiparasitic target *Schistosoma mansoni* histone deacetylase 8 (smHDAC8) and promising selectivity over the major human HDACs. Subsequent crystallographic studies of the **2 b**/smHDAC8 complex revealed key interactions between the inhibitor and the enzyme's active site, thus explaining the unique selectivity profile of the inhibitor. Further chemical modifications of **2 b** led to the discovery of 4‐fluorophenoxy derivative **21** (1‐[5‐chloro‐2‐(4‐fluorophenoxy)phenyl]‐*N*‐hydroxy‐1*H*‐1,2,3‐triazole‐4‐carboxamide), a nanomolar smHDAC8 inhibitor (IC_50_=0.5 μM), exceeding the smHDAC8 inhibitory activity of **2 b** and SAHA (vorinostat), while exhibiting an improved selectivity profile over the investigated human HDACs. Collectively, this study reveals specific interactions between smHDAC8 and the synthesized triazole‐based inhibitors and demonstrates that these small molecules represent promising lead structures, which could be further developed in the search for novel drugs for the treatment of schistosomiasis.

## Graphical abstract

**Triazole‐based smHDAC8 inhibitors** exhibiting an improved selectivity profile over human HDACs were synthesized, evaluated in *in vitro* inhibition assays, and tested for their effects on the viability of *Schistosoma mansoni* larvae. Crystallographic as well as molecular docking studies revealed key interactions between smHDAC8 and the developed triazole derivatives, thus explaining their unique selectivity profile.

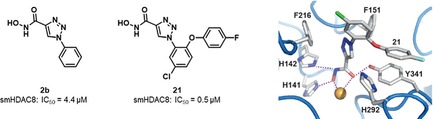

## Introduction

Schistosomiasis, or bilharzia, is a neglected tropical disease, which is caused by the trematode *Schistosoma mansoni* and other platyhelminth parasites of the same genus.^1^, ^2^, ^3^ The disease is prevalent in Africa, the Middle East, South America, and Asia, affecting over 200 million people worldwide and causing at least 300,000 deaths every year.^4^, ^5^, ^6^ Currently, praziquantel is the only drug available for treatment and control of schistosomiasis.^7^ The intensive use of this drug increases the probability of the emergence of praziquantel resistant parasite strains and worrisome data on reduced efficacy of the drug have already been reported, thus rendering the search for potential drug targets as well as novel drugs a strategic priority.^5^, ^8^, ^9^, ^10^

**Figure 1:**
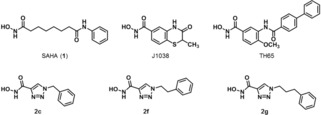
Chemical structures of pan‐HDAC inhibitor SAHA (vorinostat, **1**), smHDAC8 inhibitors J1038 and TH65, and triazole derivatives **2 c**, **2 f**, and **2 g**.

The treatment of *S. mansoni* with small‐molecule histone deacetylase (HDAC) inhibitors was shown to cause dose‐dependent mortality of schistosomula as well as adult worms, making HDACs potential targets for the treatment of schistosomiasis.^11^, ^12^, ^13^ In eukaryotes, HDACs, which belong to the epigenetic machinery of the cells, catalyze the deacetylation of ϵ‐amino groups of lysine residues in histone tails, leading in consequence to a more compact chromatin structure, which usually results in an inhibition of transcription.^14^, ^15^, ^16^, ^17^ Being drug targets in cancer therapy, human histone deacetylases (hsHDACs) were intensively studied and various HDAC inhibitors, like e. g. SAHA (**1**, Figure 1), were described.^18^, ^19^, ^20^, ^21^ The 18 human HDACs, which have been discovered so far, are grouped into 4 classes.^18^, ^22^ Whereas classes I, II, and IV comprise the Zn^2+^‐dependent HDACs, the class III enzymes require NAD^+^ for catalysis. In *S. mansoni*, three class I HDACs are known, representing orthologues of the human class I enzymes HDAC1, HDAC3, and HDAC8.^12^ These three *S. mansoni* class I HDACs are expressed in the parasite at all stages of its life‐cycle.^11^ In contrast to hsHDAC8, showing in humans the lowest level of expression of the class I enzymes, in *S. mansoni* smHDAC8 is the most abundantly expressed class I HDAC at all life‐cycle stages and was validated as drug target for schistosome‐specific inhibitors. Down‐regulation of smHDAC8 expression in schistosomula caused a decrease in their capacity to survive and mature in infected mice. In addition, the tissue egg burden was reduced by 45 %.^5^, ^12^, ^23^ Like its human orthologue, smHDAC8 folds into a single α/β domain being composed of a central parallel β‐sheet, which is sandwiched between several α‐helices.^5^, ^6^, ^24^ The active sites of the enzymes consist of a long narrow tunnel, accommodating the incoming acetylated lysine side chain of the substrate, which leads to a cavity containing the catalytic Zn^2+^‐ion. The active site residues of the two enzymes are highly conserved, with only M274 in hsHDAC8, being substituted by H292 in smHDAC8.^6^ The replacement of this hydrophobic residue by a polar one modifies the physicochemical properties of the active site, which could be exploited for the development of smHDAC8‐specific inhibitors.^5^, ^6^ Additionally, at the entrance region of the binding tunnel, F151 of smHDAC8 (corresponding to F152 in hsHDAC8) can adopt both a flipped‐in and a flipped‐out conformation, whereas in hsHDACs due to steric constriction, only the flipped‐in conformation of this highly conserved residue has been observed so far. The flipped‐out conformation of F151 leads to a wider catalytic pocket in smHDAC8, which hence is able to accommodate bulkier inhibitors.^5^, ^6^ These differences should allow the development of inhibitors that are selective for the schistosome enzyme, thereby minimizing off‐target effects caused by interactions with the human (host) orthologues.^25^, ^26^ A few smHDAC8 inhibitors have been described in the literature so far, such as J1038 and TH65 (Figure 1).^5^, ^27^, ^28^, ^29^, ^30^ These inhibitors are often aromatic hydroxamic acids and many exploit a hydrogen bond to the aforementioned histidine in the active site, whereas the methionine, which the human orthologue has in the same place, cannot be addressed in a similar fashion.

Several triazole derivatives like **2 c**, **2 f**, and **2 g** (Figure 1) have been reported to weakly inhibit hsHDAC1 and hsHDAC8.^31^ As these hydroxamic acids contain a polar triazole ring, which could possibly interact with H292 of smHDAC8, these compounds, along with other triazole derivatives, exhibiting further variations of the substituent in position 1 of the heterocycle, were synthesized, assayed for their inhibitory activity toward smHDAC8, and tested for their selectivity toward hsHDAC1 and hsHDAC8 as well as the class IIb histone deacetylase hsHDAC6.

## Results and Discussion

### Synthesis of triazole derivatives 2 b‐j

The reported triazole derivatives **2 c**, **2 f**, and **2 g** comprise a phenyl ring linked to the triazole core via a flexible alkyl chain (from one to three methylene groups, respectively). To vary length and flexibility of the side chain, besides phenyl derivative **2 b** lacking a methylene linker, diphenylacetylene and diphenylbutadiyne derivatives **2 h‐j** exhibiting a rigidified side chain should be synthesized and tested for their inhibitory profile. Furthermore, to study the effect of an additional substituent in the *para*‐position of the terminal phenyl ring, triazole derivatives **2 d**, **2 e**, and **2 i** should be accessed.

**Scheme 1:**
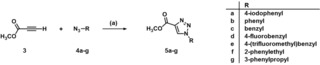
Reagents and conditions: (a) sodium ascorbate, CuSO_4_ ⋅ 5 H_2_O, *t*BuOH : H_2_O=1 : 1, RT, 16 h, **5 a** 98 %, **5 b** 85 %, **5 c** 96 %, **5 d** 95 %, **5 e** 93 %, **5 f** 85 %, **5 g** 78 %.

The synthesis of triazole derivatives **2 b**, **2 c**, **2 f**, and **2 g** had been reported in the literature before.^31^, ^32^, ^33^ However, the envisaged triazole derivatives were synthesized via an alternative route, requiring fewer steps compared to the described procedures. In the first step of the synthetic route, the triazole ring was established by performing Cu(I)‐catalyzed [3+2]‐cycloadditions.^34^ The reaction of methyl propiolate (**3**) with various azides (**4 a**–**g**)^31^, ^35^, ^36^, ^37^, ^38^, ^39^ was carried out at ambient temperature in a 1 : 1 mixture of water and *tert*‐butanol in the presence of copper(II) sulfate and sodium ascorbate giving access to triazole derivatives **5 a**–**g** (Scheme 1).

**Scheme 2:**
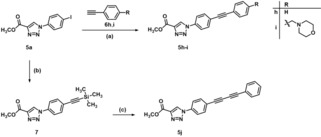
Reagents and conditions: (a) Pd(PPh_3_)_4_, CuI, Et_3_N, DMF, 70 °C, 16 h, **5 h** 96 %, **5 i** 98 %; (b) trimethylsilylacetylene, Pd(PPh_3_)_4_, CuI, Et_3_N, CH_3_CN, 70 °C, 3 h, 97 %; (c) i) TBAF, CH_2_Cl_2_, rt, 30 min, ii) phenylacetylene, Cu(OAc)_2_, pyridine, MeOH, rt, 16 h, 62 %.

In order to further vary the substituent in position 1 of the triazole ring by establishing a linear and rigid side chain, C−C coupling reactions with 4‐iodophenyl derivative **5 a** were performed (Scheme 2). Sonogashira couplings with phenylacetylene (**6 h**) and its morpholin‐4‐ylmethyl‐substituted derivative **6 i** 40 were conducted in a mixture of triethylamine and DMF at 70 °C in the presence of Pd(PPh_3_)_4_ and CuI to obtain diphenylacetylene derivatives **5 h** and **5 i**, respectively.^41^

Additionally, the butadiyne derivative **5 j** was synthesized employing the Eglinton reaction (Scheme 2).^42^ At first, an acetylene moiety was introduced to the phenyl ring of triazole derivative **5 a**. Performing a Sonogashira coupling with trimethylsilylacetylene resulted in acetylene derivative **7**. In the next step, the trimethylsilyl protecting group of **7** was cleaved off with tetrabutylammonium fluoride (TBAF). As TLC indicated a clean conversion of trimethylsilyl protected compound **7** into the corresponding terminal alkyne, after aqueous work up, the crude product of the reaction was directly subjected to a subsequent Eglinton reaction. This coupling reaction was performed with phenylacetylene in a mixture of methanol and pyridine in the presence of copper(II) acetate giving access to diacetylene derivative **5 j**.

**Scheme 3:**
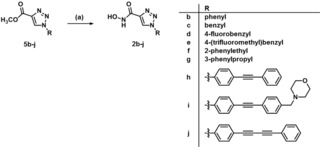
Reagents and conditions: (a) NH_2_OH ⋅ HCl, NaOMe, MeOH, rt, 20 h, **2 b** 66 %, **2 c** 72 %, **2 d** 55 %, **2 e** 48 %, **2 f** 67 %, **2 g** 61 %, **2 h** 91 %, **2 i** 76 %, **2 j** 68 %.

In the final step, the ester moiety of triazole derivatives **5 b**–**j** was transformed into a Zn^2+^‐chelating hydroxamate moiety. Therefore, the esters were reacted with hydroxylamine hydrochloride and sodium methanolate in dry methanol to obtain hydroxamic acids **2 b**–**j** (Scheme 3).

### HDAC inhibitory activity of triazole derivatives 2 b–j

All of the synthesized triazole derivatives were pretested for inhibitory activity against smHDAC8. The most active compounds (≥50 % inhibition at 10 μM) were further analyzed and their IC_50_ values against smHDAC8 and hsHDAC8 were determined in a homogenous fluorescence assay using a commercially available oligopeptide (Fluor de Lys) with an acetyl residue, which had also been employed for pretesting. Additionally, the inhibitory activity of the selected compounds was tested against hsHDAC1 and hsHDAC6, using ZMAL with an acetylated lysine as substrate.^43^

Pretesting of triazole derivatives **2 b**–**j** at a concentration of 10 μM revealed phenyl‐substituted triazole **2 b**, trifluoromethylbenzyl derivative **2 e**, phenylethyl‐ and phenylpropyl‐substituted compounds **2 f** and **2 g** to be the most potent smHDAC8 inhibitors of this series of hydroxamic acids (Table 1). Whereas benzyl derivatives **2 c** and **2 d** as well as the morpholinomethyl substituted compound **2 i** showed only slightly weaker inhibition of smHDAC8, compounds **2 h** and **2 j**, possessing an unpolar, linear, and rigid side chain, were found to exhibit nearly no inhibitory activity against the enzyme.

**Table 1:** *In vitro* inhibition of smHDAC8, hsHDAC8, hsHDAC6 and hsHDAC1. The employed substrate is given in brackets. nd: not determined. Number of replicates: all pretests n=1; all IC_50_ n=2.

compound	smHDAC8 (% inhibition, Fluor de Lys)	smHDAC8 IC_50_ [μM] (Fluor de Lys)	hsHDAC8 IC_50_ [μM] (Fluor de Lys)	hsHDAC6 IC_50_ [μM] (ZMAL)	hsHDAC1 (% inhibition, ZMAL)
SAHA (**1**)	72.0 @ 10 μM	1.17±0.33	0.64±0.21	0.14±0.12	97.3±1.3 @ 10 μM
**2 b**	57.9 @ 10 μM	4.44±1.52	12.43±1.08	42.50±3.64	8.1±1.0 @ 10 μM
**2 c**	39.6 @ 10 μM	nd	nd	nd	nd
**2 d**	36.1 @ 10 μM	nd	nd	nd	nd
**2 e**	52.5 @ 10 μM	5.12±0.64	3.47±0.53	2.2±0.5 % @ 10 μM	3.3±2.5 @ 10 μM
**2 f**	49.5 @ 10 μM	10.67±1.88	5.54±0.64	12.6±9.6 % @ 10 μM	1.7±3.4 @ 10 μM
**2 g**	50.5 @ 10 μM	8.81±1.89	5.37±0.68	31.79±6.18	5.4±7.0 @ 10 μM
**2 h**	‐9.2 @ 10 μM	nd	nd	nd	nd
**2 i**	48.0 @ 10 μM	nd	nd	nd	nd
**2 j**	6.8 @ 10 μM	nd	nd	nd	nd

The IC_50_ values of the most potent compounds against smHDAC8 were in the low micromolar range, with phenyl derivative **2 b** being the most potent inhibitor, possessing an IC_50_ value of 4.44 μM.

When being tested for hsHDAC8 inhibition, phenylalkyl derivatives **2 e**, **2 f**, and **2 g** showed slightly increased inhibitory activity against the human orthologue. In contrast, phenyl derivative **2 b** was found to inhibit hsHDAC8 with a higher IC_50_ value, exhibiting a threefold selectivity for smHDAC8. Against hsHDAC1 and hsHDAC6 all compounds exhibited considerably lower inhibitory activities.

### smHDAC8/2 b X‐ray crystal structure

**Figure 2:**
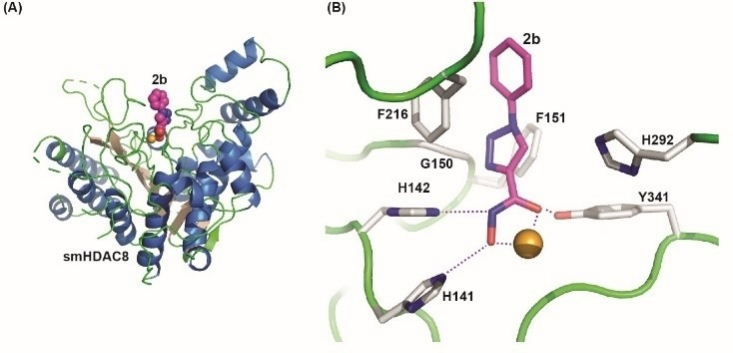
Crystal structure of the smHDAC8/**2 b** complex (PDB ID 6TLD). (A) Overall view of the complex. The protein is shown as ribbons whereas the catalytic zinc (orange) and inhibitor **2 b** (carbon, magenta; nitrogen, blue; oxygen, red) atoms are shown as spheres. **2 b** adopts a canonical pan‐HDAC binding mode with a straight conformation, its capping group pointing toward the solvent. (B) Close‐up view of **2 b** binding into the active site of smHDAC8. **2 b** (magenta carbon atoms) and specific side chains of smHDAC8 (grey carbon atoms) interacting with the inhibitor are shown as sticks (nitrogen, blue; oxygen, red). Interactions are shown with broken lines. Residues are labelled.

Due to the promising biological activity of the 1‐phenyl substituted triazole derivative **2 b**, the compound was crystallized in complex with smHDAC8 (Figure 2, Table S1, Supporting Information). The structure reveals that compound **2 b** binds in the smHDAC8 active site in a straight conformation, its capping group pointing toward the solvent, whereas its hydroxamate moiety forms a bidentate interaction with the catalytic zinc ion as well as hydrogen bonds with the side chains of histidines H141 and H142 and the hydroxyl group of catalytic tyrosine Y341. This binding mode is reminiscent of those of many pan‐HDAC inhibitors binding to HDACs. Interestingly, the triazole ring of **2 b** is observed almost stacked between the side chains of phenylalanines F151 and F216 that form the active site tunnel normally accommodating the aliphatic part of the incoming acetylated lysine side chain. Yet, the F151 side chain is observed in two conformations, one turned toward the active site, participating to the tunnel mentioned previously, and one turned away from the active site. This suggests that the stacking of the triazole ring between the two phenylalanines observed in the structure is not absolutely essential for the interaction. In addition, the nitrogen atoms of the triazole ring are not facing the smHDAC8‐specific H292 side chain but turned toward the Cα carbon atom of glycine G150. Finally, the capping group of **2 b** does not appear to make extensive interactions with smHDAC8. Yet, the rather small size of the inhibitor allows it to fit nicely into the smHDAC8 active site pocket. Replacement of smHDAC8 H292 by the somewhat bulkier human methionine M274 could potentially explain the increased IC_50_ for **2 b** measured with the human enzyme.

### Docking of the synthesized triazole derivatives and design of novel inhibitors

**Figure 3:**
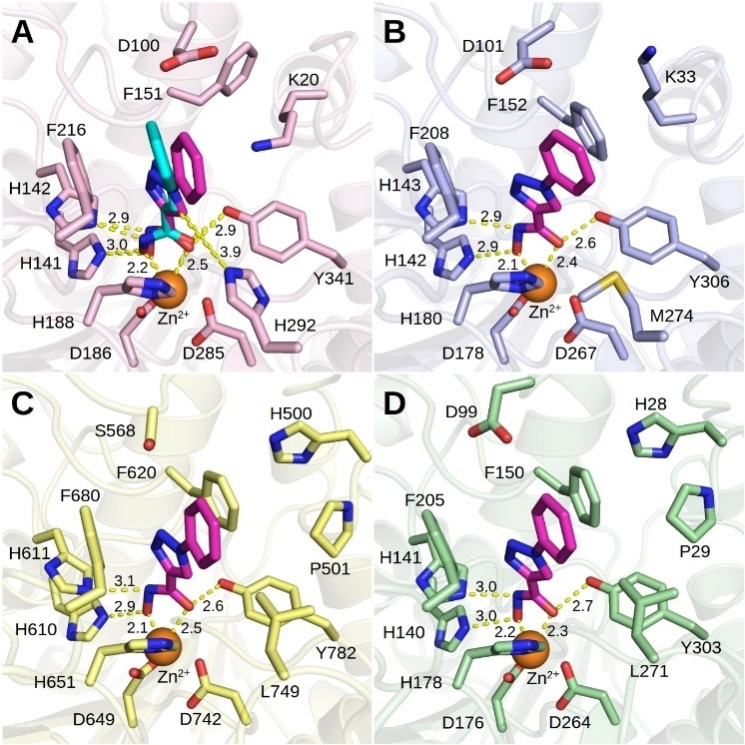
Binding modes of compound **2 b** (predicted – magenta carbon atoms, experimental – cyan carbon atoms) in: (A) smHDAC8 (pale pink carbons), (B) hsHDAC8 (pale blue carbons), (C) hsHDAC6 (pale yellow carbons), and (D) hsHDAC1 (pale green carbons). Catalytic zinc ion is shown as orange sphere. Nitrogens are colored dark blue, oxygens – red, sulfur – dark yellow. Metal interactions and hydrogen bonds are shown as dashed yellow lines with distances in Å.

Although being a slightly less potent smHDAC8 inhibitor compared to pan‐HDAC inhibitor SAHA (**1**), the phenyl‐substituted triazole derivative **2 b** was considered as a promising lead structure due to its selectivity toward the platyhelminth enzyme. Analysis of possible binding modes of this compound in different HDAC isoforms helped to rationalize the observed selectivity. The crystal structure of **2 b** with smHDAC8 showed that the inhibitor exhibits a classical binding mode characteristic to many other hydroxamic acid‐based HDAC inhibitors.^44^ Namely, its zinc‐binding group chelates the catalytic zinc ion in bidentate fashion and interacts with conserved residues H141, H142, and Y341. The triazole linker is placed in the lysine‐binding channel while the phenyl ring is pointed to the entrance of the pocket. Interestingly molecular docking studies suggested that a similar binding mode of this ligand could be possible also in human HDAC isoforms HDAC8, HDAC1, and HDAC6 (Figure 3).

Despite similar binding modes, slight differences of the binding pockets could explain the selectivity of **2 b**. Analysis of the binding mode of **2 b** in smHDAC8 suggested a number of possible interactions. As seen in Figure 3 A, the triazole ring of the compound makes a weak hydrogen bond with the smHDAC8 unique residue H292 and it is able to form an aromatic interaction with its imidazole ring. Similar interactions have been also reported in the literature.^45^, ^46^ Furthermore, the triazole ring of **2 b** forms a π‐cation interaction with K20 of smHDAC8 whereas in the case of the human enzyme the corresponding K33 is flipped out of the binding pocket (Figure 3 B). Furthermore, in hsHDAC8 an H‐bond interaction of the triazole ring of **2 b** with M274 was not observed, probably due to non‐optimal orientation of this residue and weaker hydrogen bond accepting properties of M274. It was also observed that the favorable orientation of the triazole ring in smHDAC8 allows π‐stacking interaction between the phenyl ring of **2 b** and F216, whereas in hsHDAC8 this interaction with corresponding F208 is not detected. The lack of interactions between the triazole and surrounding amino acid residues and the absence of π‐stacking interaction between the phenyl ring and Phe208 in hsHDAC8 explains why the compound is slightly selective toward smHDAC8. In hsHDAC6 and hsHDAC1 (Figure 3 C–D) the bulkier L749/L271 residues are responsible for a more closed side pocket and, in addition, no H‐bond interactions can be formed between the triazole and the leucine residue. Therefore, the inhibitory activity of **2 b** for hsHDAC1/6 is reduced.

To understand the contribution of the cap group to the activity of the compounds, docking poses of **2 c**‐**j** were analyzed (Figure S1, Supporting Information). It was observed that the benzyl, 4‐fluorobenzyl, and 4‐(trifluoromethyl)benzyl caps of compounds **2 c‐e** do not show any direct interactions with surrounding residues. The phenylethyl and phenylpropyl groups of **2 f** and **2 g** are able to form aromatic interactions with F216 or Y341. Similar binding modes are observed for these compounds in hsHDAC8. In the series of compounds with a larger and rigid scaffold (**2 h‐j**), all three ligands fitted into the smHDAC8 binding pocket, but a significant part of the compound was sticking out into the solvent. The more active compound **2 i** formed favorable cation‐π interactions between its positively charged nitrogen of the morpholine scaffold and the aromatic ring of the Y99 amino acid residue.

In order to improve the affinity toward smHDAC8 and to further enhance the selectivity of the triazole derivatives, molecular docking studies of various suggested derivatives of **2 b** were performed utilizing the obtained crystal structure of smHDAC8 with this compound. Based on these studies, further 1‐phenyl substituted triazole derivatives were envisaged possessing a phenyl ether or a benzophenone moiety. The substitutions were introduced to the *ortho*‐position of the phenyl ring to address the unique side pocket of HDAC8 with L‐shaped inhibitors. As shown by us previously, this should further increase selectivity against HDAC6 and HDAC1.^28^ Also since the side pockets of smHDAC8 and hsHDAC8 are different, we hoped to further increase selectivity against hsHDAC8. Thus, on the one hand, these substituents should lead to better interactions with smHDAC8. On the other hand, they should render the relatively polar triazole derivatives more lipophilic, as it is established, that a logP above 2.5 is beneficial for antiparasitic activity.^27^

Molecular docking studies also showed that an additional small substituent (e. g. chlorine) at position 5 of the aromatic ring fits to the pocket and stabilizes the rotation of the ring required for the expected orientation of the *ortho*‐substituent (pushes the ligand into the side pocket). Introduction of a small substituent to the *meta*‐position without *ortho*‐substitution or with a small substituent at *ortho*‐position does not induce the rotation of the ring. Therefore, it was suggested to simultaneously introduce chlorine to position 5 of the phenyl ring in addition to a large aromatic substituent in position 2.

### Synthesis of *ortho*‐substituted 1‐phenyl‐1*H*‐1,2,3‐triazole derivatives

**Scheme 4:**
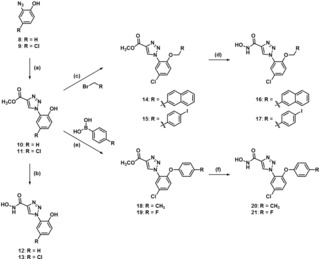
Reagents and conditions: (a) methyl propiolate, sodium ascorbate, CuSO_4_ ⋅ 5 H_2_O, *t*BuOH : H_2_O=1 : 1, rt, **10** 92 %, **11** 89 %; (b) NH_2_OH ⋅ HCl, NaOMe, MeOH, rt, **12** 45 %, **13** 43 %; (c) Cs_2_CO_3_, DMF, 90 °C, **14** 56 %, **15** 59 %; (d) NH_2_OH ⋅ HCl, NaOMe, MeOH, rt, **16** 83 %, **17** 52 %; (e) Cu(OAc)_2_, CH_2_Cl_2_, Et_3_N, rt, **18** 11 %, **19** 13 %; (f) NH_2_OH ⋅ HCl, NaOMe, MeOH, rt, **20** 99 %, **21** 95 %.

The envisaged ether derivatives were synthesized from azides **8** and **9** (Scheme 4). Copper(I)‐catalyzed [3+2]‐cycloadditions of azides **8** and **9** with methyl propiolate led to triazole derivatives **10** and **11**. Phenols **10** and **11** were subsequently subjected to an aminolysis with hydroxylamine, which gave hydroxamic acids **12** and **13**, bearing a polar substituent in position 2 of the phenyl ring. Alternatively, phenol derivative **11** was subjected to Williamson ether syntheses giving access to phenyl alkyl ethers **14** and **15**. The obtained ethers were finally reacted with hydroxylamine to yield hydroxamic acids **16** and **17**. Additionally, Chan‐Lam couplings of phenol **11** with 4‐tolylboronic acid and 4‐fluorophenylboronic acid were performed, leading to diphenyl ethers **18** and **19**,^47^ which were finally transformed into hydroxamic acids **20** and **21**. The synthesis of the latter compound has been briefly reported by us in a previous publication.^28^

**Scheme 5:**
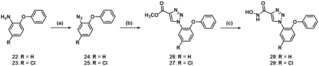
Reagents and conditions: (a) i) NaNO_2_, HCl, H_2_O, 0 °C, ii) NaN_3_, NaOAc, **24** 83 %, **25** 63 %; (b) methyl propiolate, sodium ascorbate, CuSO_4_ ⋅ 5 H_2_O, *t*BuOH : H_2_O=1 : 1, rt, **26** 72 %, **27** 69 %; (c) NH_2_OH ⋅ HCl, NaOMe, MeOH, rt, **28** 52 %, **29** 70 %.

**Scheme 6:**
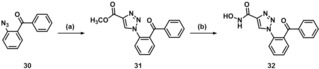
Reagents and conditions: (a) methyl propiolate, sodium ascorbate, CuSO_4_ ⋅ 5 H_2_O, *t*BuOH : H_2_O=1 : 1, rt, 42 %; (b) NH_2_OH ⋅ HCl, NaOMe, MeOH, rt, 29 %.

In contrast, diphenyl ethers **28** and **29** were synthesized from commercially available 2‐phenoxyanilines **22** and **23**, respectively (Scheme 5). From these primary aromatic amines, via a diazotization and the subsequent reaction of the resulting diazonium salts with azide ions, azides **24** and **25** were obtained.^48^ Subsequently, these azides were subjected to a copper(I)‐catalyzed [3+2]‐cycloaddition^49^ with methyl propiolate and a final aminolysis with hydroxylamine yielding diphenyl ethers **28** and **29**. In principally the same way, benzophenone derivative **32** was obtained from azide **30** (Scheme 6).

### HDAC inhibitory activity of the *ortho*‐substituted 1‐phenyl‐1*H*‐1,2,3‐triazole derivatives

Also the synthesized series of *ortho*‐substituted 1‐phenyl‐1*H*‐1,2,3‐triazole derivatives was pretested for inhibitory activity against smHDAC8 at concentrations of 50 μM and 5 μM (Table 2). Among the tested triazole derivatives, hydroxamic acids **16** and **17**, bearing an arylmethoxy substituent in position 2 of their phenyl ring, were the least active compounds. The polar phenols **12** and **13** showed moderate activity and were almost as active as benzophenone **32**. The highest inhibitory activity was found for diphenyl ethers **20**, **21**, **28**, and **29**. Among these compounds, the 4‐fluorophenoxy derivative **21** was shown to be the most potent smHDAC8 inhibitor. With an IC_50_ value of 0.50 μM, its inhibitory activity against smHDAC8 exceeds those of **2 b** and SAHA (**1**), whereas the compound exhibits only low activity against hsHDAC1 and hsHDAC6 (Table 3). Although its inhibitory activity against hsHDAC8 is increased with respect to **2 b**, diphenyl ether **21** shows an even greater selectivity for smHDAC8 than the unsubstituted phenyl derivative **2 b**. Its selectivity over all tested human HDACs makes triazole derivative **21** a promising starting point for the development of smHDAC8‐specific inhibitors.

**Table 2:** *In vitro* inhibition of smHDAC8 (Fluor de Lys) by the *ortho*‐substituted 1‐phenyl‐1*H*‐1,2,3‐triazole derivatives.

compound	% inhibition @ 50 μM	% inhibition @ 5 μM
**12**	63.5	19.5
**13**	67.3	14.5
**16**	43.5	‐1.5
**17**	21.0	‐9.3
**20**	91.1	49.8
**21**	93.4	66.2
**28**	87.9	49.1
**29**	91.5	51.4
**32**	75.4	23.4

**Table 3:** *In vitro* inhibition of smHDAC8, hsHDAC8, hsHDAC6 and hsHDAC1. The employed substrate is given in brackets.

compound	smHDAC8 IC_50_ [μM] (Fluor de Lys)	hsHDAC8 IC_50_ [μM] (Fluor de Lys)	hsHDAC6 IC_50_ [μM]/% inhibition (ZMAL)	hsHDAC1 IC_50_ [μM]/% inhibition (ZMAL)
SAHA (**1**)^5^, ^50^	1.17±0.33	0.64±0.21	0.14±0.12	0.117±0.0056 97.3±1.3 % @ 10 μM
**2 b**	4.44±1.52	12.43±1.08	42.50±3.64	8.1±1.0 % @ 10 μM
**21**	0.504±0.046	2.2±0.68	34.5 % @ 50 μM −1.5 % @ 5 μM	38.2 % @ 50 μM 11 % @ 5 μM

### Comparison of the obtained crystal structures

**Figure 4:**
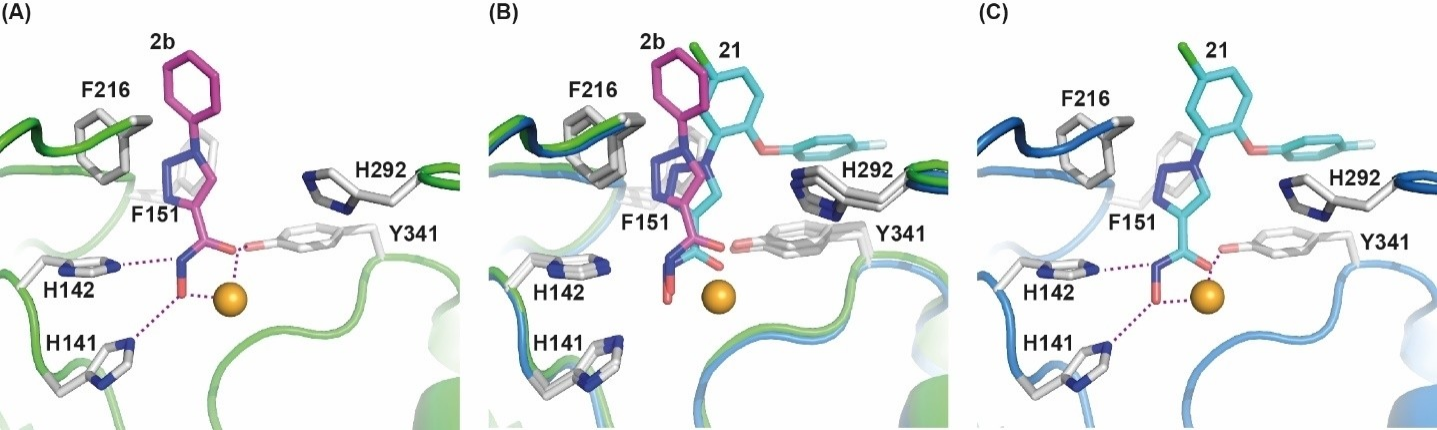
Comparison of inhibitors **2 b** (magenta carbon atoms) and **21** (cyan carbon atoms) binding to smHDAC8. Close‐up views of **2 b** (A) and **21** (C) binding to smHDAC8 active site and their superposition (B). Representation and coloring is as in Figure 1 B. Only inhibitor **21** is observed binding to the HDAC8‐selectivity pocket. This specific binding mode could be responsible for the movement observed for the triazole ring of **21** compared to **2 b**.

Comparison of the smHDAC8/**2 b** and smHDAC8/**21** structures shows that both inhibitors bind in a similar way into the smHDAC8 active site (Figure 4). However, **21** is slightly tilted within the smHDAC8 active site pocket compared to **2 b**, its triazole ring coming closer to histidine H292. We have previously shown that compound **21**, like many HDAC8‐selective inhibitors, was able to bind to a HDAC8‐selective pocket formed by HDAC8 loops L1 and L6 and the side chain of the catalytic tyrosine (smHDAC8 Y341/human HDAC8 Y306).^28^ Specifically, the fluoro‐phenyl capping group of **21** is stacked onto the Y341 side chain, an interaction that appears essential for the binding of many HDAC8‐selective inhibitors to HDAC8. We postulate that the tilting observed for **21** compared to **2 b** is caused by the maximization of the stacking interaction between H292 and the **21** capping group. This would also be in agreement with the lower IC_50_ for **21** compared to **2 b** measured for smHDAC8. Once again, the bulkier human HDAC8 M274 in replacement of smHDAC8 H292, notably considering the rapprochement of the triazole ring toward this residue, could explain the higher selectivity of **21** for smHDAC8. Interestingly, the lower IC_50_ for **21** compared to **2 b** for human HDAC8 could be explained by the stacking interaction between the catalytic tyrosine and **21** capping group, demonstrating once again how this latter interaction is essential for the selective inhibition of HDAC8 enzymes.

### Docking of the novel 1‐phenyl substituted triazole derivatives

**Figure 5:**
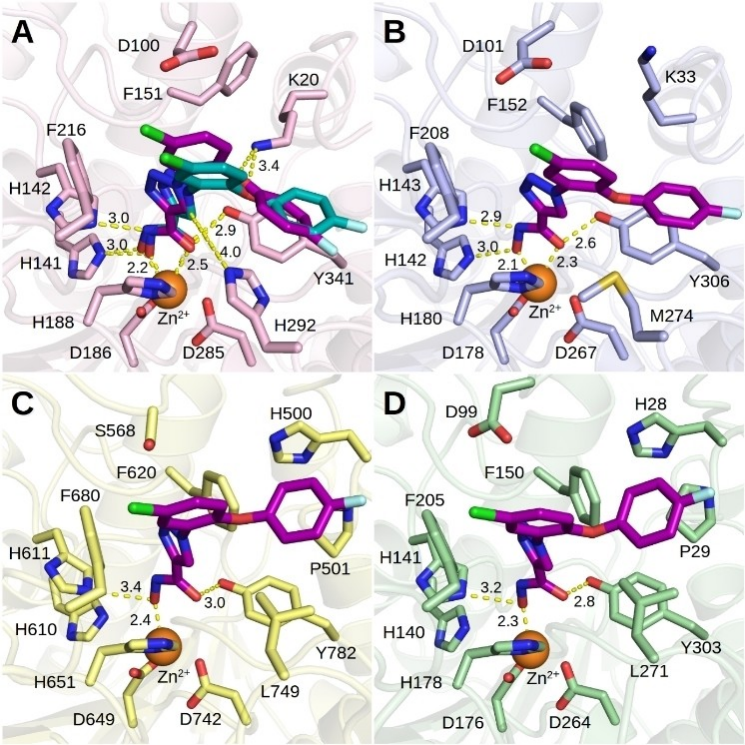
Binding modes of compound **21** (predicted – dark magenta carbon atoms, experimental – dark cyan carbon atoms) in: (A) smHDAC8 (pale pink carbons), (B) hsHDAC8 (pale blue carbons), (C) hsHDAC6 (pale yellow carbons) and (D) hsHDAC1 (pale green carbons). Catalytic zinc ion is shown as orange sphere. Nitrogens are colored dark blue, oxygens – red, sulfur – dark yellow, chlorine – green, fluorine – pale cyan. Metal interactions and hydrogen bonds are shown as dashed yellow lines.

Biological testing of the synthesized **2 b** derivatives **12**, **13**, **16**, **17**, **20**, **21**, **28**, **29**, and **32** showed that indeed all compounds are active on smHDAC8 (Schemes 5–6, Tables 2 and 3). The most active compound **21** not only showed increased activity, but also an improved selectivity profile, which could be explained by analysis of its binding modes (Figure 5). In smHDAC8 and hsHDAC8 (Figure 5A–B) the inhibitor chelates the zinc ion similar to **2 b** (Figure 3). The triazole ring is also placed in a similar way and it interacts with unique H292 in smHDAC8. However, as discussed previously for **2 b**, in the case of hsHDAC8 the hydrogen bond with M274 and the π‐cation interaction with K20 are not observed. The central aromatic chlorophenyl ring is rotated 90 degrees in comparison to the phenyl ring of **2 b** and makes an edge‐to‐face aromatic interaction with F216 and the corresponding F208 in smHDAC8 and hsHDAC8 respectively. The oxygen atom of the linker is able to make a hydrogen bond with K20 in smHDAC8, but not in hsHDAC8, which might contribute to the selectivity of the compound. The distal aromatic fluorophenyl ring is placed comfortably in the side pocket of parasitic and human HDAC8 isoform above the conserved Y341 and Y303 respectively, which probably contributes the most to the increased activity of **21** in comparison to **2 b**. The binding modes of **21** in hsHDAC6 and hsHDAC1 are completely different (Figure 5C–D). The L‐shaped form of the inhibitor prevents it from fitting into the binding pocket of these isoforms due to the absence of the side pocket and it can only reach the zinc ion with one oxygen atom of the hydroxamic acid losing a number of H‐bond interactions.

Finally, the predicted binding modes of other **2 b** derivatives were analyzed to understand why some compounds were more active than the others. Compounds **20**, **21**, **28** and **29** having an aryloxy moiety showed the highest activity and their binding modes were similar to **21**. In contrast, the least active compounds **16** and **17** with an arylmethoxy scaffold showed shifted positions of the distal and central aromatic rings due to a longer linker between them and, as a consequence, lost the hydrogen bond with K20 and did not form an aromatic interaction with Y341. The benzophenone derivative **32** interacted with K20 via its carbonyl group, but showed lower activity than diphenyl ethers probably due to penalty from the distortion of the conjugated planar system required for this interaction. The phenols **12** and **13** showed binding modes similar to **2 b** with an additional interaction between the phenol group and D100. However, they were less active than diphenyl ethers due to the absence of a large aryl group embedded into the side pocket.

### Schistosome viability testing

Selected compounds (Table 4) were tested for their effects on the viability of *S. mansoni* larvae (schistosomula) using a resazurin‐based fluorescence assay that measures mitochondrial activity. Assays were carried out in triplicate on two separate batches of schistosomula and the results are expressed as the percentage of the value obtained with the solvent (DMSO). Initial testing was done at 10 μM as part of a high‐throughput screening procedure and compounds provoking at least a 25 % reduction in viability were tested at 20 μM to determine a dose‐response. Of the triazole‐based smHDAC8 inhibitors tested, only compound **29** provoked a significant reduction in viability at 10 and 20 μM (Table 4). However, the level of reduction obtained was mediocre and this compound was not considered for further testing. We have previously shown^27^ that some potent inhibitors of smHDAC8 were inactive or had feeble effects on the parasite in our viability assay. We consider that this is most likely due to a poor level of transport across the complex barrier represented by the parasite tegument for the inactive compounds, since some hydroxamate‐based inhibitors have extremely potent effects on the parasite in the same assay.^27^ This despite an apparently favorable lipophilicity index. A variety of schistosome viability assays are currently used in the field [e. g. Panic *et al*.,^51^ Singh *et al*.^52^] that do not always concur. However, since we have compared compounds that *a priori* have the same biological target, it is unlikely that they will have different effects on the parasite and that a different viability assay, for example based on morphological characteristics,^52^ would give different results. Nevertheless, in view of the potency of the triazole‐based smHDAC8 inhibitors on the enzyme, their further development, aimed at improving their bioavailability, is justified.

**Table 4:** Effects on the viability of *S. mansoni* larvae (schistosomula) determined in a resazurin‐based fluorescence assay. nd: not determined.

compound	10 μM		20 μM	
	% viability	±SEM	% viability	±SEM
**2 b**	92.54	5.70	nd	nd
**21**	81.95	4.54	nd	nd
**29**	64.21	1.28	49.88	3.18

## Summary and Conclusion

The present study experimentally demonstrates the potential of triazole‐based small‐molecules as potent and selective *S. mansoni* HDAC8 inhibitors, which could find an application in the treatment of schistosomiasis after improving the bioavailability.

As we reported previously, HDAC8 active site residues and loops demonstrate high flexibility, thereby making the development of HDAC8 selective inhibitors a complex task.^28^ In this study, however, structural modifications of weak inhibitors of hsHDAC8 allowed us to identify the lead structure **2 b**, exhibiting promising selectivity for the parasitic smHDAC8 over the human enzymes hsHDAC8, hsHDAC1, and hsHDAC6. Crystallographic studies of the smHDAC8/**2 b** complex as well as docking studies revealed key interactions between the triazole ring of **2 b** and the enzyme's active site, being crucial for the selectivity profile of **2 b**. The triazole moiety of **2 b** was found to form a weak hydrogen bond and an aromatic interaction with the smHDAC8 specific residue H292 as well as a π‐cation interaction with K20 of the enzyme. These two interactions are not observed between **2 b** and the respective amino acids of the human orthologue hsHDAC8, due to the replacement of H292 by M274 and the flipped out conformation of K33. Furthermore, the favorable orientation of the triazole ring in smHDAC8 allows a π‐stacking interaction between the phenyl ring of **2 b** and F216, whereas for hsHDAC8 this interaction with the corresponding F208 is not detected. As evidenced by additionally performed docking studies, in hsHDAC6 and hsHDAC1 the bulkier L749/L271 residues are responsible for a more closed side pocket and, in addition, no H‐bond interactions can be formed between the triazole and the leucine residue. Therefore, the inhibitory activity of **2 b** toward hsHDAC1/6 is reduced.

In an attempt to further improve the affinity of **2 b** toward smHDAC8 and to further enhance selectivity, a series of *ortho*‐substituted 1‐phenyl‐1H‐1,2,3‐triazole‐based hydroxamates was designed and synthetically accessed via Cu(I)‐catalyzed [3+2]‐cycloaddition reactions, Chan‐Evans‐Lam couplings, and aminolyses with hydroxylamine. Among the synthesized compounds, 4‐fluorophenoxy derivative **21** was found to be the most potent smHDAC8 inhibitor showing IC_50_ value of 0.50 μM, thereby exceeding the smHDAC8 inhibitory activity of the initial lead compound **2 b** as well as of SAHA (**1**), while exhibiting an improved selectivity profile over the investigated human HDACs. Comparative analysis of the crystal structures of smHDAC8/**2 b** and smHDAC8/**21** revealed that the triazole ring of **21** resides even closer to the smHDAC8 specific residue H292 and the fluoro‐phenyl capping group of **21** acquires an additional interaction with the catalytic Y341, collectively resulting in a more potent smHDAC8 inhibitory activity of **21** compared to **2 b**.

Although the effects of the compounds on the viability of *S. mansoni* larvae was mediocre to low, our findings give an insight into the specific interactions between smHDAC8 and triazole‐based inhibitors and reveal these small molecules as promising lead structures for the development of potent and selective smHDAC8 inhibitors as so far only limited data is available on inhibitors that are more potent on smHDAC8 over the human orthologue.

## Experimental Section

### Chemistry, general

Unless otherwise mentioned, THF was dried with sodium/benzophenone and was freshly distilled before use. Thin layer chromatography (tlc): Silica gel 60 F_254_ plates (Merck). Flash chromatography (fc): Silica gel 60, 40–64 μm (Macherey‐Nagel); parentheses include: diameter of the column, fraction size, eluent, R_f_ value. Melting point (m.p.): Melting point apparatus SMP 3 (Stuart Scientific), uncorrected. ^1^H NMR (400 MHz), ^13^C NMR (100 MHz): Mercury plus 400 spectrometer (Varian); δ in ppm related to tetramethylsilane; coupling constants are given with 0.5 Hz resolution. IR: IR Prestige‐21(Shimadzu). HRMS: MicrOTOF‐QII (Bruker). HPLC methods for the determination of product purity: Method 1: Merck Hitachi Equipment; UV detector: L‐7400; autosampler: L‐7200; pump: L‐7100; degasser: L‐7614; column: LiChrospher® 60 RP‐select B (5 μm); LiChroCART® 250–4 mm cartridge; flow rate: 1.00 mL/min; injection volume: 5.0 μL; detection at λ=210 nm for 30 min; solvents: A: water with 0.05 % (V/V) trifluoroacetic acid; B: acetonitrile with 0.05 % (V/V) trifluoroacetic acid: gradient elution: (A %): 0–4 min: 90 %, 4–29 min: gradient from 90 % to 0 %, 29–31 min: 0 %, 31–31.5 min: gradient from 0 % to 90 %, 31.5–40 min: 90 %. Method 2: Merck Hitachi Equipment; UV detector: L‐7400; pump: L‐6200 A; column: phenomenex Gemini® 5 μm C6‐Phenyl 110 Å; LC Column 250×4.6 mm; flow rate: 1.00 mL/min; injection volume: 5.0 μL; detection at λ=254 nm for 20 min; solvents: A: acetonitrile : 10 mM ammonium formate=10 : 90 with 0.1 % formic acid; B: acetonitrile : 10 mM ammonium formate=90 : 10 with 0.1 % formic acid; gradient elution: (A %): 0–5 min: 100 %, 5–15 min: gradient from 100 % to 0 %, 15–20 min: 0 %, 20–22 min: gradient from 0 % to 100 %, 22–30 min: 100 %.

Synthetic procedures and analytical data of compounds **5 c–g,i**, **2 c–g,i**, **10**, **12**, **14**, **16**, **18**, **20**, **24**, **26**, **28** are given in the Supporting Information.

**Methyl 1‐(4‐iodophenyl)‐1*H*‐1,2,3‐triazole‐4‐carboxylate (5 a)**: Methyl propiolate (0.36 mL, 4.0 mmol) and 1‐azido‐4‐iodobenzene (1.47 g, 6.0 mmol) were dissolved in a 1 : 1 mixture of *t*BuOH and H_2_O (10 mL). Sodium ascorbate (79 mg, 0.4 mmol) and copper(II) sulfate pentahydrate (20 mg, 0.08 mmol) were added and the mixture was stirred overnight. Then water was added and the mixture was extracted ethyl acetate (3×). The combined organic phases were dried (Na_2_SO_4_), filtered, and evaporated. The residue was purified by flash column chromatography (Ø=2 cm, h=15 cm, V=10 mL, cyclohexane/ethyl acetate=2 : 1, R_f_=0.31) to give **5 a** as colorless solid (1.29 g, 3.9 mmol, 98 % yield). m.p.=189 °C; ^1^H NMR (CDCl_3_): δ [ppm]=4.00 (s, 3H, CO_2_C*H*_3_), 7.51–7.54 (m, 2H, 2′‐H_4‐iodophenyl_, 6′‐H_4‐iodophenyl_), 7.88–7.91 (m, 2H, 3′‐H_4‐iodophenyl_, 5′‐H_4‐iodophenyl_), 8.51 (s, 1H, 5‐H_triazole_); ^13^C NMR (CDCl_3_): δ [ppm]=52.6 (1C, CO_2_ *C*H_3_), 94.8 (1C, C‐4′_4‐iodophenyl_), 122.4 (2C, C‐2′_4‐iodophenyl_, C‐6′_4‐iodophenyl_), 125.5 (1C, C‐5_triazole_), 136.0 (1C, C‐1′_4‐iodophenyl_), 138.3 (1C, C‐4_triazole_), 139.2 (2C, C‐3′_4‐iodophenyl_, C‐5′_4‐iodophenyl_), 161.0 (1C, *C*O_2_CH_3_); IR (neat): $\tilde{\nu }$ [cm^−1^]=1728, 1551, 1493, 1435, 1400, 1342, 1250, 1196, 1150, 1038, 984, 768; HRMS (m/z): [M+H]^+^ calcd for C_10_H_9_IN_3_O_2_, 329.9734; found, 329.9704; HPLC (method 1): t_R_=18.3 min, purity 95.8 %.

**Methyl 1‐phenyl‐1*H*‐1,2,3‐triazole‐4‐carboxylate (5 b)**: Methyl propiolate (0.18 mL, 2.0 mmol) and azidobenzene (357 mg, 3.0 mmol) were dissolved in a 1 : 1 mixture of *t*BuOH and H_2_O (10 mL). Sodium ascorbate (40 mg, 0.2 mmol) and copper(II) sulfate pentahydrate (10 mg, 0.04 mmol) were added and the mixture was stirred overnight. Then water was added and the mixture was extracted ethyl acetate (3×). The combined organic phases were dried (Na_2_SO_4_), filtered, and evaporated. The residue was purified by flash column chromatography (Ø=2 cm, h=15 cm, V=10 mL, cyclohexane/ethyl acetate=2 : 1, R_f_=0.30) to give **5 b** as colorless solid (345 mg, 1.7 mmol, 85 % yield). m.p.=123 °C; ^1^H NMR (CDCl_3_): δ [ppm]=3.99 (s, 3H, CO_2_C*H*_3_), 7.47 – 7.52 (m, 1H, 4′‐H_phenyl_) 7.53 – 7.58 (m, 2H, 3′‐H_phenyl_, 5′‐H_phenyl_), 7.73–7.77 (m, 2H, 2′‐H_phenyl_, 6′‐H_phenyl_), 8.52 (s, 1H, 5‐H_triazole_); ^13^C NMR (CDCl_3_): δ [ppm]=52.5 (1C, CO_2_ *C*H_3_), 120.9 (2C, C‐2′_phenyl_, C‐6′_phenyl_), 125.7 (1C, C‐5_triazole_), 129.7 (1C, C‐4′_phenyl_), 130.1 (2C, C‐3′_phenyl_, C‐5′_phenyl_), 136.4 (1C, C‐1′_phenyl_), 140.7 (1C, C‐4_triazole_), 161.2 (1C, *C*O_2_CH_3_); IR (neat): $\tilde{\nu }$ [cm^−1^]=3152, 1728, 1593, 1528, 1497, 1369, 1227, 1150, 1038, 768, 694; HRMS (m/z): [M+H]^+^ calcd for C_10_H_10_N_3_O_2_, 204.0768; found, 204.0764; HPLC (method 1): t_R_=15.2 min, purity 95.1 %.

**Methyl 1‐[4‐(phenylethynyl)phenyl)]‐1*H*‐1,2,3‐triazole‐4‐carboxylate (5 h)**: Under N_2_ atmosphere tetrakis(triphenylphosphine)palladium(0) (12 mg, 0.01 mmol) and copper(I) iodide (4 mg, 0.02 mmol) were added to a solution of **5 a** (329 mg, 1.0 mmol) in a 1 : 2 mixture of triethylamine and DMF (15 mL). Then phenylacetylene (0.13 mL, 1.2 mmol) was added slowly and the mixture was stirred overnight at 70 °C. Then the reaction mixture was diluted with water and extracted with ethyl acetate (3×). The combined organic phases were dried with Na_2_SO_4_, filtered, and the solvent was removed in vacuo. The residue was purified by flash column chromatography (Ø=2 cm, h=15 cm, V=10 mL, cyclohexane/ethyl acetate=3 : 1, R_f_ = 0.28) to give **5 h** as colorless crystalline solid (291 mg, 0.96 mmol, 96 % yield). m.p.=189 °C; ^1^H NMR (CDCl_3_): δ [ppm]=4.00 (s, 3H, CO_2_C*H*_3_), 7.35–7.40 (m, 3H, H_arom._), 7.53–7.57 (m, 2H, H_arom._), 7.68–7.73 (m, 2H, H_arom._), 7.75–7.79 (m, 2H, H_arom._), 8.54 (s, 1H, 5‐H_triazole_); ^13^C NMR (CDCl_3_): δ [ppm]=52.6 (1C, CO_2_ *C*H_3_), 87.9 (1C, *C*≡C), 91.9 (1C, *C*≡C), 120.7 (2C, C_arom._), 122.7 (1C, C_arom._), 125.0 (1C, C_arom._), 125.5 (1C, C‐5_triazole_), 128.6 (2C, C_arom._), 129.0 (1C, C_arom._), 131.8 (2C, C_arom._), 133.2 (2C, C_arom._), 135.7 (1C, C_arom._), 140.8 (1C, C‐4_triazole_), 161.1 (1C, *C*O_2_CH_3_); IR (neat): $\tilde{\nu }$ [cm^−1^]=3132, 2951, 1701, 1543, 1520, 1435, 1350, 1265, 1157, 1034, 760, 694; HRMS (m/z): [M+H]^+^ calcd for C_18_H_14_N_3_O_2_, 304.1081; found, 304.1066; HPLC (method 1): t_R_=21.2 min, purity 98.1 %.

**Methyl 1‐{4‐[2‐(trimethylsilyl)ethynyl]phenyl}‐1*H*‐1,2,3‐triazole‐4‐carboxylate (7)**: Under N_2_ atmosphere tetrakis(triphenylphosphine)palladium(0) (12 mg, 0.01 mmol), copper(I) iodide (4 mg, 0.02 mmol) and triethylamine (0.70 mL, 5.0 mmol) were added to a solution of **5 a** (329 mg, 1.0 mmol) in dry acetonitrile (15 mL). Then trimethylsilylacetylene (0.28 mL, 2.0 mmol) was added slowly. After stirring the reaction mixture at ambient temperature for 3 h, water was added and the mixture was extracted with ethyl acetate (3×). The combined organic phases were dried with Na_2_SO_4_, filtered, and the solvent removed in vacuo. The residue was purified by flash column chromatography (Ø=2 cm, h=15 cm, V=10 mL, cyclohexane/ethyl acetate=4 : 1, R_f_=0.29) to give **7** as colorless crystalline solid (290 mg, 0.97 mmol, 97 % yield). m.p.=188 °C; ^1^H NMR (CDCl_3_): δ [ppm]=0.27 (s, 9H, Si(C*H_3_*)_3_), 4.00 (s, 3H, CO_2_C*H*_3_), 7.61 – 7.65 (m, 2H, 3′‐H_4‐[2‐(trimethylsilyl)ethynyl]phenyl_, 5′‐H_4‐[2‐(trimethylsilyl)ethynyl]phenyl_), 7.70–7.75 (m, 2H, 2′‐H_4‐[2‐(trimethylsilyl)ethynyl]phenyl_, 6′‐H_4‐[2‐(trimethylsilyl)ethynyl]phenyl_), 8.58 (s br, 1H, 5‐H_triazole_); ^13^C NMR (CDCl_3_): δ [ppm]=0.0 (3C, Si(*C*H_3_)_3_), 52.6 (1C, CO_2_ *C*H_3_), 97.3 (1C, C≡*C*Si(C*H_3_*)_3_), 103.3 (1C, *C*≡CSi(C*H_3_*)_3_), 120.6 (2C, C‐2′_4‐[2‐(trimethylsilyl)ethynyl]phenyl_, C‐6′_4‐[2‐(trimethylsilyl)ethynyl]phenyl_), 124.8 (1C, C‐1′_4‐[2‐(trimethylsilyl)ethynyl]phenyl_), 133.7 (2C, C‐3′_4‐[2‐(trimethylsilyl)ethynyl]phenyl_, C‐5′_4‐[2‐(trimethylsilyl)ethynyl]phenyl_), 136.0 (1C, C‐4′_4‐[2‐(trimethylsilyl)ethynyl]phenyl_), 161.1 (1C, *C*O_2_CH_3_), the signals for the carbon atoms of the triazole ring could not be observed; IR (neat): $\tilde{\nu }$ [cm^−1^]=3140, 2959, 1717, 1555, 1516, 1265, 1254, 1157, 1042, 841, 760, 679; HRMS (m/z): [M+H]^+^ calcd for C_15_H_18_N_3_O_2_Si, 300.1163; found, 300.1173; HPLC (method 1): t_R_=21.3 min, purity 98.7 %.

**Methyl 1‐[4‐(4‐phenylbuta‐1,3‐diynyl)phenyl]‐1*H*‐1,2,3‐triazole‐4‐carboxylate (5 j)**: Tetrabutylammonium fluoride trihydrate (315 mg, 1.0 mmol) was added to a solution of **7** (250 mg, 0.84 mmol) in dichlromethane (15 mL) and the reaction mixture was stirred at ambient temperature for 30 min. Then the solution was diluted with water and extracted with dichloromethane (3×). The combined organic phases were dried with Na_2_SO_4_, filtered, and the solvent was removed in vacuo. The crude product was dissolved in mixture of pyridine and MeOH (1 : 1, 4 mL). Then phenylacetylene (0.46 mL, 4.2 mmol), copper(II) acetate (309 mg, 1.7 mmol) were added and the mixture was stirred overnight at ambient temperature. Then the reaction mixture was diluted with water and extracted with ethyl acetate (3×). The combined organic phases were dried with Na_2_SO_4_, filtered, and the solvent was removed in vacuo. The residue was purified by flash column chromatography (Ø=2 cm, h=15 cm, V=10 mL, cyclohexane/ethyl acetate=3 : 1, R_f_=0.28) to give **5 j** as colorless crystalline solid (169 mg, 0.52 mmol, 62 % yield). m.p.=198 °C; ^1^H NMR (CDCl_3_): δ [ppm]=4.00 (s, 3H, CO_2_C*H*_3_), 7.33–7.42 (m, 3H, 3′′‐H_phenyl_, 4′′‐H_phenyl_, 5′′‐H_phenyl_), 7.53–7.56 (m, 2H, 2′′‐H_phenyl_, 6′′‐H_phenyl_), 7.68–7.72 (m, 2H, 3′‐H_4‐(4‐phenylbuta‐1,3‐diynyl)phenyl_, 5′‐H_4‐(4‐phenylbuta‐1,3‐diynyl)phenyl_), 7.75–7.79 (m, 2H, 2′‐H_4‐(4‐phenylbuta‐1,3‐diynyl)phenyl_, 6′‐H_4‐(4‐phenylbuta‐1,3‐diynyl)phenyl_), 8.53 (s, 1H, 5‐H_triazole_); ^13^C NMR (CDCl_3_): δ [ppm]=52.6 (1C, CO_2_ *C*H_3_), 73.6 (1C, *C*≡C), 76.4 (1C, *C*≡C), 79.7 (1C, *C*≡C), 83.1 (1C, *C*≡C), 120.7 (2C, C‐2′_4‐(4‐phenylbuta‐1,3‐diynyl)phenyl_, C‐6′_4‐(4‐phenylbuta‐1,3‐diynyl)phenyl_), 121.5 (1C, C‐1′′_phenyl_), 123.6 (1C, C‐1′_4‐(4‐phenylbuta‐1,3‐diynyl)phenyl_), 125.4 (1C, C‐5_triazole_), 128.7 (2C, C‐3′′_phenyl,_ C‐5′′_phenyl_), 129.7 (1C, C‐4′′_phenyl_), 132.7 (2C, C‐2′′_phenyl_, C‐6′′_phenyl_), 134.2 (2C, C‐3′_4‐(4‐phenylbuta‐1,3‐diynyl)phenyl_, C‐5′_4‐(4‐phenylbuta‐1,3‐diynyl)phenyl_), 136.3 (1C, C‐4′_4‐(4‐phenylbuta‐1,3‐diynyl)phenyl_), 140.9 (1C, C‐4_triazole_), 161.0 (1C, *C*O_2_CH_3_); IR (neat): $\tilde{\nu }$ [cm^−1^]=2986, 1728, 1520, 1439, 1369, 1231, 1153, 1042, 833, 756, 691; HRMS (m/z): [M+H]^+^ calcd for C_20_H_14_N_3_O_2_, 328.1081; found, 328.1095; HPLC (method 1): t_R_=21.8 min, purity 98.0 %.

***N*** **‐Hydroxy‐1‐phenyl‐1*H*‐1,2,3‐triazole‐4‐carboxamide (2 b)**: Hydroxylamine hydrochloride (139 mg, 2.0 mmol) and a 2.0 M solution of sodium methoxide in methanol (1.5 mL, 3.0 mmol) were added to a solution of **5 b** (203 mg, 1.0 mmol) in dry methanol (8 mL). The reaction mixture was stirred at ambient temperature for 20 h until TLC showed complete conversion of the ester. The reaction mixture was acidified with 1.0 M HCl to pH 5–6. Then the mixture was extracted with ethyl acetate (3×). The combined organic phases were dried with Na_2_SO_4_, filtered, and the solvent was dried in vacuo. The crude mixture was purified by flash column chromatography (Ø=2 cm, h=15 cm, V=10 mL, dichloromethane/methanol=10 : 1, R_f_=0.37) to give **2 b** as colorless crystalline solid (135 mg, 0.66 mmol, 66 %). m.p.=171 °C; ^1^H NMR (CD_3_OD): δ [ppm]=7.50–7.55 (m, 1H, H‐4′_phenyl_), 7.58–7.63 (m, 2H, 3′‐H_phenyl_, 5′‐H_phenyl_), 7.86–7.90 (m, 2H, 2′‐H_phenyl_, 6′‐H_phenyl_), 8.91 (s, 1H, 5‐H_triazole_); ^13^C NMR (CD_3_OD): δ [ppm]=121.8 (2C, C‐2′_phenyl_, C‐6′_phenyl_), 125.3 (1C, C‐5_triazole_), 130.5 (1C, C‐4′_phenyl_), 131.0 (2C, C‐3′_phenyl_, C‐5′_phenyl_), 138.0 (1C, C‐1′_phenyl_), 143.0 (1C, C‐4_triazole_), 160.3 (1C, *C*ONHOH); IR (neat): $\tilde{\nu }$ [cm^−1^]=3325, 3136, 1628, 1566, 1493, 1412, 1254, 1177, 1030, 876, 760, 683; HRMS (m/z): [M+H]^+^ calcd for C_9_H_9_N_4_O_2_, 205.0720; found, 205.0712; HPLC (method 2): t_R_=12.5 min, purity 99.3 %.

***N*** **‐Hydroxy‐1‐[4‐(2‐phenylethynyl)phenyl]‐1*H*‐1,2,3‐triazole‐4‐carboxamide (2 h)**: Hydroxylamine hydrochloride (195 mg, 2.8 mmol) and a 2.0 M solution of sodium methoxide in methanol (1.4 mL, 2.8 mmol) were added to a solution of **5 h** (170 mg, 0.56 mmol) in dry methanol (10 mL). The reaction mixture was stirred at ambient temperature for 20 h until TLC showed complete conversion of the ester. Then reaction mixture was acidified with a 1.0 M solution of HCl until pH 5–6 was reached. The precipitate was washed with dichloromethane, water, and ethyl acetate and dried in vacuo for 4 h to give **2 h** as colorless solid (156 mg, 0.51 mmol, 91 % yield). TLC (CH_2_Cl_2_:methanol=10 : 1): R_f_=0.35; m.p.=219 °C; ^1^H NMR (DMSO‐d_6_): δ [ppm]=7.44–7.48 (m, 3H, 3′′‐H_phenyl_, 4′′‐H_phenyl_, 5′′‐H_phenyl_), 7.58–7.62 (m, 2H, 2′′‐H_phenyl_, 6′′‐H_phenyl_), 7.77–7.81 (m, 2H, 3′‐H_4‐(2‐phenylethynyl)phenyl_, 5′‐H_4‐(2‐phenylethynyl)phenyl_), 8.00–8.06 (m, 2H, 2′‐H_4‐(2‐phenylethynyl)phenyl_, 6′‐H_4‐(2‐phenylethynyl)phenyl_), 9.18 (s br, 1H, N*H*O*H*), 9.33 (s, 1H, 5‐H_triazole_), 11.45 (s br, 1H, N*H*O*H*); ^13^C NMR (DMSO‐d_6_): δ [ppm]=88.2 (1C, *C*≡C), 90.9 (1C, *C*≡C), 120.5 (2C, C‐2′_4‐(2‐phenylethynyl)phenyl_, C‐6′_4‐(2‐phenylethynyl)phenyl_), 121.9 (1C, C‐1′′_phenyl_), 122.9 (1C, C‐4′_4‐(2‐phenylethynyl)phenyl_), 124.5 (1C, C‐5_triazole_), 128.8 (2C, C‐3′′_phenyl_, C‐5′′_phenyl_), 129.1 (1C, C‐4′′_phenyl_), 131.5 (2C, C‐2′′_phenyl_, C‐6′′_phenyl_), 132.9 (2C, C‐3′_4‐(2‐phenylethynyl)phenyl_, C‐5′_4‐(2‐phenylethynyl)phenyl_), 135.9 (1C, C‐1′_4‐(2‐phenylethynyl)phenyl_), 142.4 (1C, C‐4_triazole_), 157.1 (1C, *C*ONHOH); IR (neat): $\tilde{\nu }$ [cm^−1^]=3310, 3140, 2986, 1632, 1566, 1516, 1497, 1439, 1373, 1258, 1177, 1030, 880, 841, 752, 691; HRMS (m/z): [M+H]^+^ calcd for C_17_H_13_N_4_O_2_, 305.1033; found, 305.1035; HPLC (method 2): t_R_=16.6 min, purity 96.1 %.

***N*** **‐Hydroxy‐1‐[4‐(4‐phenylbuta‐1,3‐diynyl)phenyl]‐1*H*‐1,2,3‐triazole‐4‐carboxamide (2 j)**: Hydroxylamine hydrochloride (192 mg, 2.8 mmol) and a 2.0 M solution of sodium methoxide in methanol (1.4 mL, 2.8 mmol) were added to a solution of **5 j** (150 mg, 0.46 mmol) in dry methanol (10 mL). The reaction mixture was stirred at ambient temperature for 20 h until TLC showed complete conversion of the ester. The reaction mixture was acidified with a 1.0 M solution of HCl until pH 5–6 was reached. The precipitate was washed with dichloromethane, water, and ethyl acetate and dried in vacuo for 4 h to give **2 j** as colorless solid (102 mg, 0.31 mmol, 68 % yield). TLC (CH_2_Cl_2_:methanol=9 : 1): R_f_=0.30; m.p.=188 °C (decomposition); ^1^H NMR (DMSO‐d_6_): δ [ppm]=7.43–7.54 (m, 3H, 3′′‐H_phenyl_, 4′′‐H_phenyl_, 5′′‐H_phenyl_), 7.61–7.65 (m, 2H, 2′′‐H_phenyl_, 6′′‐H_phenyl_), 7.83–7.87 (m, 2H, 3′‐H_4‐(4‐phenylbuta‐1,3‐diynyl)phenyl_, 5′‐H_4‐(4‐phenylbuta‐1,3‐diynyl)phenyl_), 8.04–8.08 (m, 2H, 2′‐H_4‐(4‐phenylbuta‐1,3‐diynyl)phenyl_, 6′‐H_4‐(4‐phenylbuta‐1,3‐diynyl)phenyl_), 9.21 (s br, 1H, N*H*O*H*), 9.35 (s, 1H, 5‐H_triazole_), 11.46 (s br, 1H, N*H*O*H*); ^13^C NMR (DMSO‐d_6_): δ [ppm]=73.3 (1C, *C*≡C), 74.9 (1C, *C*≡C), 80.6 (1C, *C*≡C), 82.7 (1C, *C*≡C), 120.2 (1C, C‐1′′_phenyl_), 120.6 (2C, C‐2′_4‐(4‐phenylbuta‐1,3‐diynyl)phenyl_, C‐6′_4‐(4‐phenylbuta‐1,3‐diynyl)phenyl_), 121.0 (1C, C‐1′_4‐(4‐phenylbuta‐1,3‐diynyl)phenyl_), 124.6 (1C, C‐5_triazole_), 129.0 (2C, C‐3′′_phenyl,_ C‐5′′_phenyl_), 130.2 (1C, C‐4′′_phenyl_), 132.5 (2C, C‐2′′_phenyl_, C‐6′′_phenyl_), 134.1 (2C, C‐3′_4‐(4‐phenylbuta‐1,3‐diynyl)phenyl_, C‐5′_4‐(4‐phenylbuta‐1,3‐diynyl)phenyl_), 136.7 (1C, C‐4′_4‐(4‐phenylbuta‐1,3‐diynyl)phenyl_), 142.5 (1C, C‐4_triazole_), 157.1 (1C, *C*ONHOH); IR (neat): $\tilde{\nu }$ [cm^−1^]=3333, 3129, 2920, 2851, 1655, 1570, 1485, 1377, 1250, 1184, 1038, 833, 748, 683; HRMS (m/z): [M+Na]^+^ calcd for C_19_H_12_N_4_O_2_Na, 351.0852; found, 351.0867; HPLC (method 2): t_R_=18.1 min, purity 93.3 %.

**Methyl 1‐(5‐chloro‐2‐hydroxyphenyl)‐1*H*‐1,2,3‐triazole‐4‐carboxylate (11)**: Methyl propiolate (0.36 mL, 336 mg, 4.0 mmol) was added to a stirring solution of **9** (507 mg, 3.0 mmol) in a 1 : 1 mixture of water and *tert*‐butyl alcohol (15 mL). Then sodium ascorbate (40 mg, 0.20 mmol) and copper(II) sulfate pentahydrate (10 mg, 0.04 mmol) were added and the mixture was stirred for 18 h at room temperature. Then water was added and the mixture was extracted with ethyl acetate (3×). The combined organic layers were dried (Na_2_SO_4_), filtered, and the solvent was removed in vacuo. The residue was purified by flash column chromatography (Ø=3 cm, h=15 cm, cyclohexane/ethyl acetate=2/1, V=15 mL, R_f_=0.41) to give **11** (675 mg, 2.7 mmol, 89 %) as pale brown solid. m.p.=130 °C; ^1^H NMR (DMSO‐d_6_): δ [ppm]=3.88 (s, 3H, CO_2_C*H*_3_), 7.12–7.16 (m, 1H, 3‐H′_phenyl_), 7.43–7.47 (m, 1H, 4‐H′_phenyl_), 7.72–7.74 (m, 1H, 6‐H′_phenyl_), 9.07 (s, 1H, 5‐H_triazole_), 11.03 (s br, 1H, O*H*); ^13^C NMR (DMSO‐d_6_): δ [ppm]=52.0 (1C, CO_2_ *C*H_3_), 118.6 (1C, C‐3′_phenyl_), 122.6 (1C, C‐5′_phenyl_), 124.5 (1C, C‐1′_phenyl_), 125.1 (1C, C‐6′_phenyl_), 130.5 (1C, C‐5_triazole_), 130.6 (1C, C‐4′_phenyl_), 138.5 (1C, C‐4_triazole_), 149.2 (1C, C‐2′_phenyl_), 160.6 (1C, *C*O_2_CH_3_); IR (neat): $\tilde{\nu }$ [cm^−1^]=3183, 3144, 1748, 1532, 1508, 1439, 1292, 1157, 1157, 1130, 1049, 818, 733, 698, 656; HRMS (*m*/*z*): [M+H]^+^ calcd for C_10_H_9_ClN_3_O_3_, 254.0327; found, 254.0329; HPLC (method 1): t_R_=17.7 min, purity 99.6 %.

**1‐(5‐Chloro‐2‐hydroxyphenyl)‐*N*‐hydroxy‐1*H*‐1,2,3‐triazole‐4‐carboxamide (13)**: Hydroxylamine hydrochloride (165 mg, 2.4 mmol) and a 2 M solution of sodium methoxide in methanol (1.5 mL, 3.0 mmol) were added to a solution of **11** (101 mg, 0.40 mmol) in dry methanol (10 mL) and the mixture was stirred at ambient temperature for 20 h. Then water was added. The mixture was acidified with 1 M HCl to pH 5–6 and extracted with ethyl acetate (3×). The combined organic layers were dried (Na_2_SO_4_), filtered, and the solvent was removed in vacuo. The residue was purified by automatic flash column chromatography (100 % H_2_O→100 % CH_3_CN, Biotage SNAP KP‐C18‐HS 12 g) to give **13** as colorless solid (43 mg, 0.17 mmol, 43 % yield). m.p.=220 °C; TLC (dichloromethane/methanol, 10/1 V/V): R_f_=0.36; ^1^H NMR (DMSO‐d_6_): δ [ppm]=7.12–7.16 (m, 1H, 3′‐H_phenyl_), 7.41–7.46 (m, 1H, 4′‐H_phenyl_), 7.71–7.74 (m, 1H, 6′‐H_phenyl_), 8.86 (s, 1H, 5‐H_triazole_), 9.16 (s br, 1H, CON*H*O*H*), 11.05 (s br, 1H, O*H*), 11.40 (s br, 1H, CON*H*O*H*); ^13^C NMR (DMSO‐d_6_): δ [ppm]=118.6 (1C, C‐3′_phenyl_), 122.6 (1C, C‐5′_phenyl_), 124.8 (1C, C‐1′_phenyl_), 124.9 (1C, C‐6′_phenyl_), 127.5 (1C, C‐5_triazole_), 130.4 (1C, C‐4′_phenyl_), 141.1 (1C, C‐4_triazole_), 149.1 (1C, C‐2′_phenyl_), 157.4 (1C, *C*ONHOH); IR (neat): $\tilde{\nu }$ [cm^−1^]=3267, 3156, 1670, 1601, 1558, 1408, 1296, 1254, 1200, 1042, 883, 814, 737, 660; HRMS (*m*/*z*): [M+H]^+^ calcd for C_9_H_8_ClN_4_O_3_, 255.0279; found, 255.0305; HPLC (method 2): t_R_=14.0 min, purity 97.2 %.

**Methyl 1‐{5‐chloro‐2‐[(4‐iodobenzyl)oxy]phenyl}‐1*H*‐1,2,3‐triazole‐4‐carboxylate (15)**: 4‐Iodobenzyl bromide (490 mg, 1.7 mmol) was added to a stirring suspension of **11** (350 mg, 1.4 mmol) and cesium carbonate (900 mg, 2.8 mmol) in *N*,*N*‐dimethylformamide (6.5 mL). The reaction mixture was heated to 90 °C for 100 min. After cooling to room temperature, water was added and the mixture was extracted with ethyl acetate (3×). The combined organic layers were dried (Na_2_SO_4_), filtered, and the solvent was removed in vacuo. The residue was purified by flash column chromatography (Ø=3 cm, h=15 cm, cyclohexane/ethyl acetate=9/1→0/1, V=15 mL) to give **15** (380 mg, 0.82 mmol, 59 %) as colorless solid. m.p.=186 °C; TLC (cyclohexane/ethyl acetate, 2/1 V/V): R_f_=0.58; ^1^H NMR (DMSO‐d_6_): δ [ppm]=3.88 (s, 3H, CO_2_C*H*_3_), 5.21 (s, 2H, OC*H_2_*Ar), 7.14–7.17 (m, 2H, 2′′‐H_4‐iodophenyl_, 6′′‐H_4‐iodophenyl_), 7.39–7.42 (m, 1H, 3′‐H_5‐chlorophenyl_), 7.62–7.66 (m, 1H, 4′‐H_5‐chlorophenyl_), 7.70–7.74 (m, 2H, 3′′‐H_4‐iodophenyl_, 5′′‐H_4‐iodophenyl_), 7.82–7.84 (m, 1H, 6′‐H_5‐chlorophenyl_), 9.14 (s, 1H, 5‐H_triazole_); ^13^C NMR (DMSO‐d_6_): δ [ppm]=52.0 (1C, CO_2_ *C*H_3_), 70.0 (1C, O*C*H_2_Ar), 94.3 (1C, C‐4′′_4‐iodophenyl_), 116.1 (1C, C‐3′_5‐chlorophenyl_), 124.7 (1C, C_arom._), 126.0 (1C, C‐6′_5‐chlorophenyl_), 126.2 (1C, C_arom._), 129.6 (2C, C‐2′′_4‐iodophenyl_, C‐6′′_4‐iodophenyl_), 131.0 (1C, C‐4′_5‐chlorophenyl_), 131.1 (1C, C‐5_triazole_), 135.7 (1C, C‐1′′_4‐iodophenyl_), 137.2 (2C, C‐3′′_4‐iodophenyl_, C‐5′′_4‐iodophenyl_), 138.5 (1C, C‐4_triazole_), 149.9 (1C, C‐2′_5‐chlorophenyl_), 160.5 (1C, *C*O_2_CH_3_); IR (neat): $\tilde{\nu }$ [cm^−1^]=3657, 3063, 2978, 1744, 1501, 1462, 1369, 1285, 1246, 1211, 1134, 1038, 999, 949, 826, 795, 772; HRMS (*m*/*z*): [M+H]^+^ calcd for C_17_H_14_ClIN_3_O_3_, 469.9763; found, 469.9786; HPLC (method 1): t_R_=23.3 min, purity 96.7 %.

**1‐{5‐Chloro‐2‐[(4‐iodobenzyl)oxy]phenyl}‐*N*‐hydroxy‐1*H*‐1,2,3‐triazole‐4‐carboxamide (17)**: A 5.4 M solution of sodium methoxide in methanol (0.3 mL, 1.6 mmol) was added to a solution of **15** (110 mg, 0.23 mmol) and hydroxylamine hydrochloride (94 mg, 1.4 mmol) in dry methanol (5 mL). The mixture was stirred at ambient temperature overnight. Then the solvent was removed in vacuo and the residue was purified by automatic flash column chromatography using a Biotage purification apparatus (5 %→100 % ACN in H_2_O, Biotage® SNAP KP‐C18‐HS 30 g). Fractions containing the desired product were combined, dried from acetonitrile under reduced pressure, and subjected to lyophilization to give **17** (55 mg, 0.12 mmol, 52 %) as colorless solid. m.p.=168–170 °C; ^1^H NMR (DMSO‐d_6_): δ [ppm]=5.22 (s, 2H, OC*H_2_*Ar), 7.15–7.19 (m, 2H, 2′′‐H_4‐iodophenyl_, 6′′‐H_4‐iodophenyl_), 7.37–7.40 (m, 1H, 3′‐H_5‐chlorophenyl_), 7.59–7.62 (m, 1H, 4′‐H_5‐chlorophenyl_), 7.69–7.73 (m, 2H, 3′′‐H_4‐iodophenyl_, 5′′‐H_4‐iodophenyl_), 7.79–7.81 (m, 1H, 6′‐H_5‐chlorophenyl_), 8.82 (s, 1H, 5‐H_triazole_); ^13^C NMR (DMSO‐d_6_): δ [ppm]=69.8 (1C, O*C*H_2_Ar), 94.3 (1C, C‐4′′_4‐iodophenyl_), 116.1 (1C, C‐3′_5‐chlorophenyl_), 124.7 (1C, C‐5′_5‐chlorophenyl_), 125.8 (1C, C‐6′_5‐chlorophenyl_), 126.5 (1C, C‐1′_5‐chlorophenyl_), 127.5 (1C, C‐5_triazole_), 129.6 (2C, C‐2′′_4‐iodophenyl_, C‐6′′_4‐iodophenyl_), 130.6 (1C, C‐4′_5‐chlorophenyl_), 135.8 (1C, C‐1′′_4‐iodophenyl_), 137.2 (2C, C‐3′′_4‐iodophenyl_, C‐5′′_4‐iodophenyl_), 141.9 (1C, C‐4_triazole_), 149.7 (1C, C‐2′_5‐chlorophenyl_), 157.3 (1C, *C*ONHOH); IR (neat): $\tilde{\nu }$ [cm^−1^]=3159, 2978, 2886, 1647, 1620, 1578, 1504, 1462, 1404, 1373, 1285, 1250, 1177, 1130, 1096, 1007, 880, 799, 745, 660; HRMS (*m*/*z*): [M+H]^+^ calcd for C_16_H_13_ClIN_4_O_3_, 470.9715; found, 470.9706; HPLC (method 2): t_R_=16.6 min, purity 96.8 %.

**Methyl 1‐[5‐chloro‐2‐(4‐fluorophenoxy)phenyl]‐1*H*‐1,2,3‐triazole‐4‐carboxylate (19)**: A 25 mL round‐bottom flask was charged with **11** (150 mg, 0.59 mmol), Cu(OAc)_2_ (107 mg, 0.59 mmol), 4‐fluorophenylboronic acid (99 mg, 0.71 mmol), and powdered 4 Å molecular sieves. Then dichloromethane (4.5 mL) was added. After the addition of triethylamine (0.41 mL, 2.9 mmol), the reaction mixture was stirred at ambient temperature overnight. Then the suspension was filtered. The filtrate diluted with water and extracted with ethyl acetate (3×). The combined organic layers were dried (Na_2_SO_4_), filtered, and the solvent was removed in vacuo. The residue was purified by flash column chromatography (Ø=2 cm, h=15 cm, cyclohexane/ethyl acetate=9/1→3/1, V=10 mL) to give **19** (27 mg, 0.08 mmol, 13 %) as colorless solid. m.p.=163‐164 °C; TLC (cyclohexane/ethyl acetate, 2/1 V/V): R_f_=0.68; ^1^H NMR (DMSO‐d_6_): δ [ppm]=3.86 (s, 3H, CO_2_C*H*_3_), 7.03–7.07 (m, 1H, 3′‐H_5‐chlorophenyl_), 7.16–7.22 (m, 2H, 2′′‐H_4‐fluorophenyl_, 6′′‐H_4‐fluorophenyl_), 7.23–7.29 (m, 2H, 3′′‐H_4‐fluorophenyl_, 5′′‐H_4‐fluorophenyl_), 7.61–7.65 (m, 1H, 4′‐H_5‐chlorophenyl_), 7.97–7.98 (m, 1H, 6′‐H_5‐chlorophenyl_), 9.25 (s, 1H, 5‐H_triazole_); ^13^C NMR (DMSO‐d_6_): δ [ppm]=52.0 (1C, CO_2_ *C*H_3_), 116.9 (d, *J*=23.6 Hz, 2C, C‐3′′_4‐fluorophenyl_, C‐5′′_4‐fluorophenyl_), 119.8 (1C, C‐3′_5‐chlorophenyl_), 121.5 (d, *J*=8.6 Hz, 2C, C‐2′′_4‐fluorophenyl_, C‐6′′_4‐fluorophenyl_), 126.7 (1C, C‐6′_5‐chlorophenyl_), 127.2 (1C, C_arom._), 127.5 (1C, C_arom._), 131.0 (1C, C‐5_triazole_), 131.5 (1C, C‐4′_5‐chlorophenyl_), 138.7 (1C, C‐4_triazole_), 149.4 (1C, C‐2′_5‐chlorophenyl_), 150.9 (d, *J*=2.6 Hz, 1C, C‐1′′_4‐fluorophenyl_), 159.0 (d, *J*=241 Hz, 1C, C‐4′′_4‐fluorophenyl_), 160.4 (1C, *C*O_2_CH_3_); IR (neat): $\tilde{\nu }$ [cm^−1^]=3175, 3063, 2924, 1728, 1524, 1504, 1489, 1458, 1435, 1369, 1254, 1211, 1184, 1150, 1123, 1034, 991, 853, 814, 775, 687; HRMS (*m*/*z*): [M+H]^+^ calcd for C_16_H_12_ClFN_3_O_3_, 348.0546; found, 348.0543; HPLC (method 1): t_R_=22.3 min, purity 97.6 %.

**1‐[5‐Chloro‐2‐(4‐fluorophenoxy)phenyl]‐*N*‐hydroxy‐1*H*‐1,2,3‐triazole‐4‐carboxamide (21)**: A 5.4 M solution of sodium methoxide in methanol (0.2 mL, 1.1 mmol) was added to a solution of **19** (39 mg, 0.11 mmol) and hydroxylamine hydrochloride (38 mg, 0.55 mmol) in dry methanol (3 mL). The mixture was stirred at ambient temperature overnight. Then the solvent was removed in vacuo and the residue was purified by automatic flash column chromatography using a Biotage purification apparatus (5 %→50 % ACN in H_2_O, Biotage® SNAP KP‐C18‐HS 12 g). Fractions containing the desired product were combined, dried from acetonitrile under reduced pressure, and subjected to lyophilization to give **21** (37 mg, 0.11 mmol, 95 %) as yellowish solid. m.p.=168 °C (decomposition); ^1^H NMR (DMSO‐d_6_): δ [ppm]=7.02–7.06 (m, 1H, 3′‐H_5‐chlorophenyl_), 7.15–7.20 (m, 2H, 2′′‐H_4‐fluorophenyl_, 6′′‐H_4‐fluorophenyl_), 7.22–7.28 (m, 2H, 3′′‐H_4‐fluorophenyl_, 5′′‐H_4‐fluorophenyl_), 7.53–7.57 (m, 1H, 4′‐H_5‐chlorophenyl_), 7.87–7.90 (m, 1H, 6′‐H_5‐chlorophenyl_), 8.31 (s, 1H, 5‐H_triazole_); ^13^C NMR (DMSO‐d_6_): δ [ppm]=116.9 (d, *J*=23.7 Hz, 2C, C‐3′′_4‐fluorophenyl_, C‐5′′_4‐fluorophenyl_), 120.4 (1C, C‐3′_5‐chlorophenyl_), 121.1 (d, *J*=8.7 Hz, 2C, C‐2′′_4‐fluorophenyl_, C‐6′′_4‐fluorophenyl_), 123.1 (1C, C‐5_triazole_), 125.7 (1C, C‐6′_5‐chlorophenyl_), 127.4 (1C, C‐5′_5‐chlorophenyl_), 128.8 (1C, C‐1′_5‐chlorophenyl_), 130.2 (1C, C‐4′_5‐chlorophenyl_), 147.1 (1C, C‐4_triazole_), 148.4 (1C, C‐2′_5‐chlorophenyl_), 151.3 (d, *J*=2.4 Hz, 1C, C‐1′′_4‐fluorophenyl_), 158.3 (1C, *C*ONHOH), 158.8 (d, *J*=241 Hz, 1C, C‐4′′_4‐fluorophenyl_); IR (neat): $\tilde{\nu }$ [cm^−1^]=3175, 2978, 2886, 1605, 1493, 1454, 1393, 1250, 1219, 1184, 1130, 1092, 1030, 876, 849, 822, 772, 683; HRMS (*m*/*z*): [M+H]^+^ calcd for C_15_H_11_ClFN_4_O_3_, 349.0498; found, 349.0522; HPLC (method 2): t_R_=16.3 min, purity 92.4 %.

**2‐Azido‐4‐chloro‐1‐phenoxybenzene (25)**: 2‐Amino‐4‐chlorophenyl phenyl ether (650 mg, 3.0 mmol) was dissolved in 2 M HCl (10 mL) and the solution was stirred in an ice bath. Then an ice‐cold solution of sodium nitrite (301 mg, 4.4 mmol) in water (1.5 mL) was added dropwise over a period of 5 min. After additional 5 min, urea (27 mg) was added to destroy the excess of nitrous acid. Then an ice‐cold solution of sodium azide (384 mg, 5.9 mmol) and sodium acetate (1.7 mg, 0.02 mmol) in water (8 mL) was added. The mixture was stirred in an ice bath for 2 h. Then it was extracted with ethyl acetate (3×). The combined organic layers were dried (Na_2_SO_4_), filtered, and the solvent was removed in vacuo. The residue was purified by flash column chromatography (Ø=4 cm, h=15 cm, cyclohexane/ethyl acetate=9/1, V=30 mL) to give **25** (459 mg, 1.9 mmol, 63 %) as yellowish oil. ^1^H NMR (DMSO‐d_6_): δ [ppm]=6.95–7.00 (m, 2H, 2′‐H_phenyl_, 6′‐H_phenyl_), 7.02–7.05 (m, 1H, 6‐H_2‐azido‐4‐chlorophenyl_), 7.12–7.17 (m, 1H, 4′‐H_phenyl_), 7.23–7.26 (m, 1H, 5‐H_2‐azido‐4‐chlorophenyl_), 7.36–7.42 (m, 3H, 3‐H_2‐azido‐4‐chlorophenyl_, 3′‐H_phenyl_, 5′‐H_phenyl_); ^13^C NMR (DMSO‐d_6_): δ [ppm]=117.2 (2C, C‐2′_phenyl_, C‐6′_phenyl_), 121.3 (1C, C‐3_2‐azido‐4‐chlorophenyl_), 122.5 (1C, C‐6_2‐azido‐4‐chlorophenyl_), 123.6 (1C, C‐4′_phenyl_), 126.0 (1C, C‐5_2‐azido‐4‐chlorophenyl_), 128.8 (1C, C‐4_2‐azido‐4‐chlorophenyl_), 130.1 (2C, C‐3′_phenyl_, C‐5′_phenyl_), 132.6 (1C, C‐2_2‐azido‐4‐chlorophenyl_), 146.4 (1C, C‐1_2‐azido‐4‐chlorophenyl_), 156.7 (1C, C‐1′_phenyl_); IR (neat): $\tilde{\nu }$ [cm^−1^]=3063, 2106, 1585, 1481, 1400, 1296, 1227, 1196, 1161, 1142, 1107, 833, 748, 691; HRMS (*m*/*z*): [M+H]^+^ calcd for C_12_H_9_ClN_3_O, 246.0429; found, 246.0442; HPLC (method 1): t_R_=24.5 min, purity 99.8 %.

**Methyl 1‐(5‐chloro‐2‐phenoxyphenyl)‐1*H*‐1,2,3‐triazole‐4‐carboxylate (27)**: Methyl propiolate (0.13 mL, 140 mg, 1.6 mmol) was added to a stirring solution of **25** (380 mg, 1.6 mmol) in a 1 : 1 mixture of water and *tert*‐butyl alcohol (10 mL). Then sodium ascorbate (30 mg, 0.15 mmol) and copper(II) sulfate pentahydrate (9 mg, 0.04 mmol) were added and the mixture was stirred for 14 h at room temperature. Then water was added and the mixture was extracted with ethyl acetate (3×). The combined organic layers were dried (Na_2_SO_4_), filtered, and the solvent was removed in vacuo. The residue was purified by flash column chromatography (Ø=4 cm, h=15 cm, cyclohexane/ethyl acetate=9/1→3/1, V=30 mL) to give **27** (350 mg, 1.1 mmol, 69 %) as colorless solid. m.p.=135‐136 °C; TLC (cyclohexane/ethyl acetate, 2/1 V/V): R_f_=0.71; ^1^H NMR (DMSO‐d_6_): δ [ppm]=3.86 (s, 3H, CO_2_C*H*_3_), 7.07–7.12 (m, 3H, 2′′‐H_phenyl_, 6′′‐H_phenyl_, 3′‐H_5‐chloro‐2‐phenoxyphenyl_), 7.19–7.23 (m, 1H, 4′′‐H_phenyl_), 7.39–7.44 (m, 2H, 3′′‐H_phenyl_, 5′′‐H_phenyl_), 7.63–7.66 (m, 1H, 4′‐H_5‐chloro‐2‐phenoxyphenyl_), 7.97–7.99 (m, 1H, 6′‐H_5‐chloro‐2‐phenoxyphenyl_), 9.22 (s, 1H, 5‐H_triazole_); ^13^C NMR (DMSO‐d_6_): δ [ppm]=52.0 (1C, CO_2_ *C*H_3_), 119.2 (2C, C‐2′′_phenyl_, C‐6′′_phenyl_), 120.5 (1C, C‐3′_5‐chloro‐2‐phenoxyphenyl_), 124.9 (1C, C‐4′′_phenyl_), 126.7 (1C, C‐6′_5‐chloro‐2‐phenoxyphenyl_), 127.3 (1C, C_arom._), 127.9 (1C, C_arom._), 130.3 (2C, C‐3′′_phenyl_, C‐5′′_phenyl_), 130.9 (1C, C‐5_triazole_), 131.5 (1C, C‐4′_5‐chloro‐2‐phenoxyphenyl_), 138.7 (1C, C‐4_triazole_), 149.0 (1C, C‐2′_5‐chloro‐2‐phenoxyphenyl_), 155.0 (1C, C‐1′′_phenyl_), 160.4 (1C, *C*O_2_CH_3_); IR (neat): $\tilde{\nu }$ [cm^−1^]=2978, 1744, 1585, 1528, 1485, 1462, 1435, 1369, 1238, 1215, 1153, 1123, 1034, 995, 945, 868, 814, 772, 691; HRMS (*m*/*z*): [M+H]^+^ calcd for C_16_H_13_ClN_3_O_3_, 330.0640; found, 330.0634; HPLC (method 1): t_R_=22.3 min, purity 99.7 %.

**1‐(5‐Chloro‐2‐phenoxyphenyl)‐*N*‐hydroxy‐1*H*‐1,2,3‐triazole‐4‐carboxamide (29)**: A 5.4 M solution of sodium methoxide in methanol (0.6 mL, 3.2 mmol) was added to a solution of **27** (300 mg, 0.91 mmol) and hydroxylamine hydrochloride (250 mg, 3.6 mmol) in dry methanol (12 mL). The mixture was stirred at ambient temperature overnight. Then the solvent was removed in vacuo and the residue was purified by automatic flash column chromatography using a Biotage purification apparatus (5 %→80 % ACN in H_2_O, Biotage® SNAP KP‐C18‐HS 30 g). Fractions containing the desired product were combined, dried from acetonitrile under reduced pressure, and subjected to lyophilization to give **29** (210 mg, 0.64 mmol, 70 %) as colorless solid. m.p.=116‐118 °C; ^1^H NMR (DMSO‐d_6_): δ [ppm]=7.07–7.12 (m, 3H, 2′′‐H_phenyl_, 6′′‐H_phenyl_, 3′‐H_5‐chloro‐2‐phenoxyphenyl_), 7.19–7.23 (m, 1H, 4′′‐H_phenyl_), 7.39–7.43 (m, 2H, 3′′‐H_phenyl_, 5′′‐H_phenyl_), 7.61–7.63 (m, 1H, 4′‐H_5‐chloro‐2‐phenoxyphenyl_), 7.95–7.97 (m, 1H, 6′‐H_5‐chloro‐2‐phenoxyphenyl_), 8.92 (s, 1H, 5‐H_triazole_); ^13^C NMR (DMSO‐d_6_): δ [ppm]=119.1 (2C, C‐2′′_phenyl_, C‐6′′_phenyl_), 120.7 (1C, C‐3′_5‐chloro‐2‐phenoxyphenyl_), 124.8 (1C, C‐4′′_phenyl_), 126.3 (1C, C‐6′_5‐chloro‐2‐phenoxyphenyl_), 127.4 (1C, C‐5′_5‐chloro‐2‐phenoxyphenyl_), 127.6 (1C, C‐5_triazole_), 128.3 (1C, C‐1′_5‐chloro‐2‐phenoxyphenyl_), 130.3 (2C, C‐3′′_phenyl_, C‐5′′_phenyl_), 131.1 (1C, C‐4′_5‐chloro‐2‐phenoxyphenyl_), 141.7 (1C, C‐4_triazole_), 148.6 (1C, C‐2′_5‐chloro‐2‐phenoxyphenyl_), 155.1 (1C, C‐1′′_phenyl_), 157.2 (1C, *C*ONHOH); IR (neat): $\tilde{\nu }$ [cm^−1^]=3152, 2978, 2886, 1647, 1578, 1485, 1458, 1381, 1265, 1238, 1177, 1126, 1030, 876, 768, 691; HRMS (*m*/*z*): [M+H]^+^ calcd for C_15_H_12_ClN_4_O_3_, 331.0592; found, 331.0624; HPLC (method 2): t_R_=16.2 min, purity 99.3 %.

**Methyl 1‐(2‐benzoylphenyl)‐1*H*‐1,2,3‐triazole‐4‐carboxylate (31)**: Methyl propiolate (0.15 mL, 140 mg, 1.7 mmol) was added to a stirring solution of **30** (390 mg, 1.7 mmol) in a 1 : 1 mixture of water and *tert*‐butyl alcohol (10 mL). Then sodium ascorbate (34 mg, 0.17 mmol) and copper(II) sulfate pentahydrate (9 mg, 0.04 mmol) were added and the mixture was stirred for 79 h at room temperature. Then water was added and the mixture was extracted with ethyl acetate (3×). The combined organic layers were dried (Na_2_SO_4_), filtered, and the solvent was removed in vacuo. The residue was purified by flash column chromatography (Ø=3 cm, h=15 cm, cyclohexane/ethyl acetate=4/1→1/1, V=20 mL) to give **31** (225 mg, 0.73 mmol, 42 %) as colorless solid. m.p.=147‐148 °C; TLC (cyclohexane/ethyl acetate, 2/1 V/V): R_f_=0.33; ^1^H NMR (DMSO‐d_6_): δ [ppm]=3.82 (s, 3H, CO_2_C*H*_3_), 7.41–7.46 (m, 2H, 3′′‐H_phenyl_, 5′′‐H_phenyl_), 7.57–7.62 (m, 3H, 2′′‐H_phenyl_, 4′′‐H_phenyl_, 6′′‐H_phenyl_), 7.70–7.73 (m, 1H, 3′‐H_2‐benzoylphenyl_), 7.75–7.79 (m, 1H, 4′‐H_2‐benzoylphenyl_), 7.83–7.89 (m, 2H, 5′‐H_2‐benzoylphenyl_, 6′‐H_2‐benzoylphenyl_), 9.28 (s, 1H, 5‐H_triazole_); ^13^C NMR (DMSO‐d_6_): δ [ppm]=52.0 (1C, CO_2_ *C*H_3_), 125.1 (1C, C‐6′_2‐benzoylphenyl_), 128.6 (2C, C‐3′′_phenyl_, C‐5′′_phenyl_), 129.0 (2C, C‐2′′_phenyl_, C‐6′′_phenyl_), 129.8 (1C, C‐5_triazole_), 129.9 (1C, C‐3′_2‐benzoylphenyl_), 130.2 (1C, C‐4′_2‐benzoylphenyl_), 132.0 (1C, C‐5′_2‐benzoylphenyl_), 133.6 (1C, C‐4′′_phenyl_), 133.7 (1C, C‐1′_2‐benzoylphenyl_), 134.0 (1C, C‐2′_2‐benzoylphenyl_), 135.8 (1C, C‐1′′_phenyl_), 139.1 (1C, C‐4_triazole_), 160.3 (1C, *C*O_2_CH_3_), 193.7 (1C, *C*=O); IR (neat): $\tilde{\nu }$ [cm^−1^]=3113, 3048, 2963, 1721, 1663, 1597, 1531, 1504, 1453, 1369, 1296, 1269, 1231, 1150, 1034, 930, 775, 752, 710, 671, 637; HRMS (*m*/*z*): [M+H]^+^ calcd for C_17_H_14_N_3_O_3_, 308.1030; found, 308.1050; HPLC (method 1): t_R_=19.0 min, purity 99.8 %.

**1‐(2‐Benzoylphenyl)‐*N*‐hydroxy‐1*H*‐1,2,3‐triazole‐4‐carboxamide (32)**: A 5.4 M solution of sodium methoxide in methanol (0.6 mL, 3.2 mmol) was added to a solution of **31** (200 mg, 0.64 mmol) and hydroxylamine hydrochloride (178 mg, 2.6 mmol) in dry methanol (10 mL). The mixture was stirred at ambient temperature overnight. Then the solvent was removed in vacuo and the residue was purified by automatic flash column chromatography using a Biotage purification apparatus (5 %→60 % ACN in H_2_O, Biotage® SNAP KP‐C18‐HS 30 g). Fractions containing the desired product were combined, dried from acetonitrile under reduced pressure, and subjected to lyophilization to give **32** (58 mg, 0.19 mmol, 29 %) as colorless solid. m.p.=114‐116 °C; ^1^H NMR (DMSO‐d_6_): δ [ppm]=7.39–7.45 (m, 2H, 3′′‐H_phenyl_, 5′′‐H_phenyl_), 7.55–7.61 (m, 3H, 2′′‐H_phenyl_, 4′′‐H_phenyl_, 6′′‐H_phenyl_), 7.68–7.71 (m, 1H, 3′‐H_2‐benzoylphenyl_), 7.72–7.77 (m, 1H, 4′‐H_2‐benzoylphenyl_), 7.81–7.89 (m, 2H, 5′‐H_2‐benzoylphenyl_, 6′‐H_2‐benzoylphenyl_), 8.98 (s, 1H, 5‐H_triazole_); ^13^C NMR (DMSO‐d_6_): δ [ppm]=124.6 (1C, C‐6′_2‐benzoylphenyl_), 126.7 (1C, C‐5_triazole_), 128.6 (2C, C‐3′′_phenyl_, C‐5′′_phenyl_), 129.0 (2C, C‐2′′_phenyl_, C‐6′′_phenyl_), 129.7 (1C, C‐3′_2‐benzoylphenyl_), 129.9 (1C, C‐4′_2‐benzoylphenyl_), 131.9 (1C, C‐5′_2‐benzoylphenyl_), 133.5 (1C, C‐4′′_phenyl_), 133.9 (2C, C‐1′_2‐benzoylphenyl_, C‐2′_2‐benzoylphenyl_), 135.8 (1C, C‐1′′_phenyl_), 142.0 (1C, C‐4_triazole_), 156.9 (1C, *C*ONHOH), 193.8 (1C, *C*=O); IR (neat): $\tilde{\nu }$ [cm^−1^]=3144, 2978, 2886, 1655, 1597, 1574, 1497, 1450, 1315, 1273, 1180, 1153, 1030, 930, 876, 768, 702, 633; HRMS (*m*/*z*): [M+H]^+^ calcd for C_16_H_13_N_4_O_3_, 309.0982; found, 309.0992; HPLC (method 2): t_R_=14.4 min, purity 99.5 %.

**X‐ray crystal structures**: Crystallization, X‐ray data collection, structure determination, model building, and refinement were performed as described earlier.^28^ Briefly, smHDAC8 expression was carried out in BL21(DE3) cells in 2xLB medium. Cultures were grown and induced at 37 °C with 0.7 mM IPTG in the presence of 100 μM ZnCl_2_. After overnight incubation at 37 °C, cells were harvested and resuspended in a lysis buffer composed of 150 mM NaCl and 50 mM Tris pH 8.0. Lysis was done by sonication, the lysate was clarified by centrifugation. The supernatant was loaded onto Talon Superflow Metal Affinity Resin (Clontech) pre‐equilibrated with the lysis buffer. The his‐tagged protein was released from the Talon resin by thrombin protease treatment in a buffer composed of 50 mM KCl and 10 mM Tris pH 8.0 and subsequently loaded onto a 16/60 Superdex 200 gel filtration column (GE Healthcare) pre‐equilibrated with a buffer composed of 50 mM KCl, 10 mM Tris‐HCl pH 8.0 and 2 mM DTT. Peak fractions were concentrated with an Amicon Ultra centrifugal filter unit. Diffraction‐quality crystals of the native smHDAC8 enzyme were obtained at 207 °C after 3 days by mixing equal volumes of smHDAC8 (2.5 mg/mL) with reservoir solution composed of 21 % PEG 3350 (Fluka) and 0.05 M Na^+^/K^+^ L‐tartrate, and crystallized using the sitting‐drop vapor diffusion technique. After 3 days, grown crystals were soaked for 20 h in mother liquor supplemented with inhibitor **2 b** at 10 mM. Crystals used for X‐ray data collection were briefly transferred in reservoir solution supplemented with 22 % glycerol and flash‐frozen in liquid nitrogen. The crystallographic data were processed and scaled using XDS.^53^ Phases for smHDAC8/inhibitor complexes were obtained by molecular replacement followed by rigid body refinement against smHDAC8 native structure as a model (4BZ5). The initial models were refined through several cycles of manual building using Coot^54^ and automated refinement with Phenix.^55^ Crystallographic statistics are provided in the Supporting Information, Table S1. The smHDAC8/**2 b** structure has been deposited in the PDB under the PDB code 6TLD.

**Docking**: To prepare protein structures for docking studies, available crystal structures were taken: in‐house crystal structure of smHDAC8 with triazole derivative **2 b** (PDB ID 6TLD) and human HDAC isoform crystal structures downloaded from the Protein Data Bank (http://www.rscb.org)^56^ (hsHDAC8 PDB ID 2 V5X, hsHDAC6 PDB ID 5EDU, hsHDAC1 PDB ID 4BKX). All protein structures were prepared with Protein Preparation Wizard from Schrödinger Suite^57^ in several steps. First, hydrogen atoms and missing side chains were added. Second, solvent molecules were removed. Only one water molecule bound to the conserved catalytic zinc ion coordinating histidine and observed in multiple HDAC crystal structures was considered. Third, structure was optimized by automatic assignment of protonation states and tautomers of the amino acid residues with PROPKA tool at pH 7.0. Finally, optimized structure was minimized using default settings: 0.3 Å restraint of the atom displacement, OPLS 2005 force field.^58^

Ligand preparation was carried out with MOE software.^59^ A random starting conformation for each ligand was generated from SMILES and energy minimized with default settings: Amber10:EHT force field.^60^, ^61^

Molecular docking studies were performed with our well established protocol^3^ using Glide docking program from Schrödinger Suite. Grids were generated with default settings (centroid on ligand), except the grid size for smHDAC8 was specified as 30 Å instead of automatic size determination by the ligand size, because the co‐crystal triazole **2 b** is rather small and default grid size was not enough to accommodate large ligands **2 i**‐**j**. For docking run default standard precision mode was chosen with flexible ligand sampling and enhanced planarity of conjugated π‐systems. No constraints were used. One water molecule described previously was toggled. Twenty poses were submitted for post‐docking energy minimization and ten final poses per each ligand were output and ranked with Glide SP score.

Performance of the docking protocol was evaluated by calculation of RMSD value between co‐crystal ligands binding modes and their docking poses. Low values were observed despite using non‐native protein conformations in some cases: SAHA (**1**), **2 b** and **21** in smHDAC8 were showing RMSD of 1.4 Å, 0.9 Å and 1.1 Å respectively.

### Biological evaluation

**Enzymes and** ***in vitro*** **inhibition assays**: Recombinant human HDAC1 and 6 were purchased from BPS biosciences. Recombinant human HDAC8 was produced as previously described.^5^ Recombinant *sm*HDAC8 enzyme was overproduced in *E. coli* cells and purified by a method previously described.^5^ Inhibition assays of *sm*HDAC8 and human HDACs were performed as described earlier.^3^, ^5^ Briefly, the commercial Fluor de Lys drug discovery kit (BML‐KI178) was used for testing inhibition of *sm*HDAC8 and human HDAC8. Test compounds, Fluor de Lys‐HDAC8 substrate (50 μM) and enzyme were incubated for 90 min at 37 °C with subsequent addition of 50 μL Developer II (BML‐KI176) and further incubation for 45 min at 30 °C. Fluorescence was measured in a plate reader (BMG Polarstar) with excitation at λ=390 nm and emission at λ=460 nm. Inhibition tests of human HDAC1 and 6 were conducted using ZMAL (Cbz‐(Ac)Lys‐AMC) as substrate and trypsin as a developer. After incubation of test compounds, ZMAL (10.5 μM) and enzyme for 90 min at 37 °C, 60 μL of trypsin was added and further incubated for 20 min at 37 °C. Trichostatin A (2 μM) was used in both assays to stop the reaction. Fluorescence was measured similarly as mentioned above. IC_50_ values were determined with OriginPro (version 9.0.0, Northampton, Massachusetts). Values represent mean ± S.E.M.

**Schistosome viability testing**: The resazurin‐based assay to determine the effects of novel inhibitors targeting smHDAC8 on the viability of *S. mansoni* schistosomula was carried out exactly as previously described.^27^, ^62^ Briefly, newly transformed schistosomula (NTS) were obtained *in vitro* as previously described^63^ by mechanical transformation of *S. mansoni* cercaria. A suspension of NTS was prepared at a concentration of 100 per 100 μL using Medium 199 (Invitrogen) supplemented with 10 % fetal calf serum (Gibco), penicillin (50 U×mL^−1^), streptomycin (50 μg×mL^−1^), and rifampicin (60 μg×mL^−1^). Schistosomula were kept in culture for 3 h at 37 °C and 5 % CO_2_ prior to use in screening. Drug stock solutions of 20 mM in DMSO were used. Mid‐dilutions were performed in 100 % DMSO and 1 μL added to 100 μL/well of M199 medium in black 96 well plates (Nunc, UK) with supplemented Medium 199 and 100 μL of the prepared NTS suspension (100 NTS/well). Live and dead schistosomula (treated with 70 % ethanol) were used as positive and negative controls. Experiments were carried out in triplicate wells in two biological replicates and the compounds were tested at final concentrations of 10 and 20 μM. After 48 h of drug exposure, 20 μL of resazurin solution (Abd Serotec) were added to each well. Finally, after 72 h of exposure, the fluorescence intensity of the highly red fluorescent resorufin product was measured using an excitation wavelength of 530 nm and an emission wavelength of 590 nm in an Infinite M200 Pro microplate reader (TECAN). Background fluorescence of the drug containing medium were determined for each drug dilution using wells containing only DMSO as control.

## Supporting information

As a service to our authors and readers, this journal provides supporting information supplied by the authors. Such materials are peer reviewed and may be re‐organized for online delivery, but are not copy‐edited or typeset. Technical support issues arising from supporting information (other than missing files) should be addressed to the authors.

Supplementary
