# Supplementary material for: Structure‐Based Design, Synthesis, and Biological Evaluation of Triazole‐Based smHDAC8 Inhibitors
Source: ChemMedChem. 2020 Jan 9;15(7):571–84. doi: 10.1002/cmdc.201900583 (PMC7187165; doi:10.1002/cmdc.201900583)
Supplement: Supplementary file 1 — Supplementary [file CMDC-15-571-s001.pdf]

CHEMMEDCHEM

Supporting Information

Structure-Based Design, Synthesis, and Biological Evaluation of Triazole-Based smHDAC8 Inhibitors

Dmitrii V. Kalinin, Sunit K. Jana, Maxim Pfafenrot, Alokta Chakrabarti, Jelena Melesina, Tajith B. Shaik, Julien Lancelot, Raymond J. Pierce, Wolfgang Sippl, Christophe Romier, Manfred Jung, and Ralph Holl*© 2019 The Authors. Published by Wiley-VCH Verlag GmbH & Co. KGaA. This is an open access article under the terms of the Creative Commons Attribution License, which permits use, distribution and reproduction in any medium, provided the original work is properly cited.

Contents

Table S1 S2

Figure S1 S3

Synthetic procedures and analytical data of compounds

5c-g,i, **2c-g,i**, **10**, **12**, **14**, **16**, **18**, **20**, **24**, **26**, **28** S4

^{1}H and ^{13}C NMR spectra of representative compounds S22

Table S1: Data collection and refinement statistics for the smHDAC8/**2b** complex (PDB code 6TLD).

**Data collection***	**smHDAC8-2b**
Space group	P1
Cell dimensions	
a, b, c (Å)	70.64 70.72 98.05
α, β, γ (°)	78.08 75.69 85.72
Resolution (Å)	50 – 1.61 (1.71 – 1.61)
Rsym or Rmerge	0.064 (0.110)
I / σI	11.40 (1.04)
Completeness (%)	92.2 (86.3)
Redundancy	3.6 (3.5)
CC(1/2)	99.9 (49.8)
**Refinement**	
Resolution (Å)	49.8 – 1.615
No. reflections	213108
Rwork / Rfree	0.166 / 0.195
Number of atoms	
Protein	13170
Ligand/ion	228
Water	955
B-factors	
Protein	31.27
Ligand/ion	51.78
Water	40.48
R.m.s. deviations	
Bond lengths (Å)	0.007
Bond angles (°)	0.855
** Values in parentheses are for the highest-resolution shell.*	

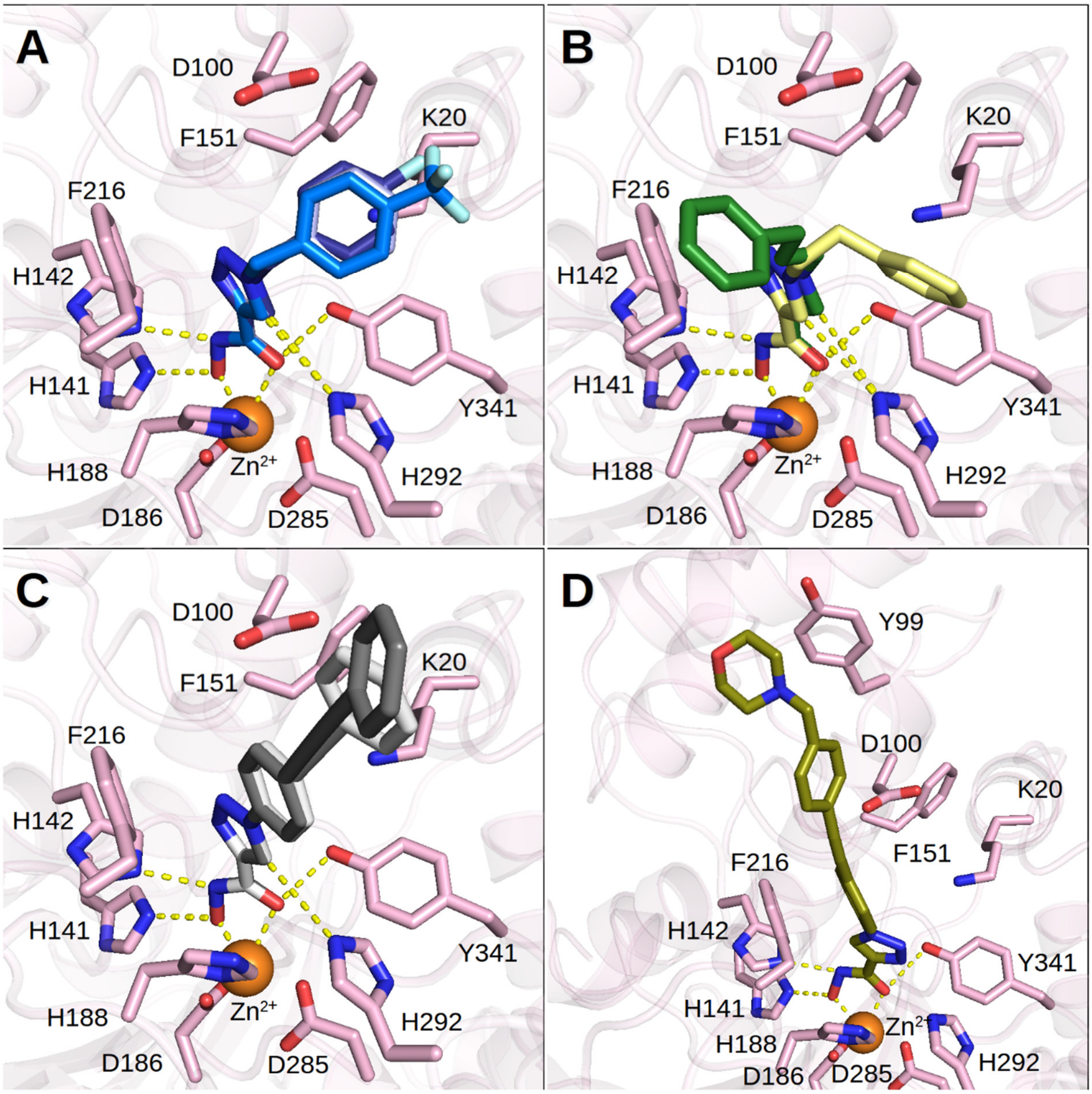

Figure S1: Docking poses of compounds **2c**-**2j** in smHDAC8 (pale pink carbons): (A) **2c** (pale blue carbons), **2d** (dark purple-blue carbons) and **2e** (marine blue carbons), (B) **2f** (pale yellow carbons) and **2g** (forest green carbons), (C) **2h** (white carbons) and **2j** (grey carbons), (D) **2i** (dark olive carbons). Catalytic zinc ion is shown as orange sphere. Nitrogens are colored dark blue, oxygens – red, fluorine – pale cyan. Metal interactions and hydrogen bonds are shown as dashed yellow lines.

Synthetic procedures and analytical data

Methyl 1-benzyl-1*H*-1,2,3-triazole-4-carboxylate (5c)

Methyl propiolate (0.18 mL, 2.0 mmol) and (azidomethyl)benzene (399 mg, 3.0 mmol) were dissolved in a 1:1 mixture of *t*BuOH and H_2O (10 mL). Sodium ascorbate (40 mg, 0.2 mmol) and copper(II) sulfate pentahydrate (10 mg, 0.04 mmol) were added and the mixture was stirred overnight. Then water was added and the mixture was extracted ethyl acetate (3×). The combined organic phases were dried (Na_2SO_4), filtered and evaporated. The residue was purified by flash column chromatography (Ø = 2 cm, h = 15 cm, V = 10 mL, cyclohexane/ethyl acetate = 2:1, R_f = 0.31) to give **5c** as colorless solid (415 mg, 1.9 mmol, 96% yield). m.p. = 108 °C; ^{1}H NMR ($CDCl_3$): δ [ppm] = 3.92 (s, 3H, CO_2CH_3), 5.58 (s, 2H, CH_2Ph), 7.27 – 7.30 (m, 2H, H_{phenyl}), 7.37 – 7.42 (m, 3H, H_{phenyl}), 7.97 (s, 1H, 5-$H_{triazole}$); ^{13}C NMR ($CDCl_3$): δ [ppm] = 52.4 (1C, CO_2CH_3), 54.7 (1C, CH_2Ph), 127.5 (1C, C-$5_{triazole}$), 128.4 (2C, C-$2'_{phenyl}$, C-$6'_{phenyl}$), 129.3 (1C, C-$4'_{phenyl}$), 129.5 (2C, C-$3'_{phenyl}$, C-$5'_{phenyl}$), 133.7 (1C, C-$1'_{phenyl}$), 140.5 (1C, C-$4_{triazole}$), 161.2 (1C, CO_2CH_3); IR (neat): $\tilde{\nu}$ [cm^{-1}] = 2916, 2847, 1728, 1697, 1543, 1435, 1238, 1018, 779, 718; HRMS (m/z): $[M+H]^+$ calcd for $C_{11}H_{12}N_3O_2$ 218.0924; found, 218.0906; HPLC (method 1): t_R = 14.9 min, purity 99.0%.

Methyl 1-(4-fluorobenzyl)-1*H*-1,2,3-triazole-4-carboxylate (5d)

Methyl propiolate (0.18 mL, 2.0 mmol) and 1-(azidomethyl)-4-fluorobenzene (453 mg, 3.0 mmol) were dissolved in a 1:1 mixture of *t*BuOH and H_2O (10 mL). Sodium ascorbate (40 mg, 0.2 mmol) and copper(II) sulfate pentahydrate (10 mg, 0.04 mmol) were added and the mixture was stirred overnight. Then water was added and the

mixture was extracted ethyl acetate (3×). The combined organic phases were dried (Na_2SO_4), filtered and evaporated. The residue was purified by flash column chromatography (Ø = 2 cm, h = 15 cm, V = 10 mL, cyclohexane/ethyl acetate = 1:1, R_f = 0.30) to give **5d** as colorless solid (446 mg, 1.9 mmol, 95% yield). m.p. = 120 °C; ^1H NMR ($CDCl_3$): δ [ppm] = 3.92 (s, 3H, CO_2CH_3), 5.55 (s, 2H, CH_2Ph), 7.05 – 7.11 (m, 2H, 3'-$H_{4\text{-fluorophenyl}}$, 5'-$H_{4\text{-fluorophenyl}}$) 7.27 – 7.31 (m, 2H, 2'-$H_{4\text{-fluorophenyl}}$, 6'-$H_{4\text{-fluorophenyl}}$), 7.99 (s, 5-$H_{triazole}$); ^{13}C NMR ($CDCl_3$): δ [ppm] = 52.5 (1C, CO_2CH_3), 54.0 (1C, CH_2Ph), 116.6 (d, J = 21.9 Hz, 2C, C-3'$_{4\text{-fluorophenyl}}$, C-5'$_{4\text{-fluorophenyl}}$), 127.5 (1C, C-5$_{triazole}$), 129.8 (d, J = 3.4 Hz, 1C, C-1'$_{4\text{-fluorophenyl}}$), 130.4 (d, J = 8.5 Hz, 2C, C-2'$_{4\text{-fluorophenyl}}$, C-6'$_{4\text{-fluorophenyl}}$), 140.6 (1C, C-4$_{triazole}$), 161.2 (1C, CO_2CH_3), 163.3 (d, J = 249 Hz, 1C, C-4'$_{4\text{-fluorophenyl}}$); IR (neat): $\tilde{\nu}$ [cm^{-1}] = 3117, 1721, 1605, 1539, 1508, 1435, 1342, 1223, 1045, 1015, 772; HRMS (m/z): $[M+H]^+$ calcd for $C_{11}H_{11}FN_3O_2$, 236.0830; found, 236.0846; HPLC (method 1): t_R = 15.3 min, purity 98.6%.

Methyl 1-[4-(trifluoromethyl)benzyl]-1*H*-1,2,3-triazole-4-carboxylate (5e)

Methyl propiolate (0.18 mL, 2.0 mmol) and 1-(azidomethyl)-4-(trifluoromethyl)benzene (603 mg, 3.0 mmol) were dissolved in a 1:1 mixture of *t*BuOH and H_2O (10 mL). Sodium ascorbate (40 mg, 0.2 mmol) and copper(II) sulfate pentahydrate (10 mg, 0.04 mmol) were added and the mixture was stirred overnight. Then water was added and the mixture was extracted ethyl acetate (3×). The combined organic phases were dried (Na_2SO_4), filtered and evaporated. The residue was purified by flash column chromatography (Ø = 2 cm, h = 15 cm, V = 10 mL, cyclohexane/ethyl acetate = 1:1, R_f = 0.30) to give **5e** as colorless solid (530 mg, 1.9 mmol, 93% yield). m.p. = 143 °C; ^1H NMR ($CDCl_3$): δ [ppm] = 3.93 (s, 3H, CO_2CH_3), 5.65 (s, 2H, CH_2Ph), 7.38 – 7.41 (m, 2H, 2'-$H_{4\text{-trifluoromethylphenyl}}$, 6'-$H_{4\text{-trifluoromethylphenyl}}$), 7.64 – 7.67 (m, 2H, 3'-$H_{4\text{-trifluoromethylphenyl}}$,

5'-$H_{4\text{-trifluoromethylphenyl}}$), 8.04 (s, 5-$H_{\text{triazole}}$); ^{13}C NMR ($CDCl_3$): δ [ppm] = 52.4 (1C, CO_2CH_3), 53.9 (1C, CH_2Ph), 123.8 (q, J = 272 Hz, 1C, CF_3), 126.5 (q, J = 3.8 Hz, 2C, C-3'$_{4\text{-trifluoromethylphenyl}}$, C-5'$_{4\text{-trifluoromethylphenyl}}$), 127.6 (1C, C-5$_{\text{triazole}}$), 128.5 (2C, C-2'$_{4\text{-trifluoromethylphenyl}}$, C-6'$_{4\text{-trifluoromethylphenyl}}$), 131.6 (q, J = 32.4 Hz, 1C, C-4'$_{4\text{-trifluoromethylphenyl}}$), 137.8 (1C, C-1'$_{4\text{-trifluoromethylphenyl}}$), 140.8 (1C, C-4$_{\text{triazole}}$), 161.0 (1C, CO_2CH_3); IR (neat): $\tilde{\nu}$ [cm^{-1}] = 3129, 2955, 1728, 1543, 1427, 1327, 1231, 1157, 1115, 1049, 1018, 814, 775; HRMS (m/z): $[M+H]^+$ calcd for $C_{12}H_{11}F_3N_3O_2$, 286.0798; found, 286.0846; HPLC (method 1): t_R = 18.0 min, purity 99.3%.

Methyl 1-phenethyl-1*H*-1,2,3-triazole-4-carboxylate (5f)

Methyl propiolate (0.18 mL, 2.0 mmol) and (2-azidoethyl)benzene (442 mg, 3.0 mmol) were dissolved in a 1:1 mixture of *t*BuOH and H_2O (10 mL). Sodium ascorbate (40 mg, 0.2 mmol) and copper(II) sulfate pentahydrate (10 mg, 0.04 mmol) were added and the mixture was stirred overnight. Then water was added and the mixture was extracted ethyl acetate (3×). The combined organic phases were dried (Na_2SO_4), filtered and evaporated. The residue was purified by flash column chromatography (Ø = 2 cm, h = 15 cm, V = 10 mL, cyclohexane/ethyl acetate = 2:1, R_f = 0.31) to give **5f** as colorless solid (393 mg, 1.7 mmol, 85% yield). m.p. = 101 °C; ^{1}H NMR (CD_3OD): δ [ppm] = 3.23 (t, J = 7.0 Hz, 2H, CH_2CH_2Ph), 3.87 (s, 3H, CO_2CH_3), 4.68 – 4.74 (m, 2H, CH_2CH_2Ph), 7.10 – 7.15 (m, 2H, H_{phenyl}), 7.17 – 7.29 (m, 3H, H_{phenyl}), 8.30 (s, 5-H_{triazole}); ^{13}C NMR (CD_3OD): δ [ppm] = 37.2 (1C, CH_2CH_2Ph), 52.4 (1C, CO_2CH_3), 52.9 (1C, CH_2CH_2Ph), 128.0 (1C, C-4'$_{\text{phenyl}}$), 129.7 (2C, C-3'$_{\text{phenyl}}$, C-5'$_{\text{phenyl}}$), 129.8 (3C, C-2'$_{\text{phenyl}}$, C-6'$_{\text{phenyl}}$, C-5$_{\text{triazole}}$), 138.3 (1C, C-1'$_{\text{phenyl}}$), 140.1 (1C, C-4$_{\text{triazole}}$), 162.3 (1C, CO_2CH_3); IR (neat): $\tilde{\nu}$ [cm^{-1}] = 3121, 2959, 1709, 1543, 1435, 1354, 1231, 1049, 1018, 752, 698; HRMS

(m/z): $[M+H]^+$ calcd for $C_{12}H_{14}N_3O_2$, 232.1081; found, 232.1098; HPLC (method 1): t_R = 15.4 min, purity 99.8%.

Methyl 1-(3-phenylpropyl)-1*H*-1,2,3-triazole-4-carboxylate (5g)

Methyl propiolate (0.18 mL, 2.0 mmol) and (3-azidopropyl)benzene (484 mg, 3.0 mmol) were dissolved in a 1:1 mixture of *t*BuOH and H_2O (10 mL). Sodium ascorbate (40 mg, 0.2 mmol) and copper(II) sulfate pentahydrate (10 mg, 0.04 mmol) were added and the mixture was stirred overnight. Then water was added and the mixture was extracted ethyl acetate (3×). The combined organic phases were dried (Na_2SO_4), filtered and evaporated. The residue was purified by flash column chromatography (Ø = 2 cm, h = 15 cm, V = 10 mL, cyclohexane/ethyl acetate = 2:1, R_f = 0.31) to give **5g** as colorless solid (383 mg, 1.6 mmol, 78% yield). m.p. = 102 °C; ^{1}H NMR ($CDCl_3$): δ [ppm] = 2.25 – 2.33 (m, 2H, $CH_2C\mathit{H}_2CH_2Ph$), 2.66 (t, *J* = 7.5 Hz, 2H, $CH_2CH_2C\mathit{H}_2Ph$), 3.96 (s, 3H, $CO_2C\mathit{H}_3$), 4.41 (t, *J* = 7.2 Hz, 2H, $C\mathit{H}_2CH_2CH_2Ph$), 7.14 – 7.17 (m, 2H, 2'-H_{phenyl}, 6'-H_{phenyl}), 7.20 – 7.25 (m, 1H, 4'-H_{phenyl}), 7.28 – 7.33 (m, 2H, 3'-H_{phenyl}, 5'-H_{phenyl}), 8.05 (s, 5-$H_{triazole}$); ^{13}C NMR ($CDCl_3$): δ [ppm] = 31.6 (1C, $CH_2\mathit{C}H_2CH_2Ph$), 32.5 (1C, $CH_2CH_2\mathit{C}H_2Ph$), 50.0 (1C, $\mathit{C}H_2CH_2CH_2Ph$), 52.4 (1C, $CO_2\mathit{C}H_3$), 126.7 (1C, C-4'$_{phenyl}$), 127.5 (1C, C-5$_{triazole}$), 128.5 (2C, C-2'$_{phenyl}$, C-6'$_{phenyl}$), 128.8 (2C, C-3'$_{phenyl}$, C-5'$_{phenyl}$), 139.8 (1C, C-1'$_{phenyl}$) 140.1 (1C, C-4$_{triazole}$), 161.3 (1C, $\mathit{C}O_2CH_3$); IR (neat): $\tilde{\nu}$ [cm^{-1}] = 2955, 1713, 1697, 1539, 1431, 1346, 1223, 1053, 1011, 779, 745, 698; HRMS (m/z): $[M+H]^+$ calcd for $C_{13}H_{16}N_3O_2$, 246.1237; found, 246.1218; HPLC (method 1): t_R = 17.3 min, purity 99.0%.

Methyl 1-(4-{[4-(morpholinomethyl)phenyl]ethynyl}phenyl)-1*H*-1,2,3-triazole-4-carboxylate (5i)

Under N_2 atmosphere tetrakis(triphenylphosphine)palladium(0) (6 mg, 0.005 mmol) and copper(I) iodide (2 mg, 0.01 mmol) were added to a solution of **5a** (165 mg, 0.5 mmol) 1:2 mixture of triethylamine and DMF (15 mL). 4-(4-ethynylbenzyl)morpholine (121 mg, 0.6 mmol) was added in portions and the mixture was stirred overnight at 70 °C. Then the reaction mixture was diluted with water and extracted with ethyl acetate (3×). The combined organic phases were dried with Na_2SO_4, filtered and the solvent was removed in vacuo. The residue was purified by flash column chromatography (Ø = 2 cm, h = 15 cm, V = 10 mL, cyclohexane/ethyl acetate = 1:2, R_f = 0.31) to give **5i** as colorless crystalline solid (197 mg, 0.49 mmol, 98% yield). m.p. = 171 °C; ^{1}H NMR ($CDCl_3$): δ [ppm] = 2.43 – 2.48 (m, 4H, NCH_2CH_2O), 3.52 (s, 2H, NCH_2Ar), 3.70 – 3.74 (m, 4H, NCH_2CH_2O), 4.01 (s, 3H, CO_2CH_3), 7.33 – 7.37 (m, 2H, $H_{arom.}$), 7.49 – 7.53 (m, 2H, $H_{arom.}$), 7.68 – 7.72 (m, 2H, $H_{arom.}$), 7.76 – 7.79 (m, 2H, $H_{arom.}$), 8.54 (s, 5-$H_{triazole}$); ^{13}C NMR ($CDCl_3$): δ [ppm] = 52.6 (1C, CO_2CH_3), 53.8 (2C, NCH_2CH_2O), 63.3 (1C, NCH_2Ar), 67.1 (2C, NCH_2CH_2O), 87.8 (1C, *C*≡C), 91.9 (1C, *C*≡C), 120.7 (2C, $C_{arom.}$), 121.4 (1C, $C_{arom.}$), 125.1 (1C, $C_{arom.}$), 125.5 (1C, C-$5_{triazole}$), 129.3 (2C, $C_{arom.}$), 131.8 (2C, $C_{arom.}$), 133.2 (2C, $C_{arom.}$), 135.6 (1C, $C_{arom.}$), 139.1 (1C, $C_{arom.}$), 140.8 (1C, C-$4_{triazole}$), 161.1 (1C, CO_2CH_3); IR (neat): $\tilde{\nu}$ [cm^{-1}] = 2947, 2808, 1724, 1547, 1520, 1261, 1115, 1034, 1007, 860, 841, 768; HRMS (m/z): $[M+H]^+$ calcd for $C_{23}H_{23}N_4O_3$, 403.1765; found, 403.1723; HPLC (method 1): t_R = 16.9 min, purity 98.6%.

1-Benzyl-*N*-hydroxy-1*H*-1,2,3-triazole-4-carboxamide (2c)

Hydroxylamine hydrochloride (139 mg, 2.0 mmol) and a 2.0 M solution of sodium methoxide in methanol (1.5 mL, 3.0 mmol) were added to a solution of **5c** (217 mg, 1.0

mmol) in dry methanol (8 mL). The reaction mixture was stirred at ambient temperature for 20 h until TLC showed complete conversion of the ester. The reaction mixture was acidified with 1.0 M HCl to pH 5-6. Then the mixture was extracted with ethyl acetate (3×). The combined organic phases were dried with Na_2SO_4, filtered and the solvent was dried in vacuo. The crude mixture was purified by flash column chromatography (Ø = 2 cm, h = 15 cm, V = 10 mL, dichloromethane/methanol= 10:1, R_f = 0.37) to give **2c** as colorless crystalline solid (157 mg, 0.72 mmol, 72%). m.p. = 183 °C; ^{1}H NMR (DMSO-d_6): δ [ppm] = 5.64 (s, 2H, C*H*$_2$Ph), 7.31 – 7.42 (m, 5H, H_{phenyl}), 8.60 (s, 5-H $_{triazole}$), 9.06 (s br, 1H, CON*HOH*), 11.28 (s br, 1H, CON*HOH*); ^{13}C NMR (DMSO-d_6): δ [ppm] = 53.0 (1C, *C*H$_2$Ph), 126.3 (1C, C-5$_{triazole}$), 128.1 (2C, C-2'$_{phenyl}$, C-6'$_{phenyl}$), 128.3 (1C, C-4'$_{phenyl}$), 128.9 (2C, C-3'$_{phenyl}$, C-5'$_{phenyl}$), 135.7 (1C, C-1'$_{phenyl}$), 141.5 (1C, C-4$_{triazole}$), 157.6 (1C, *C*ONHOH); IR (neat): $\tilde{\nu}$ [cm^{-1}] = 3333, 3109, 2859, 1620, 1574, 1454, 1435, 1254, 1234, 1123, 1045, 880, 694; HRMS (m/z): [M+H]$^+$ calcd for $C_{10}H_{11}N_4O_2$, 219.0877; found, 219.0864; HPLC (method 2): t_R = 12.6 min, purity 99.9%.

1-(4-Fluorobenzyl)-*N*-hydroxy-1*H*-1,2,3-triazole-4-carboxamide (2d)

Hydroxylamine hydrochloride (89 mg, 1.3 mmol) and a 2.0 M solution of sodium methoxide in methanol (0.96 mL, 1.9 mmol) were added to a solution of **5d** (150 mg, 0.64 mmol) in dry methanol (10 mL). The reaction mixture was stirred at ambient temperature for 20 h until TLC showed complete conversion of the ester. The reaction mixture was acidified with 1.0 M HCl to pH 5-6. Then the mixture was extracted with ethyl acetate (3×). The combined organic phases were dried with Na_2SO_4, filtered and the solvent was dried in vacuo. The crude mixture was purified by flash column chromatography (Ø = 2 cm, h = 15 cm, V = 10 mL, dichloromethane/methanol= 10:1, R_f = 0.37) to give **2d** as colorless crystalline solid (83 mg, 0.35 mmol, 55%). m.p. = 181

°C; ^{1}H NMR (CD_3OD): δ [ppm] = 5.63 (s, 2H, CH_2Ph), 7.09 - 7.14 (m, 2H, 3'-$H_{4\text{-fluorophenyl}}$, 5'-$H_{4\text{-fluorophenyl}}$), 7.38 – 7.42 (m, 2H, 2'-$H_{4\text{-fluorophenyl}}$, 6'-$H_{4\text{-fluorophenyl}}$), 8.35 (s, 5-$H_{\text{triazole}}$); ^{13}C NMR ($CD_3OD$): δ [ppm] = 54.2 (1C, CH_2Ph), 116.8 (d, J = 22.0 Hz, 2C, C-3'$_{4\text{-fluorophenyl}}$, C-5'$_{4\text{-fluorophenyl}}$), 127.1 (1C, C-5$_{\text{triazole}}$), 131.5 (d, J = 8.5 Hz, 2C, C-2'$_{4\text{-fluorophenyl}}$, C-6'$_{4\text{-fluorophenyl}}$), 132.6 (d, J = 2.8 Hz, 1C, C-1'$_{4\text{-fluorophenyl}}$), 142.5 (1C, C-4$_{\text{triazole}}$), 160.5 (1C, *C*ONHOH), 164.3 (d, J = 246 Hz, 1C, C-4'$_{4\text{-fluorophenyl}}$); IR (neat): $\tilde{\nu}$ [cm^{-1}] = 3314, 3125, 1620, 1585, 1508, 1450, 1234, 1157, 1130, 1042, 880, 775, 748; HRMS (m/z): $[M+H]^+$ calcd for $C_{10}H_{10}FN_4O_2$, 237.0782; found, 237.0759; HPLC (method 2): t_R = 12.8 min, purity 99.9%.

1-[4-(Trifluoromethyl)benzyl]-*N*-hydroxy-1*H*-1,2,3-triazole-4-carboxamide (2e)

Hydroxylamine hydrochloride (70 mg, 1.0 mmol) and a 2.0 M solution of sodium methoxide in methanol (0.75 mL, 1.5 mmol) were added to a solution of **5e** (142 mg, 0.5 mmol) in dry methanol (10 mL). The reaction mixture was stirred at ambient temperature for 20 h until TLC showed complete conversion of the ester. The reaction mixture was acidified with 1.0 M HCl to pH 5-6. Then the mixture was extracted with ethyl acetate (3×). The combined organic phases were dried with Na_2SO_4, filtered and the solvent was removed in vacuo. The crude mixture was purified by flash column chromatography (Ø = 2 cm, h = 15 cm, V = 10 mL, dichloromethane/methanol= 10:1, R_f = 0.37) to give **2e** as colorless crystalline solid (69 mg, 0.24 mmol, 48% yield). m.p. = 192 °C; ^{1}H NMR (DMSO-d_6): δ [ppm] = 5.77 (s, 2H, CH_2Ar), 7.52 – 7.54 (m, 2H, 2'-$H_{4\text{-trifluoromethylphenyl}}$, 6'-$H_{4\text{-trifluoromethylphenyl}}$) 7.75 – 7.77 (m, 2H, 3'-$H_{4\text{-trifluoromethylphenyl}}$, 5'-$H_{4\text{-trifluoromethylphenyl}}$), 8.66 (s, 5-$H_{\text{triazole}}$), 9.07 (s br, 1H, N*HOH*), 11.29 (s br, 1H, N*HOH*); ^{13}C NMR (DMSO-d_6): δ [ppm] = 52.4 (1C, CH_2Ph), 124.1 (q, J = 272 Hz, 1C, ArCF_3) 125.7 (q, J = 3.8 Hz, 2C, C-3'$_{4\text{-trifluoromethylphenyl}}$, C-5'$_{4\text{-trifluoromethylphenyl}}$), 126.6 (1C, C-5$_{\text{triazole}}$),

128.7 (2C, C-2'$_{4\text{-trifluoromethylphenyl}}$, C-6'$_{4\text{-trifluoromethylphenyl}}$), 128.8 (q, *J* = 32 Hz, 1C, C-4'$_{4\text{-trifluoromethylphenyl}}$), 140.3 (1C, C-1'$_{4\text{-trifluoromethylphenyl}}$), 141.6 (1C, C-4$_{triazole}$), 157.5 (1C, *C*ONHOH); IR (neat): $\tilde{\nu}$ [cm^{-1}] = 3314, 3102, 1655, 1632, 1570, 1458, 1416, 1327, 1242, 1161, 1103, 1069, 1042, 880, 818, 772; HRMS (m/z): $[M+H]^+$ calcd for $C_{11}H_{10}F_3N_4O_2$, 287.0750; found, 287.0761; HPLC (method 2): t_R = 14.4 min, purity 98.7%.

***N*-Hydroxy-1-phenethyl-1*H*-1,2,3-triazole-4-carboxamide (2f)**

Hydroxylamine hydrochloride (139 mg, 2.0 mmol) and a 2.0 M solution of sodium methoxide in methanol (1.5 mL, 3.0 mmol) were added to a solution of **5f** (231 mg, 1.0 mmol) in dry methanol (10 mL). The reaction mixture was stirred at ambient temperature for 20 h until TLC showed complete conversion of the ester. The reaction mixture was acidified with 1.0 M HCl to pH 5-6. Then the mixture was extracted with ethyl acetate (3×). The combined organic phases were dried with Na_2SO_4, filtered and the solvent was dried in vacuo. The crude mixture was purified by flash column chromatography (Ø = 2 cm, h = 15 cm, V = 10 mL, dichloromethane/methanol= 10:1, R_f = 0.37) to give **2f** as colorless crystalline solid (155 mg, 0.67 mmol, 67%). m.p. = 178 °C; ^{1}H NMR (CD_3OD): δ [ppm] = 3.22 (t, *J* = 6.8 Hz, 2H, $CH_2C\mathit{H}_2Ph$), 4.69 (t, *J* = 6.8 Hz, 2H, $C\mathit{H}_2CH_2Ph$), 7.11 – 7.15 (m, 2H, 2'-H$_{phenyl}$, 6'-H$_{phenyl}$), 7.17 – 7.28 (m, 3H, 3'-H$_{phenyl}$, 4'-H$_{phenyl}$, 5'-H$_{phenyl}$), 8.14 (s, 5-H$_{triazole}$); ^{13}C NMR (CD_3OD): δ [ppm] = 37.3 (1C, $CH_2\mathit{C}H_2Ph$), 52.8 (1C, $\mathit{C}H_2CH_2Ph$), 127.3 (1C, C-5$_{triazole}$), 128.0 (1C, C-4'$_{phenyl}$), 129.7 (2C, C-3'$_{phenyl}$, C-5'$_{phenyl}$), 129.8 (2C, C-2'$_{phenyl}$, C-6'$_{phenyl}$), 138.5 (1C, C-1'$_{phenyl}$), 141.9 (1C, C-4$_{triazole}$), 160.5 (1C, *C*ONHOH); IR (neat): $\tilde{\nu}$ [cm^{-1}] = 3310, 3109, 1620, 1574, 1454, 1238, 1049, 880, 698; HRMS (m/z): $[M+H]^+$ calcd for $C_{11}H_{13}N_4O_2$, 233.1033; found, 233.1063; HPLC (method 2): t_R = 12.8 min, purity 98.9%.

N-Hydroxy-1-(3-phenylpropyl)-1H-1,2,3-triazole-4-carboxamide (2g)

Hydroxylamine hydrochloride (139 mg, 2.0 mmol) and a 2.0 M solution of sodium methoxide in methanol (1.5 mL, 3.0 mmol) were added to a solution of **5g** (245 mg, 1.0 mmol) in dry methanol (10 mL). The reaction mixture was stirred at ambient temperature for 20 h until TLC showed complete conversion of the ester. The reaction mixture was acidified with 1.0 M HCl to pH 5-6. Then the mixture was extracted with ethyl acetate (3×). The combined organic phases were dried with Na_2SO_4, filtered and the solvent was dried in vacuo. The crude mixture was purified by flash column chromatography (Ø = 2 cm, h = 15 cm, V = 10 mL, dichloromethane/methanol= 10:1, R_f = 0.37) to give **2g** as colorless crystalline solid (150 mg, 0.61 mmol, 61%). m.p. = 182 °C; ^{1}H NMR (DMSO-d_6): δ [ppm] = 2.09 – 2.19 (m, 2H, $CH_2CH_2CH_2Ph$), 2.52 – 2.58 (m, 2H, $CH_2CH_2CH_2Ph$), 4.40 (t, *J* = 7.0 Hz, 2H, $CH_2CH_2CH_2Ph$), 7.15 – 7.22 (m, 3H, H_{phenyl}), 7.25 – 7.31 (m, 2H, H_{phenyl}), 8.54 (s, 5-$H_{triazole}$); ^{13}C NMR (DMSO-d_6): δ [ppm] = 31.4 (1C, $CH_2CH_2CH_2Ph$), 32.0 (1C, $CH_2CH_2CH_2Ph$), 49.4 (1C, $CH_2CH_2CH_2Ph$), 126.2 (2C, C-4'$_{phenyl}$, C-5$_{triazole}$), 128.5 (2C, C_{phenyl}), 128.6 (2C, C_{phenyl}), 140.8 (1C, C-1'$_{phenyl}$), 141.5 (1C, C-4$_{triazole}$), 157.9 (1C, CO_2NHOH); IR (neat): $\tilde{\nu}$ [cm^{-1}] = 3291, 3132, 2893, 1643, 1574, 1493, 1454, 1234, 1150, 1123, 1049, 880, 745, 698; HRMS (m/z): $[M+H]^+$ calcd for $C_{12}H_{15}N_4O_2$, 247.1190; found, 247.1198; HPLC (method 2): t_R = 14.1 min, purity 96.2%.

N-Hydroxy-1-(4-{2-[4-(morpholinomethyl)phenyl]ethynyl}phenyl)-1H-1,2,3-triazole-4-carboxamide hydrochloride (2i)

Hydroxylamine hydrochloride (259 mg, 3.7 mmol) and a 2.0 M solution of sodium methoxide in methanol (1.85 mL, 3.7 mmol) were added to a solution of **5i** (150 mg, 0.37 mmol) in dry methanol (10 mL). The reaction mixture was stirred at ambient

temperature for 20 h until TLC showed complete conversion of the ester. The reaction mixture was acidified with a 1.0 M solution of HCl until pH 5-6 was reached. The precipitate was washed with dichloromethane, water and ethyl acetate and in vacuo for 4 h to give **2i** as colorless solid (125 mg, 0.28 mmol, 76% yield). TLC (CH_2Cl_2:methanol = 9:1): R_f = 0.24; m.p. = 239 °C (decomposition); ^{1}H NMR (DMSO-d_6): δ [ppm] = 3.02 – 3.27 (m, 4H, NC*H*$_2$CH$_2$O), 3.77 – 3.98 (m, 4H, NCH$_2$C*H*$_2$O), 4.37 (s, 2H, NC*H*$_2$Ar), 7.65 – 7.74 (m, 4H, 2''-H$_{4\text{-(morpholinomethyl)phenyl}}$, 3''-H$_{4\text{-(morpholinomethyl)phenyl}}$, 5''-H$_{4\text{-(morpholinomethyl)phenyl}}$, 6''-H$_{4\text{-(morpholinomethyl)phenyl}}$), 7.78 – 7.82 (m, 2H, 3'-H$_{4\text{-\{2-[4-(morpholinomethyl)phenyl]ethynyl\}phenyl}}$, 5'-H$_{4\text{-\{2-[4-(morpholinomethyl)phenyl]ethynyl\}phenyl}}$), 8.03 – 8.08 (m, 2H, 2'-H$_{4\text{-\{2-[4-(morpholinomethyl)phenyl]ethynyl\}phenyl}}$, 6'-H$_{4\text{-\{2-[4-(morpholinomethyl)phenyl]ethynyl\}phenyl}}$), 9.19 (s br, 1H, N*HOH*), 9.35 (s, 5-H$_{triazole}$), 11.46 (s, 1H, N*HOH*), 11.55 (s br, 1H, R_3NH^+); ^{13}C NMR (DMSO-d_6): δ [ppm] = 50.7 (2C, N*C*H$_2$CH$_2$O), 58.5 (1C, N*C*H$_2$Ar), 63.0 (2C, NCH$_2$*C*H$_2$O), 89.3 (1C, *C*≡C), 90.2 (1C, *C*≡C), 120.6 (2C, C-2'$_{4\text{-\{2-[4-(morpholinomethyl)phenyl]ethynyl\}phenyl}}$, C-6'$_{4\text{-\{2-[4-(morpholinomethyl)phenyl]ethynyl\}phenyl}}$), 122.6 (1C, C-1'$_{4\text{-\{2-[4-(morpholinomethyl)phenyl]ethynyl\}phenyl}}$), 123.0 (1C, C-1''$_{4\text{-(morpholinomethyl)phenyl}}$), 124.5 (1C, C-5$_{triazole}$), 130.1 (1C, C-4''$_{4\text{-(morpholinomethyl)phenyl}}$), 131.7 (2C, C-2''$_{4\text{-(morpholinomethyl)phenyl}}$, C-6''$_{4\text{-(morpholinomethyl)phenyl}}$), 131.9 (2C, C-3''$_{4\text{-(morpholinomethyl)phenyl}}$, C-5''$_{4\text{-(morpholinomethyl)phenyl}}$), 133.0 (2C, C-3'$_{4\text{-\{2-[4-(morpholinomethyl)phenyl]ethynyl\}phenyl}}$, C-5'$_{4\text{-\{2-[4-(morpholinomethyl)phenyl]ethynyl\}phenyl}}$), 136.1 (1C, C-4'$_{4\text{-\{2-[4-(morpholinomethyl)phenyl]ethynyl\}phenyl}}$), 142.4 (1C, C-4$_{triazole}$), 157.1 (1C, *C*ONHOH); IR (neat): $\tilde{\nu}$ [cm^{-1}] = 3356, 2654, 1686, 1558, 1524, 1489, 1435, 1408, 1258, 1130, 1034, 964, 914, 868, 833, 698, 679; HRMS (m/z): $[M+H]^+$ calcd for $C_{22}H_{22}N_5O_3$, 404.1717; found, 404.1731; HPLC (method 2): t_R = 13.0 min, purity 98.3%.

Methyl 1-(2-hydroxyphenyl)-1*H*-1,2,3-triazole-4-carboxylate (10)

Methyl propiolate (0.36 mL, 336 mg, 4.0 mmol) was added to a stirring solution of **8** (405 mg, 3.0 mmol) in a 1:1 mixture of water and *tert*-butyl alcohol (15 mL). Then sodium ascorbate (40 mg, 0.20 mmol) and copper(II) sulfate pentahydrate (10 mg, 0.04 mmol) were added and the mixture was stirred for 18 h at room temperature. Then water was added and the mixture was extracted with ethyl acetate (3×). The combined organic layers were dried (Na_2SO_4), filtered and the solvent was removed in vacuo. The residue was purified by flash column chromatography (Ø = 2 cm, h = 15 cm, cyclohexane/ethyl acetate = 2/1, V = 10 mL, R_f = 0.51) to give **10** (605 mg, 2.8 mmol, 92%) as brown solid. m.p. = 126 °C; ^{1}H NMR (D_3COD): δ [ppm] = 3.95 (s, 3H, CO_2CH_3), 7.00 – 7.05 (m, 1H, 5-H'_{phenyl}), 7.07 – 7.10 (m, 1H, 3-H'_{phenyl}), 7.34 – 7.39 (m, 1H, 4-H'_{phenyl}), 7.69 – 7.73 (m, 1H, 6-H'_{phenyl}), 8.92 (s, 1H, 5-$H_{triazole}$); ^{13}C NMR (D_3COD): δ [ppm] = 52.6 (1C, CO_2CH_3), 118.0 (1C, C-3'$_{phenyl}$), 121.1 (1C, C-5'$_{phenyl}$), 125.5 (1C, C-1'$_{phenyl}$), 126.0 (1C, C-6'$_{phenyl}$), 131.1 (1C, C-5$_{triazole}$), 131.9 (1C, C-4'$_{phenyl}$), 140.2 (1C, C-4$_{triazole}$), 150.9 (1C, C-2'$_{phenyl}$), 162.5 (1C, CO_2CH_3); IR (neat): $\tilde{\nu}$ [cm^{-1}] = 3021, 2955, 1732, 1601, 1532, 1435, 1373, 1219, 1153, 1057, 752; HRMS (*m*/*z*): [M+H]$^+$ calcd for $C_{10}H_{10}N_3O_3$, 220.0717; found, 220.0722; HPLC (method 1): t_R = 15.2 min, purity 99.9 %.

***N*-Hydroxy-1-(2-hydroxyphenyl)-1*H*-1,2,3-triazole-4-carboxamide (12)**

Hydroxylamine hydrochloride (165 mg, 2.4 mmol) and a 2 M solution of sodium methoxide in methanol (1.5 mL, 3.0 mmol) were added to a solution of **10** (87 mg, 0.40 mmol) in dry methanol (10 mL) and the mixture was stirred at ambient temperature for 20 h. Then water was added. The mixture was acidified with 1 M HCl to pH 5-6 and extracted with ethyl acetate (3×). The combined organic layers were dried (Na_2SO_4),

filtered and the solvent was removed in vacuo. The residue was purified by automatic flash column chromatography (100 % H_2O → 100 % CH_3CN, Biotage SNAP KP-C18-HS 12 g) to give **12** as colorless solid (40 mg, 0.18 mmol, 45% yield). m.p. = 210 °C; TLC (dichloromethane/methanol, 10/1 V/V): R_f = 0.36; ^{1}H NMR (DMSO-d$_6$): δ [ppm] = 6.94 – 7.04 (m, 1H, 5'-H_{phenyl}), 7.09 – 7.17 (m, 1H, 3'-H_{phenyl}), 7.31 – 7.41 (m, 1H, 4'-H_{phenyl}), 7.57 – 7.65 (m, 1H, 6'-H_{phenyl}), 8.82 (s, 1H, 5-$H_{triazole}$), 9.17 (s br, 1H, CON*HOH*), 10.67 (s br, 1H, O*H*), 11.34 (s br, 1H, CON*HOH*); ^{13}C NMR (DMSO-d$_6$): δ [ppm] = 117.1 (1C, C-3'$_{phenyl}$), 119.5 (1C, C-5'$_{phenyl}$), 124.1 (1C, C-1'$_{phenyl}$), 125.4 (1C, C-6'$_{phenyl}$), 127.4 (1C, C-5$_{triazole}$), 130.6 (1C, C-4'$_{phenyl}$), 140.9 (1C, C-4$_{triazole}$), 149.9 (1C, C-2'$_{phenyl}$), 157.6 (1C, *C*ONHOH); IR (neat): $\tilde{\nu}$ [cm^{-1}] = 3156, 3082, 1639, 1601, 1473, 1393, 1277, 1234, 1177, 1045, 999, 891, 845, 752; HRMS (*m*/*z*): [M+H]$^+$ calcd for $C_9H_9N_4O_3$, 221.0669; found, 221.0668; HPLC (method 2): t_R = 12.4 min, purity 98.8 %.

Methyl 1-[5-chloro-2-(naphthalen-2-ylmethoxy)phenyl]-1*H*-1,2,3-triazole-4-carboxylate (14)

2-(Bromomethyl)naphthalene (260 mg, 1.2 mmol) was added to a stirring suspension of **11** (250 mg, 0.99 mmol) and cesium carbonate (640 mg, 2.0 mmol) in *N*,*N*-dimethylformamide (5 mL). The reaction mixture was heated to 90 °C for 60 min. After cooling to room temperature, water was added and the mixture was extracted with ethyl acetate (3×). The combined organic layers were dried (Na_2SO_4), filtered and the solvent was removed in vacuo. The residue was purified by flash column chromatography (Ø = 3 cm, h = 15 cm, cyclohexane/ethyl acetate = 9/1 → 0/1, V = 15 mL) to give **14** (220 mg, 0.55 mmol, 56%) as colorless solid. m.p. = 186 °C; TLC (cyclohexane/ethyl acetate, 2/1 V/V): R_f = 0.66; ^{1}H NMR (DMSO-d$_6$): δ [ppm] = 3.86 (s, 3H, CO_2CH_3), 5.42 (s, 2H, OCH_2Ar), 7.46 – 7.56 (m, 4H, $H_{arom.}$), 7.64 – 7.68 (m,

1H, $H_{arom.}$), 7.83 – 7.87 (m, 3H, $H_{arom.}$), 7.89 – 7.93 (m, 2H, $H_{arom.}$), 9.23 (s, 1H, 5-$H_{triazole}$); ^{13}C NMR (DMSO-d_6): δ [ppm] = 52.0 (1C, $CO_2\mathit{C}H_3$), 70.8 (1C, O*C*H_2Ar), 116.2 (1C, $C_{arom.}$), 124.7 (1C, $C_{arom.}$), 125.1 (1C, $C_{arom.}$), 125.9 (1C, $C_{arom.}$), 126.0 (1C, $C_{arom.}$), 126.2 (1C, $C_{arom.}$), 126.3 (1C, $C_{arom.}$), 126.4 (1C, $C_{arom.}$), 127.6 (1C, $C_{arom.}$), 127.7 (1C, $C_{arom.}$), 128.1 (1C, $C_{arom.}$), 131.0 (1C, $C_{arom.}$), 131.2 (1C, C-$5_{triazole}$), 132.5 (1C, $C_{arom.}$), 132.6 (1C, $C_{arom.}$), 133.5 (1C, $C_{arom.}$), 138.5 (1C, C-$4_{triazole}$), 150.1 (1C, $C_{arom.}$), 160.5 (1C, $\mathit{C}O_2CH_3$); IR (neat): $\tilde{\nu}$ [cm^{-1}] = 3657, 3183, 2978, 1736, 1528, 1501, 1454, 1435, 1373, 1285, 1250, 1215, 1153, 1130, 1038, 1018, 995, 949, 849, 806, 775, 745; HRMS (*m*/*z*): $[M+H]^+$ calcd for $C_{21}H_{17}ClN_3O_3$, 394.0953; found, 394.0967; HPLC (method 1): t_R = 23.3 min, purity 99.5 %.

1-[5-Chloro-2-(naphthalen-2-ylmethoxy)phenyl]-*N*-hydroxy-1*H*-1,2,3-triazole-4-carboxamide (16)

A 5.4 M solution of sodium methoxide in methanol (0.2 mL, 1.1 mmol) was added to a solution of **14** (110 mg, 0.28 mmol) and hydroxylamine hydrochloride (77 mg, 1.1 mmol) in dry methanol (5 mL). The mixture was stirred at ambient temperature overnight. Then the solvent was removed in vacuo and the residue was purified by automatic flash column chromatography using a Biotage purification apparatus (5% → 100% ACN in H_2O, Biotage® SNAP KP-C18-HS 30 g). Fractions containing the desired product were combined, dried from acetonitrile under reduced pressure and then subjected to lyophilization to give **16** (92 mg, 0.23 mmol, 83%) as colorless solid. m.p. = 161 °C; ^1H NMR (DMSO-d_6): δ [ppm] = 5.44 (s, 2H, OC*H_2*Ar), 7.45 – 7.54 (m, 4H, $H_{arom.}$), 7.58 – 7.61 (m, 1H, $H_{arom.}$), 7.79 – 7.81 (m, 1H, $H_{arom.}$), 7.87 – 7.92 (m, 4H, $H_{arom.}$), 8.73 (s, 1H, 5-$H_{triazole}$); ^{13}C NMR (DMSO-d_6): δ [ppm] = 70.6 (1C, O*C*H_2Ar), 116.2 (1C, $C_{arom.}$), 124.6 (1C, $C_{arom.}$), 125.0 (1C, $C_{arom.}$), 125.5 (1C, $C_{arom.}$), 125.9 (1C,

$C_{arom.}$), 126.1 (1C, $C_{arom.}$), 126.2 (1C, $C_{arom.}$), 126.4 (1C, $C_{arom.}$), 126.8 (1C, $C_{arom.}$), 127.6 (1C, $C_{arom.}$), 127.9 (1C, $C_{arom.}$), 128.2 (1C, $C_{arom.}$), 130.3 (1C, $C_{arom.}$), 132.5 (1C, $C_{arom.}$), 132.7 (1C, $C_{arom.}$), 133.6 (1C, $C_{arom.}$), 143.7 (1C, C-$4_{triazole}$), 149.7 (1C, $C_{arom.}$), 157.7 (1C, *C*ONHOH); IR (neat): $\tilde{\nu}$ [cm^{-1}] = 3306, 3179, 2978, 2886, 1620, 1578, 1504, 1462, 1381, 1281, 1246, 1138, 1030, 991, 957, 845, 810, 760, 667; HRMS (*m*/*z*): $[M+H]^+$ calcd for $C_{20}H_{16}ClN_4O_3$, 395.0905; found, 395.0912; HPLC (method 2): t_R = 16.6 min, purity 94.2 %.

Methyl 1-[5-chloro-2-(*p*-tolyloxy)phenyl]-1*H*-1,2,3-triazole-4-carboxylate (18)

A 25 mL round-bottom flask was charged with **11** (150 mg, 0.59 mmol), $Cu(OAc)_2$ (107 mg, 0.59 mmol), 4-tolylboronic acid (96 mg, 0.71 mmol), and powdered 4 Å molecular sieves. Then dichloromethane (4.5 mL) was added. After the addition of triethylamine (0.41 mL, 2.9 mmol), the reaction mixture was stirred at ambient temperature overnight. Then the suspension was filtered. The filtrate diluted with water and extracted with ethyl acetate (3×). The combined organic layers were dried (Na_2SO_4), filtered and the solvent was removed in vacuo. The residue was purified by flash column chromatography (Ø = 2 cm, h = 15 cm, cyclohexane/ethyl acetate = 9/1 → 3/1, V = 10 mL) to give **18** (22 mg, 0.06 mmol, 11%) as colorless solid. m.p. = 185 °C; TLC (cyclohexane/ethyl acetate, 2/1 V/V): R_f = 0.77; ^1H NMR (DMSO-d_6): δ [ppm] = 2.29 (s, 3H, C*H*3), 3.86 (s, 3H, CO_2CH_3), 6.99 – 7.04 (m, 3H, 2''-H_{tolyl}, 6''-H_{tolyl}, 3'-$H_{5\text{-}chlorophenyl}$), 7.20 – 7.24 (m, 2H, 3''-H_{tolyl}, 5''-H_{tolyl}), 7.59 – 7.63 (m, 1H, 4'-$H_{5\text{-}chlorophenyl}$), 7.95 – 7.97 (m, 1H, 6'-$H_{5\text{-}chlorophenyl}$), 9.22 (s, 1H, 5-$H_{triazole}$); ^{13}C NMR (DMSO-d_6): δ [ppm] = 20.3 (1C, *C*H3), 52.0 (1C, CO_2CH_3), 119.4 (2C, C-2''$_{tolyl}$, C-6''$_{tolyl}$), 119.9 (1C, C-3'$_{5\text{-}chlorophenyl}$), 126.6 (1C, C-6'$_{5\text{-}chlorophenyl}$), 126.9 (1C, $C_{arom.}$), 127.6 (1C, $C_{arom.}$), 130.6 (2C, C-3''$_{tolyl}$, C-5''$_{tolyl}$), 130.9 (1C, C-$5_{triazole}$), 131.4 (1C, C-4'$_{5\text{-}chlorophenyl}$), 134.2 (1C, C-

4''$_{tolyl}$), 138.7 (1C, C-4$_{triazole}$), 149.5 (1C, C-2'$_{5\text{-}chlorophenyl}$), 152.6 (1C, C-1''$_{tolyl}$), 160.4 (1C, *C*O_2CH_3); IR (neat): $\tilde{\nu}$ [cm^{-1}] = 3175, 2924, 1732, 1524, 1508, 1485, 1458, 1435, 1369, 1246, 1215, 1192, 1169, 1150, 1123, 1034, 991, 872, 845, 818, 772, 691; HRMS (*m*/*z*): [M+H]$^+$ calcd for $C_{17}H_{15}ClN_3O_3$, 344.0796; found, 344.0830; HPLC (method 1): t_R = 23.1 min, purity 98.9 %.

1-[5-Chloro-2-(*p*-tolyloxy)phenyl]-*N*-hydroxy-1*H*-1,2,3-triazole-4-carboxamide (20)

A 5.4 M solution of sodium methoxide in methanol (0.2 mL, 1.1 mmol) was added to a solution of **18** (28 mg, 0.08 mmol) and hydroxylamine hydrochloride (28 mg, 0.40 mmol) in dry methanol (3 mL). The mixture was stirred at ambient temperature overnight. Then the solvent was removed in vacuo and the residue was purified by automatic flash column chromatography using a Biotage purification apparatus (5% → 50% ACN in H_2O, Biotage® SNAP KP-C18-HS 12 g). Fractions containing the desired product were combined, dried from acetonitrile under reduced pressure and then subjected to lyophilization to give **20** (28 mg, 0.08 mmol, 99%) as yellowish solid. m.p. = 168 °C (decomposition); ^{1}H NMR (DMSO-d$_6$): δ [ppm] = 2.29 (s, 3H, C*H*$_3$), 6.98 – 7.04 (m, 3H, 2''-H$_{tolyl}$, 6''-H$_{tolyl}$, 3'-H$_{5\text{-}chlorophenyl}$), 7.19 – 7.23 (m, 2H, 3''-H$_{tolyl}$, 5''-H$_{tolyl}$), 7.51 – 7.55 (m, 1H, 4'-H$_{5\text{-}chlorophenyl}$), 7.87 – 7.89 (m, 1H, 6'-H$_{5\text{-}chlorophenyl}$), 8.27 (s, 1H, 5-H$_{triazole}$); ^{13}C NMR (DMSO-d$_6$): δ [ppm] = 20.3 (1C, *C*H$_3$), 119.0 (2C, C-2''$_{tolyl}$, C-6''$_{tolyl}$), 120.6 (1C, C-3'$_{5\text{-}chlorophenyl}$), 123.0 (1C, C-5$_{triazole}$), 125.5 (1C, C-6'$_{5\text{-}chlorophenyl}$), 127.2 (1C, C-5'$_{5\text{-}chlorophenyl}$), 128.9 (1C, C-1'$_{5\text{-}chlorophenyl}$), 130.1 (1C, C-4'$_{5\text{-}chlorophenyl}$), 130.6 (2C, C-3''$_{tolyl}$, C-5''$_{tolyl}$), 133.8 (1C, C-4''$_{tolyl}$), 147.1 (1C, C-4$_{triazole}$), 148.3 (1C, C-2'$_{5\text{-}chlorophenyl}$), 153.6 (1C, C-1''$_{tolyl}$), 158.4 (1C, *C*ONHOH); IR (neat): $\tilde{\nu}$ [cm^{-1}] = 3179, 2978, 2886, 1609, 1493, 1454, 1393, 1238, 1196, 1165, 1126, 1030, 876, 810, 764, 683; HRMS

(*m*/*z*): $[M+H]^+$ calcd for $C_{16}H_{14}ClN_4O_3$, 345.0749; found, 345.0754; HPLC (method 2): t_R = 16.7 min, purity 92.2 %.

1-Azido-2-phenoxybenzene (24)

2-Phenoxyaniline (550 mg, 3.0 mmol) was dissolved in 2 M HCl (11 mL) and the solution was stirred in an ice bath. Then an ice-cold solution of sodium nitrite (302 mg, 4.4 mmol) in water (1.5 mL) was added dropwise over a period of 5 min. After additional 5 min, urea (27 mg) was added to destroy the excess of nitrous acid. Then an ice-cold solution of sodium azide (385 mg, 5.9 mmol) and sodium acetate (1.7 mg, 0.02 mmol) in water (8 mL) was added. The mixture was stirred in an ice bath for 2 h. Then it was extracted with ethyl acetate (3×). The combined organic layers were dried (Na_2SO_4), filtered and the solvent was removed in vacuo. The residue was purified by flash column chromatography (Ø = 4 cm, h = 15 cm, cyclohexane/ethyl acetate = 9.5/0.5, V = 30 mL) to give **24** (520 mg, 2.5 mmol, 83%) as yellowish oil. ^1H NMR (DMSO-d_6): δ [ppm] = 6.92 – 6.95 (m, 2H, 2'-H_{phenyl}, 6'-H_{phenyl}), 7.02 – 7.05 (m, 1H, 3-$H_{1\text{-}azidobenzene}$), 7.10 – 7.14 (m, 1H, 4'-H_{phenyl}), 7.19 – 7.23 (m, 1H, 4-$H_{1\text{-}azidobenzene}$), 7.23 – 7.26 (m, 1H, 5-$H_{1\text{-}azidobenzene}$), 7.27 – 7.29 (m, 1H, 6-$H_{1\text{-}azidobenzene}$), 7.36 – 7.39 (m, 2H, 3'-H_{phenyl}, 5'-H_{phenyl}); ^{13}C NMR (DMSO-d_6): δ [ppm] = 117.0 (2C, C-2'$_{phenyl}$, C-6'$_{phenyl}$), 121.4 (2C, C-$3_{1\text{-}azidobenzene}$, C-$6_{1\text{-}azidobenzene}$), 123.2 (1C, C-4'$_{phenyl}$), 125.5 (1C, C-$5_{1\text{-}azidobenzene}$), 126.4 (1C, C-$4_{1\text{-}azidobenzene}$), 130.0 (1C, C-$1_{1\text{-}azidobenzene}$), 131.0 (2C, C-3'$_{phenyl}$, C-5'$_{phenyl}$), 147.3 (1C, C-$2_{1\text{-}azidobenzene}$), 157.0 (1C, C-1'$_{phenyl}$); IR (neat): $\tilde{\nu}$ [cm^{-1}] = 3063, 2095, 1582, 1485, 1450, 1308, 1296, 1227, 1196, 1161, 1096, 876, 745, 691, 660; HPLC (method 1): t_R = 23.4 min, purity 98.5 %.

Methyl 1-(2-phenoxyphenyl)-1*H*-1,2,3-triazole-4-carboxylate (26)

Methyl propiolate (0.19 mL, 190 mg, 2.2 mmol) was added to a stirring solution of **24** (470 mg, 2.2 mmol) in a 1:1 mixture of water and *tert*-butyl alcohol (10 mL). Then sodium ascorbate (44 mg, 0.22 mmol) and copper(II) sulfate pentahydrate (11 mg, 0.04 mmol) were added and the mixture was stirred overnight at room temperature. Then water was added and the mixture was extracted with ethyl acetate (3×). The combined organic layers were dried (Na_2SO_4), filtered and the solvent was removed in vacuo. The residue was purified by flash column chromatography (Ø = 4 cm, h = 15 cm, cyclohexane/ethyl acetate = 9/1 → 3/1, V = 30 mL) to give **26** (480 mg, 1.6 mmol, 72%) as colorless solid. m.p. = 137 °C; TLC (cyclohexane/ethyl acetate, 2/1 V/V): R_f = 0.60; ^1H NMR (DMSO-d_6): δ [ppm] = 3.85 (s, 3H, CO_2CH_3), 7.06 – 7.09 (m, 3H, 2''-H_{phenyl}, 6''-H_{phenyl}, 3'-$H_{2\text{-phenoxyphenyl}}$), 7.17 – 7.20 (m, 1H, 4''-H_{phenyl}), 7.36 – 7.42 (m, 3H, 3''-H_{phenyl}, 5''-H_{phenyl}, 5'-$H_{2\text{-phenoxyphenyl}}$), 7.56 – 7.60 (m, 1H, 4'-$H_{2\text{-phenoxyphenyl}}$), 7.79 – 7.80 (m, 1H, 6'-$H_{2\text{-phenoxyphenyl}}$), 9.19 (s, 1H, 5-$H_{triazole}$); ^{13}C NMR (DMSO-d_6): δ [ppm] = 52.0 (1C, CO_2CH_3), 119.0 (1C, C-3'$_{2\text{-phenoxyphenyl}}$), 119.1 (2C, C-2''$_{phenyl}$, C-6''$_{phenyl}$), 124.1 (1C, C-5'$_{2\text{-phenoxyphenyl}}$), 124.6 (1C, C-4''$_{phenyl}$), 127.0 (1C, C-6'$_{2\text{-phenoxyphenyl}}$), 127.2 (1C, C-1'$_{2\text{-phenoxyphenyl}}$), 130.2 (2C, C-3''$_{phenyl}$, C-5''$_{phenyl}$), 130.8 (1C, C-5$_{triazole}$), 131.7 (1C, C-4'$_{2\text{-phenoxyphenyl}}$), 138.7 (1C, C-4$_{triazole}$), 149.8 (1C, C-2'$_{2\text{-phenoxyphenyl}}$), 155.3 (1C, C-1''$_{phenyl}$), 160.5 (1C, CO_2CH_3); IR (neat): $\tilde{\nu}$ [cm^{-1}] = 2978, 2889, 1740, 1585, 1528, 1493, 1470, 1431, 1369, 1242, 1211, 1146, 1034, 945, 868, 849, 799, 752, 694; HRMS (*m*/*z*): $[M+H]^+$ calcd for $C_{16}H_{14}N_3O_3$, 296.1030; found, 296.1038; HPLC (method 1): t_R = 21.0 min, purity 99.6 %.

***N*-Hydroxy-1-(2-phenoxyphenyl)-1*H*-1,2,3-triazole-4-carboxamide (28)**

A 5.4 M solution of sodium methoxide in methanol (0.6 mL, 3.2 mmol) was added to a solution of **26** (270 mg, 0.92 mmol) and hydroxylamine hydrochloride (321 mg, 4.6 mmol) in dry methanol (13 mL). The mixture was stirred at ambient temperature overnight. Then the solvent was removed in vacuo and the residue was purified by automatic flash column chromatography using a Biotage purification apparatus (5% → 80% ACN in H_2O, Biotage® SNAP KP-C18-HS 30 g). Fractions containing the desired product were combined, dried from acetonitrile under reduced pressure and then subjected to lyophilization to give **28** (140 mg, 0.48 mmol, 52%) as colorless solid. m.p. = 168-170 °C; ^{1}H NMR (DMSO-d_6): δ [ppm] = 7.05 – 7.10 (m, 3H, 2''-H_{phenyl}, 6''-H_{phenyl}, 3'-$H_{2\text{-phenoxyphenyl}}$), 7.16 – 7.20 (m, 1H, 4''-H_{phenyl}), 7.35 – 7.42 (m, 3H, 3''-H_{phenyl}, 5''-H_{phenyl}, 5'-$H_{2\text{-phenoxyphenyl}}$), 7.54 – 7.59 (m, 1H, 4'-$H_{2\text{-phenoxyphenyl}}$), 7.79 – 7.82 (m, 1H, 6'-$H_{2\text{-phenoxyphenyl}}$), 8.92 (s, 1H, 5-$H_{triazole}$); ^{13}C NMR (DMSO-$d_6$): δ [ppm] = 119.0 (2C, C-2''$_{phenyl}$, C-6''$_{phenyl}$), 119.2 (1C, C-3'$_{2\text{-phenoxyphenyl}}$), 124.2 (1C, C-5'$_{2\text{-phenoxyphenyl}}$), 124.5 (1C, C-4''$_{phenyl}$), 126.7 (1C, C-6'$_{2\text{-phenoxyphenyl}}$), 127.5 (1C, C-1'$_{2\text{-phenoxyphenyl}}$), 127.7 (1C, C-5$_{triazole}$), 130.2 (2C, C-3''$_{phenyl}$, C-5''$_{phenyl}$), 131.5 (1C, C-4'$_{2\text{-phenoxyphenyl}}$), 141.3 (1C, C-4$_{triazole}$), 149.5 (1C, C-2'$_{2\text{-phenoxyphenyl}}$), 155.4 (1C, C-1''$_{phenyl}$), 157.2 (1C, *C*ONHOH); IR (neat): $\tilde{\nu}$ [cm^{-1}] =3260, 3136, 2978, 2889, 1647, 1562, 1466, 1234, 1184, 1161, 1123, 1026, 984, 864, 802, 748, 694, 648; HRMS (*m*/*z*): $[M+H]^+$ calcd for $C_{15}H_{13}N_4O_3$, 297.0982; found, 297.0983; HPLC (method 2): t_R = 15.4 min, purity 99.6 %.

^{1}H and ^{13}C NMR spectra of representative compounds

Compound **5a**

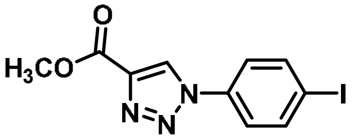

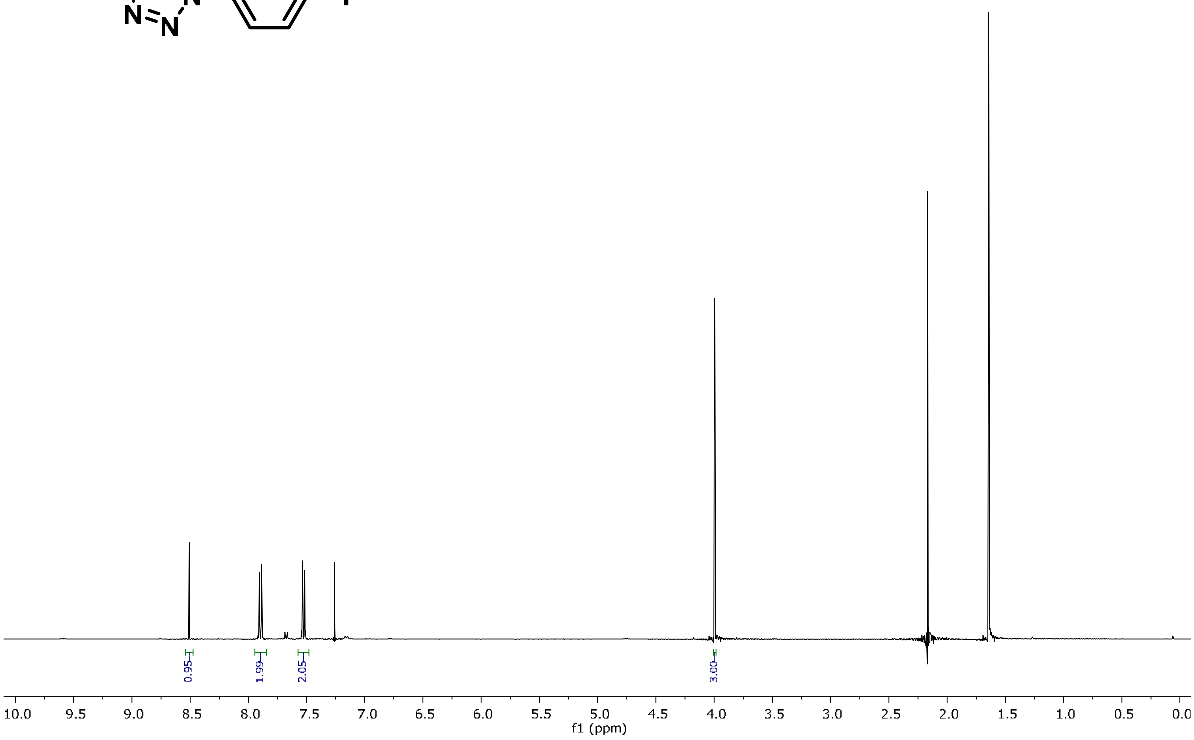

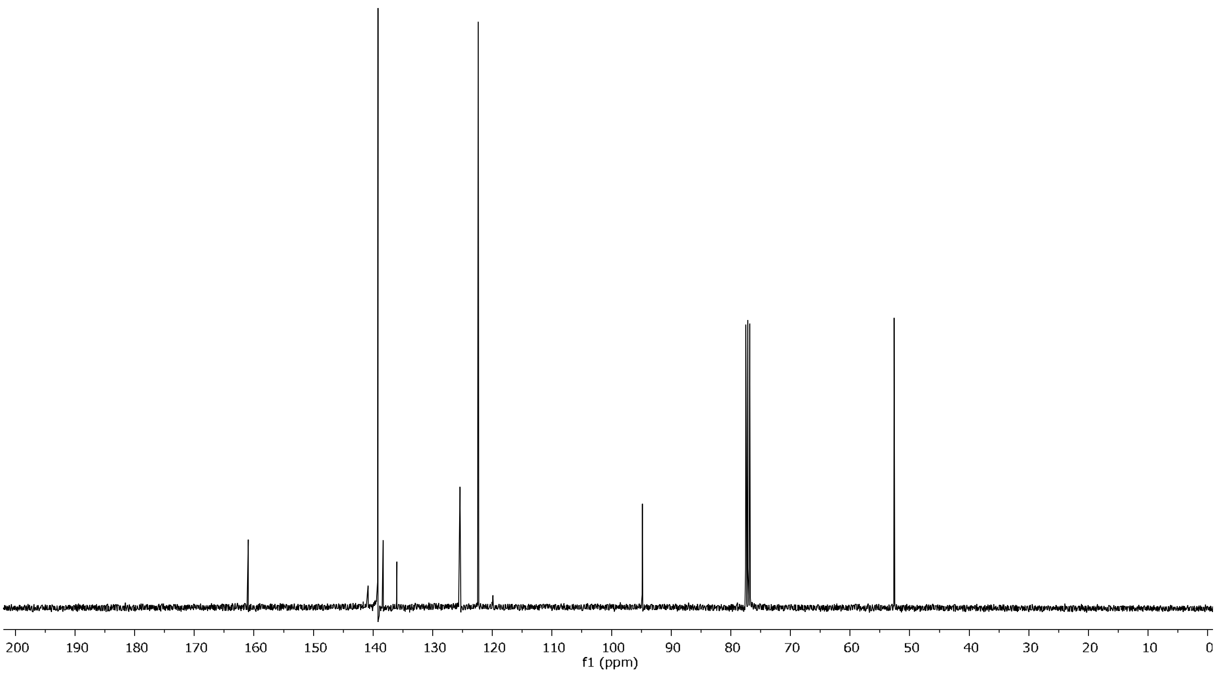

Compound **5b**

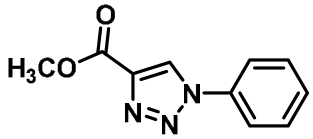

O
H_3CO
N
N
N

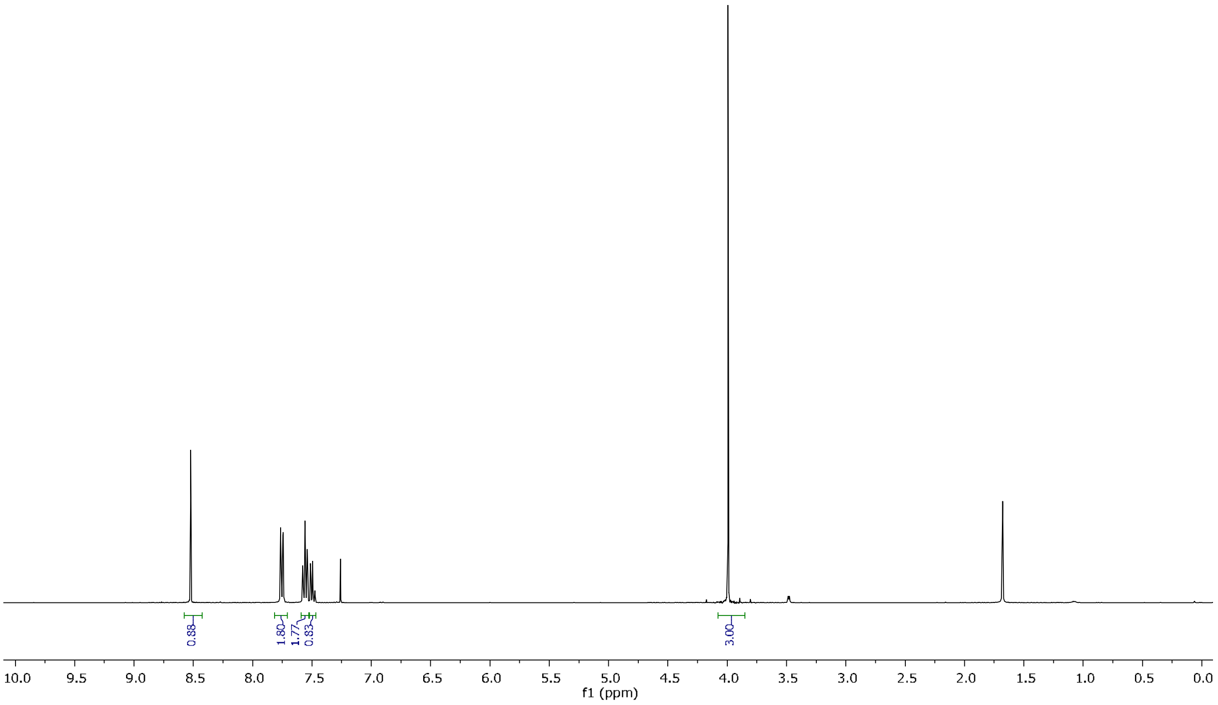

0.88
1.80
1.77
0.83
3.00
10.0
9.5
9.0
8.5
8.0
7.5
7.0
6.5
6.0
5.5
5.0
4.5
4.0
3.5
3.0
2.5
2.0
1.5
1.0
0.5
0.0
f1 (ppm)

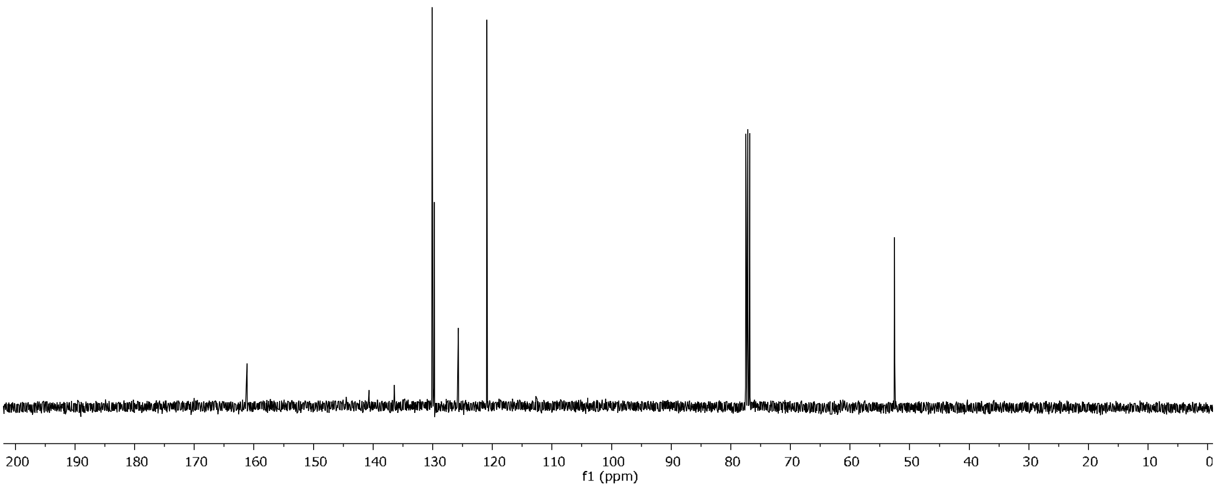

200
190
180
170
160
150
140
130
120
110
100
90
80
70
60
50
40
30
20
10
0
f1 (ppm)

Compound **5c**

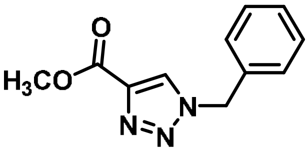
O
H3CO
N
N=N

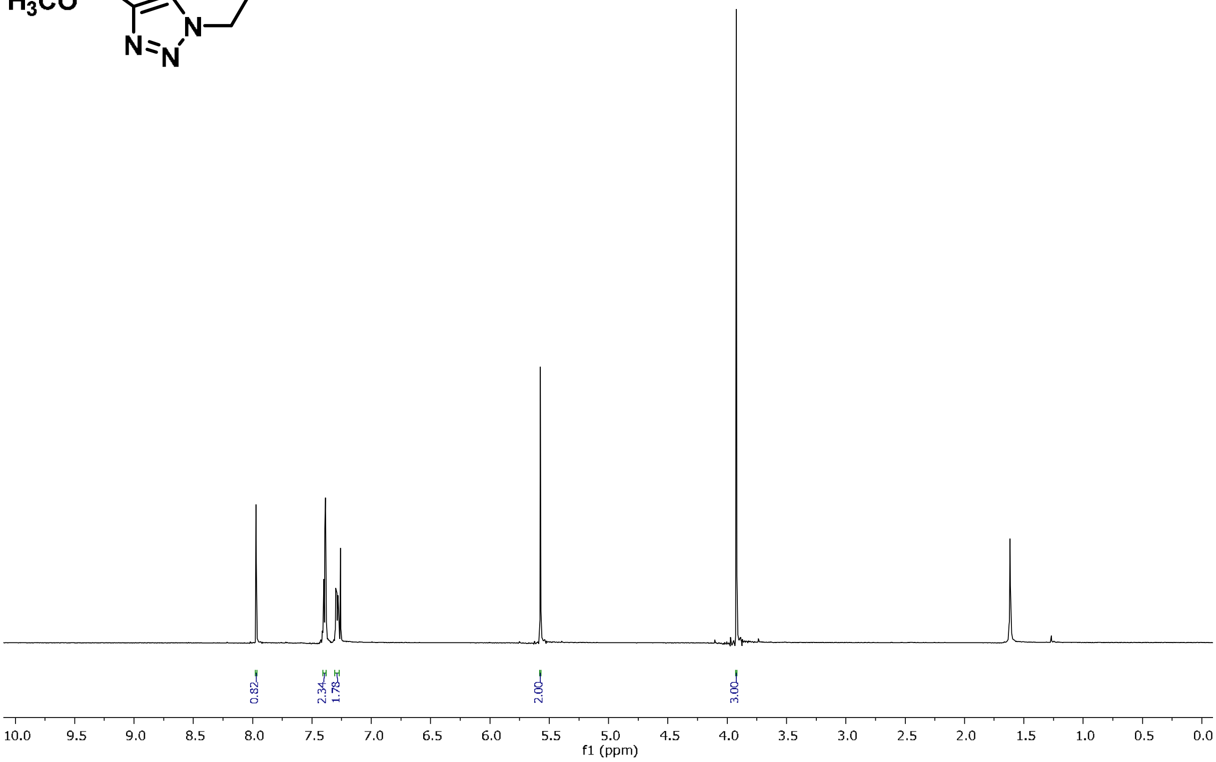
0.82
2.34
1.78
2.00
3.00
10.0 9.5 9.0 8.5 8.0 7.5 7.0 6.5 6.0 5.5 5.0 4.5 4.0 3.5 3.0 2.5 2.0 1.5 1.0 0.5 0.0
f1 (ppm)

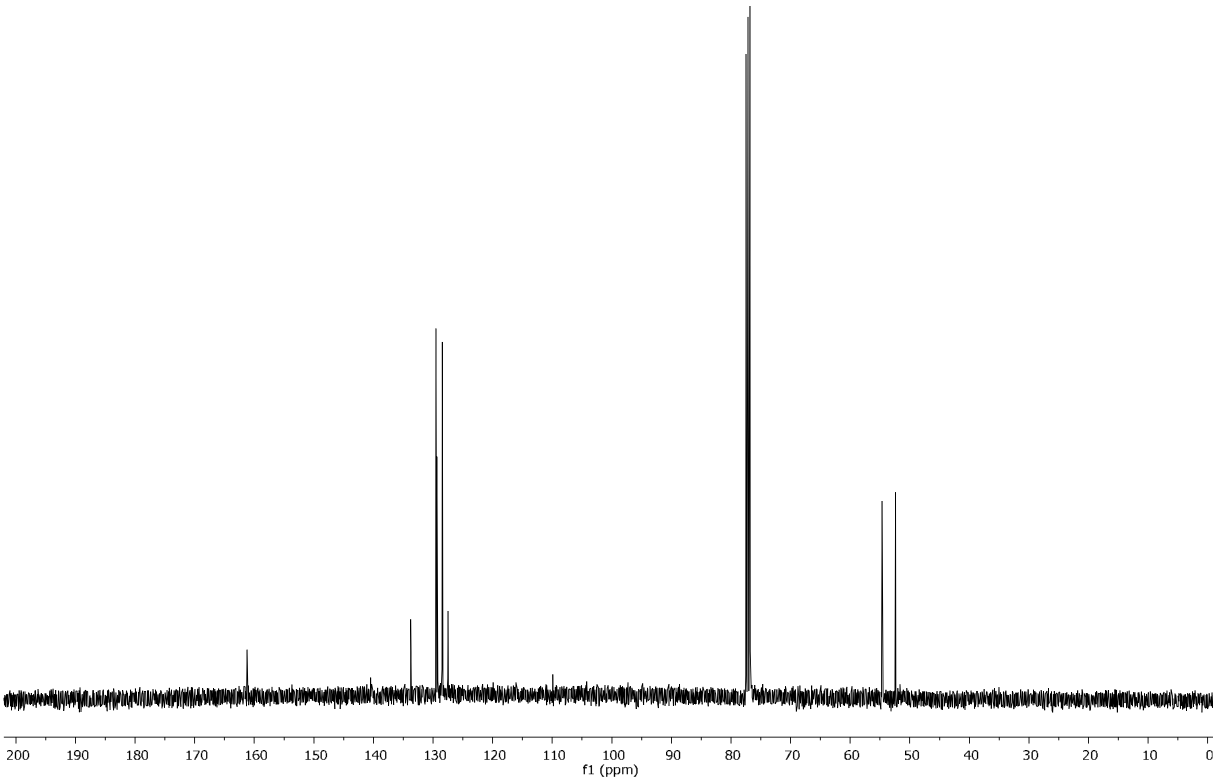
200 190 180 170 160 150 140 130 120 110 100 90 80 70 60 50 40 30 20 10 0
f1 (ppm)

Compound **5d**

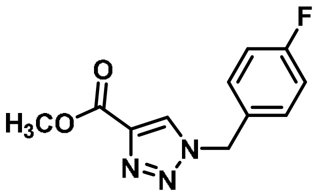

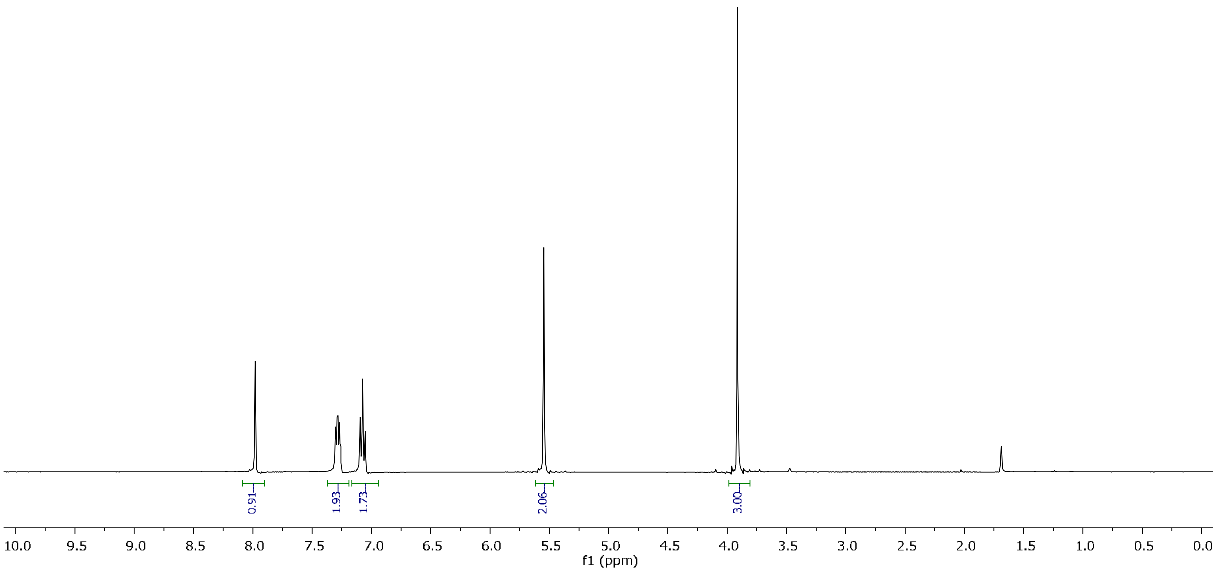
0.91
1.93
1.73
2.06
3.00
10.0 9.5 9.0 8.5 8.0 7.5 7.0 6.5 6.0 5.5 5.0 4.5 4.0 3.5 3.0 2.5 2.0 1.5 1.0 0.5 0.0
f1 (ppm)

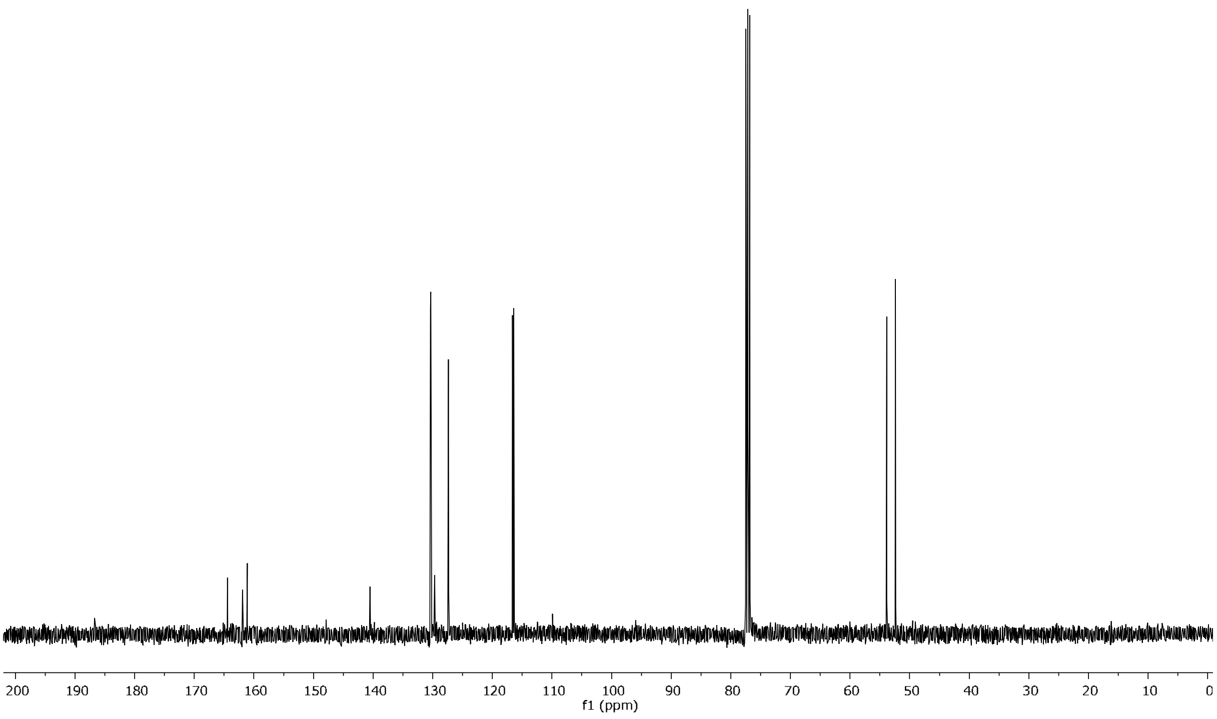
200 190 180 170 160 150 140 130 120 110 100 90 80 70 60 50 40 30 20 10 0
f1 (ppm)

Compound **5e**

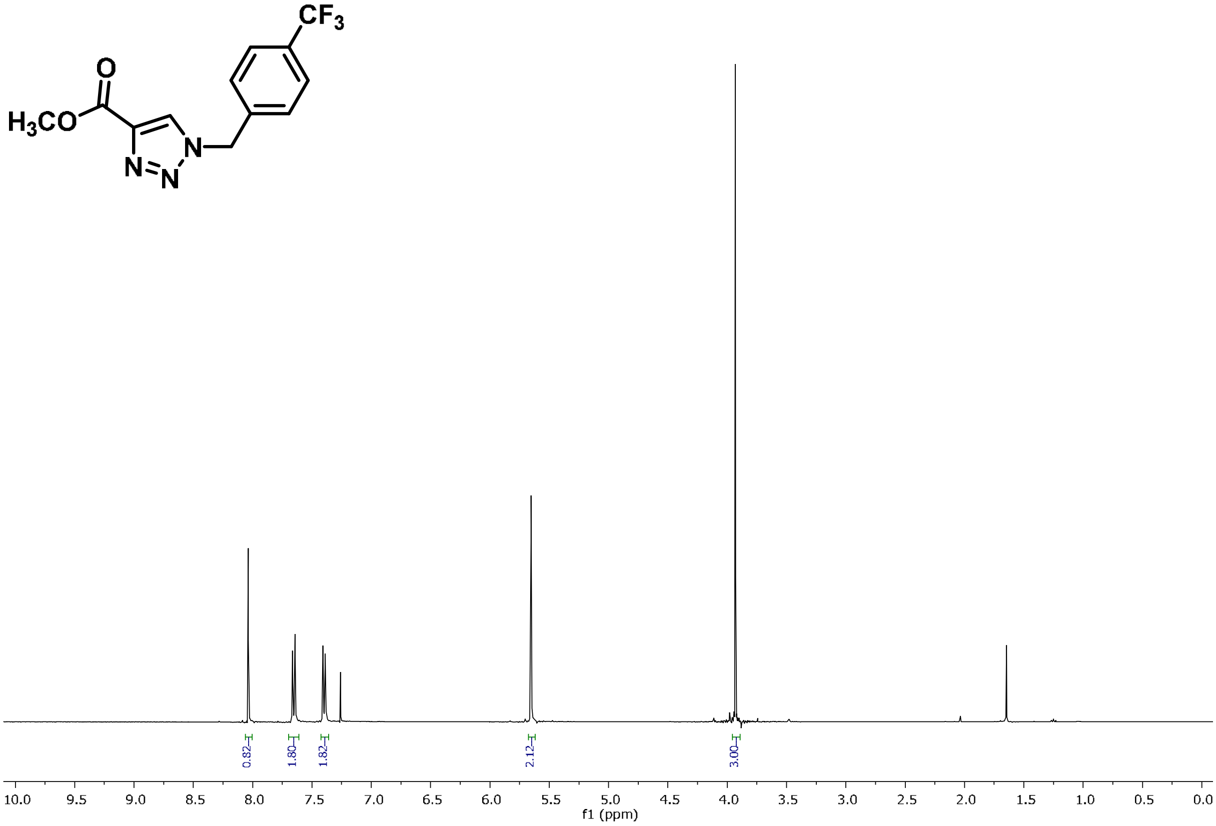
CF3
O
H3CO
N
N
N
0.82
1.80
1.82
2.12
3.00
10.0 9.5 9.0 8.5 8.0 7.5 7.0 6.5 6.0 5.5 5.0 4.5 4.0 3.5 3.0 2.5 2.0 1.5 1.0 0.5 0.0
f1 (ppm)

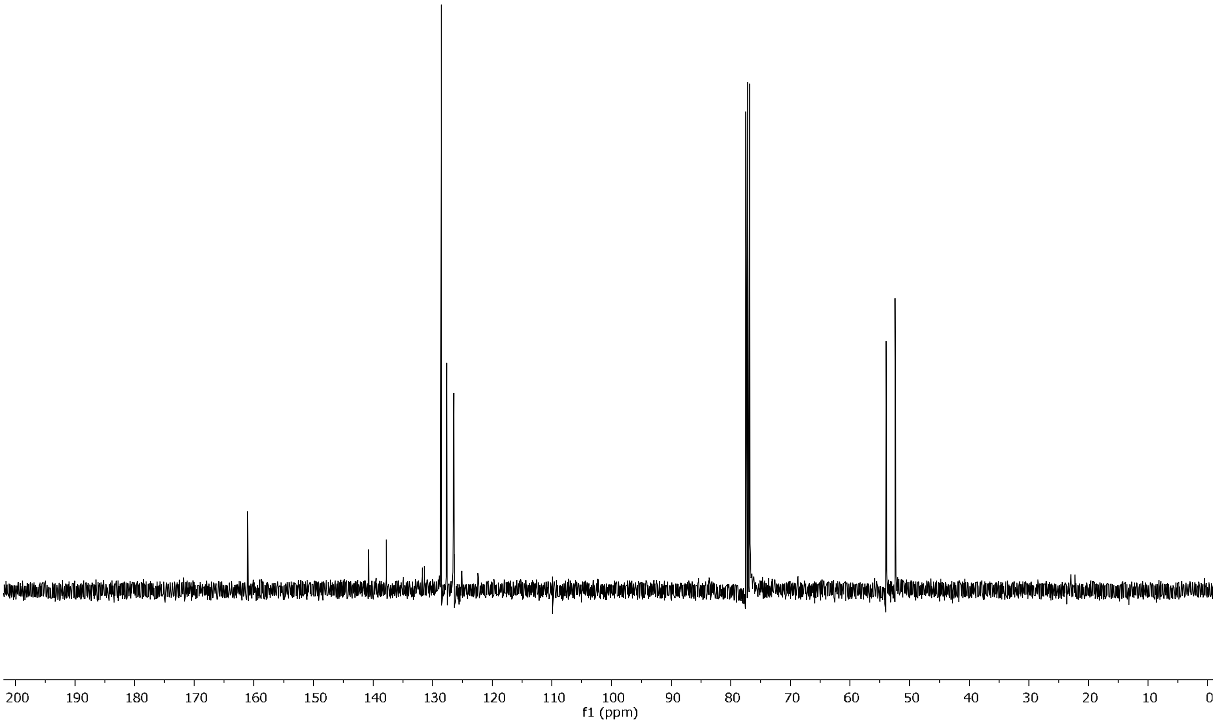
200 190 180 170 160 150 140 130 120 110 100 90 80 70 60 50 40 30 20 10 0
f1 (ppm)

Compound **5f**

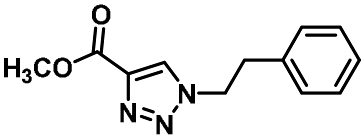
O
H3CO
N
N
N

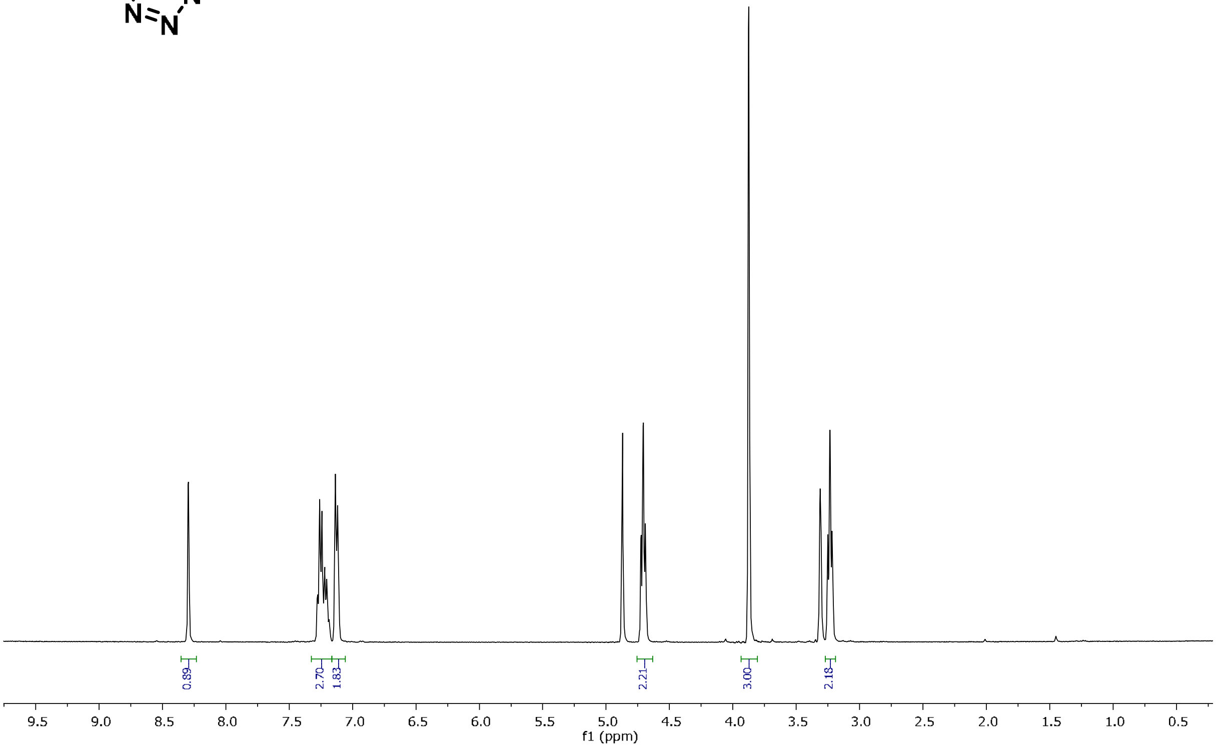
0.89
2.70
1.83
2.21
3.00
2.18
9.5 9.0 8.5 8.0 7.5 7.0 6.5 6.0 5.5 5.0 4.5 4.0 3.5 3.0 2.5 2.0 1.5 1.0 0.5
f1 (ppm)

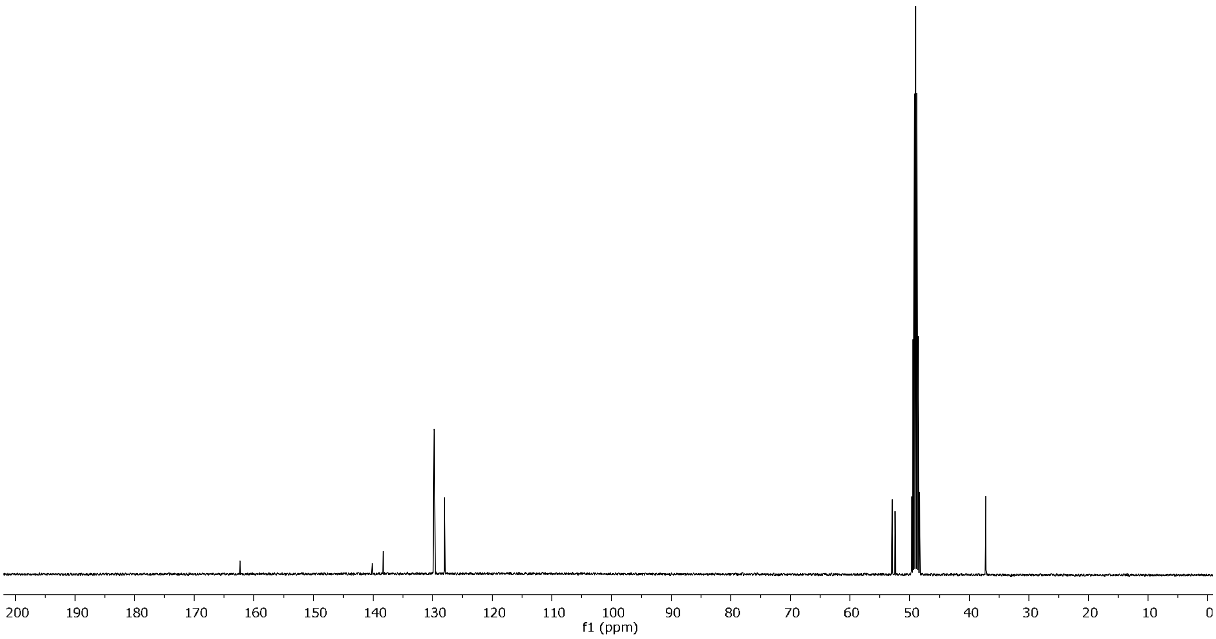
200 190 180 170 160 150 140 130 120 110 100 90 80 70 60 50 40 30 20 10 0
f1 (ppm)

Compound **5g**

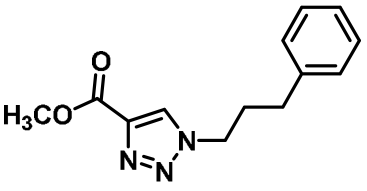

O
H_3CO
N
N
N

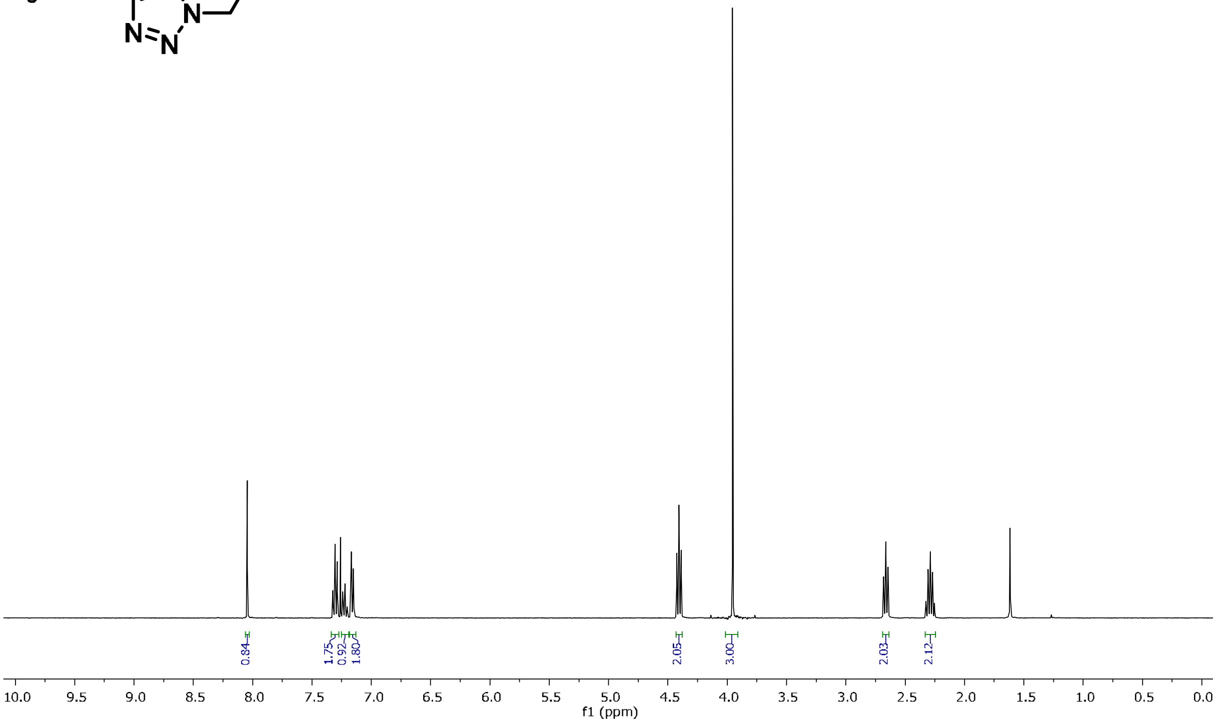

0.84
1.75
0.92
1.80
2.05
3.00
2.03
2.12
10.0 9.5 9.0 8.5 8.0 7.5 7.0 6.5 6.0 5.5 5.0 4.5 4.0 3.5 3.0 2.5 2.0 1.5 1.0 0.5 0.0
f1 (ppm)

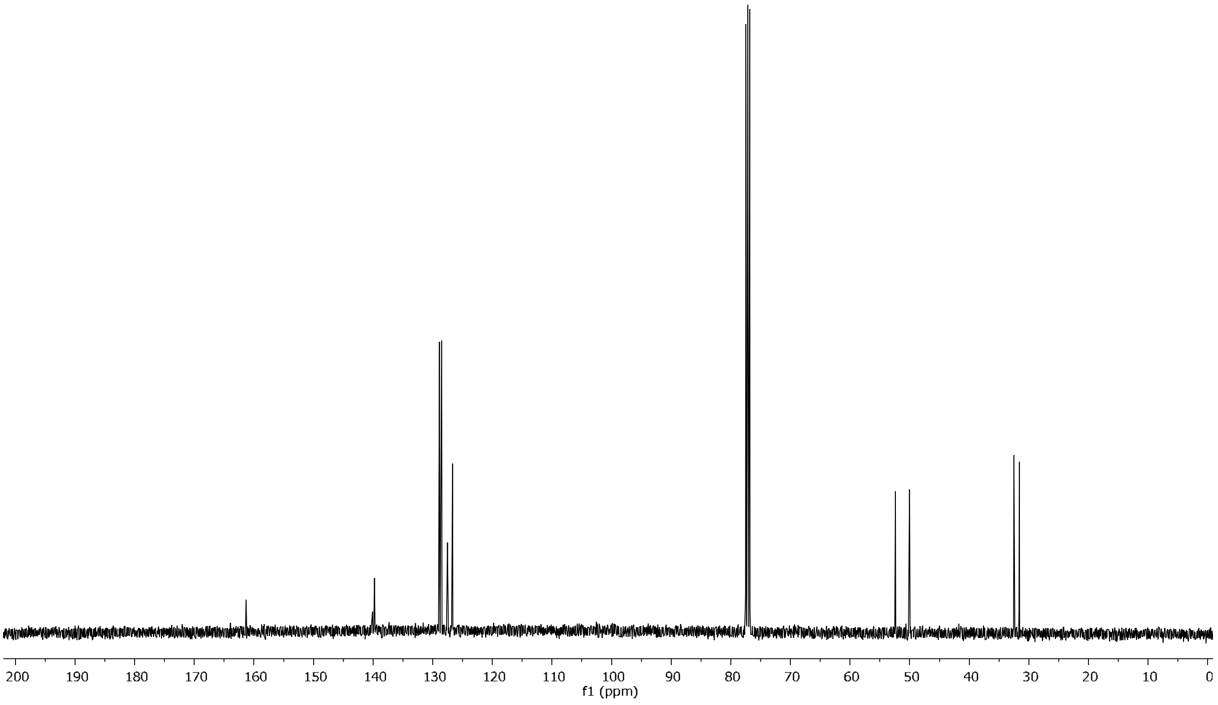

200 190 180 170 160 150 140 130 120 110 100 90 80 70 60 50 40 30 20 10 0
f1 (ppm)

Compound **5h**

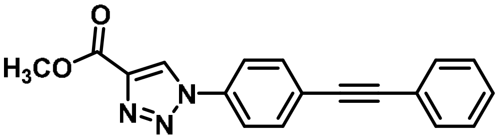
O
H3CO
N
N
N

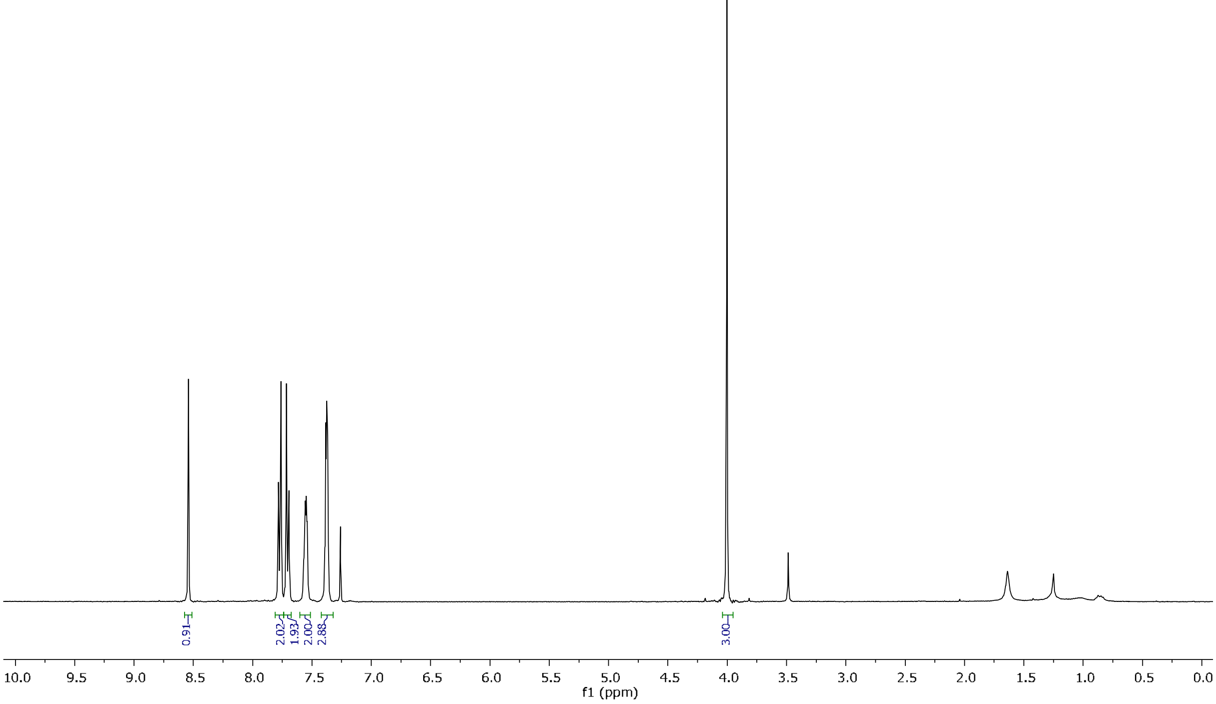
0.91
2.02
1.93
2.00
2.88
3.00
10.0
9.5
9.0
8.5
8.0
7.5
7.0
6.5
6.0
5.5
5.0
4.5
4.0
3.5
3.0
2.5
2.0
1.5
1.0
0.5
0.0
f1 (ppm)

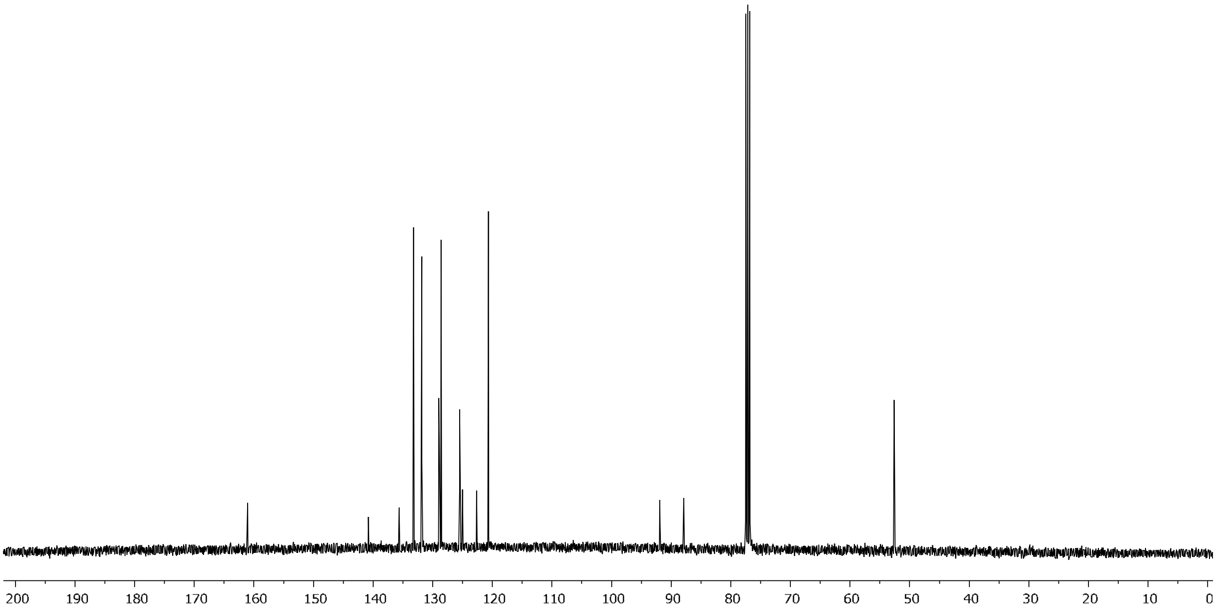
200
190
180
170
160
150
140
130
120
110
100
90
80
70
60
50
40
30
20
10
0
f1 (ppm)

Compound **5i**

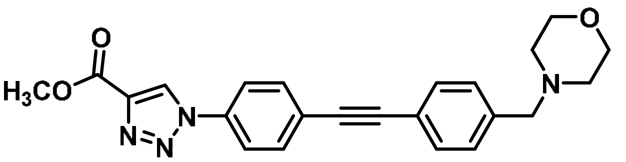

O
H3CO
N
N
N
O
N

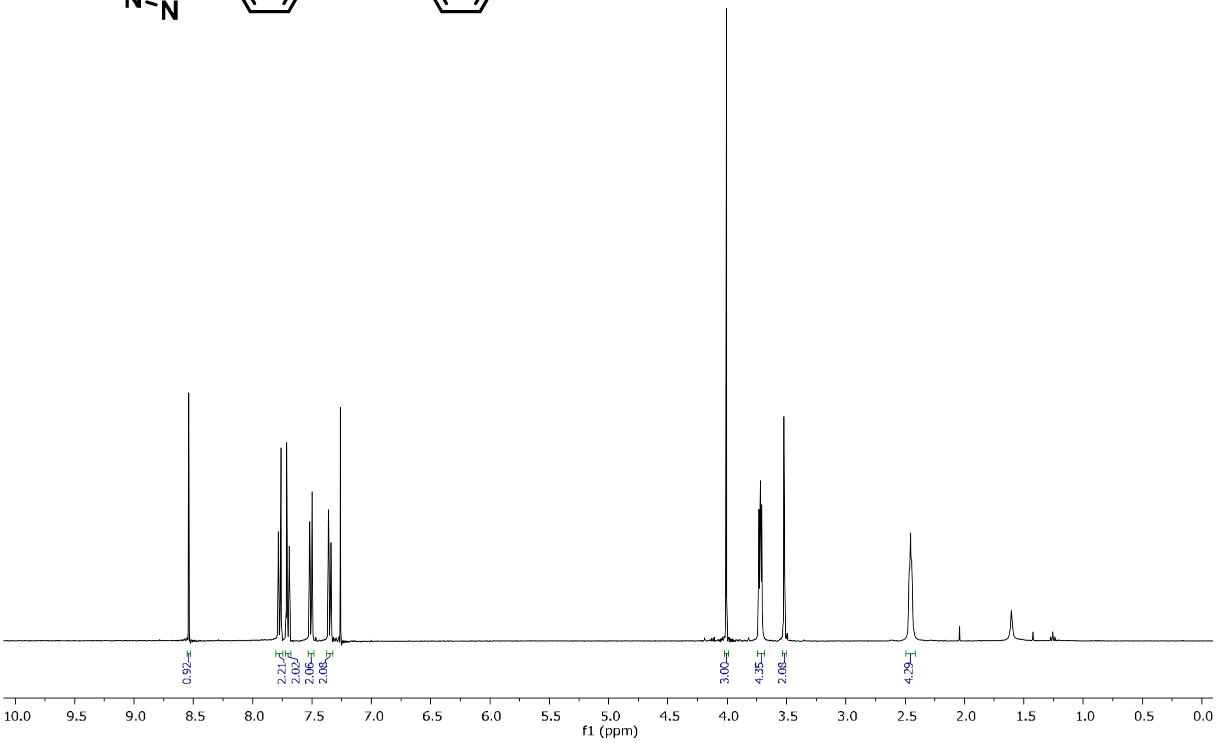

0.92
2.21
2.02
2.06
2.08
3.00
4.35
2.08
4.29
10.0 9.5 9.0 8.5 8.0 7.5 7.0 6.5 6.0 5.5 5.0 4.5 4.0 3.5 3.0 2.5 2.0 1.5 1.0 0.5 0.0
f1 (ppm)

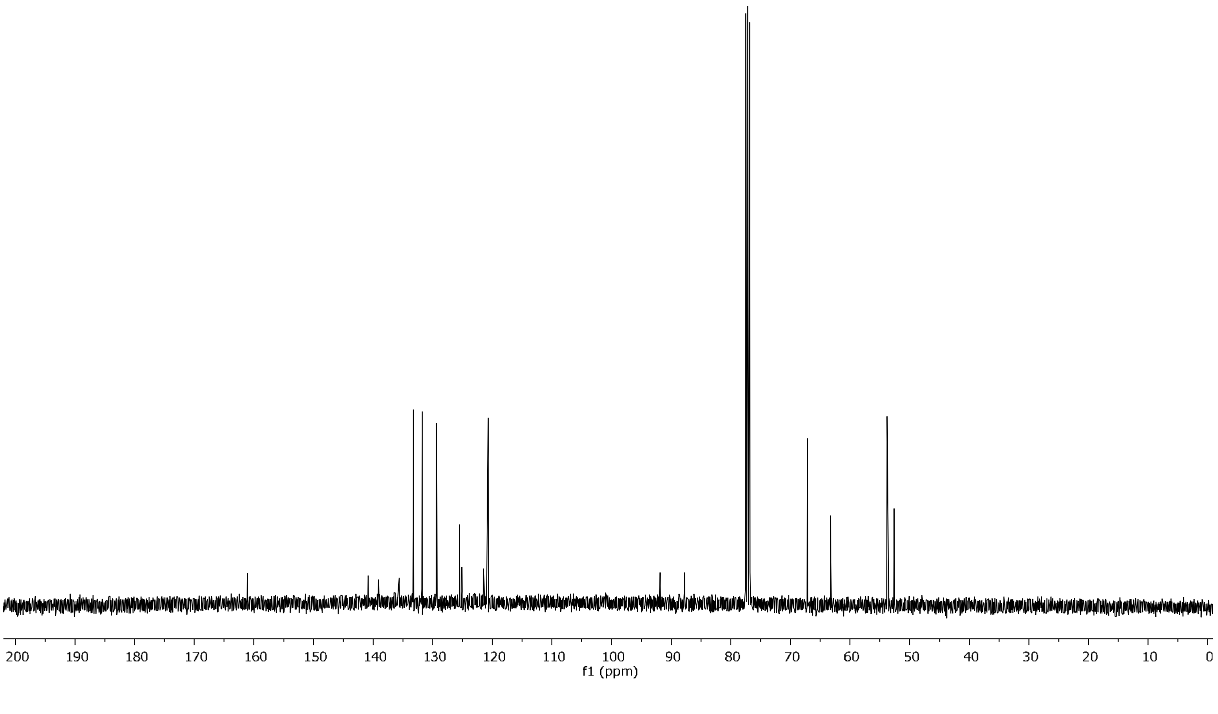

200 190 180 170 160 150 140 130 120 110 100 90 80 70 60 50 40 30 20 10 0
f1 (ppm)

Compound **7**

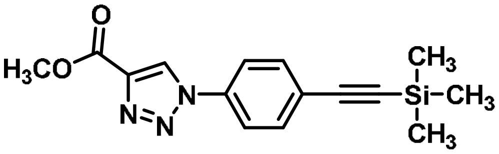
O
H3CO
N
N=N
CH3
Si–CH3
CH3

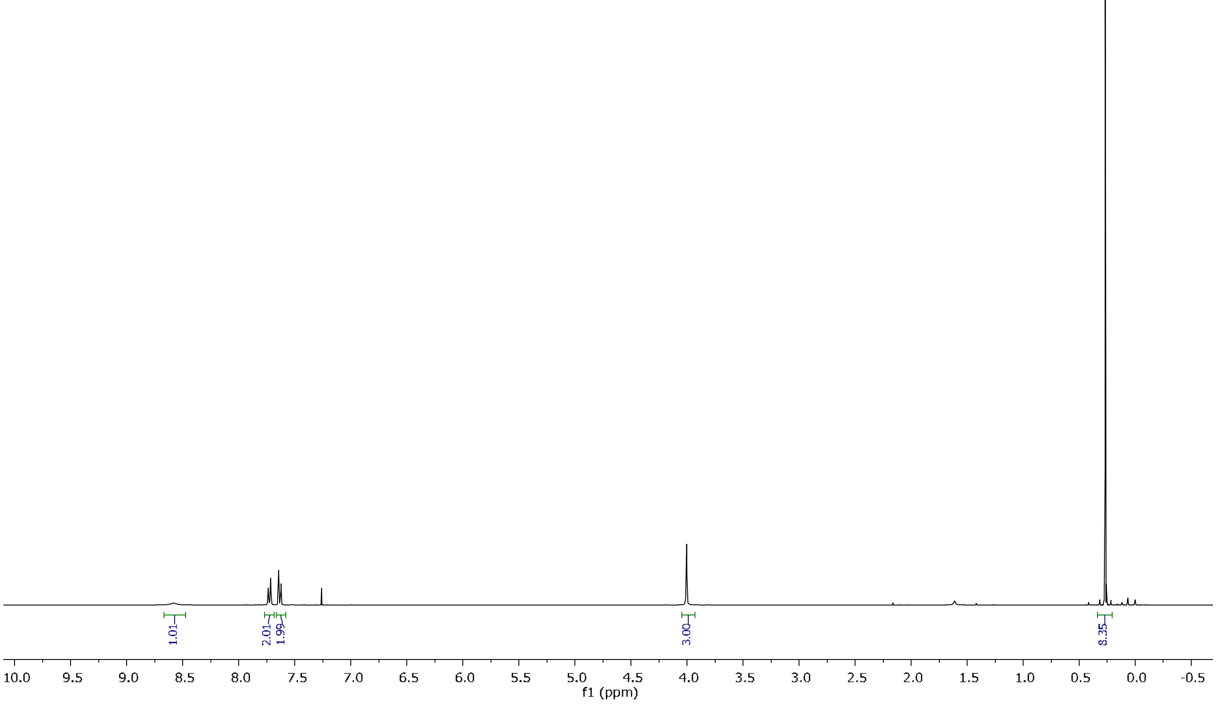
1.01
2.01
1.99
3.00
8.35
10.0 9.5 9.0 8.5 8.0 7.5 7.0 6.5 6.0 5.5 5.0 4.5 4.0 3.5 3.0 2.5 2.0 1.5 1.0 0.5 0.0 -0.5
f1 (ppm)

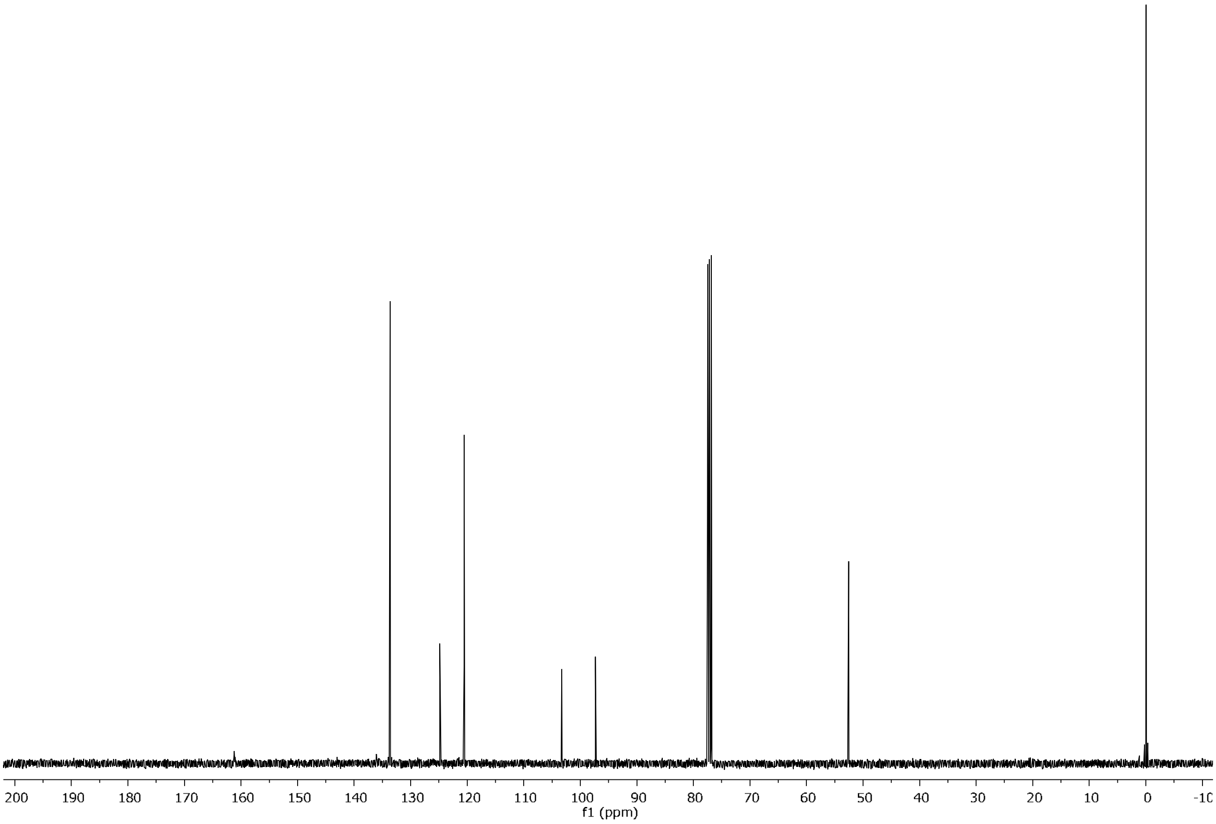
200 190 180 170 160 150 140 130 120 110 100 90 80 70 60 50 40 30 20 10 0 -10
f1 (ppm)

Compound **5j**

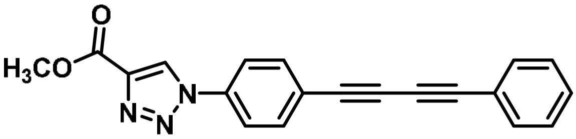

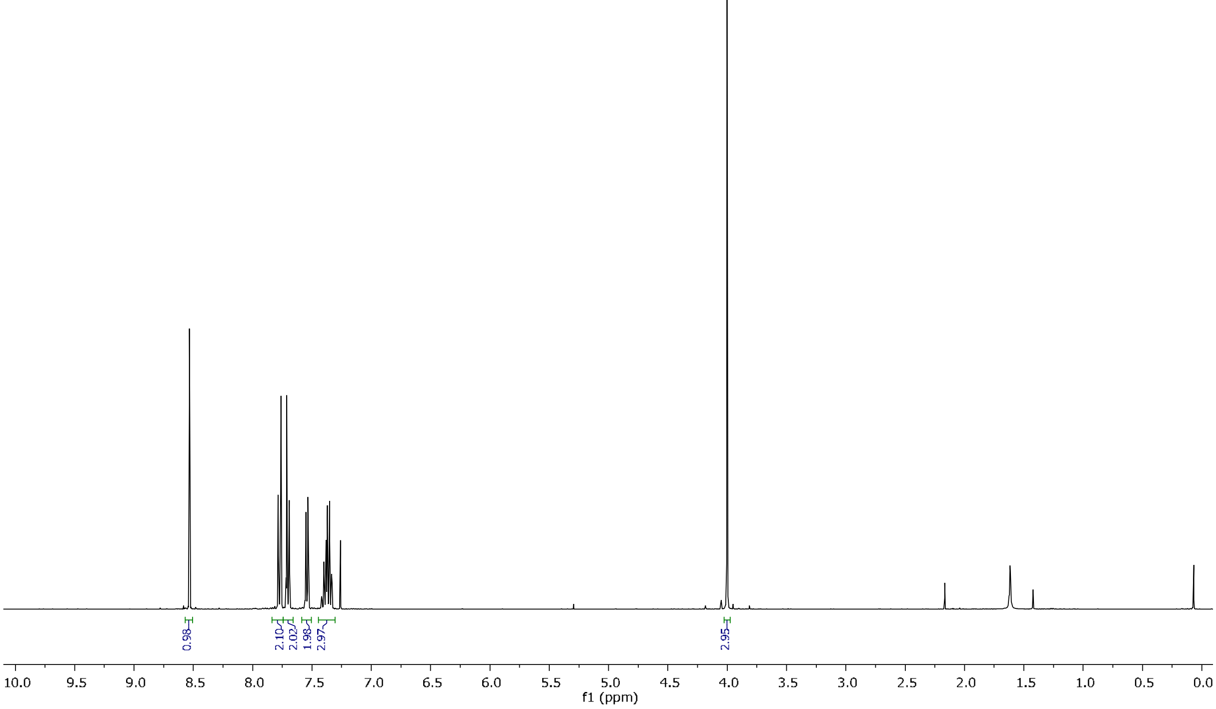
0.98
2.10
2.02
1.98
2.97
2.95
10.0 9.5 9.0 8.5 8.0 7.5 7.0 6.5 6.0 5.5 5.0 4.5 4.0 3.5 3.0 2.5 2.0 1.5 1.0 0.5 0.0
f1 (ppm)

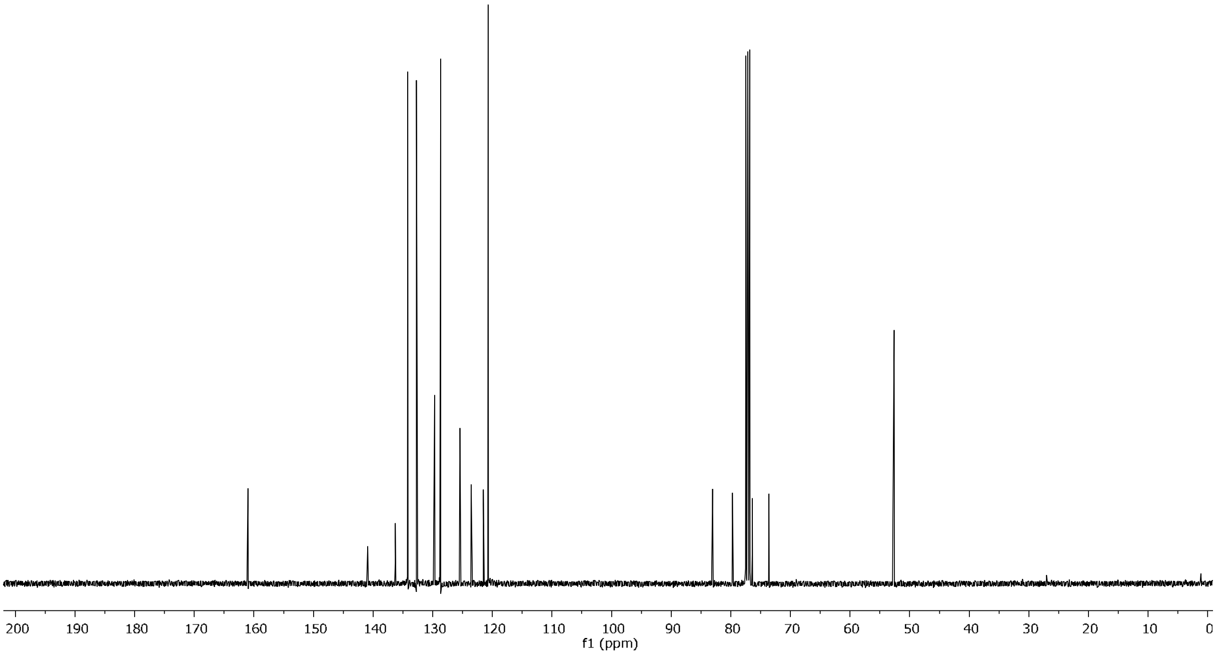
200 190 180 170 160 150 140 130 120 110 100 90 80 70 60 50 40 30 20 10 0
f1 (ppm)

Compound **2b**

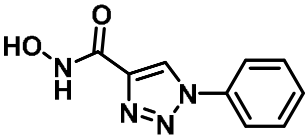

HO
N
H
O
N
N
N

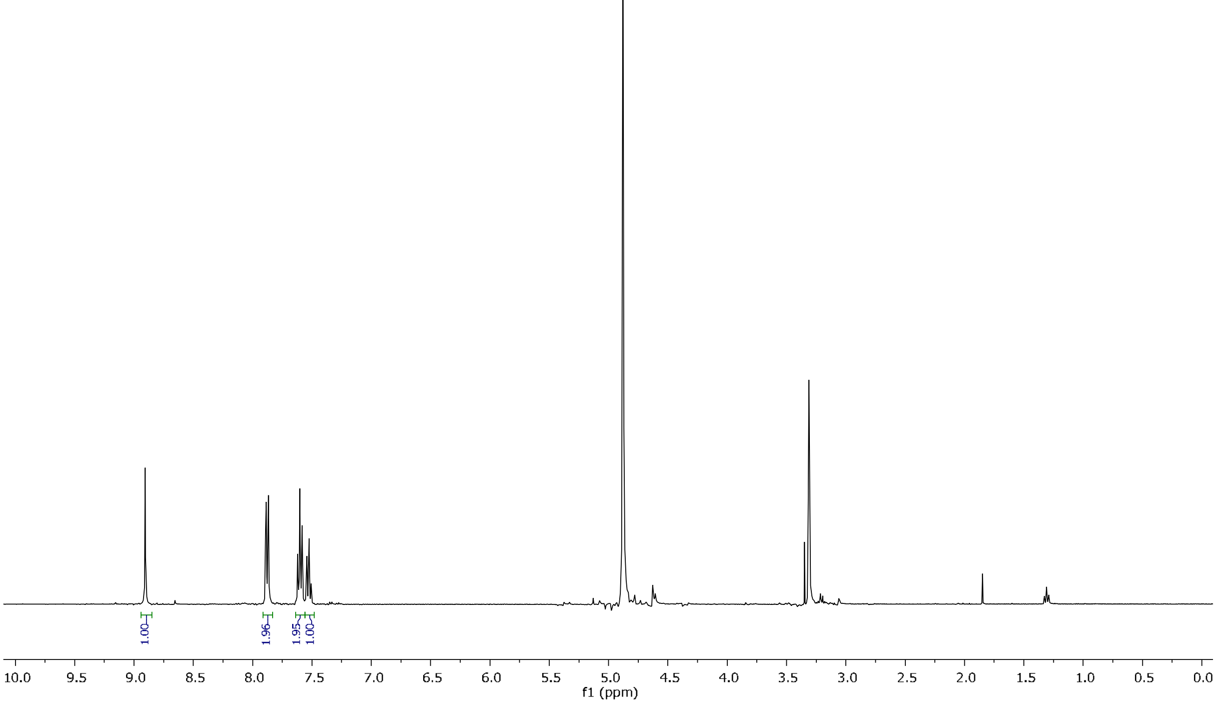

1.00
1.96
1.95
1.00
10.0 9.5 9.0 8.5 8.0 7.5 7.0 6.5 6.0 5.5 5.0 4.5 4.0 3.5 3.0 2.5 2.0 1.5 1.0 0.5 0.0
f1 (ppm)

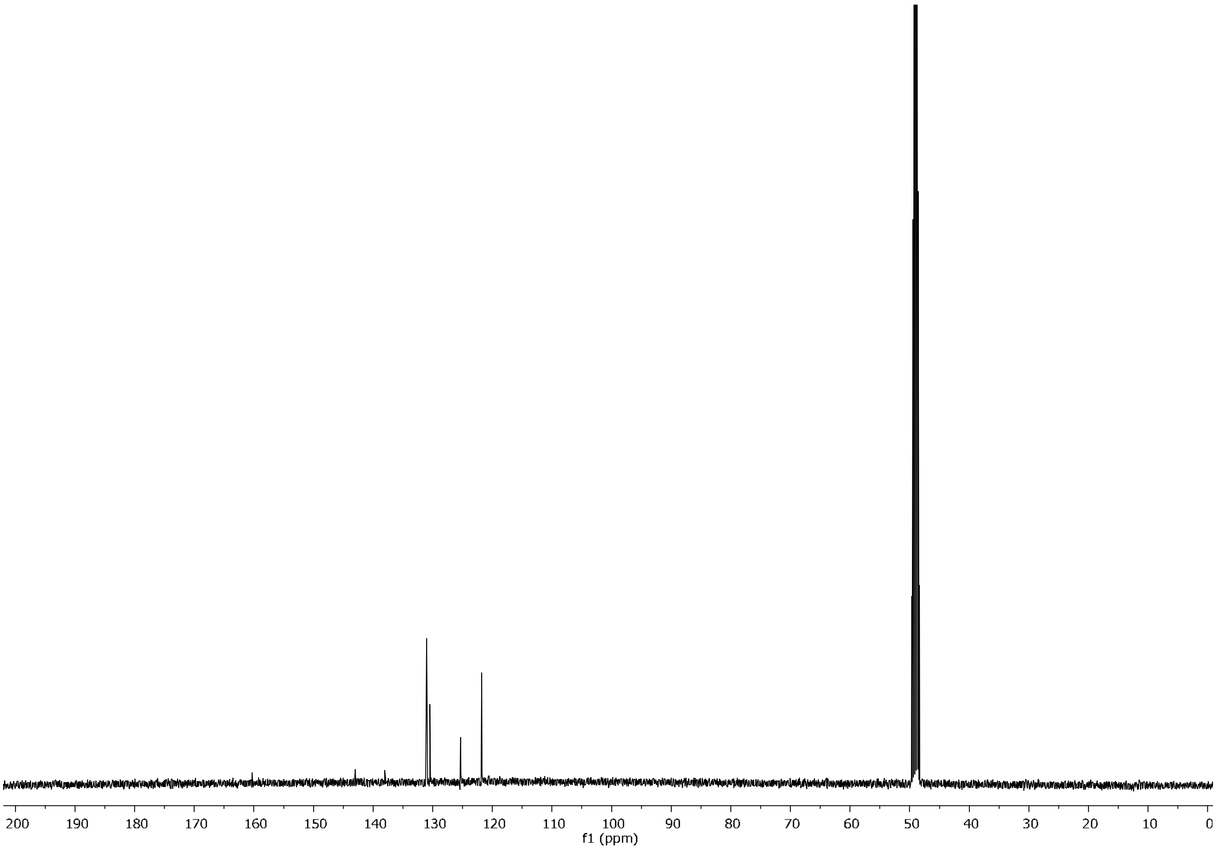

200 190 180 170 160 150 140 130 120 110 100 90 80 70 60 50 40 30 20 10 0
f1 (ppm)

Compound **2c**

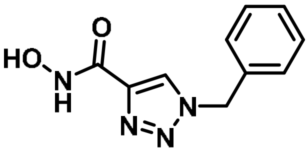
HO
N
H
O
N
N
N

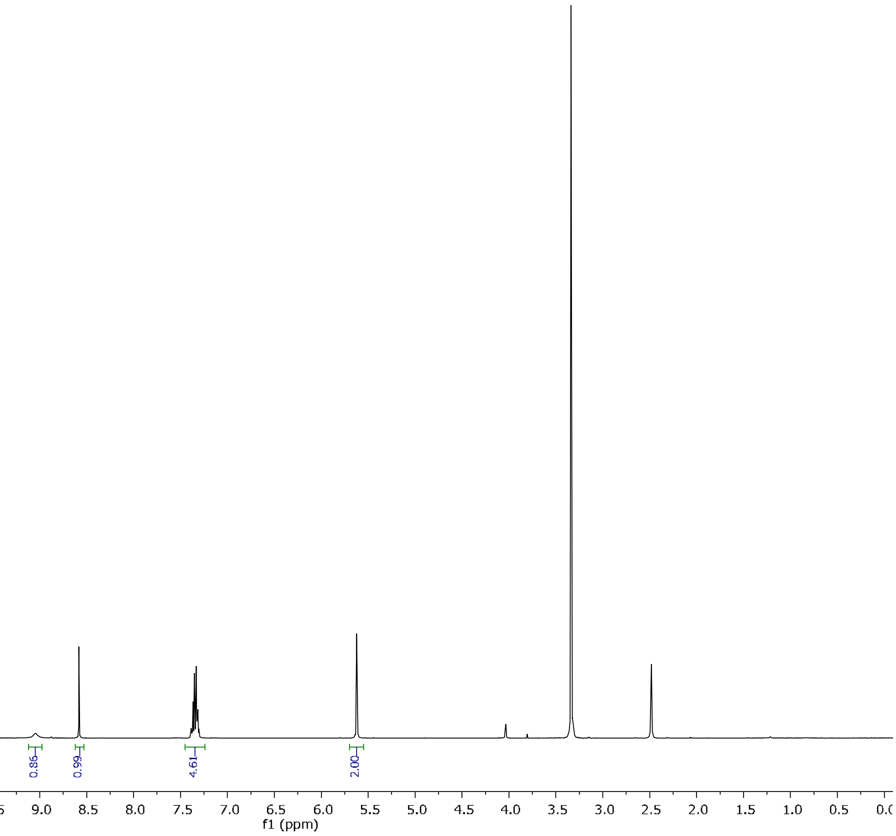
0.97
0.86
0.99
4.61
2.00
12.5
12.0
11.5
11.0
10.5
10.0
9.5
9.0
8.5
8.0
7.5
7.0
6.5
6.0
5.5
5.0
4.5
4.0
3.5
3.0
2.5
2.0
1.5
1.0
0.5
f1 (ppm)

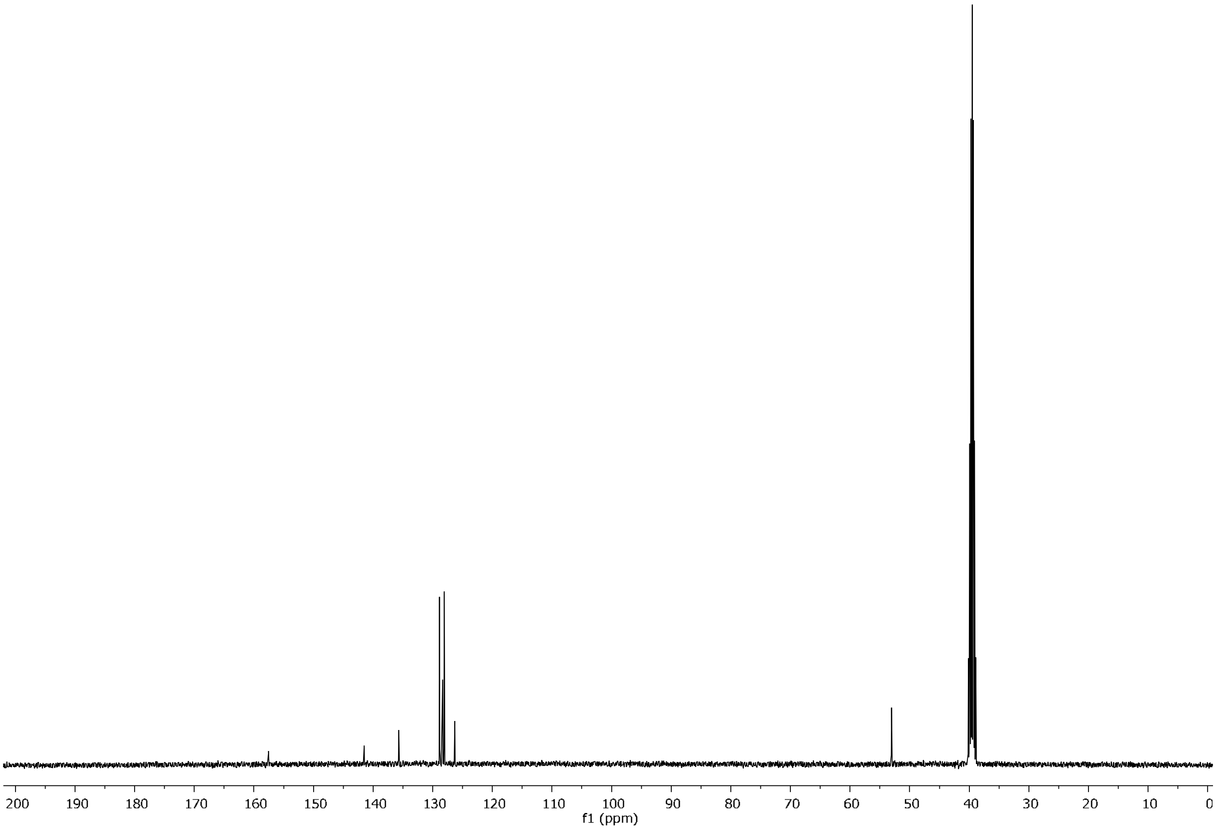
200
190
180
170
160
150
140
130
120
110
100
90
80
70
60
50
40
30
20
10
0
f1 (ppm)

Compound **2d**

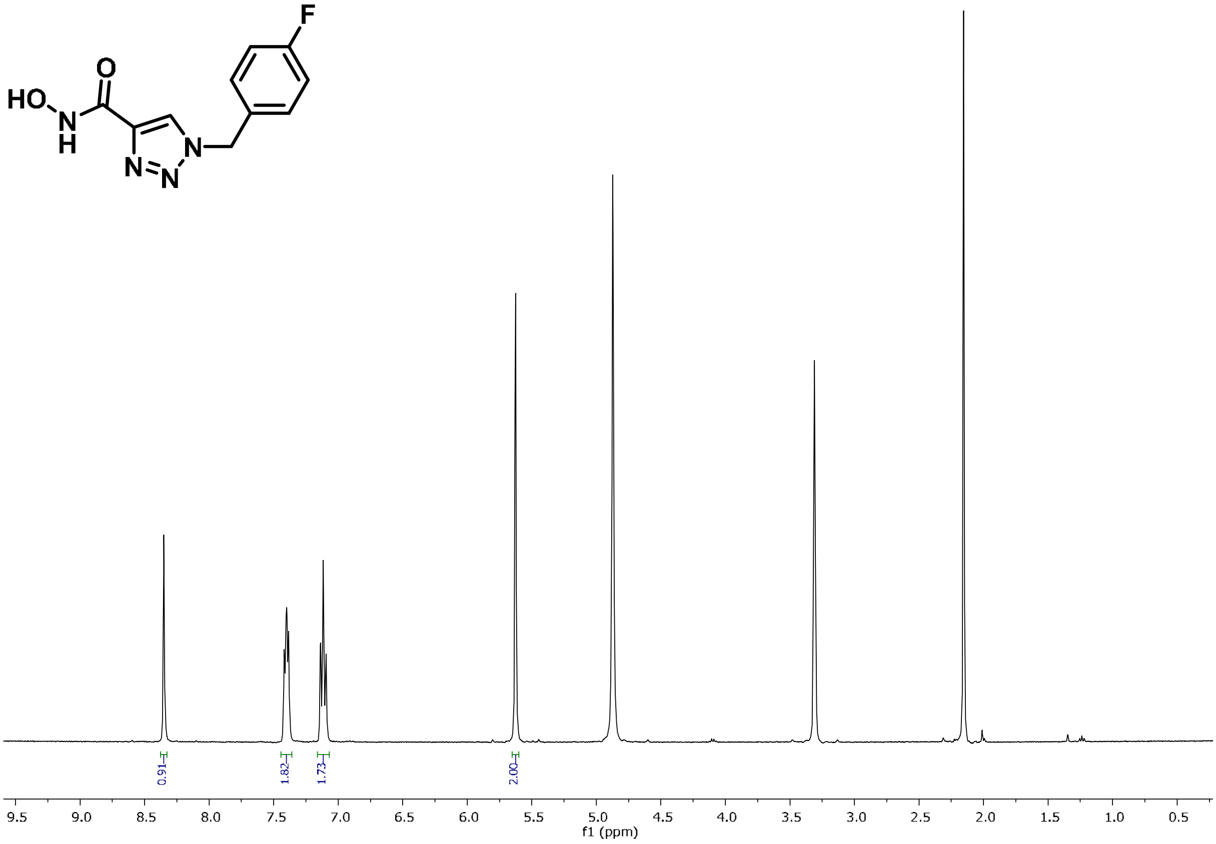

HO
N
H
O
N
N
N
F
0.91
1.82
1.73
2.00
9.5 9.0 8.5 8.0 7.5 7.0 6.5 6.0 5.5 5.0 4.5 4.0 3.5 3.0 2.5 2.0 1.5 1.0 0.5
f1 (ppm)

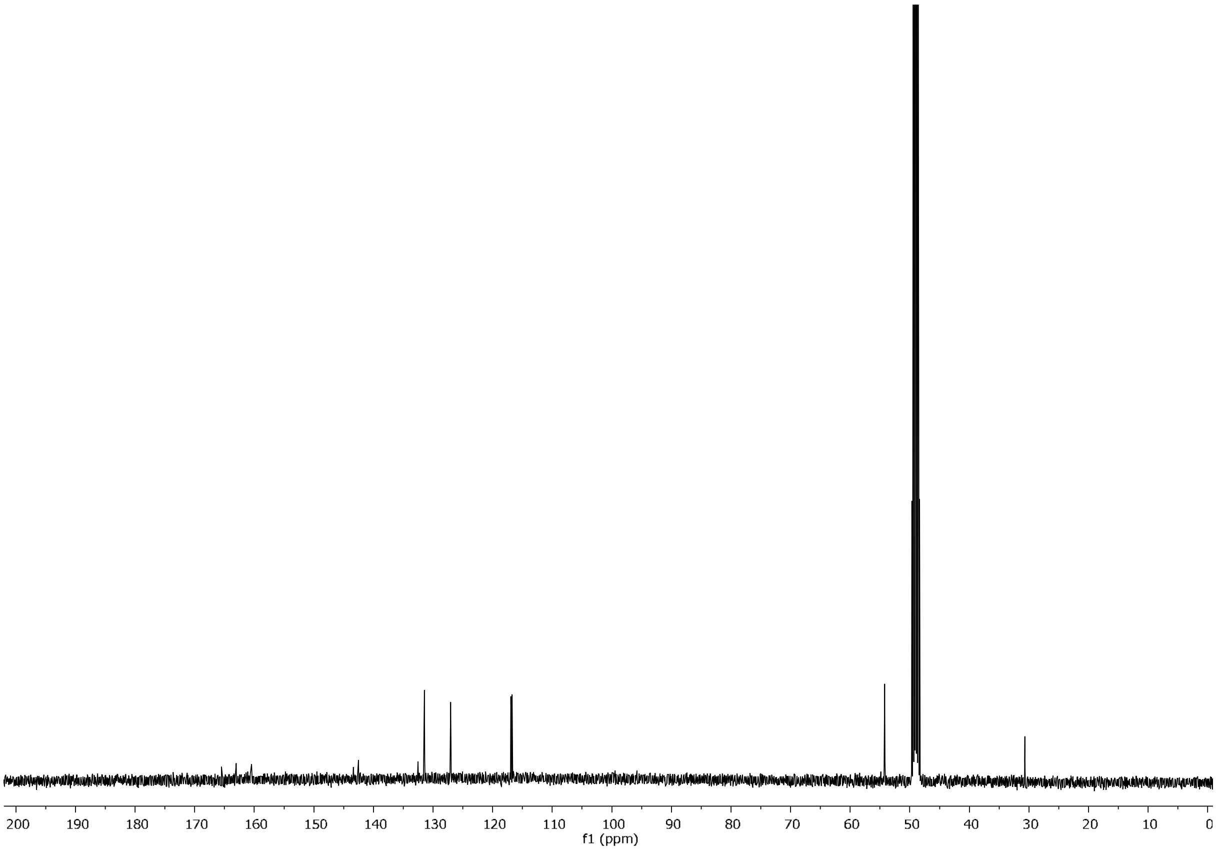

200 190 180 170 160 150 140 130 120 110 100 90 80 70 60 50 40 30 20 10 0
f1 (ppm)

Compound **2e**

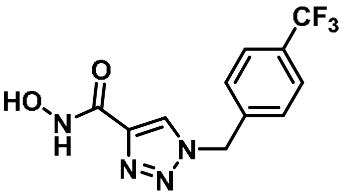

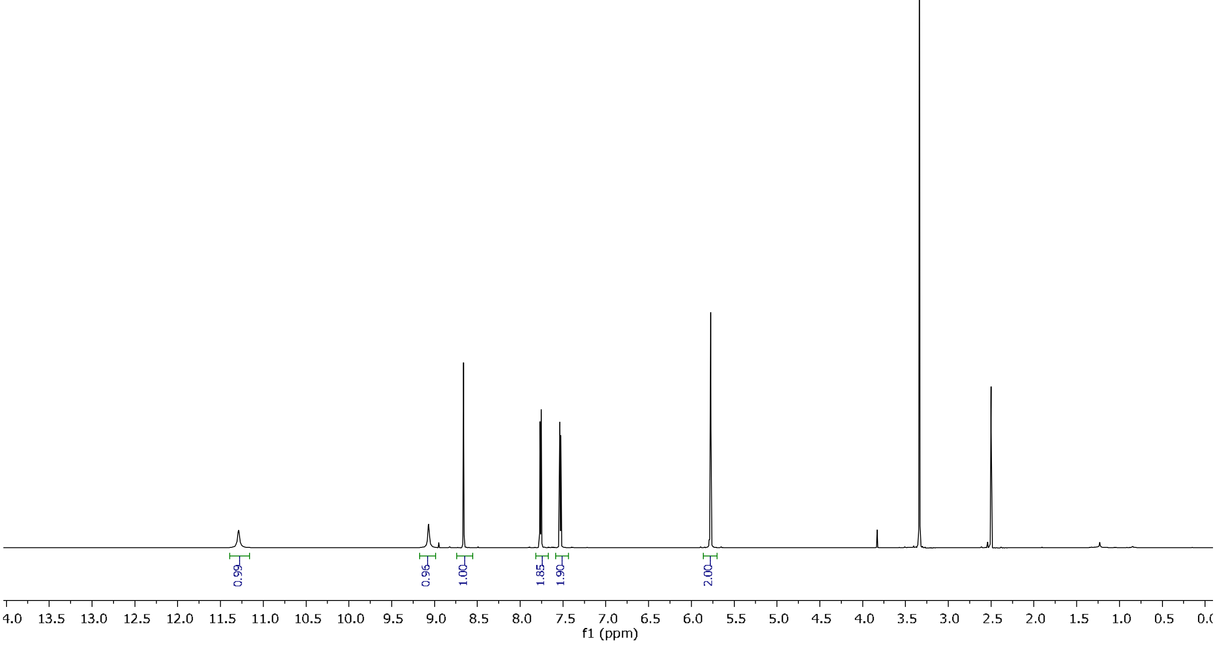

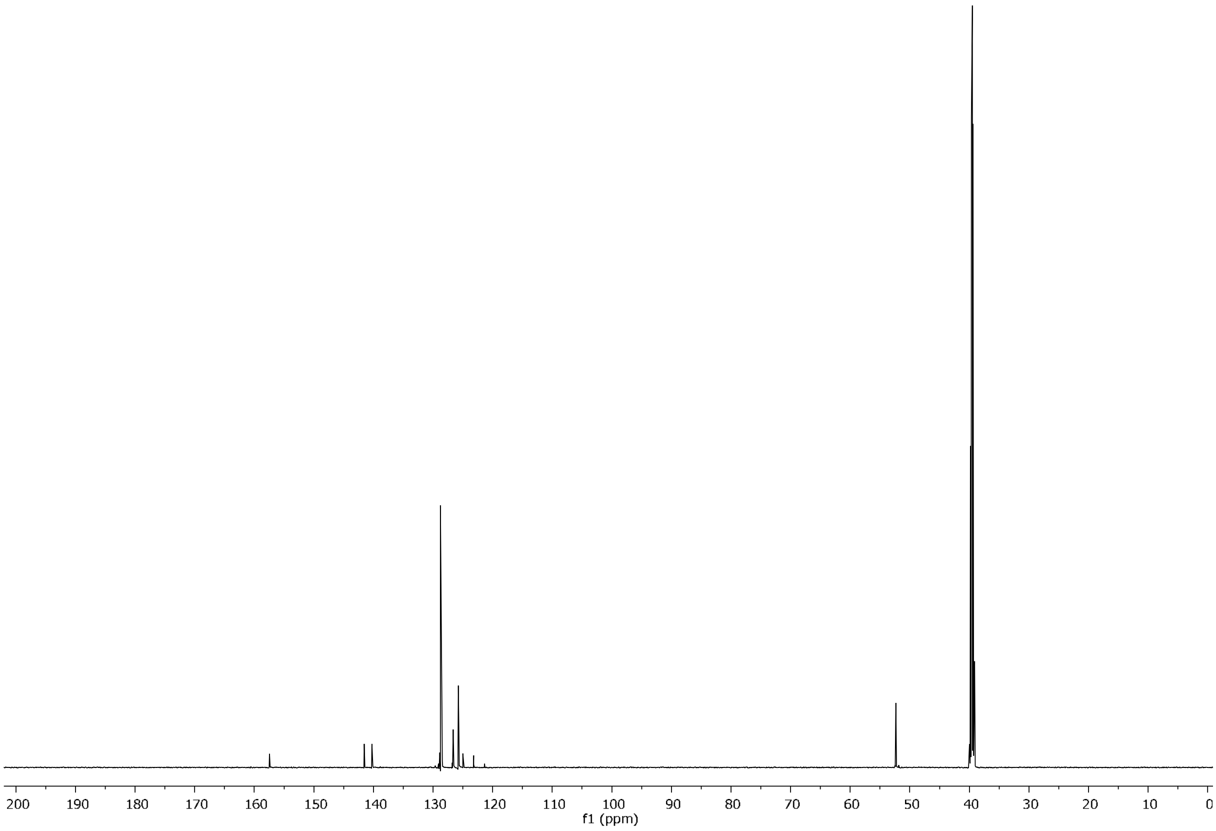

Compound **2f**

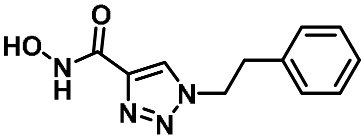

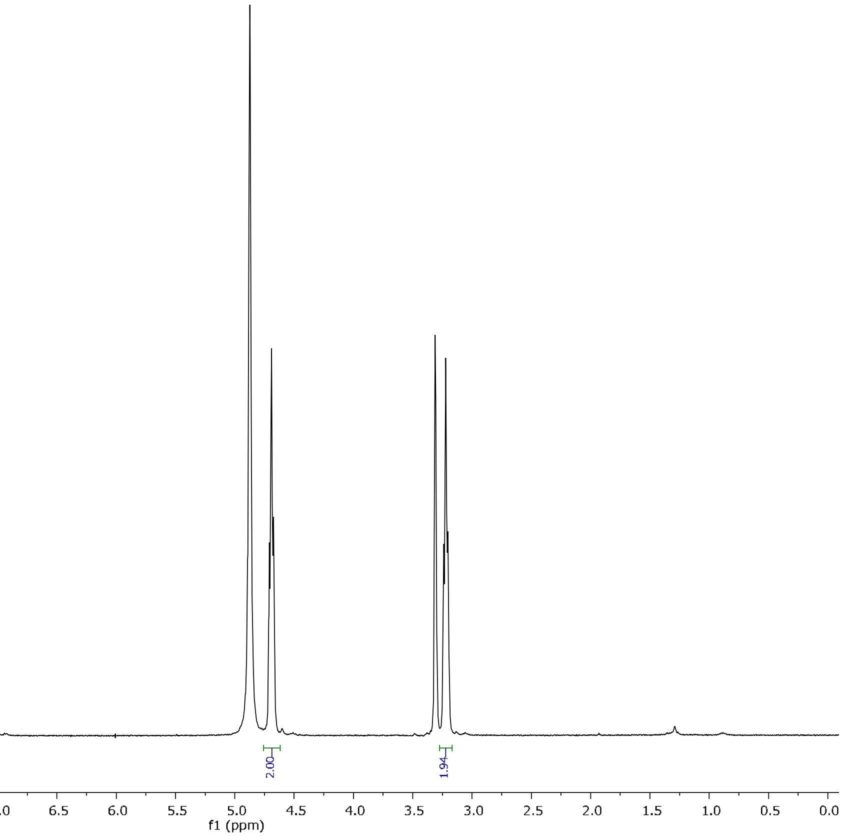
0.99
2.78
1.90
2.00
1.94
10.0
9.5
9.0
8.5
8.0
7.5
7.0
6.5
6.0
5.5
5.0
4.5
4.0
3.5
3.0
2.5
2.0
1.5
1.0
0.5
0.0
f1 (ppm)

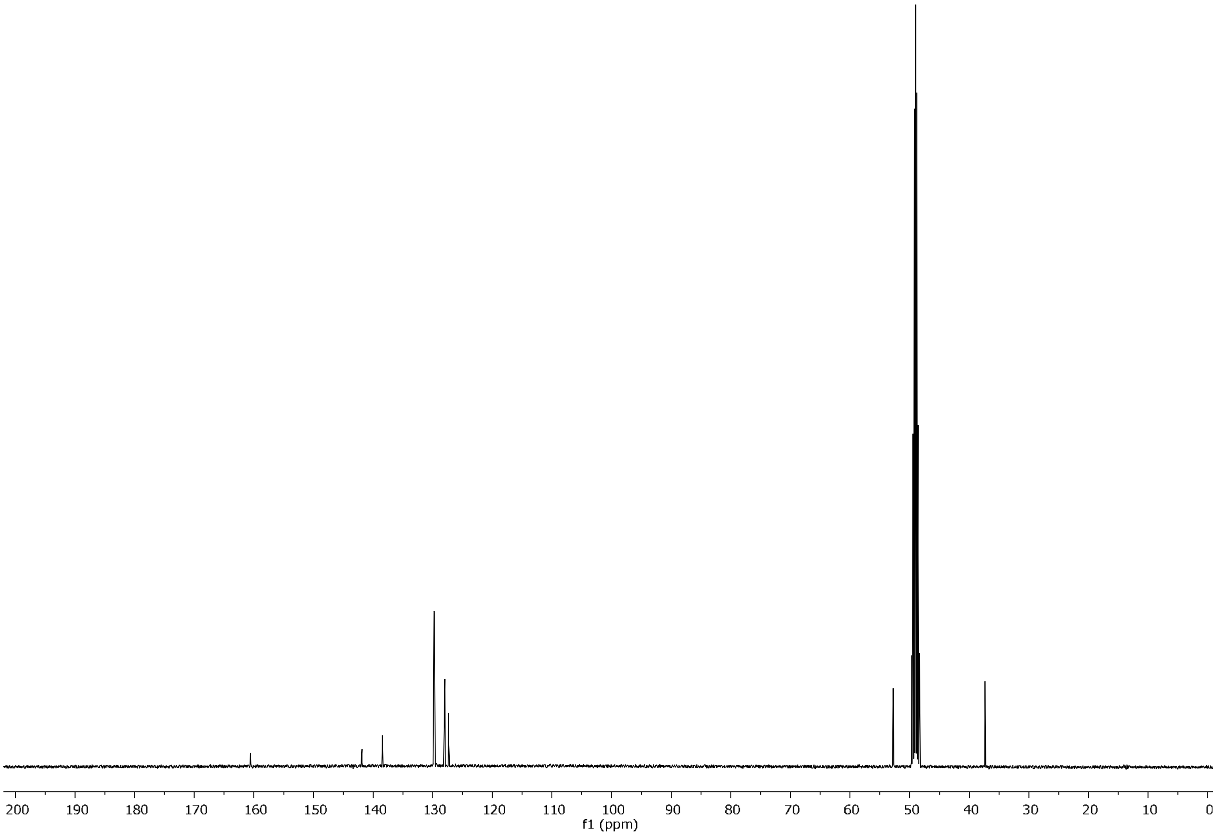
200
190
180
170
160
150
140
130
120
110
100
90
80
70
60
50
40
30
20
10
0
f1 (ppm)

Compound **2g**

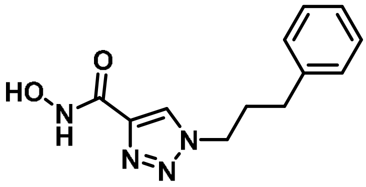

HO
N
H
O
N
N
N

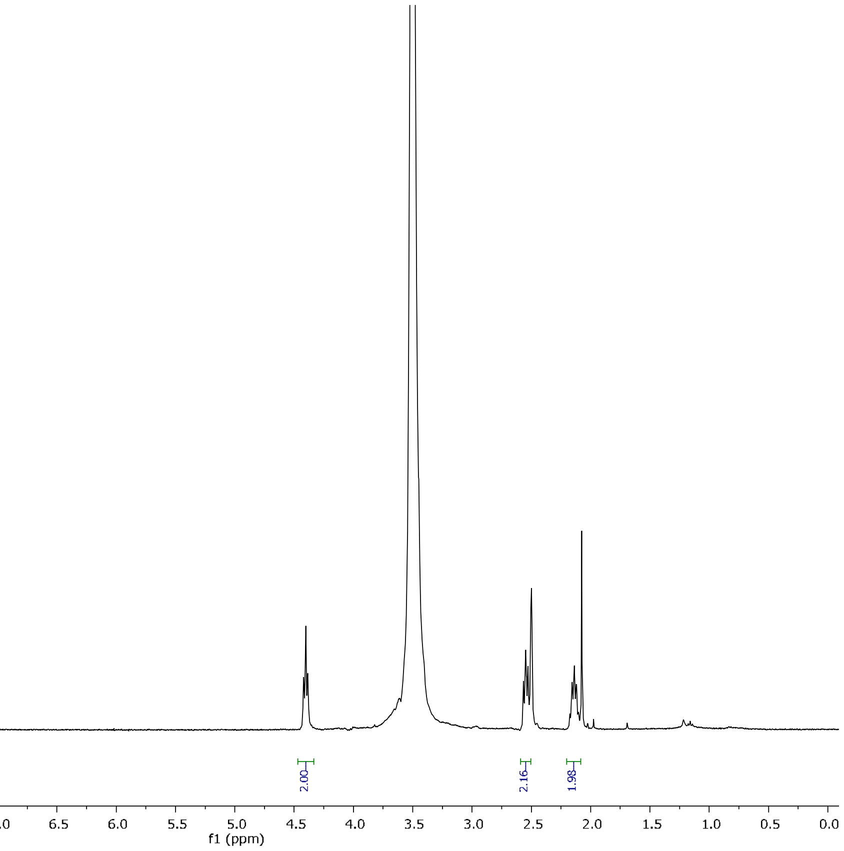

0.95
1.78
2.65
2.00
2.16
1.98
10.0 9.5 9.0 8.5 8.0 7.5 7.0 6.5 6.0 5.5 5.0 4.5 4.0 3.5 3.0 2.5 2.0 1.5 1.0 0.5 0.0
f1 (ppm)

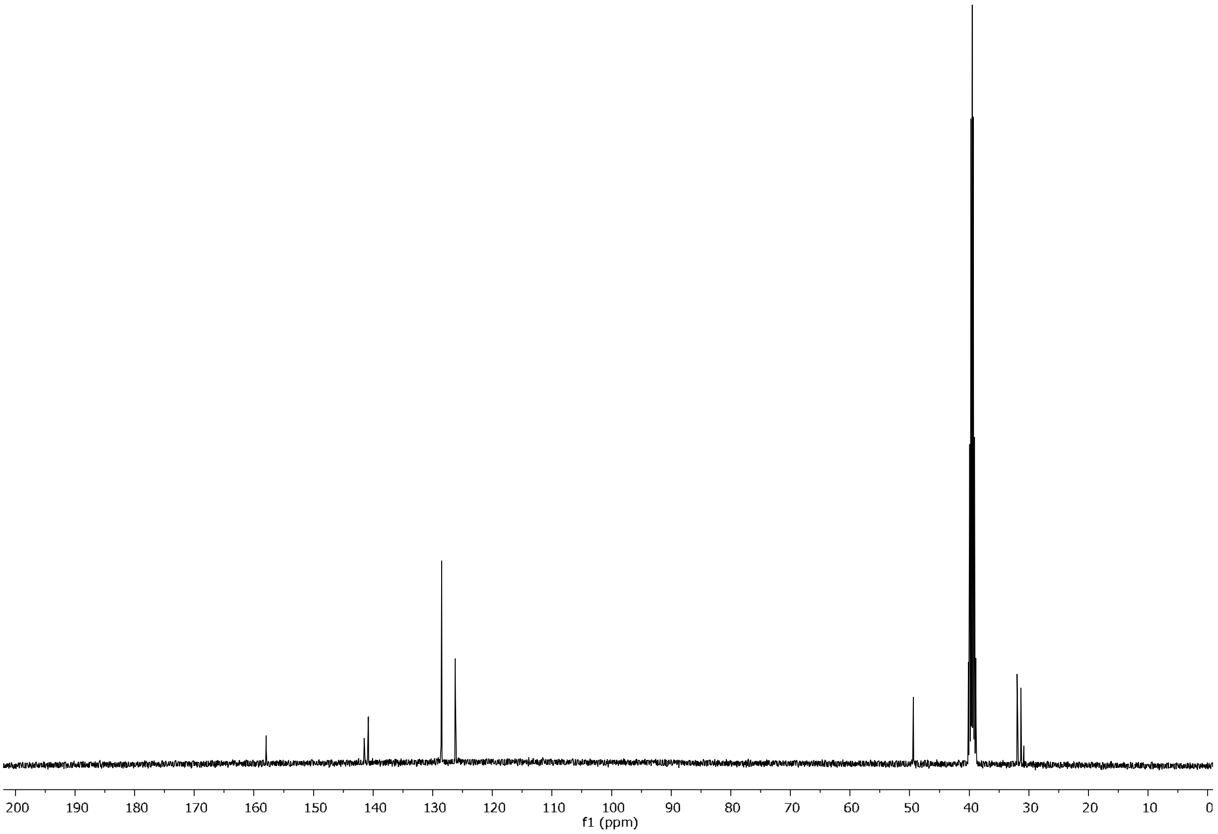

200 190 180 170 160 150 140 130 120 110 100 90 80 70 60 50 40 30 20 10 0
f1 (ppm)

Compound **2h**

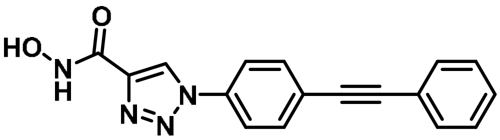

O
HO
N
H
N
N
N

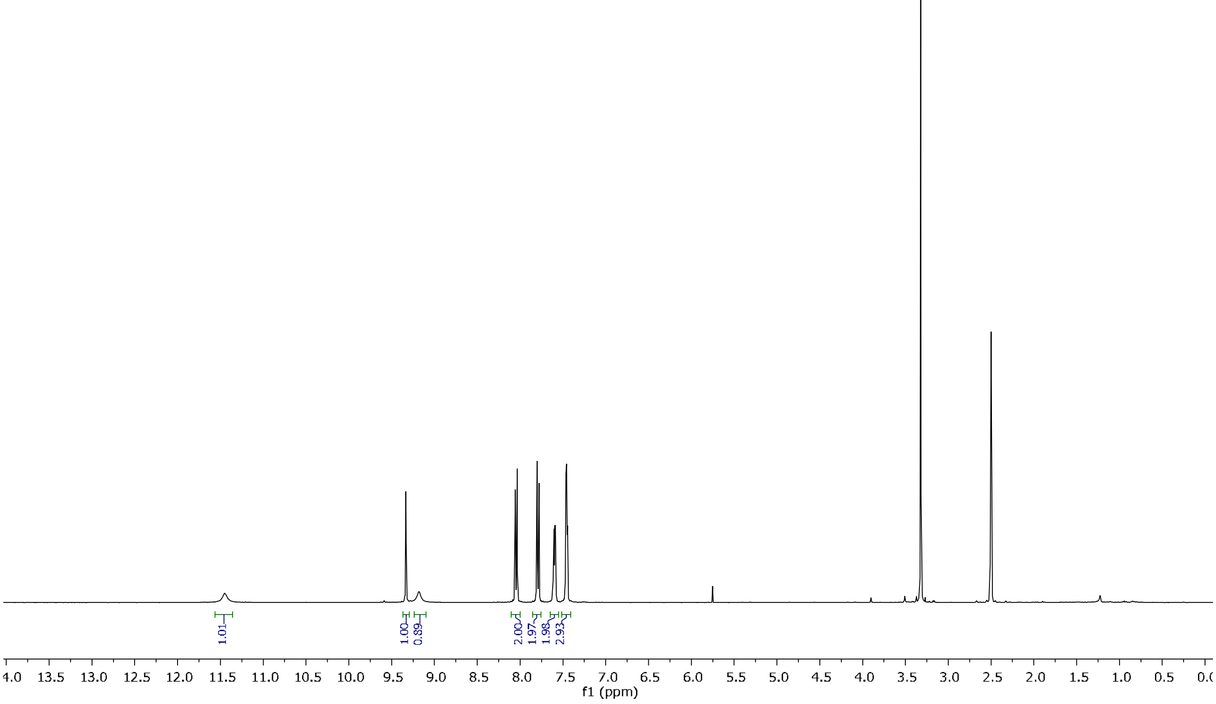

1.01
1.00
0.89
2.00
1.97
1.98
2.93
4.0 13.5 13.0 12.5 12.0 11.5 11.0 10.5 10.0 9.5 9.0 8.5 8.0 7.5 7.0 6.5 6.0 5.5 5.0 4.5 4.0 3.5 3.0 2.5 2.0 1.5 1.0 0.5 0.(
f1 (ppm)

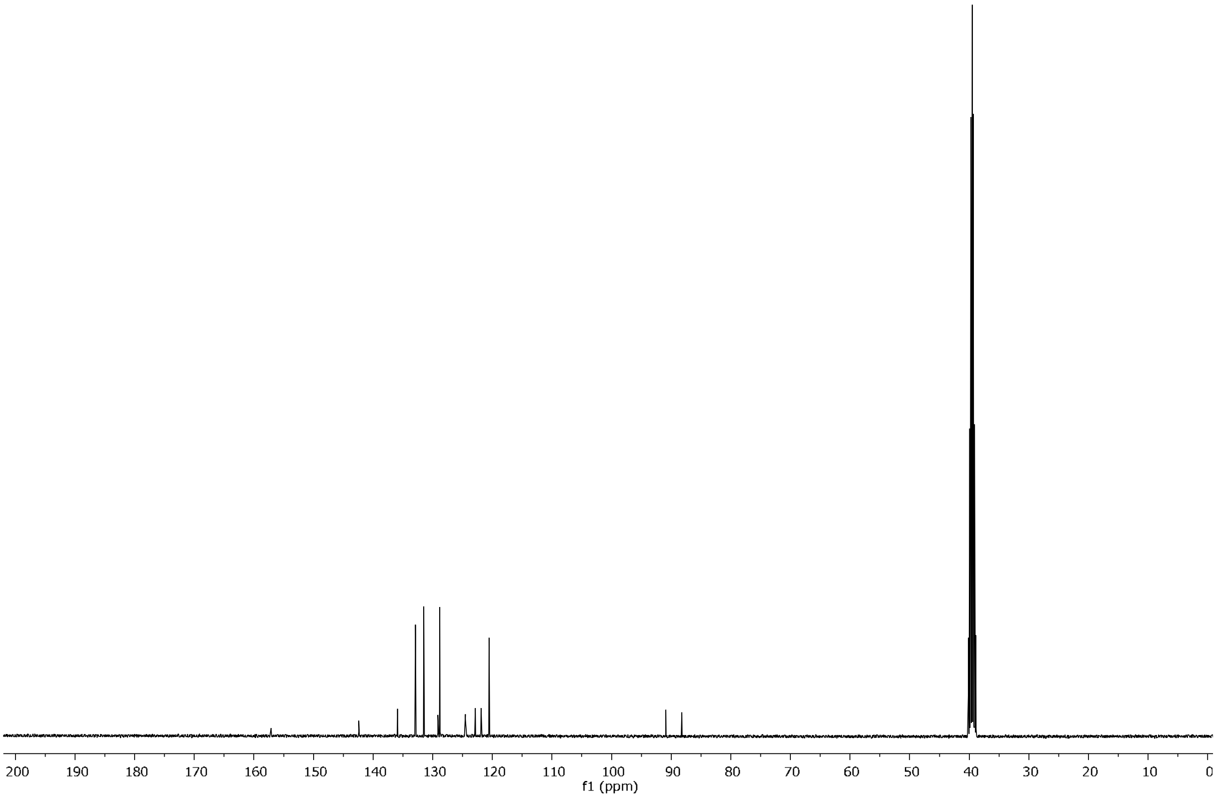

200 190 180 170 160 150 140 130 120 110 100 90 80 70 60 50 40 30 20 10 0
f1 (ppm)

Compound **2i**

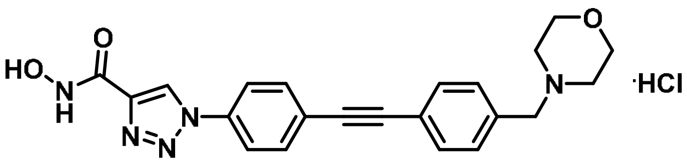

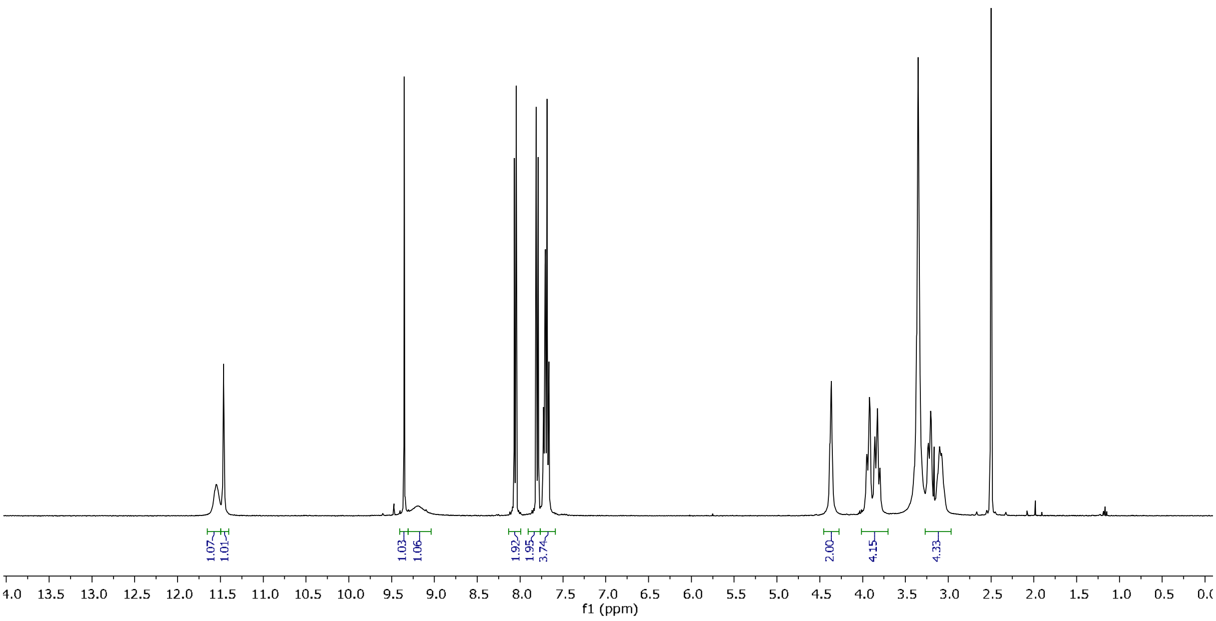

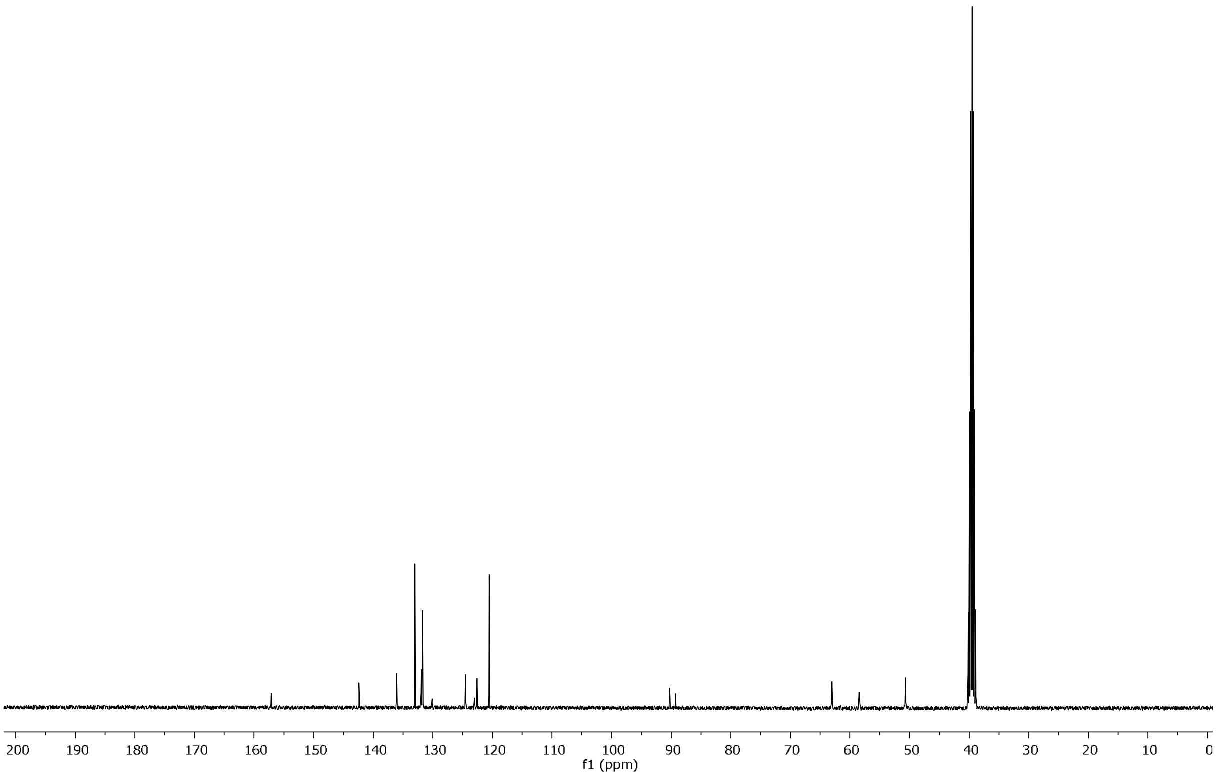

Compound **2j**

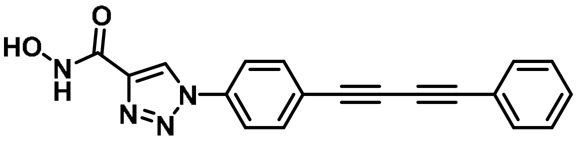

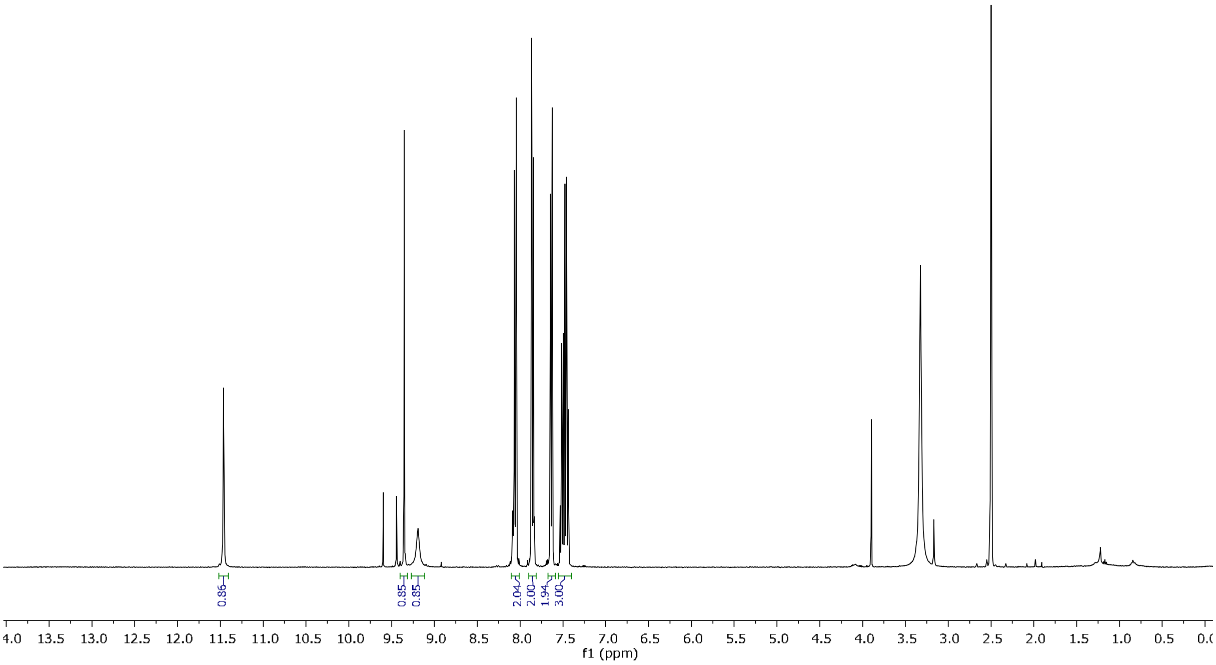
0.86
0.85
0.85
2.04
2.00
1.94
3.00
4.0 13.5 13.0 12.5 12.0 11.5 11.0 10.5 10.0 9.5 9.0 8.5 8.0 7.5 7.0 6.5 6.0 5.5 5.0 4.5 4.0 3.5 3.0 2.5 2.0 1.5 1.0 0.5 0.0
f1 (ppm)

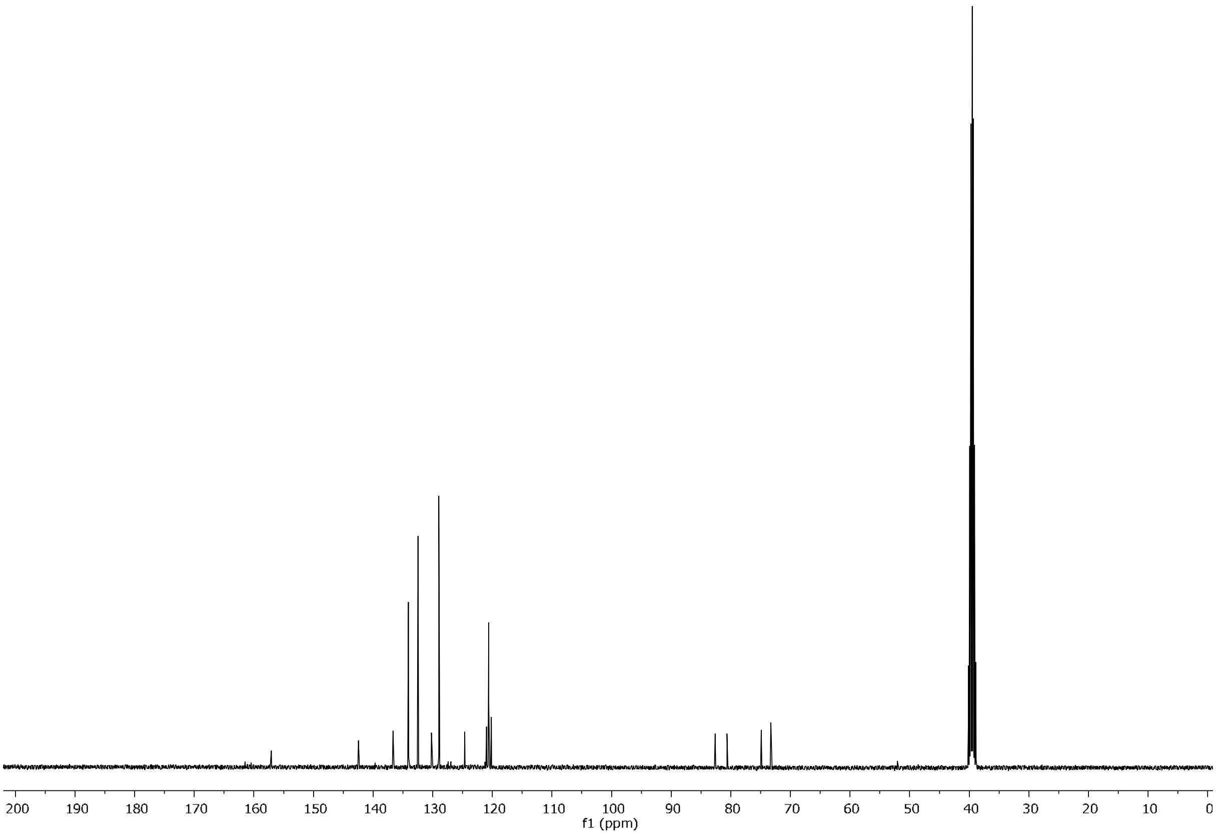
200 190 180 170 160 150 140 130 120 110 100 90 80 70 60 50 40 30 20 10 0
f1 (ppm)

Compound **10**

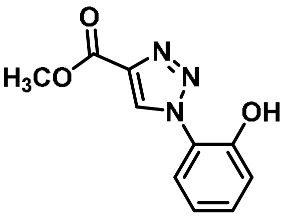

O
H3CO
N
N
N
OH

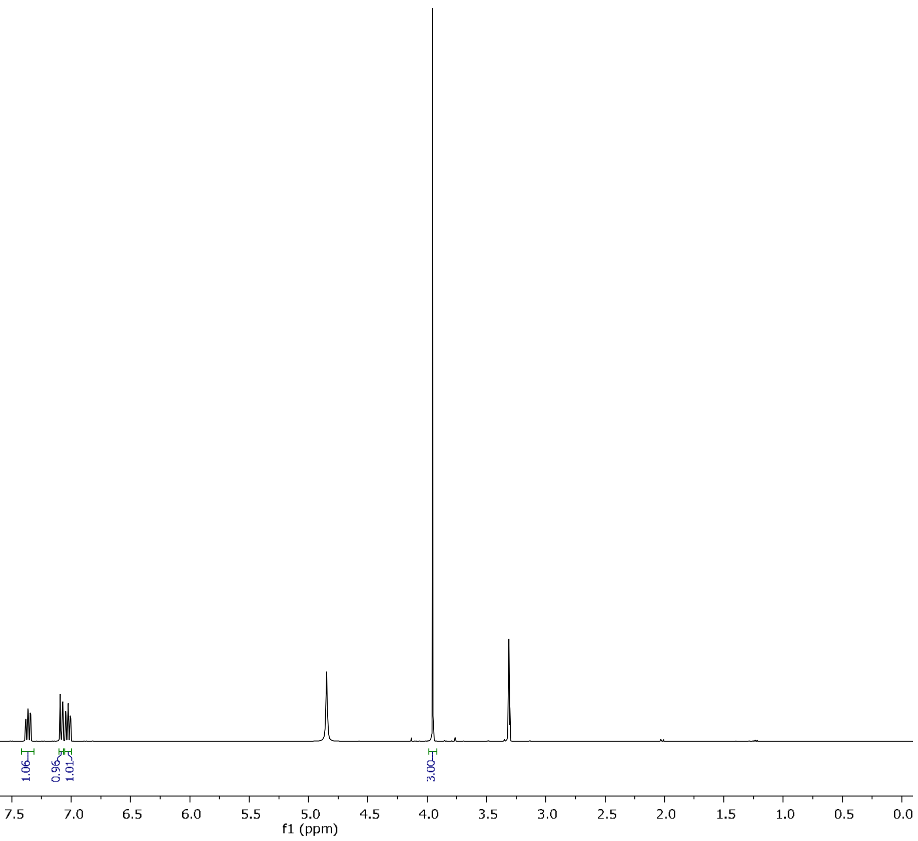

0.94
1.03
1.06
0.96
1.01
3.00
10.0
9.5
9.0
8.5
8.0
7.5
7.0
6.5
6.0
5.5
5.0
4.5
4.0
3.5
3.0
2.5
2.0
1.5
1.0
0.5
0.0
f1 (ppm)

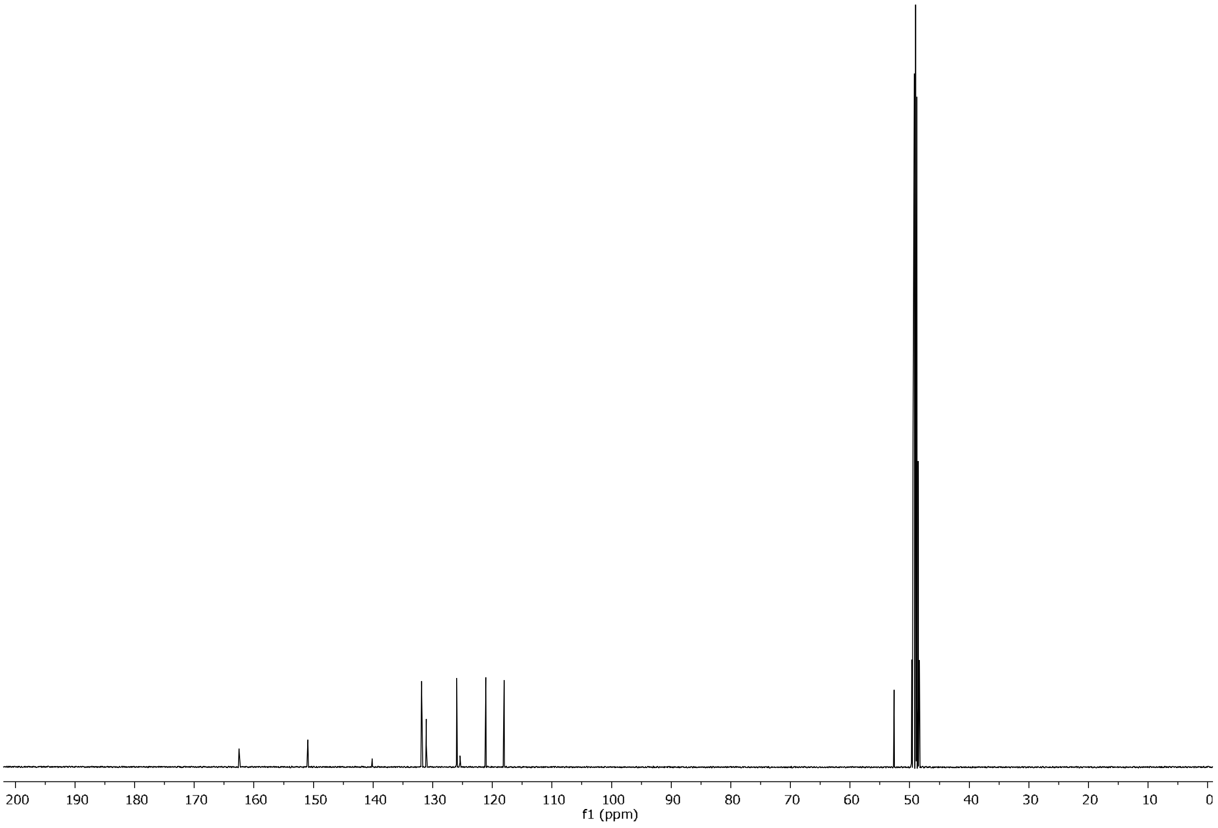

200
190
180
170
160
150
140
130
120
110
100
90
80
70
60
50
40
30
20
10
0
f1 (ppm)

Compound **11**

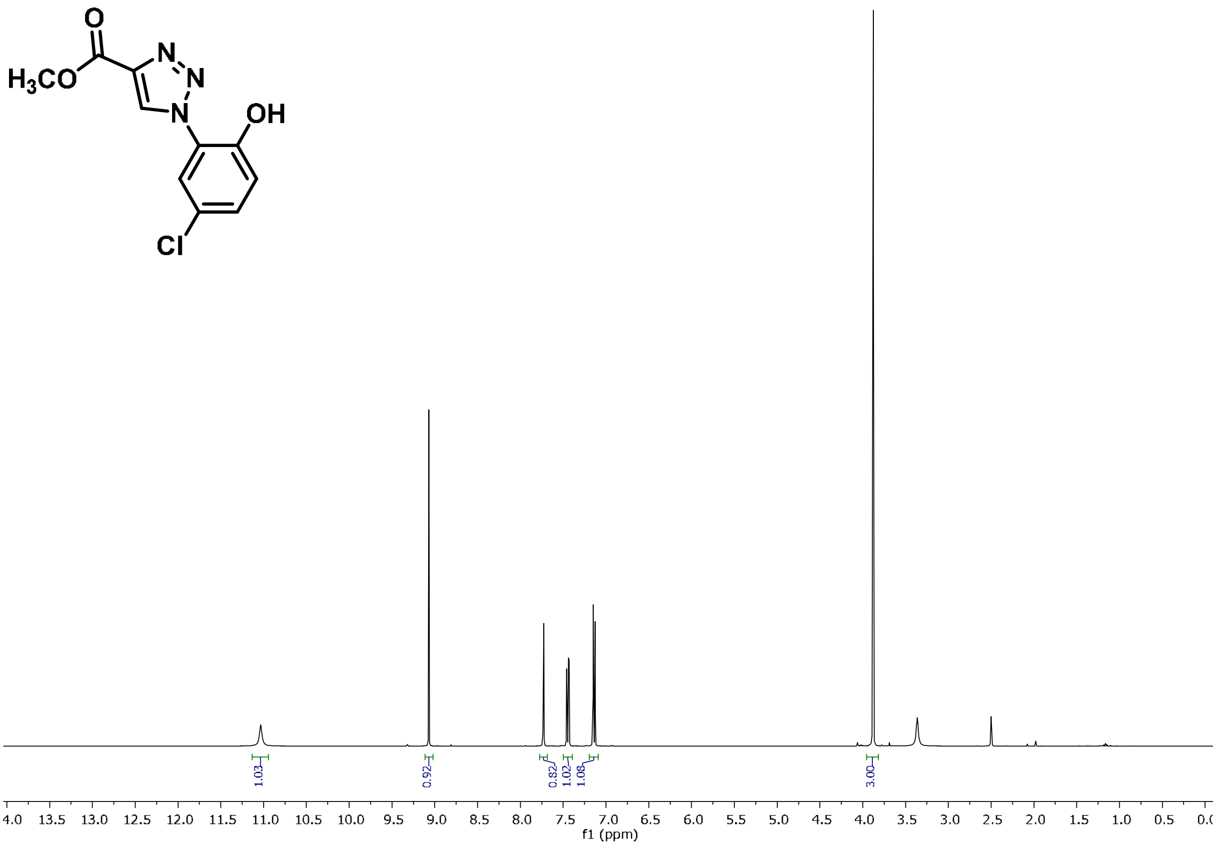

H3CO
N
N
N
OH
Cl
1.03
0.92
0.82
1.02
1.08
3.00
.0 13.5 13.0 12.5 12.0 11.5 11.0 10.5 10.0 9.5 9.0 8.5 8.0 7.5 7.0 6.5 6.0 5.5 5.0 4.5 4.0 3.5 3.0 2.5 2.0 1.5 1.0 0.5 0.0
f1 (ppm)

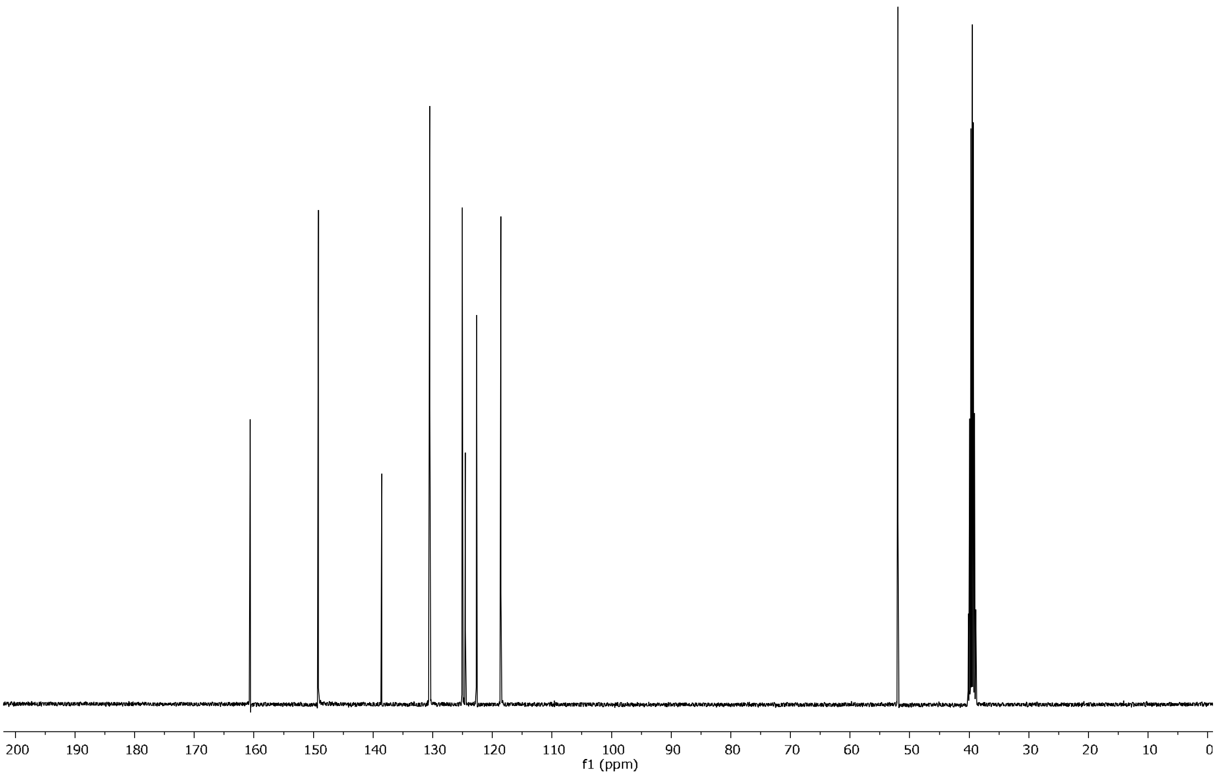

200 190 180 170 160 150 140 130 120 110 100 90 80 70 60 50 40 30 20 10 0
f1 (ppm)

Compound **12**

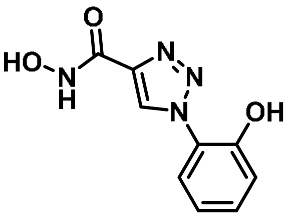
HO
N
H
O
N
N
N
OH

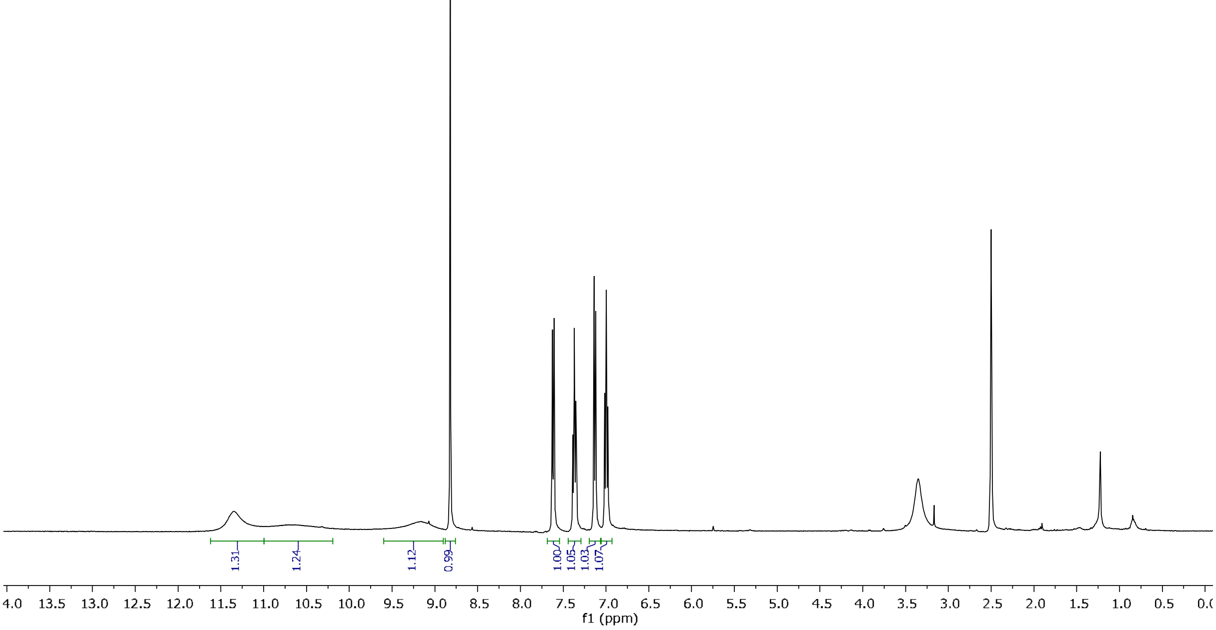
1.31
1.24
1.12
0.99
1.00
1.05
1.03
1.07
14.0 13.5 13.0 12.5 12.0 11.5 11.0 10.5 10.0 9.5 9.0 8.5 8.0 7.5 7.0 6.5 6.0 5.5 5.0 4.5 4.0 3.5 3.0 2.5 2.0 1.5 1.0 0.5 0.0
f1 (ppm)

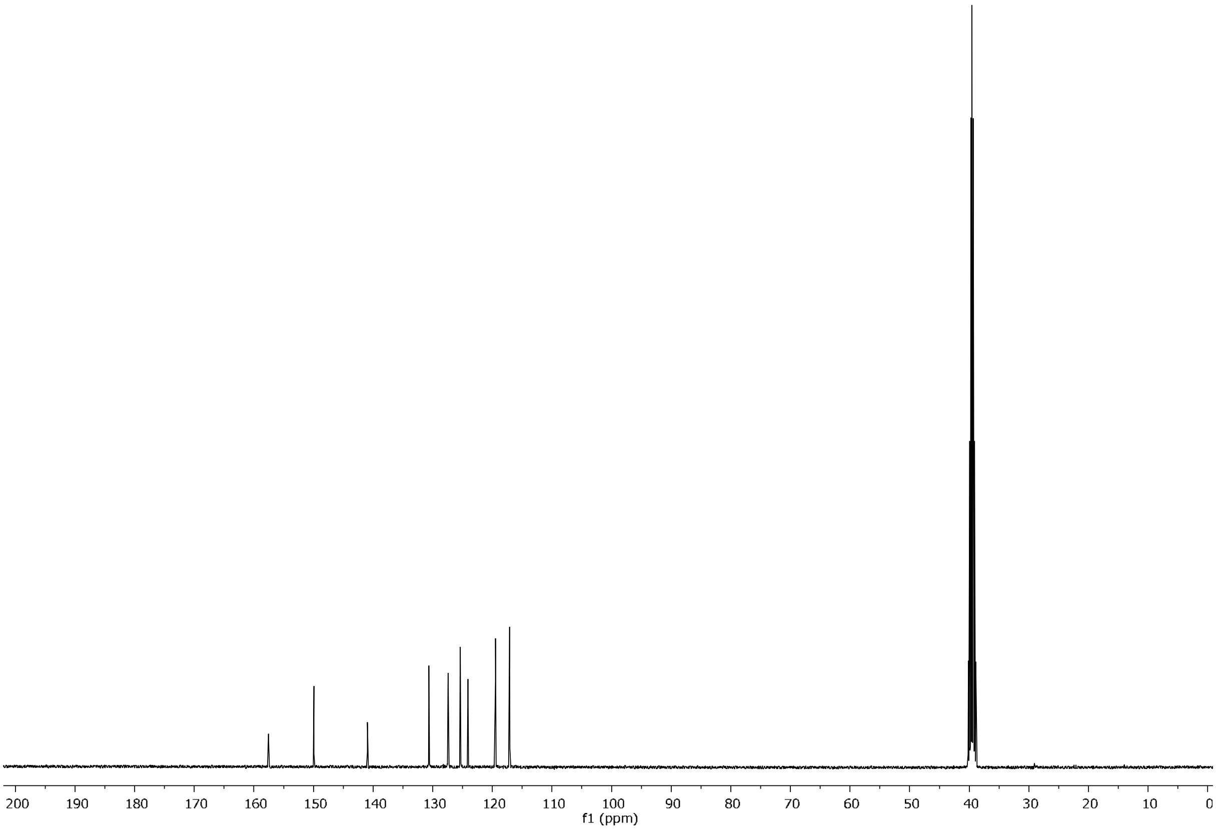
200 190 180 170 160 150 140 130 120 110 100 90 80 70 60 50 40 30 20 10 0
f1 (ppm)

Compound **13**

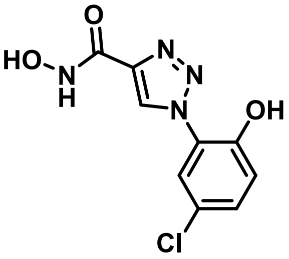

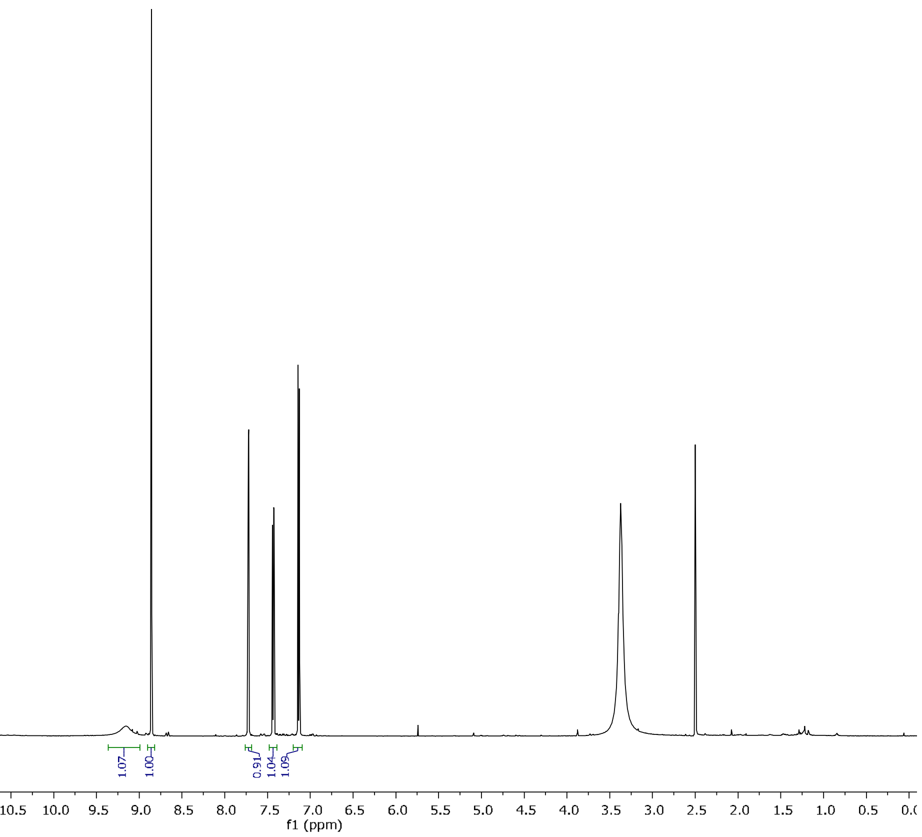

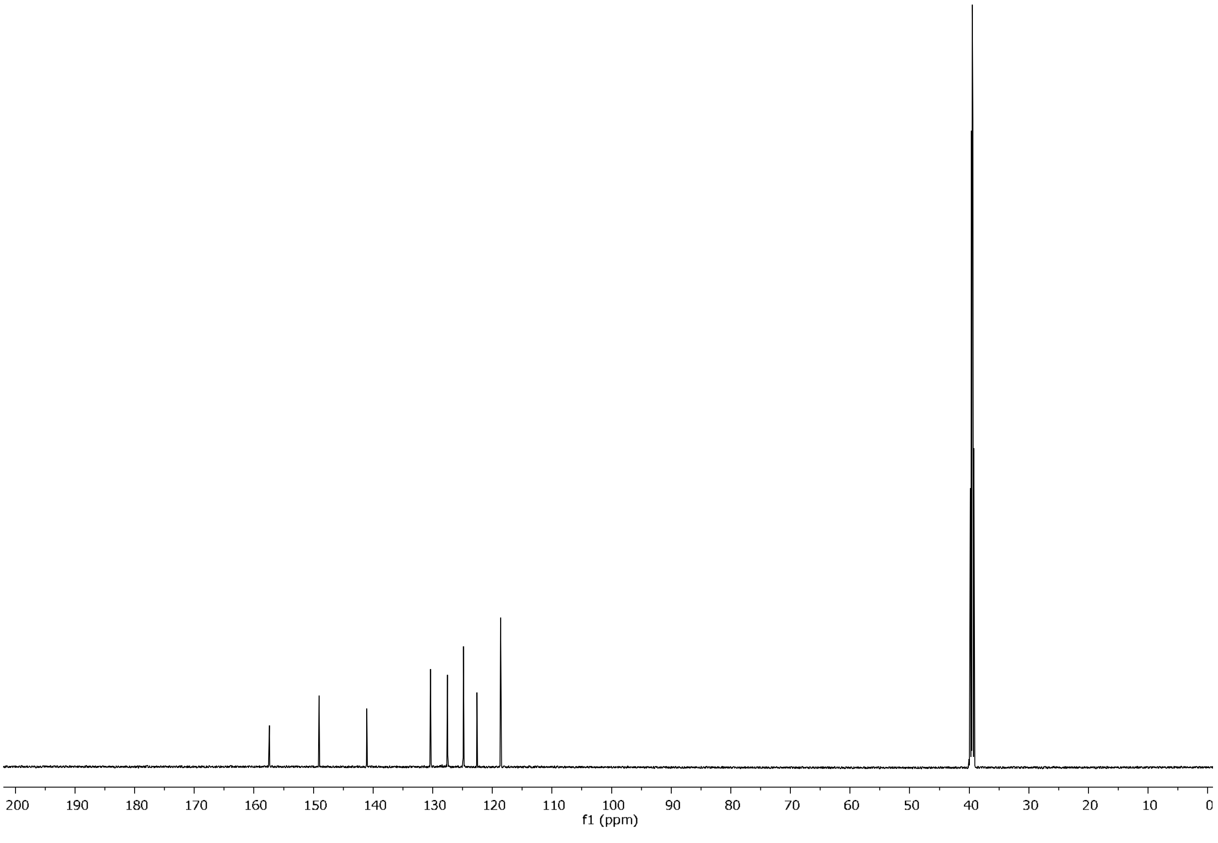

Compound **14**

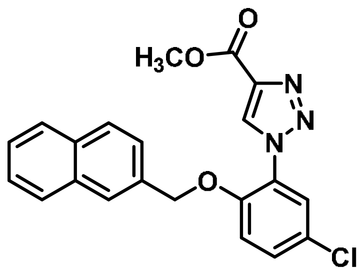

O
H_3CO
N
N
N
O
Cl

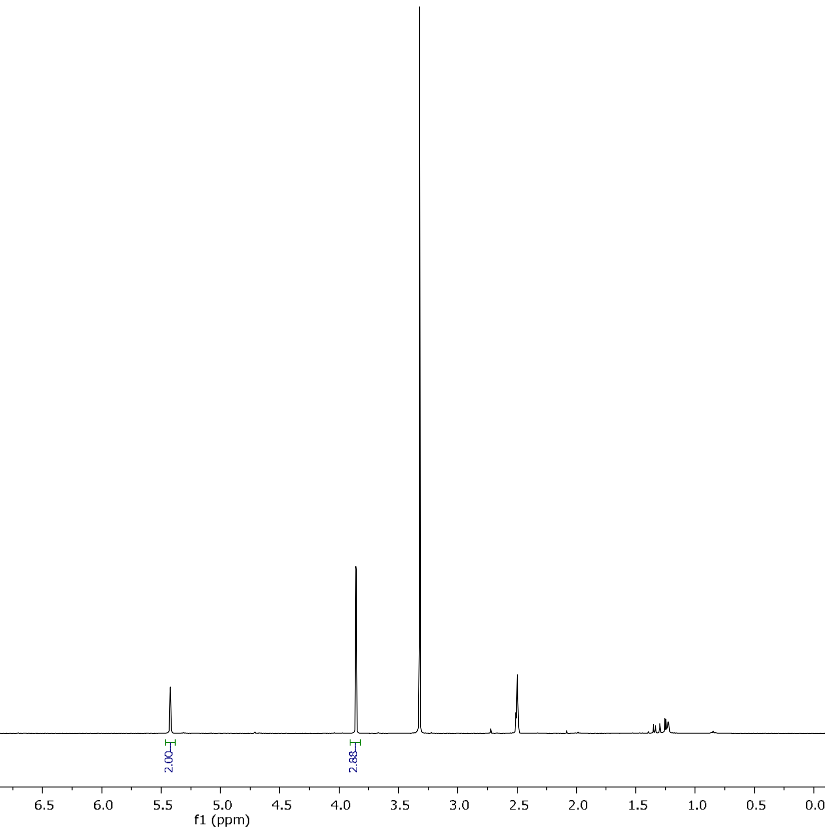

0.95
2.12
2.87
1.12
4.17
2.00
2.88
10.0
9.5
9.0
8.5
8.0
7.5
7.0
6.5
6.0
5.5
5.0
4.5
4.0
3.5
3.0
2.5
2.0
1.5
1.0
0.5
0.0
f1 (ppm)

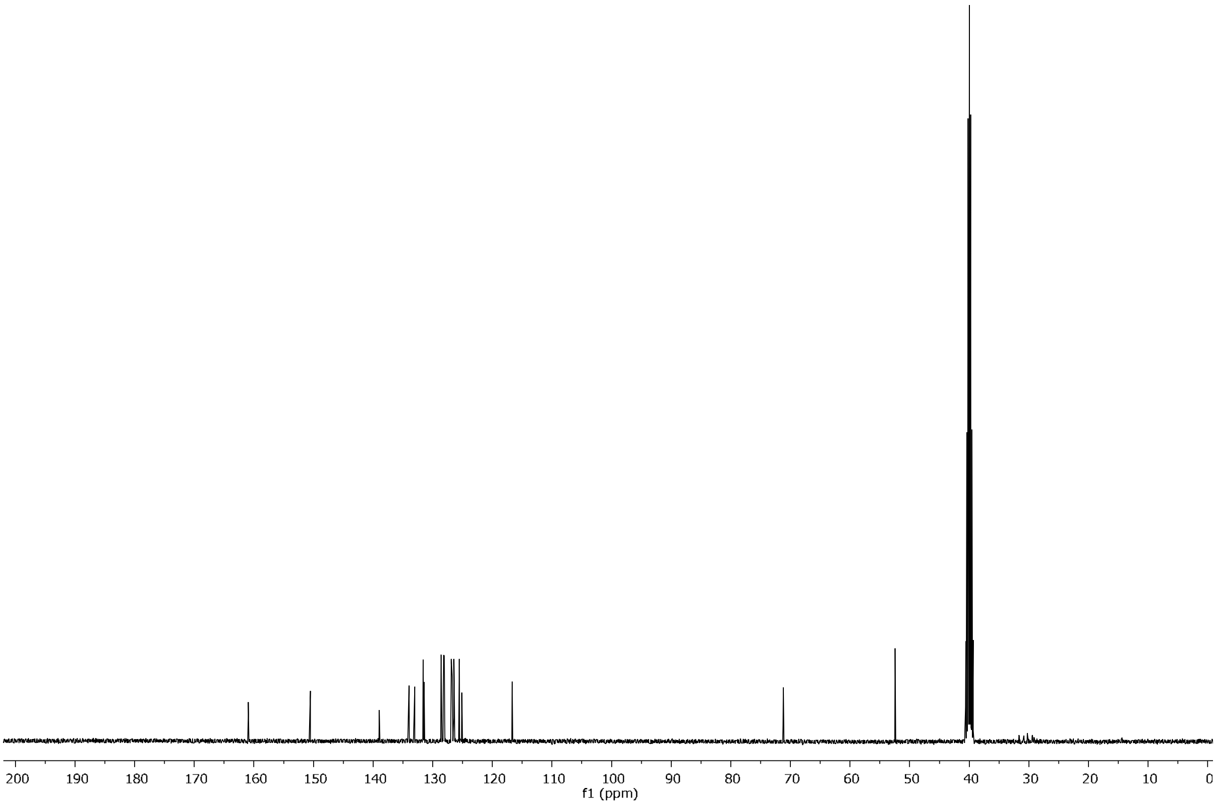

200
190
180
170
160
150
140
130
120
110
100
90
80
70
60
50
40
30
20
10
0
f1 (ppm)

Compound **15**

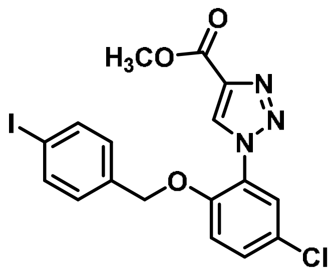

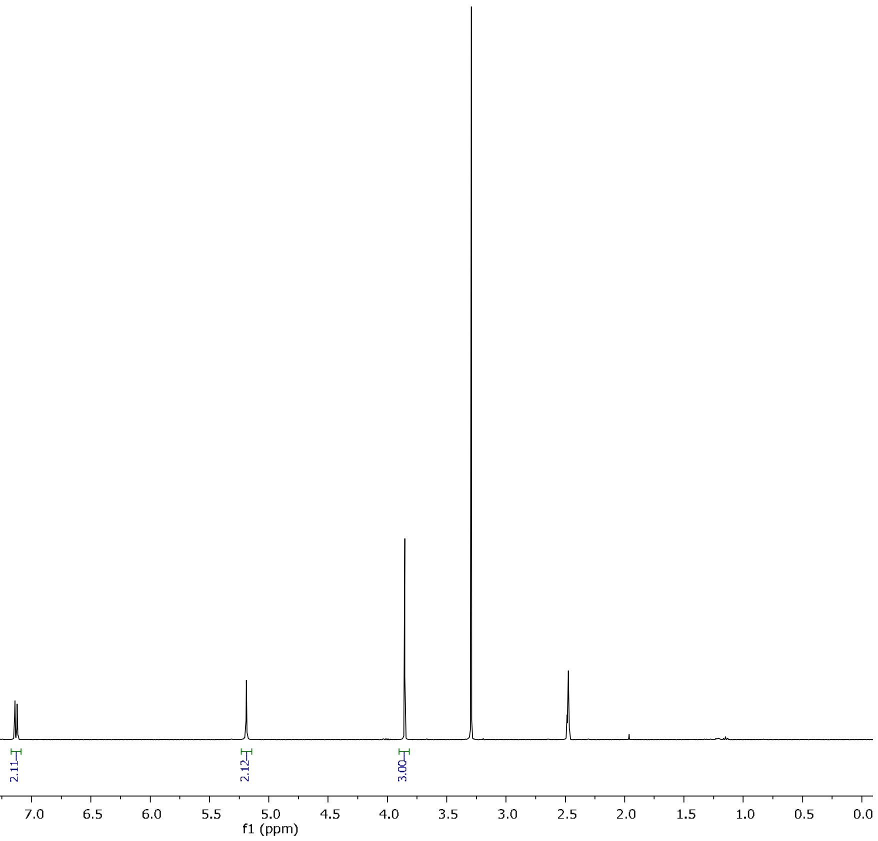
0.96
0.89
2.01
1.13
1.11
2.11
2.12
3.00
10.0 9.5 9.0 8.5 8.0 7.5 7.0 6.5 6.0 5.5 5.0 4.5 4.0 3.5 3.0 2.5 2.0 1.5 1.0 0.5 0.0
f1 (ppm)

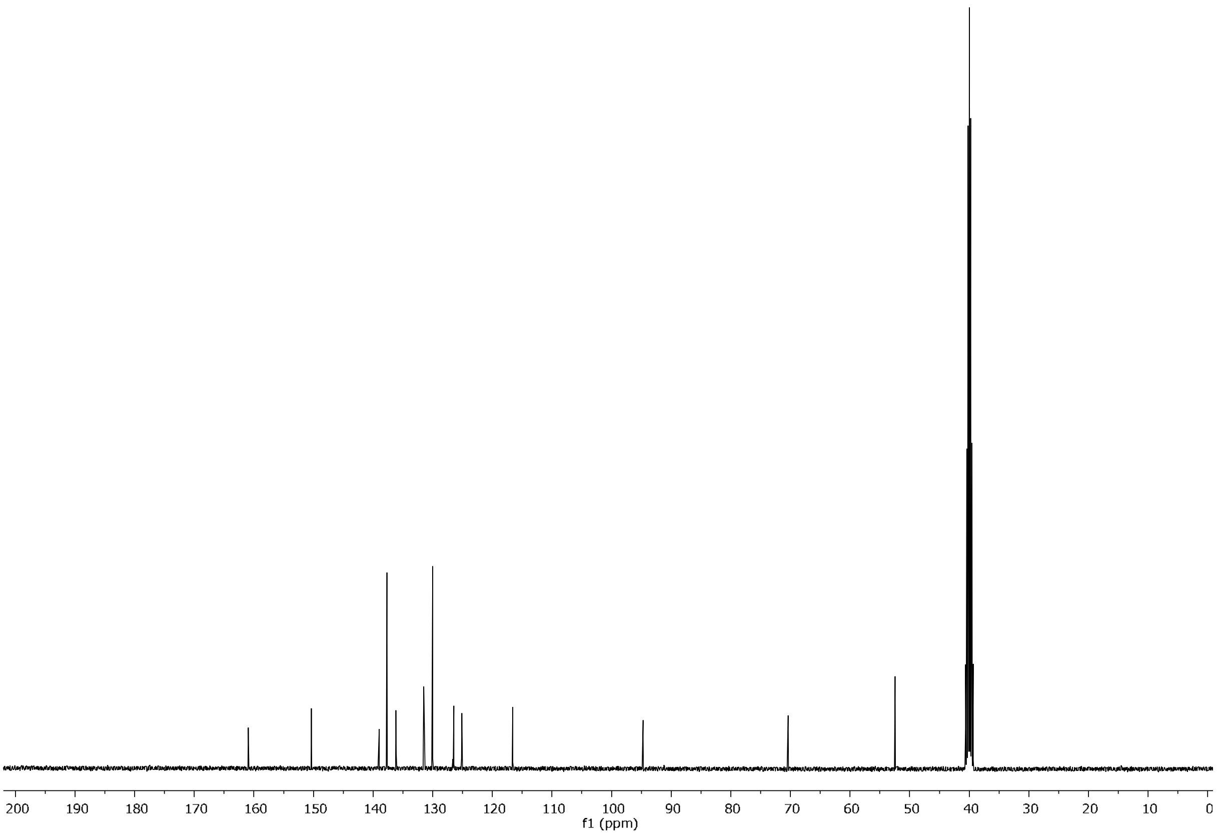
200 190 180 170 160 150 140 130 120 110 100 90 80 70 60 50 40 30 20 10 0
f1 (ppm)

Compound **16**

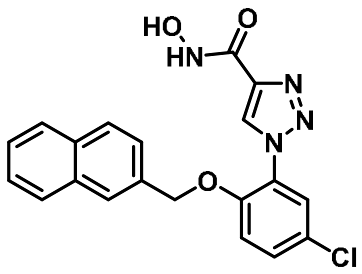
HO
HN
O
N
N
N
O
Cl

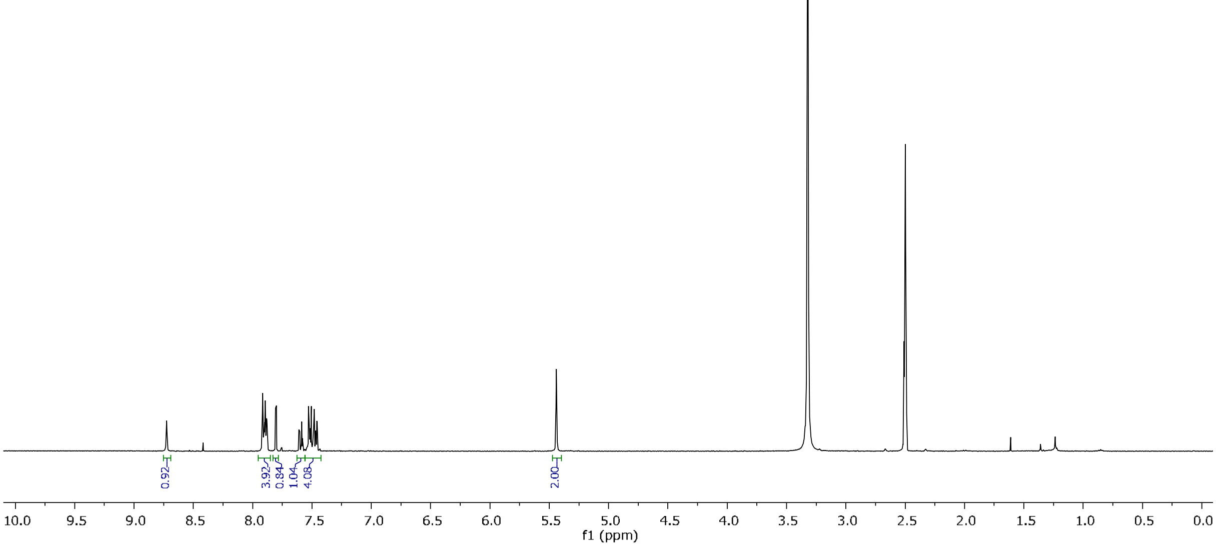
0.92
3.92
0.84
1.04
4.08
2.00
10.0
9.5
9.0
8.5
8.0
7.5
7.0
6.5
6.0
5.5
5.0
4.5
4.0
3.5
3.0
2.5
2.0
1.5
1.0
0.5
0.0
f1 (ppm)

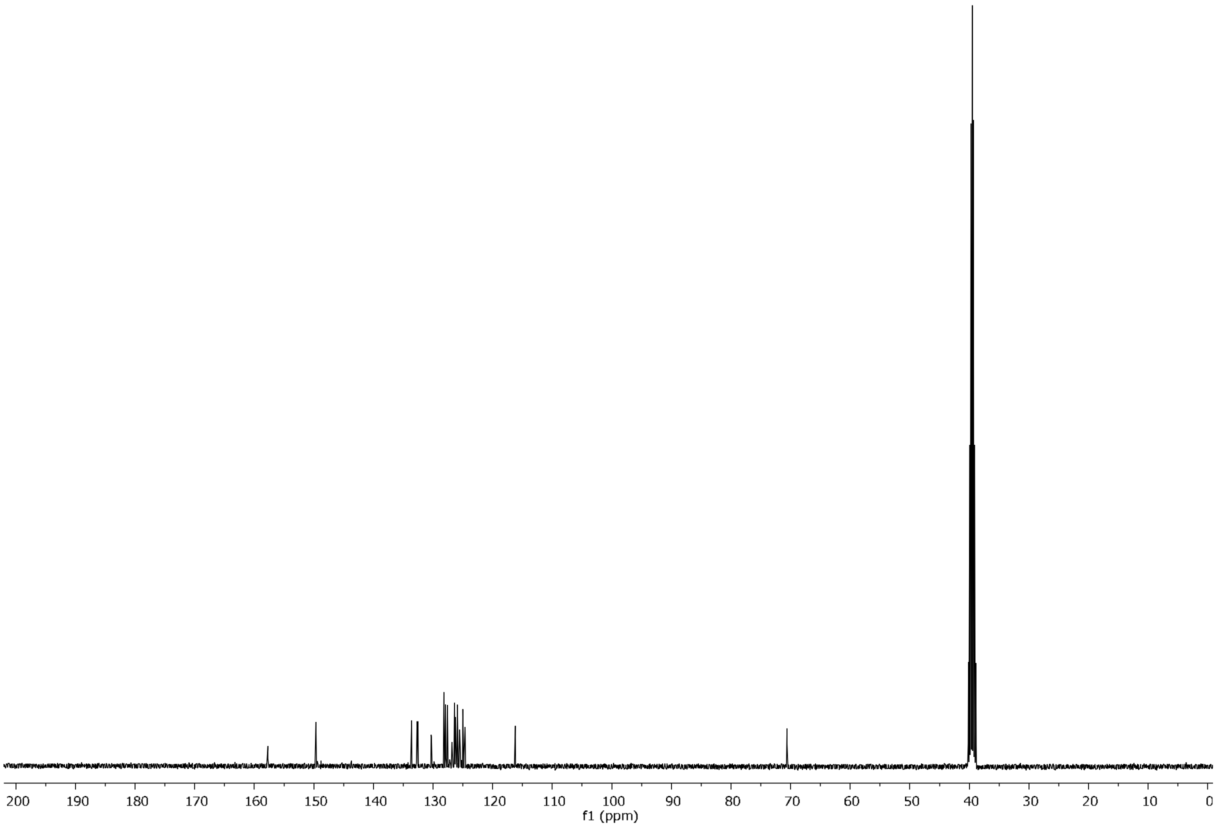
200
190
180
170
160
150
140
130
120
110
100
90
80
70
60
50
40
30
20
10
0
f1 (ppm)

Compound **17**

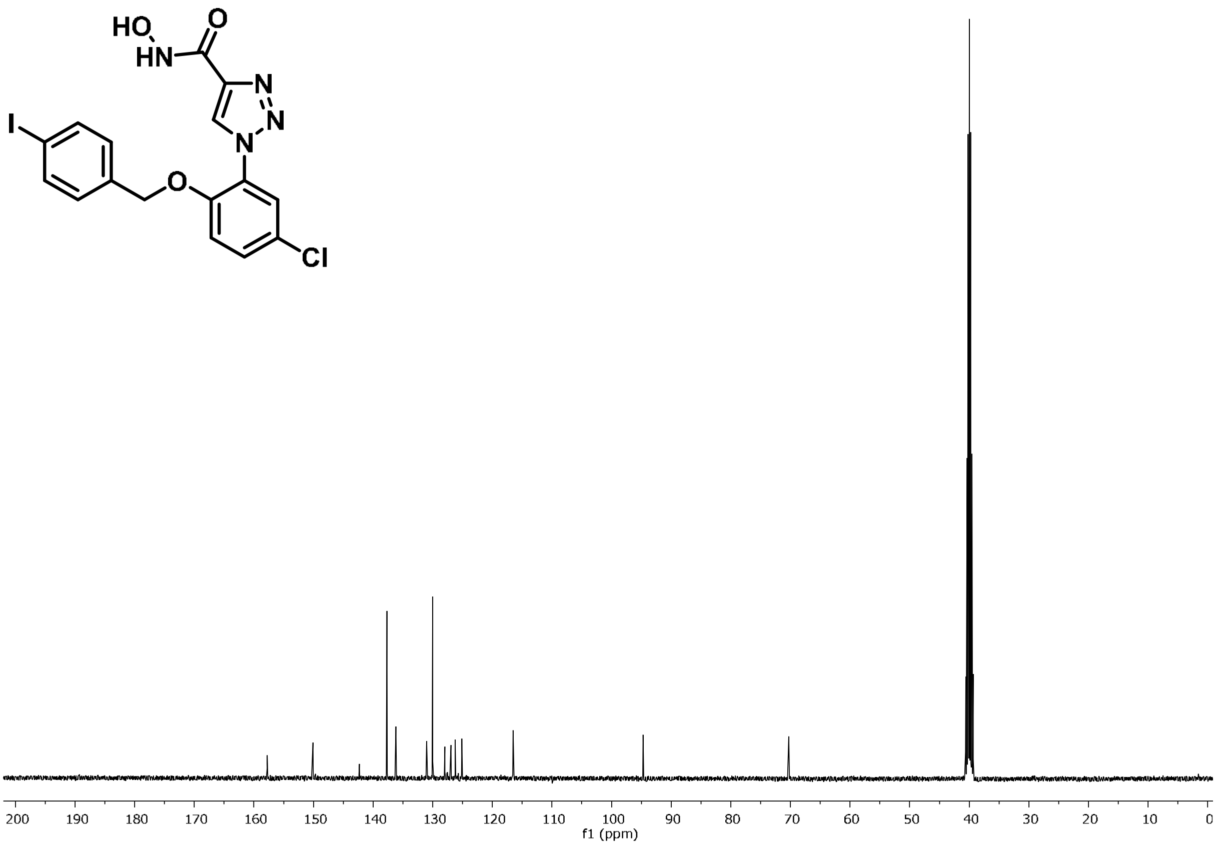
HO
HN
O
N
N
N
I
O
Cl
200 190 180 170 160 150 140 130 120 110 100 90 80 70 60 50 40 30 20 10 0
f1 (ppm)

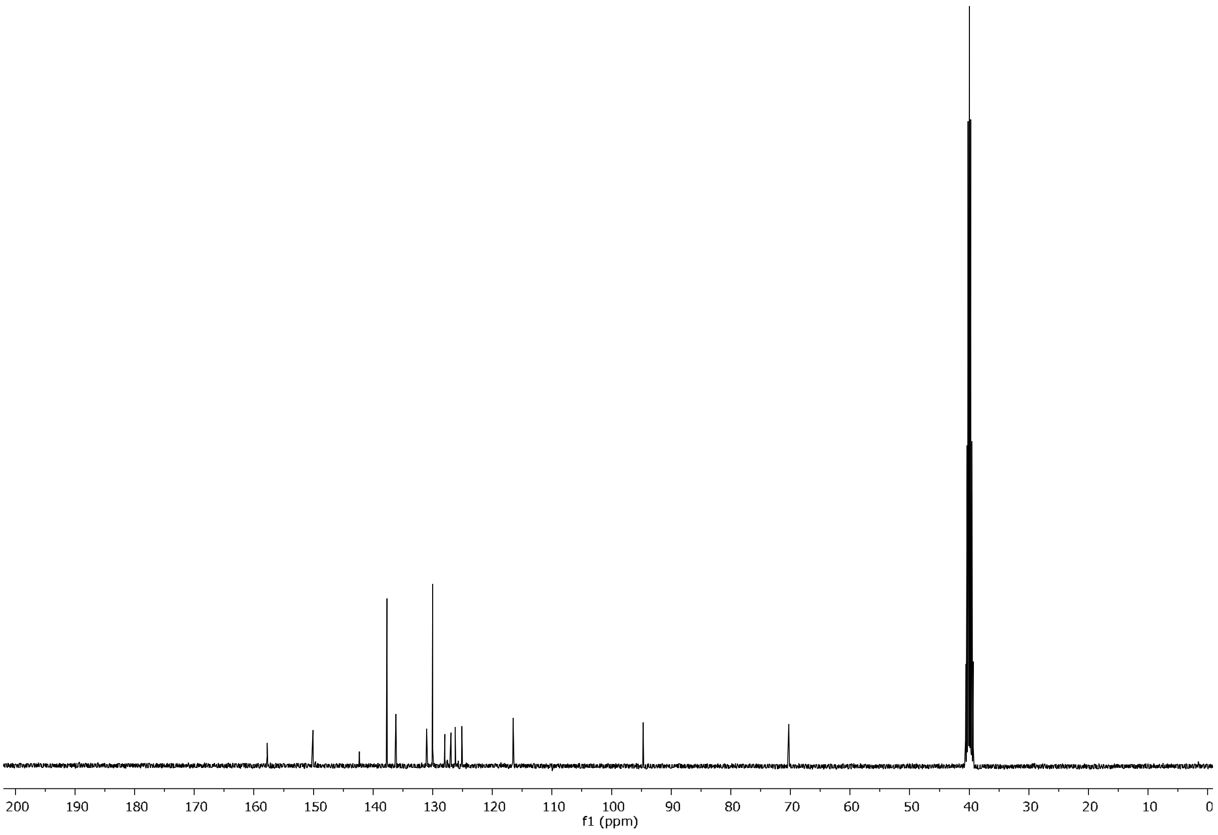
200 190 180 170 160 150 140 130 120 110 100 90 80 70 60 50 40 30 20 10 0
f1 (ppm)

Compound **18**

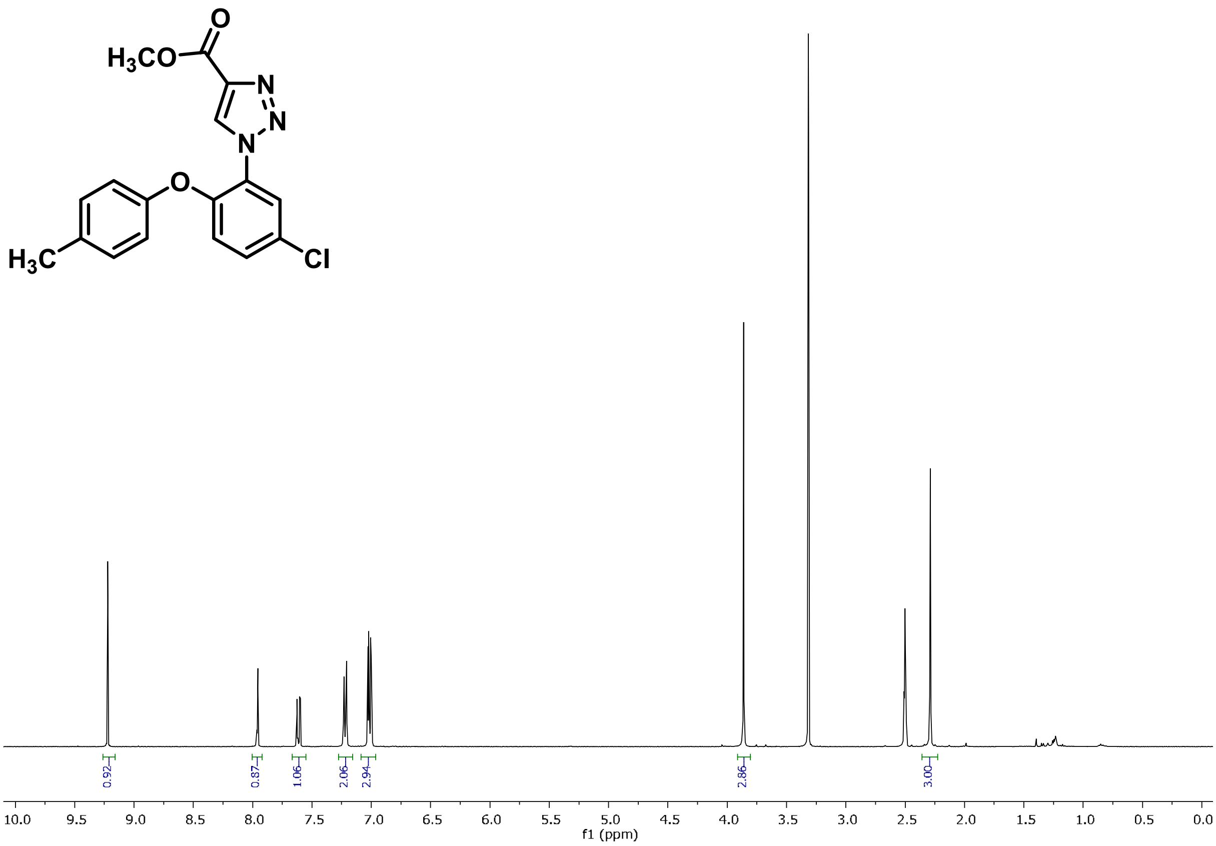
H3CO
O
N
N
N
O
H3C
Cl
0.92
0.87
1.06
2.06
2.94
2.86
3.00
10.0
9.5
9.0
8.5
8.0
7.5
7.0
6.5
6.0
5.5
5.0
4.5
4.0
3.5
3.0
2.5
2.0
1.5
1.0
0.5
0.0
f1 (ppm)

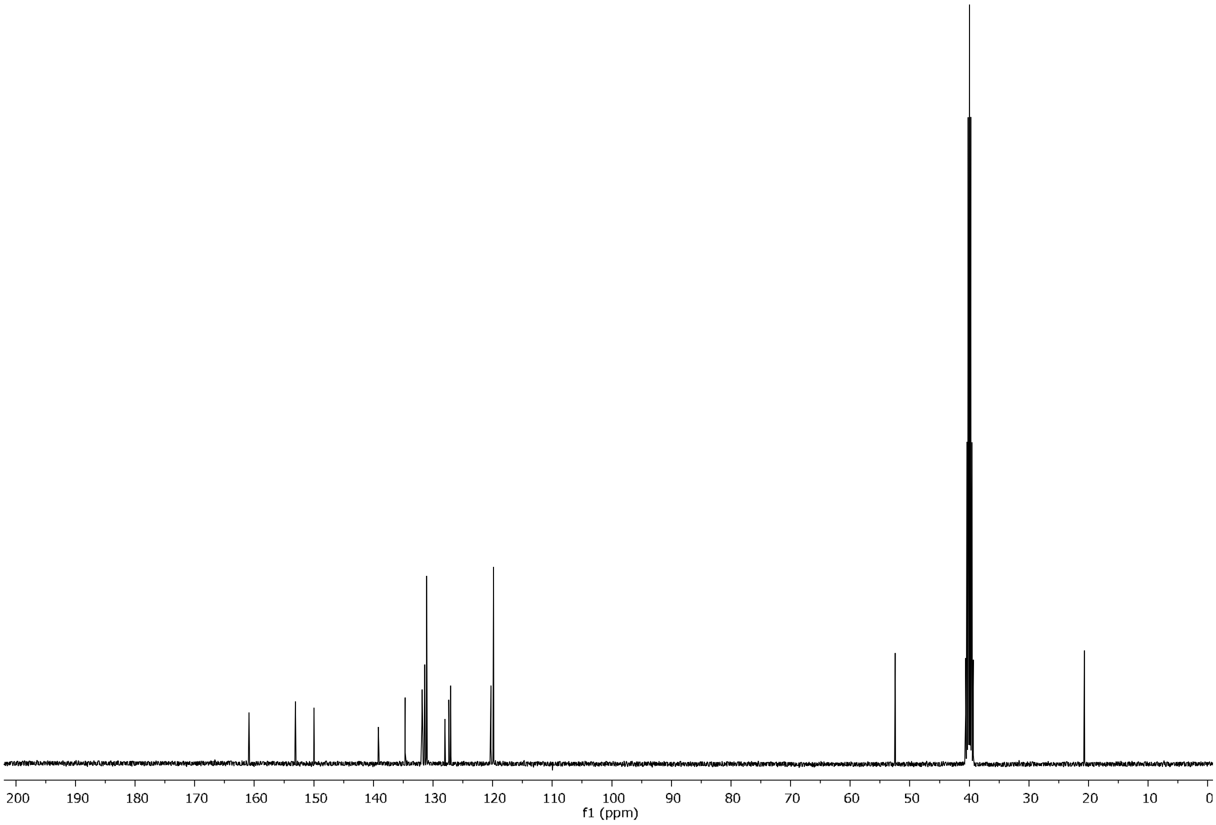
200
190
180
170
160
150
140
130
120
110
100
90
80
70
60
50
40
30
20
10
0
f1 (ppm)

Compound **19**

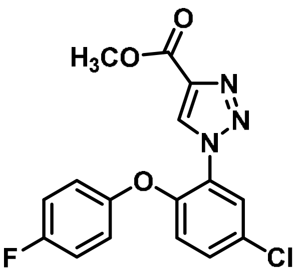

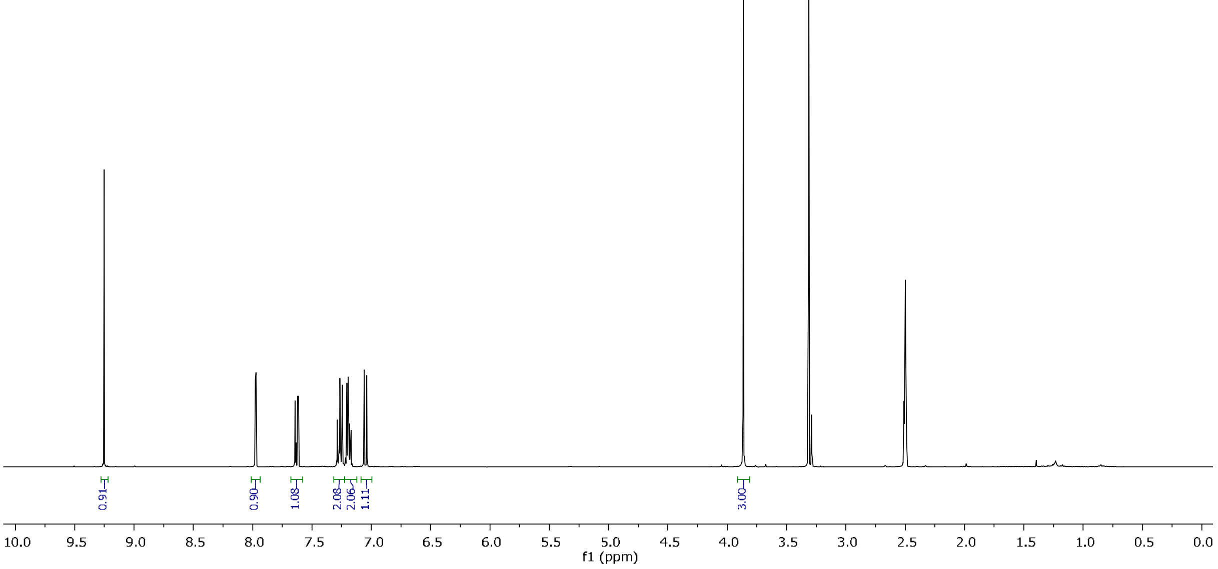

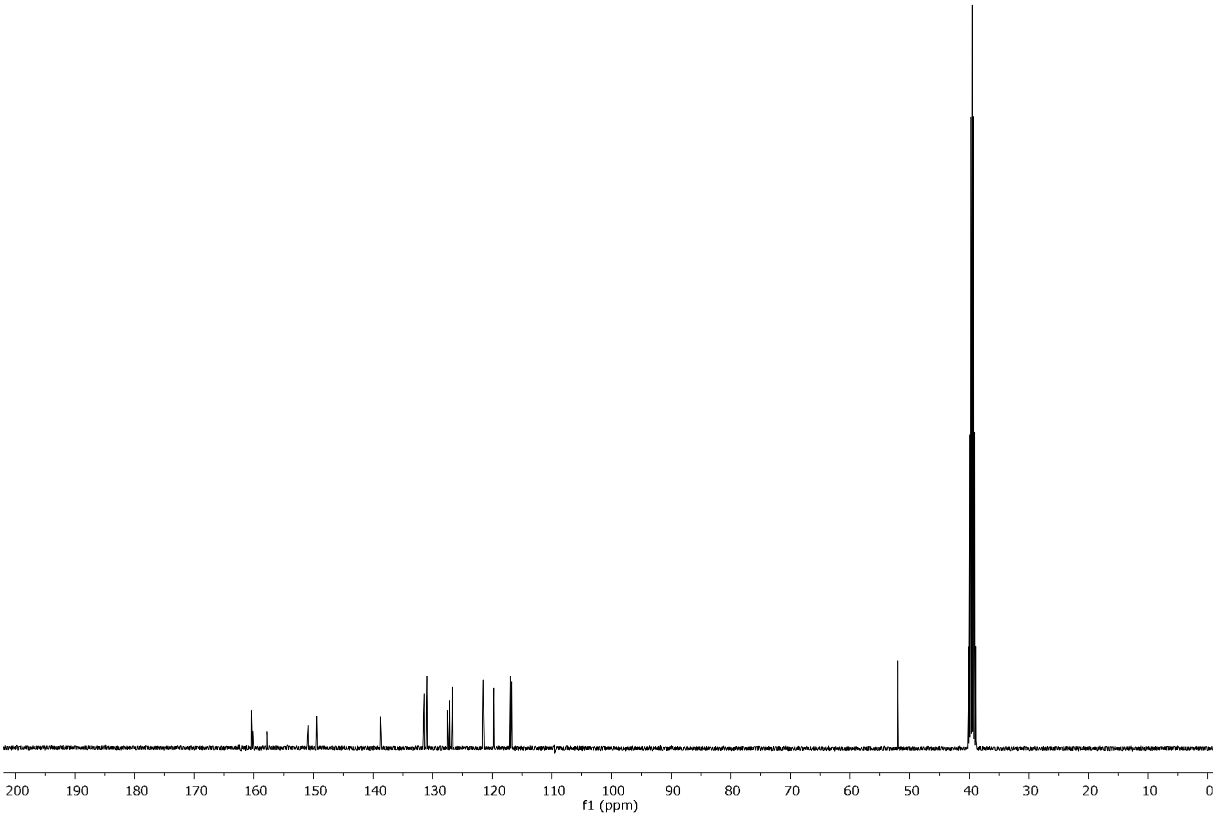

Compound **20**

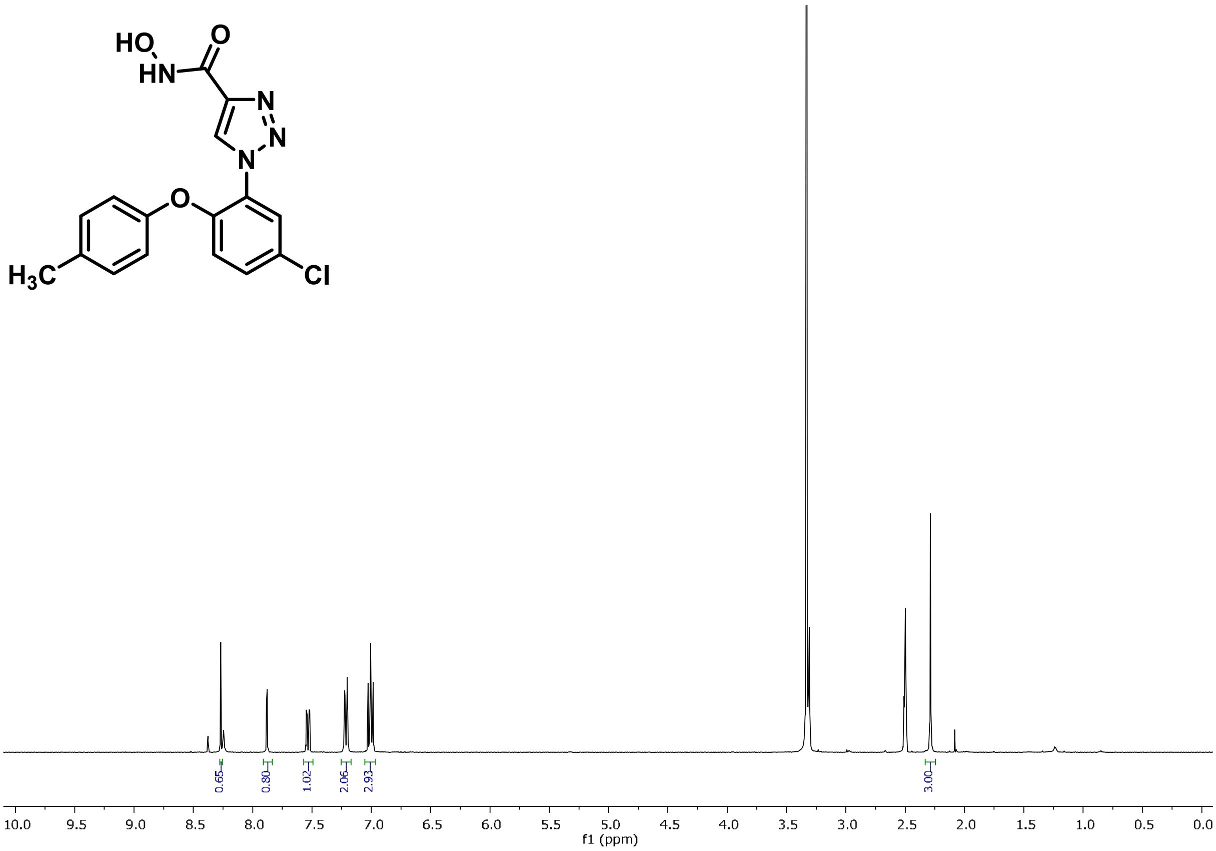

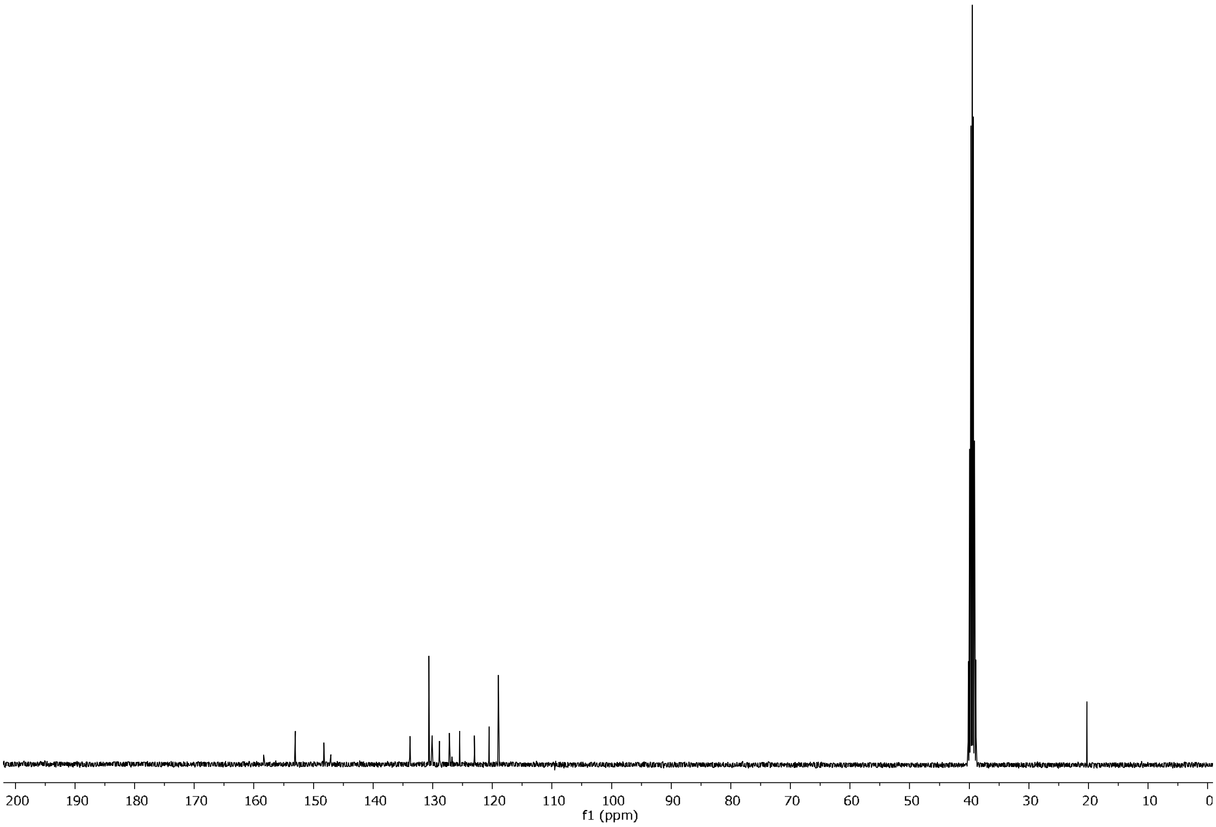

Compound **21**

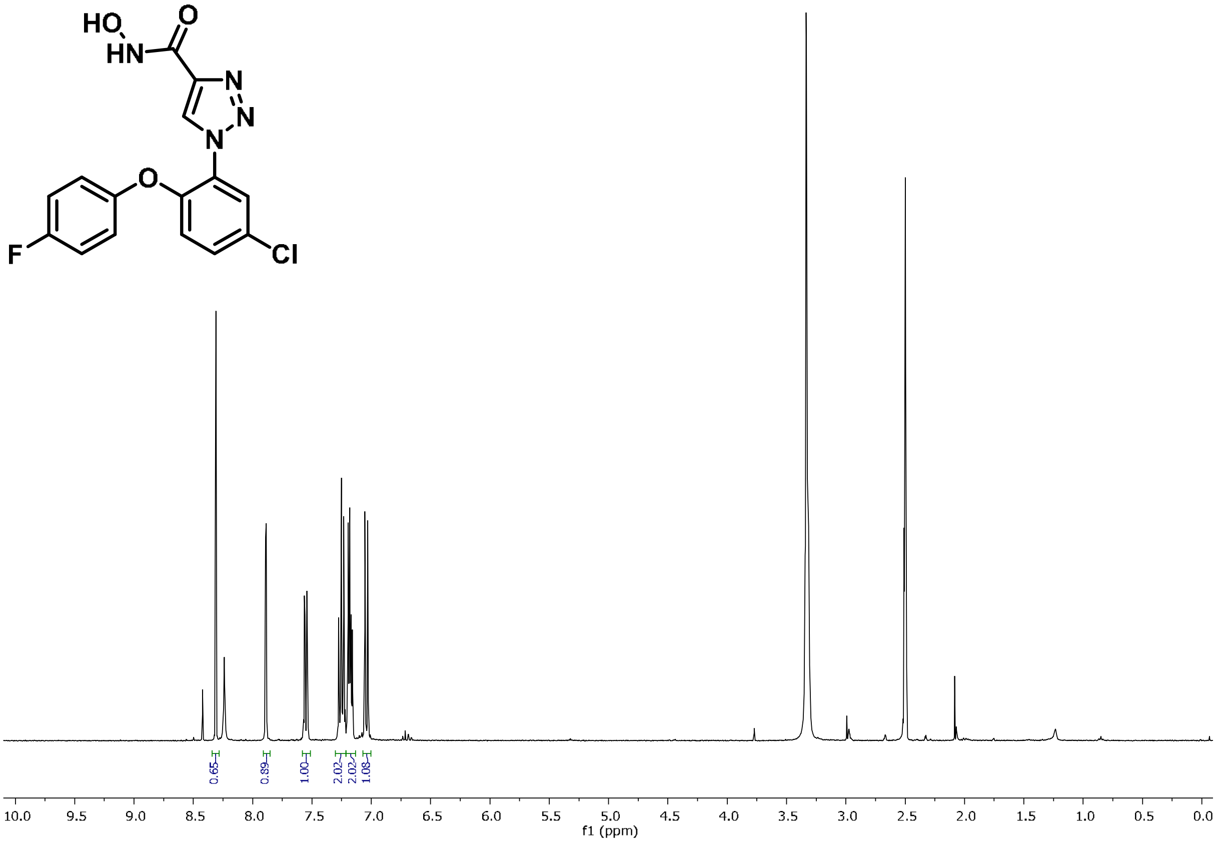
HO
HN
O
N
N
N
O
F
Cl
0.65
0.89
1.00
2.02
2.02
1.08
10.0 9.5 9.0 8.5 8.0 7.5 7.0 6.5 6.0 5.5 5.0 4.5 4.0 3.5 3.0 2.5 2.0 1.5 1.0 0.5 0.0
f1 (ppm)

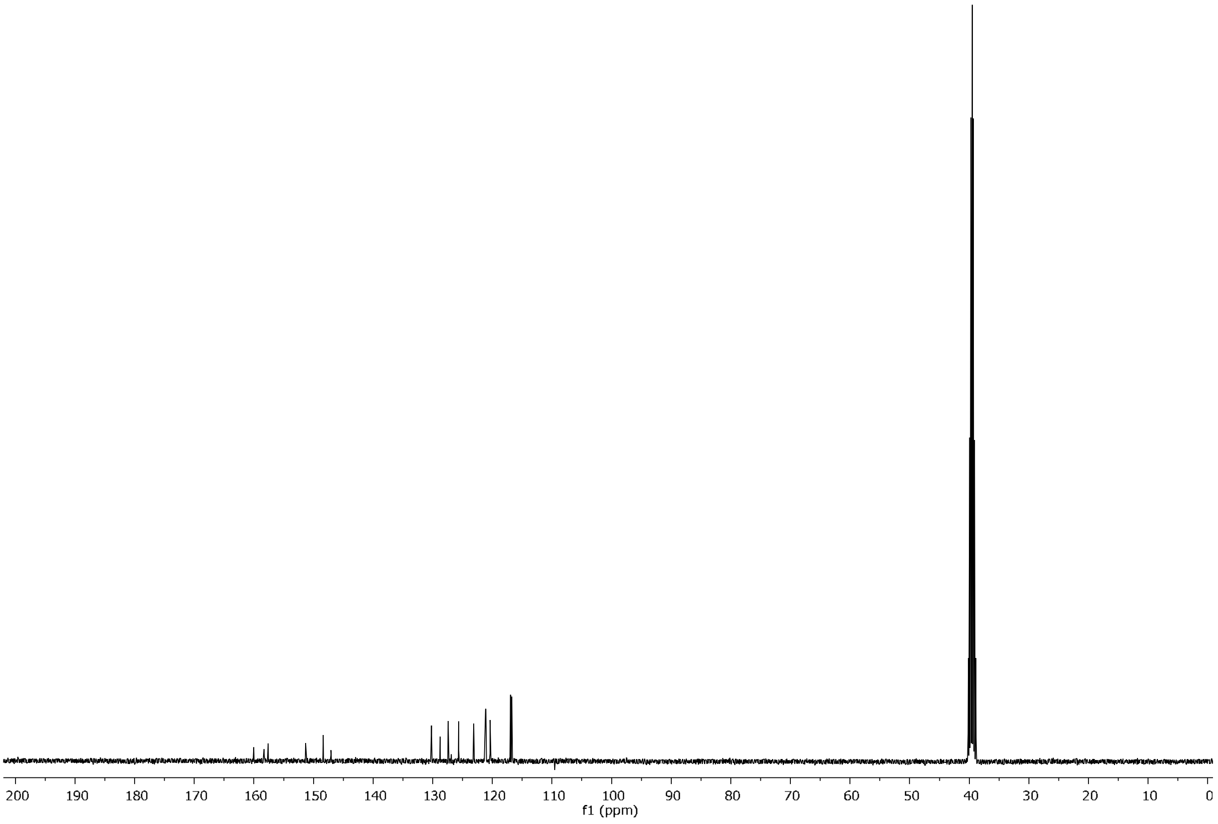
200 190 180 170 160 150 140 130 120 110 100 90 80 70 60 50 40 30 20 10 0
f1 (ppm)

Compound **24**

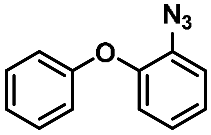

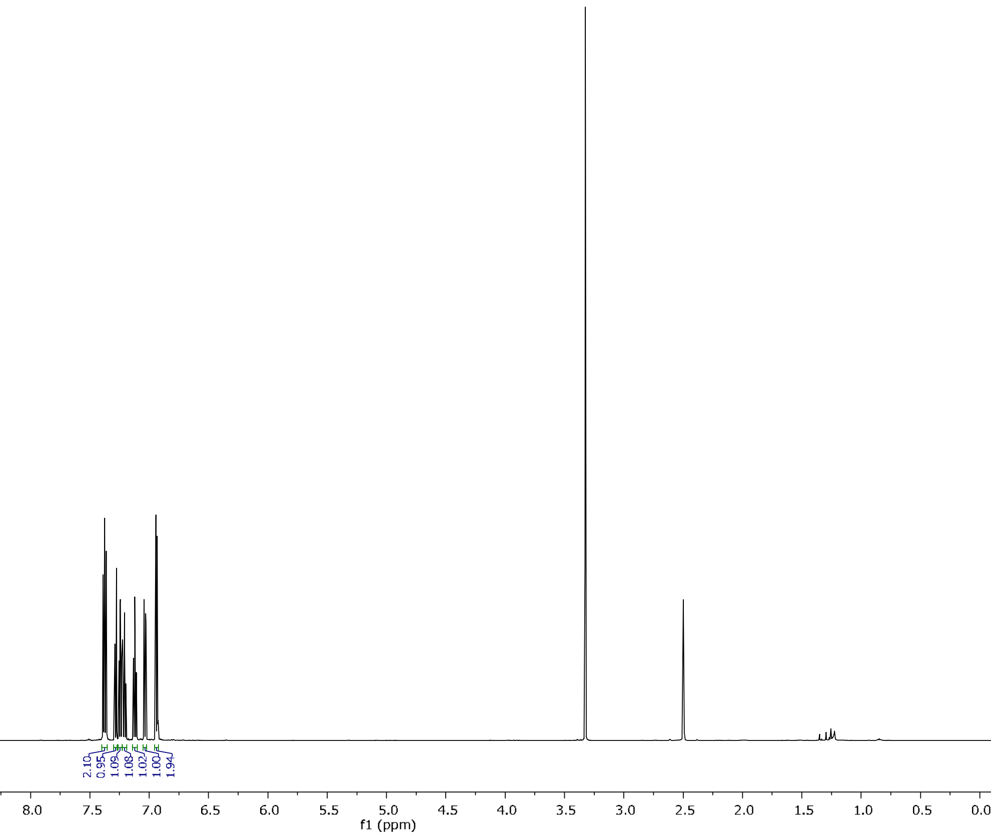

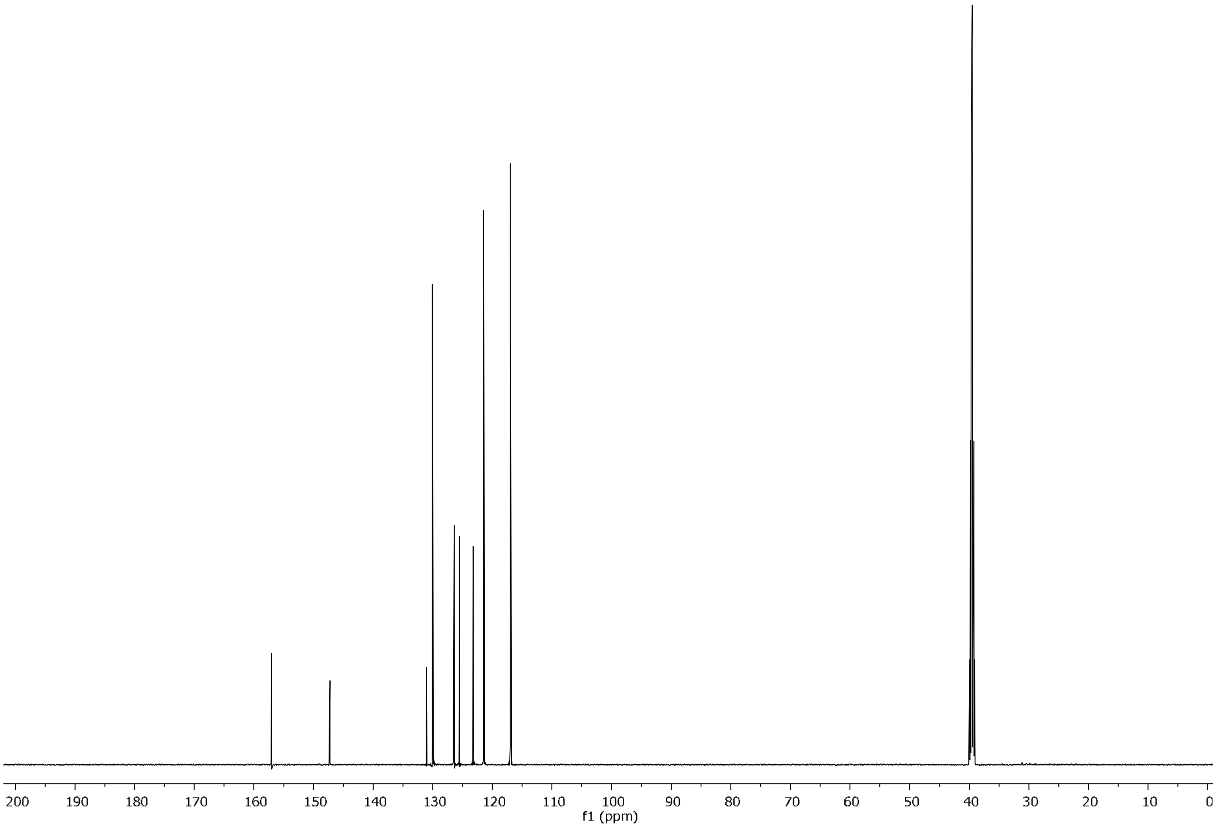

Compound **25**

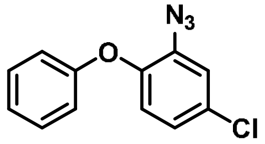

N3
O
Cl

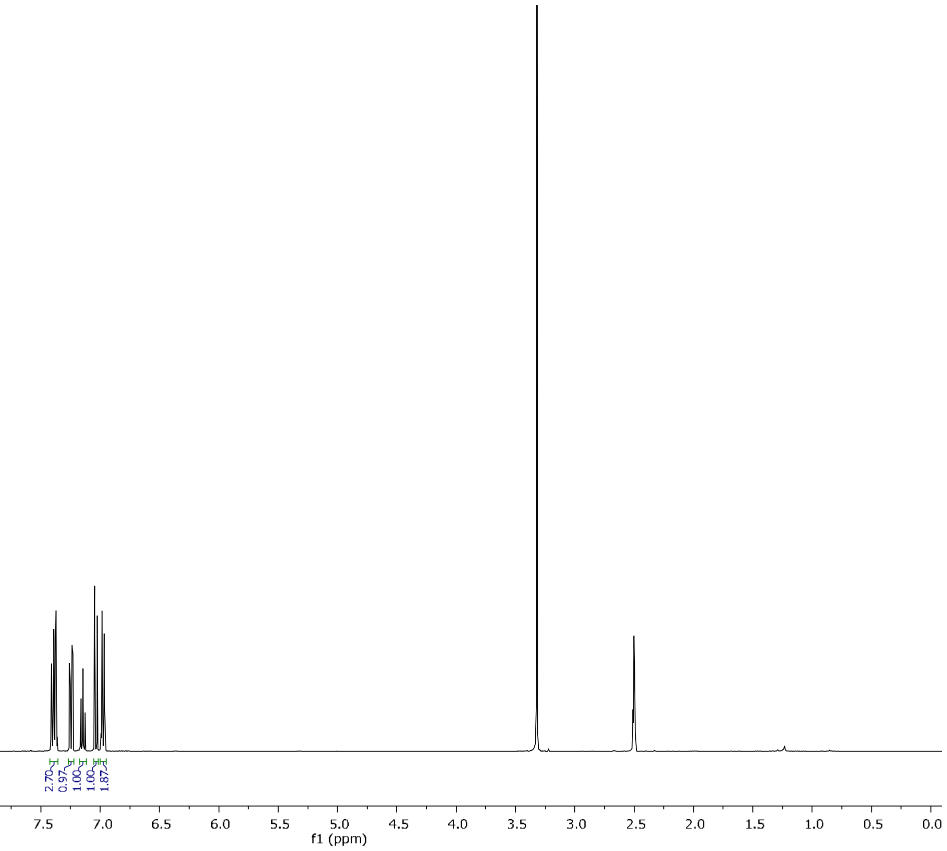

2.70
0.97
1.00
1.00
1.87
10.0
9.5
9.0
8.5
8.0
7.5
7.0
6.5
6.0
5.5
5.0
4.5
4.0
3.5
3.0
2.5
2.0
1.5
1.0
0.5
0.0
f1 (ppm)

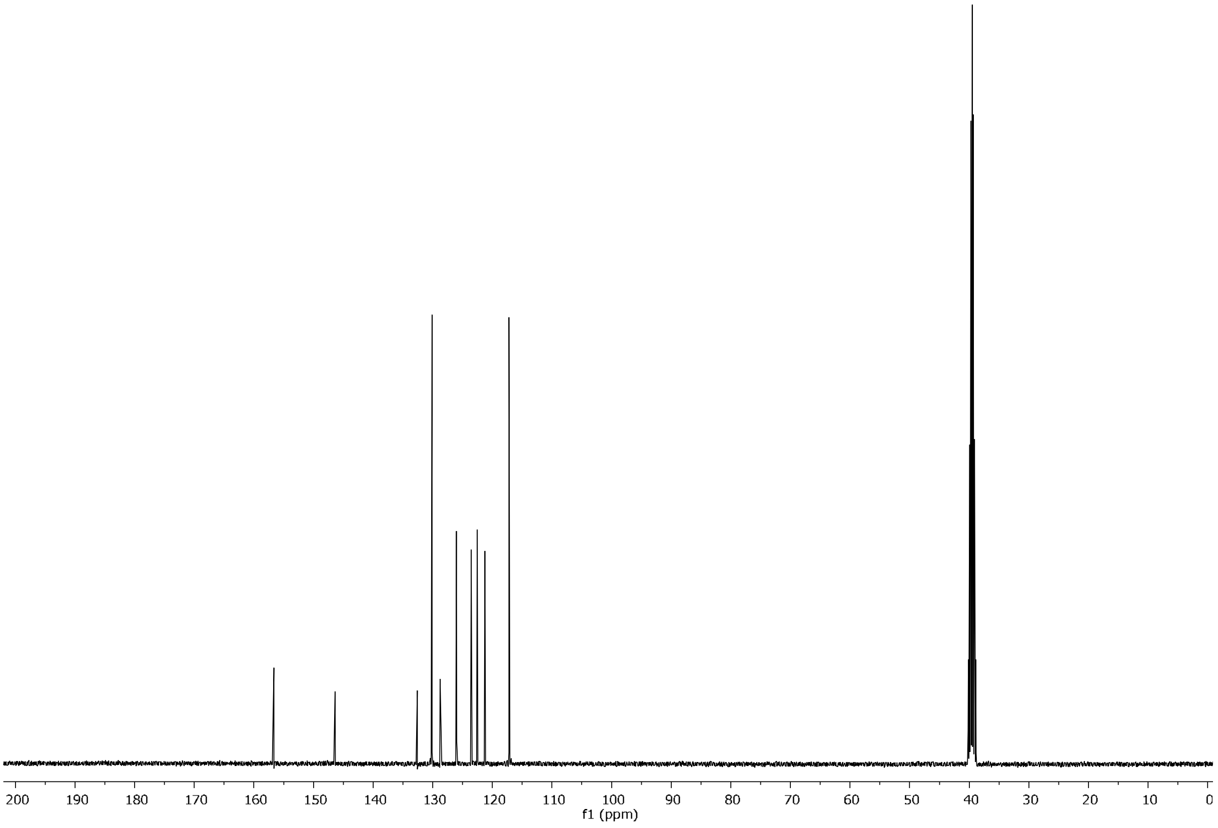

200
190
180
170
160
150
140
130
120
110
100
90
80
70
60
50
40
30
20
10
0
f1 (ppm)

Compound **26**

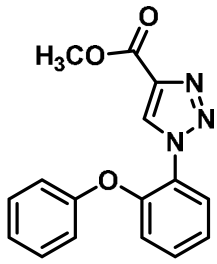

H3CO
O
N
N
N
O

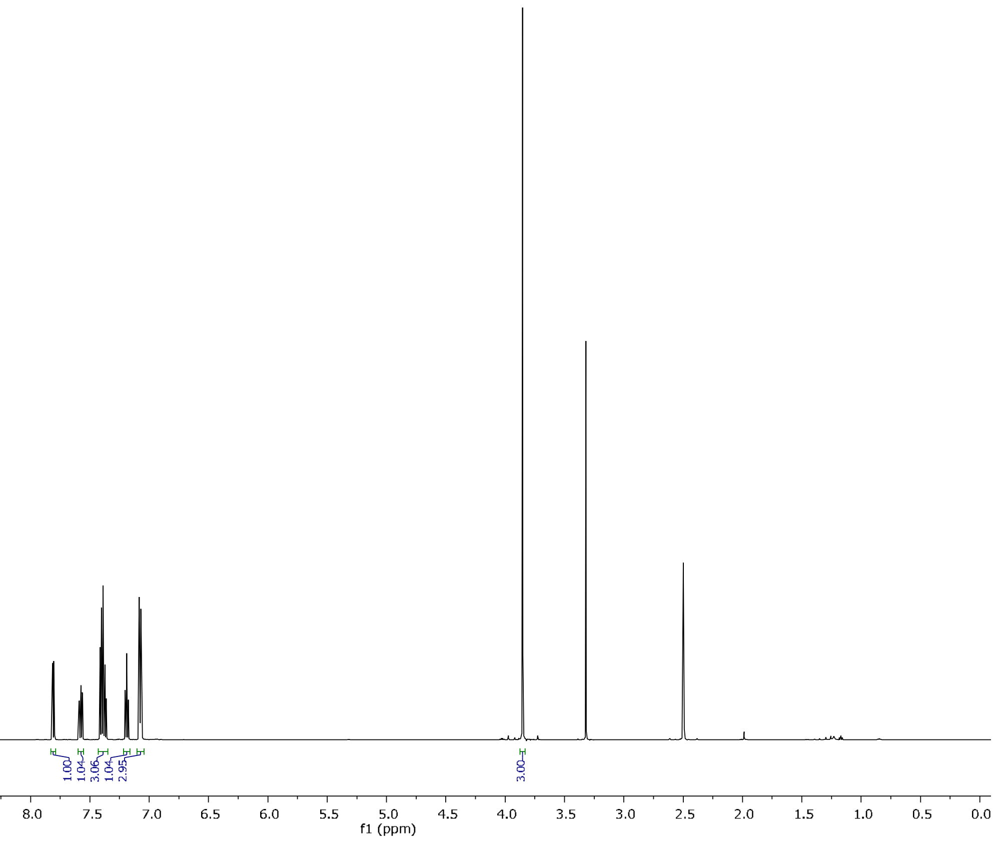

0.87
1.00
1.04
3.06
1.04
2.95
3.00
10.0
9.5
9.0
8.5
8.0
7.5
7.0
6.5
6.0
5.5
5.0
4.5
4.0
3.5
3.0
2.5
2.0
1.5
1.0
0.5
0.0
f1 (ppm)

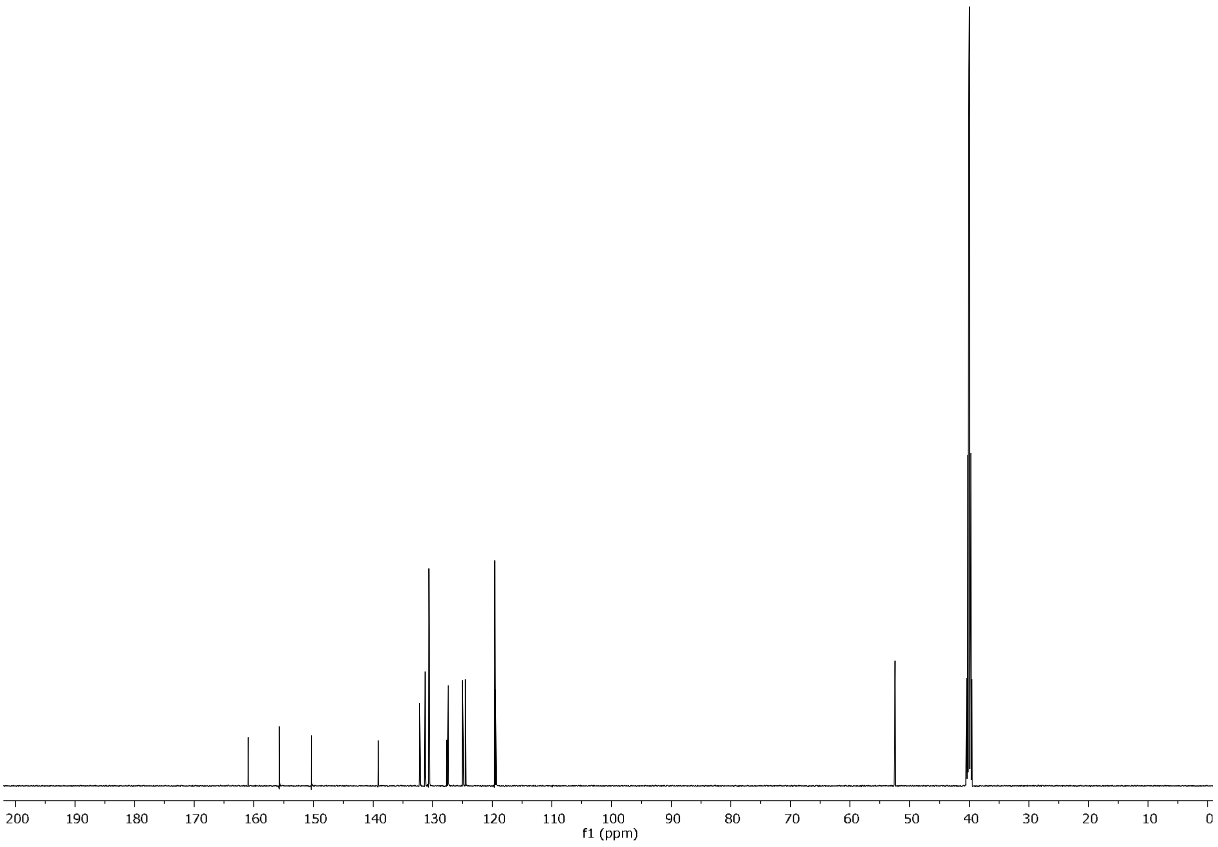

200
190
180
170
160
150
140
130
120
110
100
90
80
70
60
50
40
30
20
10
0
f1 (ppm)

Compound **27**

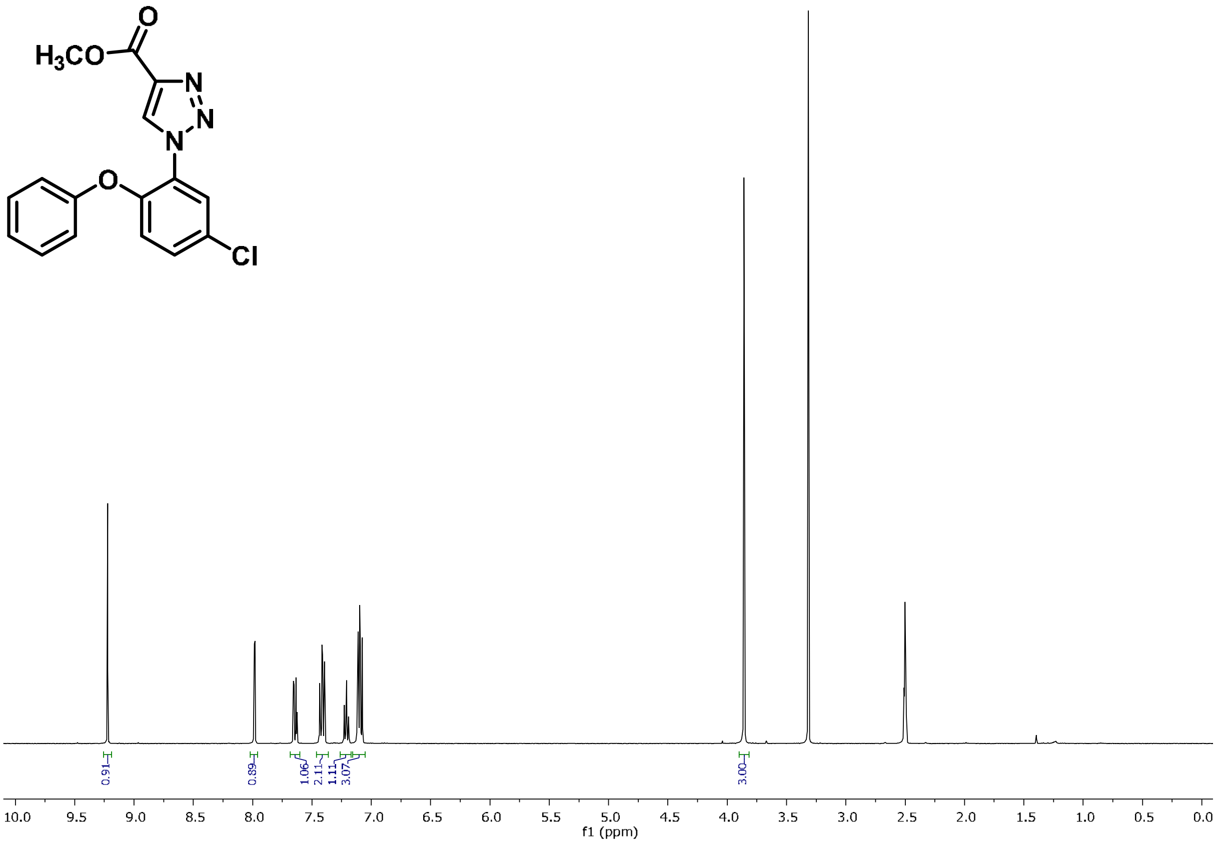
H3CO
O
N
N
N
O
Cl
0.91
0.89
1.06
2.11
1.11
3.07
3.00
10.0 9.5 9.0 8.5 8.0 7.5 7.0 6.5 6.0 5.5 5.0 4.5 4.0 3.5 3.0 2.5 2.0 1.5 1.0 0.5 0.0
f1 (ppm)

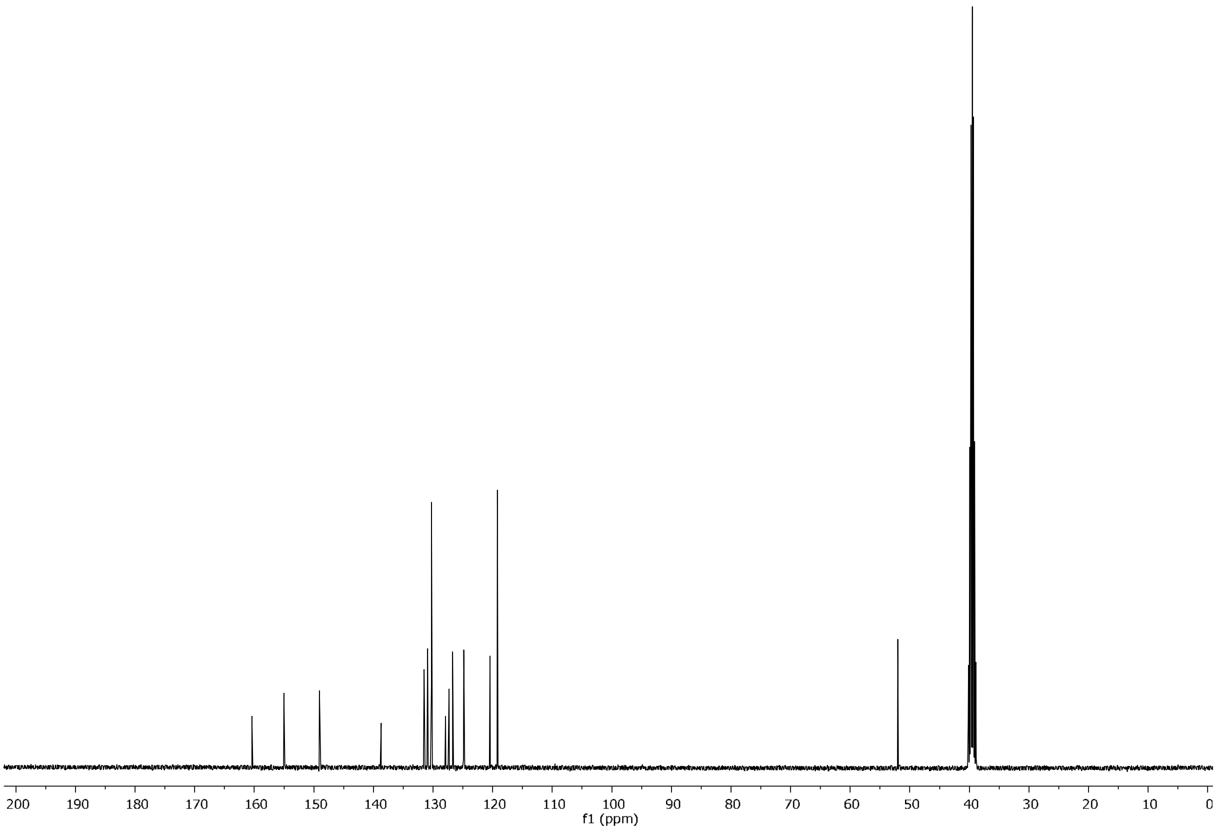
200 190 180 170 160 150 140 130 120 110 100 90 80 70 60 50 40 30 20 10 0
f1 (ppm)

Compound **28**

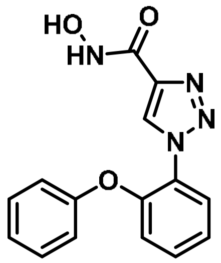

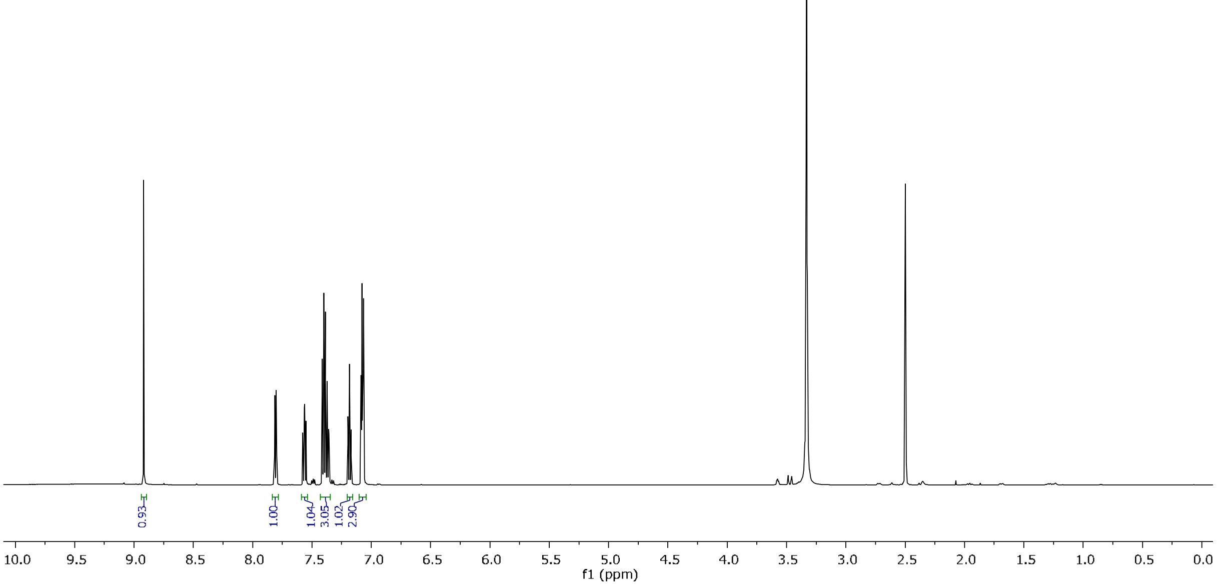
0.93
1.00
1.04
3.05
1.02
2.90
10.0 9.5 9.0 8.5 8.0 7.5 7.0 6.5 6.0 5.5 5.0 4.5 4.0 3.5 3.0 2.5 2.0 1.5 1.0 0.5 0.0
f1 (ppm)

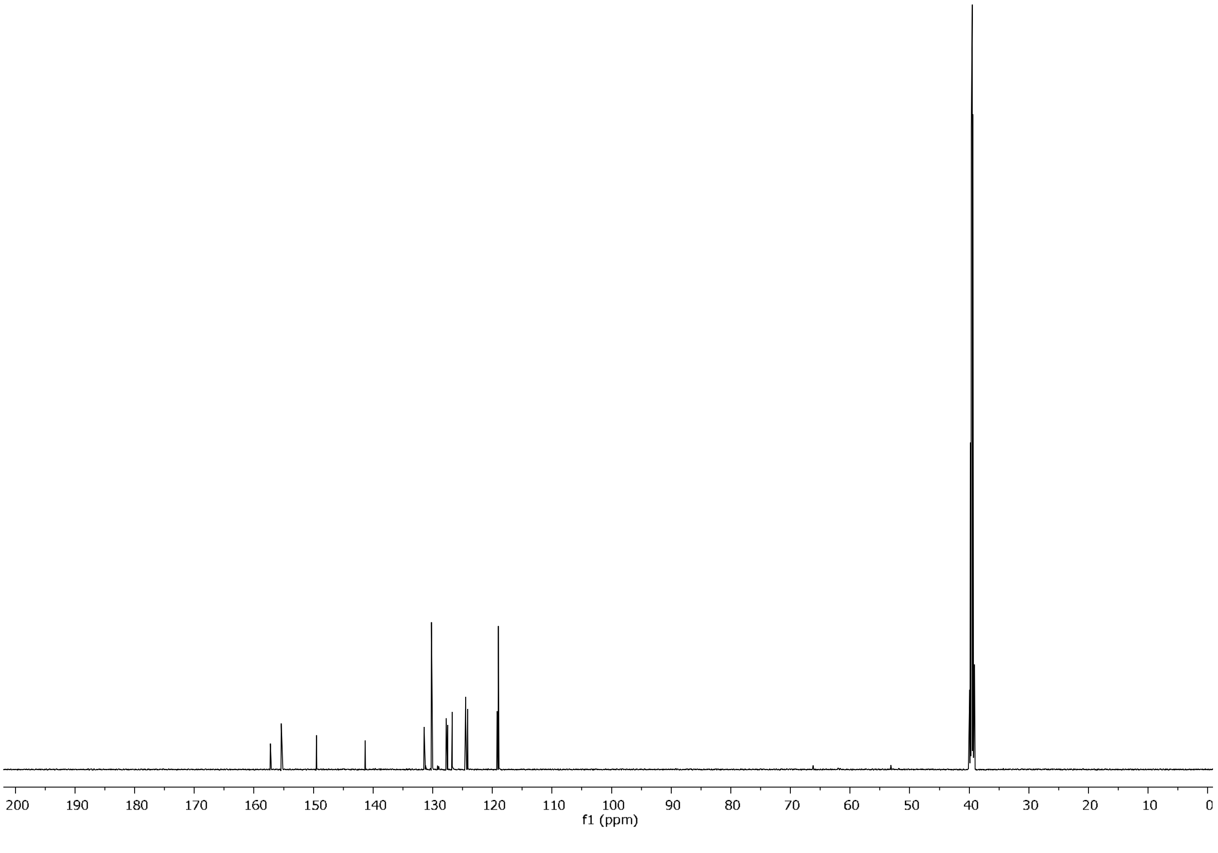
200 190 180 170 160 150 140 130 120 110 100 90 80 70 60 50 40 30 20 10 0
f1 (ppm)

Compound **29**

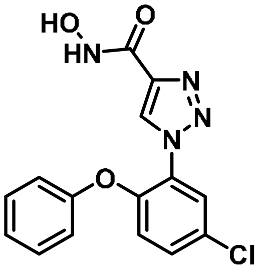
HO
HN
O
N
N
N
O
Cl

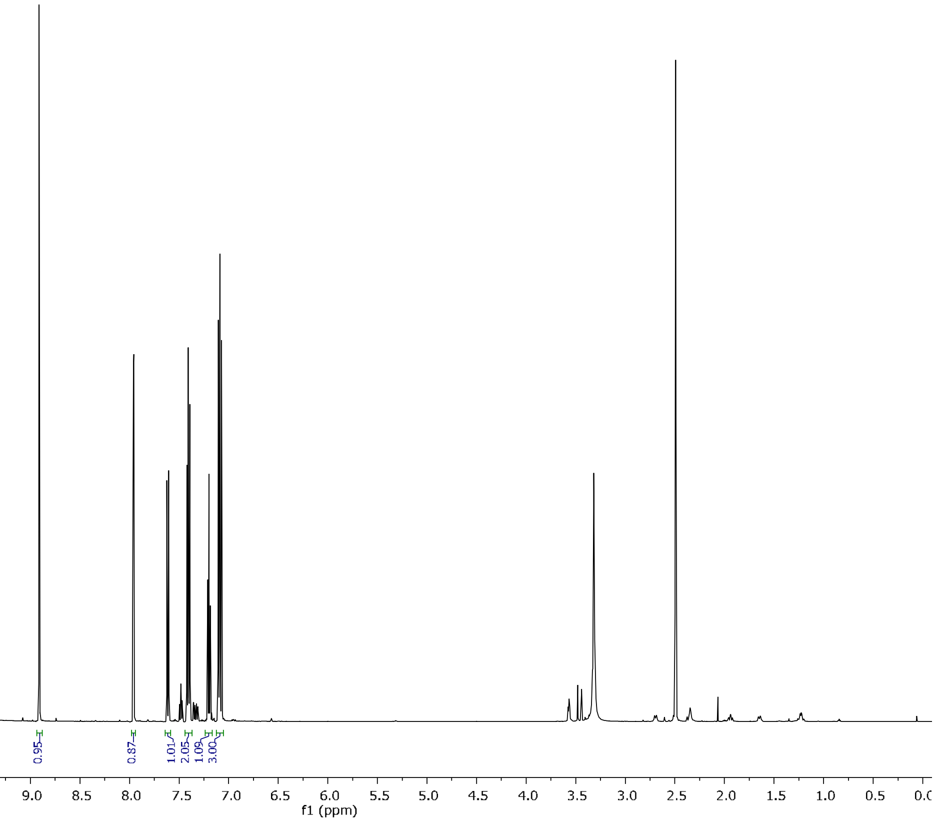
0.95
0.87
1.01
2.05
1.09
3.00
12.0 11.5 11.0 10.5 10.0 9.5 9.0 8.5 8.0 7.5 7.0 6.5 6.0 5.5 5.0 4.5 4.0 3.5 3.0 2.5 2.0 1.5 1.0 0.5 0.0
f1 (ppm)

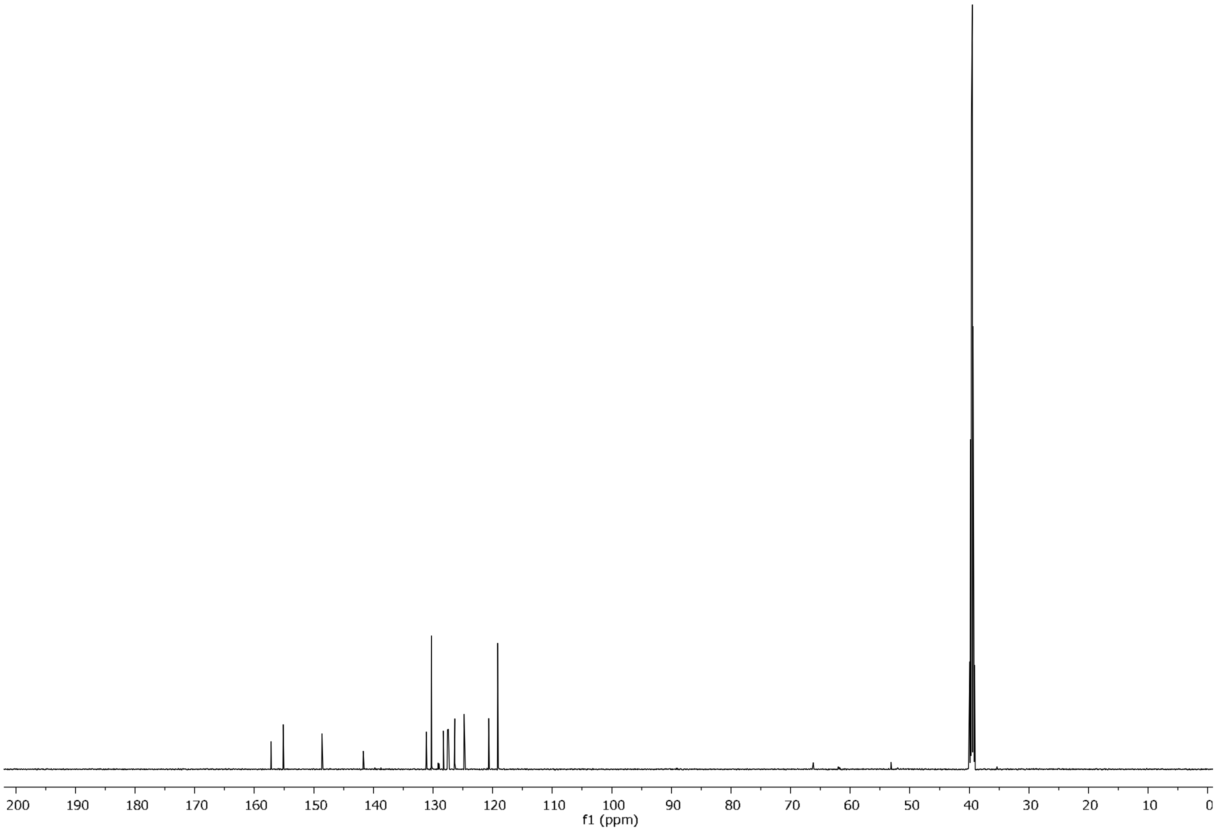
200 190 180 170 160 150 140 130 120 110 100 90 80 70 60 50 40 30 20 10 0
f1 (ppm)

Compound **31**

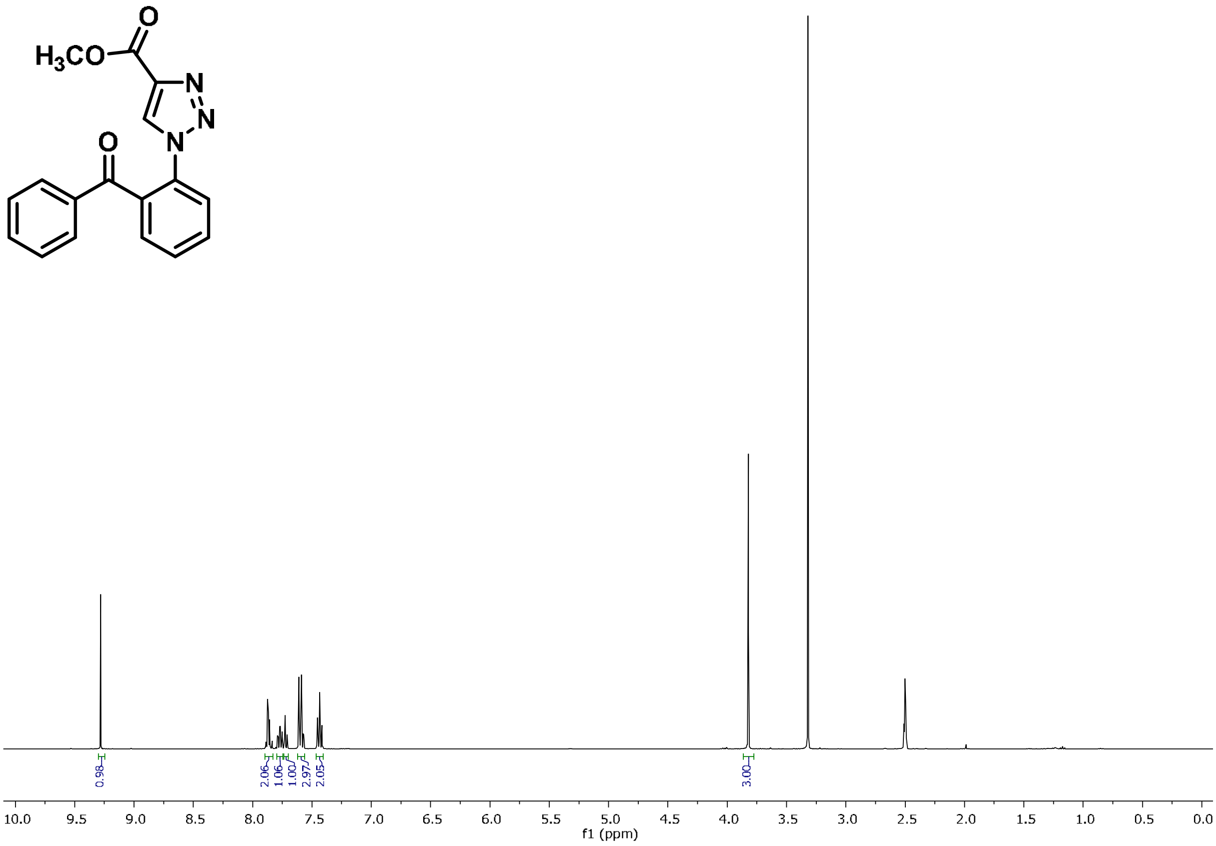
O
H3CO
N
N
N
O
0.98
2.06
1.06
1.00
2.97
2.05
3.00
10.0 9.5 9.0 8.5 8.0 7.5 7.0 6.5 6.0 5.5 5.0 4.5 4.0 3.5 3.0 2.5 2.0 1.5 1.0 0.5 0.0
f1 (ppm)

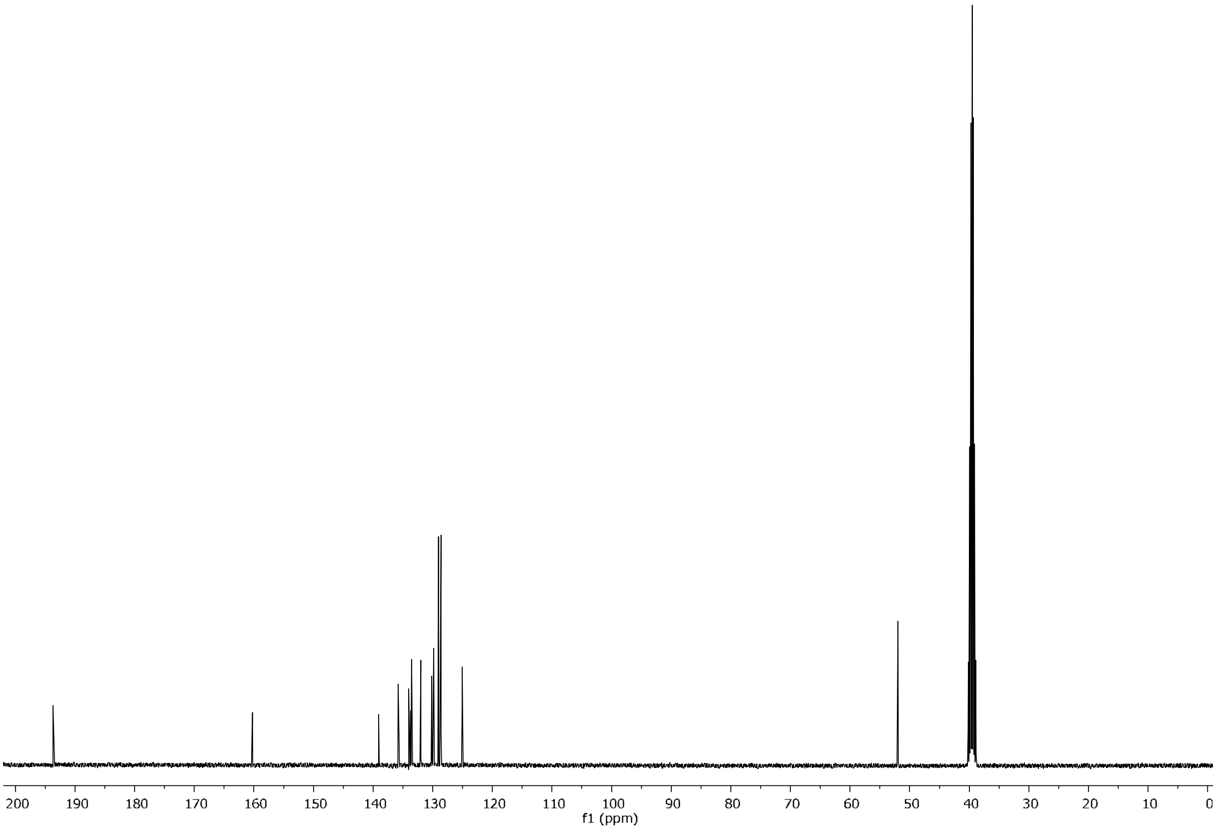
200 190 180 170 160 150 140 130 120 110 100 90 80 70 60 50 40 30 20 10 0
f1 (ppm)

Compound **32**

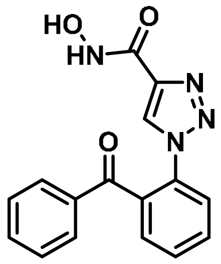
HO
HN
O
N
N
N
O

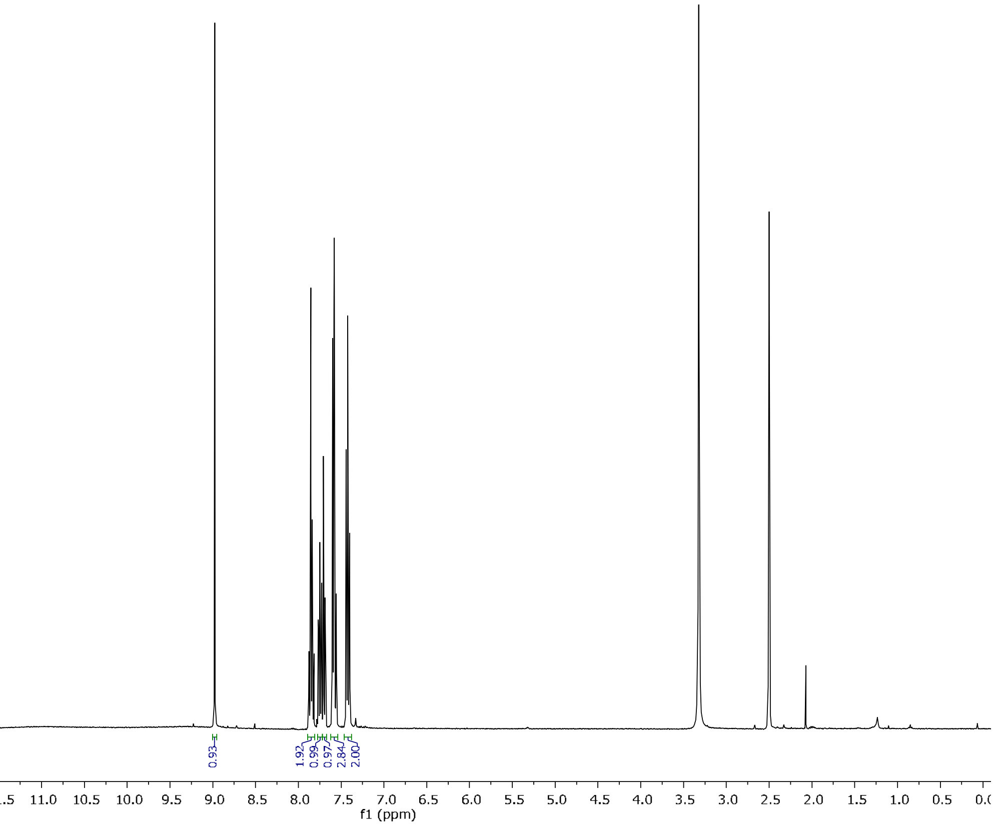
0.93
1.92
0.99
0.97
2.84
2.00
4.0 13.5 13.0 12.5 12.0 11.5 11.0 10.5 10.0 9.5 9.0 8.5 8.0 7.5 7.0 6.5 6.0 5.5 5.0 4.5 4.0 3.5 3.0 2.5 2.0 1.5 1.0 0.5 0.0
f1 (ppm)

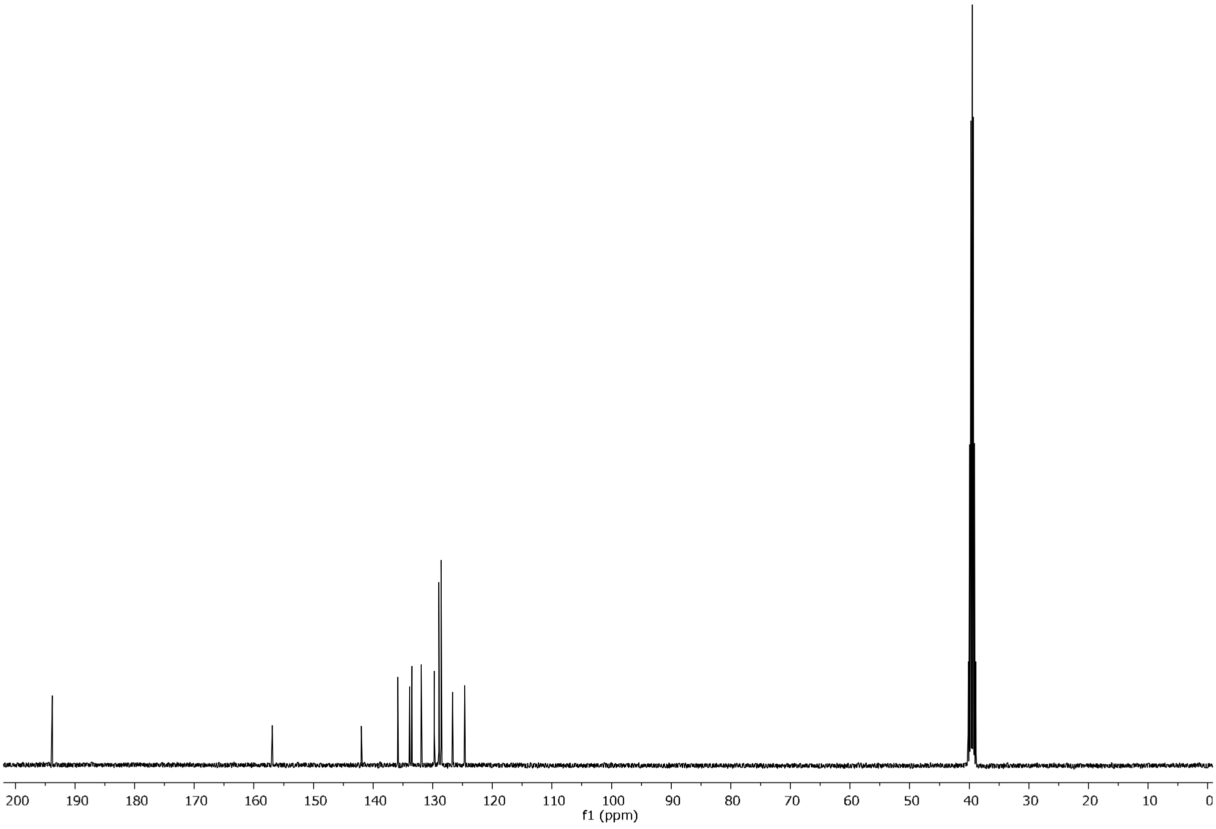
200 190 180 170 160 150 140 130 120 110 100 90 80 70 60 50 40 30 20 10 0
f1 (ppm)
